# Eplontersen with and without background transthyretin stabilizers in transthyretin amyloid cardiomyopathy: secondary analysis of a phase 3, randomized controlled trial

**DOI:** 10.1038/s41591-026-04670-6

**Published:** 2026-08-30

**Authors:** Pablo García-Pavía, Francesco Cappelli, Margot K. Davis, Marianna Fontana, Julian D. Gillmore, Mazen Hanna, Ahmad Masri, Laura Obici, Scott D. Solomon, Brett W. Sperry, Nobuhiro Tahara, Márcia Waddington-Cruz, Kurt Boman, Sarah A. M. Cuddy, Philippe Debonnaire, Nowell M. Fine, Piotr Podolec, Ali Yilmaz, Tom Conrad, Qingqing Yang, Brian L. Claggett, Jersey Chen, Sotirios Tsimikas, Mathew S. Maurer

**Affiliations:** 1 Department of Cardiology, Hospital Universitario Puerta de Hierro Majadahonda, IDIPHIM, CIBERCV; Centro Nacional de Investigaciones Cardiovasculares (CNIC), Madrid, Spain; 2 Tuscan Regional Amyloidosis Center, Department of Clinical and Experimental Medicine, Careggi University Hospital, Florence, Italy; 3 Division of Cardiology, University of British Columbia, Vancouver, British Columbia Canada; 4 National Amyloidosis Centre, Division of Medicine, University College London, Royal Free Hospital, London, UK; 5 Amyloidosis Center, Department of Cardiovascular Medicine, Cleveland Clinic, Cleveland, OH USA; 6 Division of Cardiovascular Medicine, Oregon Health and Sciences University, Portland, OR USA; 7 Amyloidosis Research and Treatment Centre, IRCCS Fondazione Policlinico San Matteo, Pavia, Italy; 8 Cardiovascular Division, Brigham and Women’s Hospital, Boston, MA USA; 9 Saint Luke’s Mid America Heart Institute, Kansas City, MO USA; 10 Division of Cardiovascular Medicine, Department of Medicine, Kurume University School of Medicine, Kurume, Japan; 11 Centro de Paramiloidose Antônio Rodrigues de Mello (CEPARM), Amyloidosis Center, University Hospital, Federal University of Rio de Janeiro, Rio de Janeiro, Brazil; 12 Amyloidosis Center, Complexo Hospitalar Américas-Samaritano-Vitória, Rio de Janeiro, Brazil; 13 Research Unit, Skellefteå County Hospital, Department of Public Health and Clinical Medicine, Umeå University, Umeå, Sweden; 14 Amyloidosis Program, Division of Cardiovascular Medicine, Department of Medicine, Brigham and Women’s Hospital, Harvard Medical School, Boston, MA USA; 15 Cardiology Department, AZ Sint-Jan Brugge, Bruges, Belgium; 16 Libin Cardiovascular Institute, Cumming School of Medicine, University of Calgary, Calgary, Alberta Canada; 17 Department of Cardiac and Vascular Diseases, Jagiellonian University Medical College, John Paul II Hospital, Krakow, Poland; 18 Division of Cardiovascular Imaging, Department of Cardiology I, University Hospital Münster, Münster, Germany; 19 Late-Stage Development, Cardiovascular, Renal, and Metabolism (CVRM), BioPharmaceuticals R&D, AstraZeneca, Gaithersburg, MD USA; 20 Ionis Pharmaceuticals Inc., Carlsbad, CA USA; 21 Columbia University Irving Medical Center, New York, NY USA

**Keywords:** Cardiomyopathies, Randomized controlled trials

## Abstract

Transthyretin (TTR) amyloid cardiomyopathy (ATTR-CM) therapies include both TTR gene silencers and TTR stabilizers. The role of TTR gene silencers added to background TTR stabilizer therapy is unknown, and the effects of silencer treatment in the absence of stabilizer use are unclear. In the CARDIO-TTRansform study, 1,432 patients (*n* = 135 (9.4%) women and *n* = 1,297 (90.6%) men) with ATTR-CM were randomized (1:1) and treated with the antisense oligonucleotide eplontersen (45 mg every 4 weeks) that targets TTR or with placebo for up to 140 weeks. At baseline, 57% of the patients were taking TTR stabilizers. In the overall study, treatment with eplontersen did not reduce the primary composite endpoint. Here, in a prespecified analysis, we show that the primary endpoint (a composite of cardiovascular mortality and recurrent cardiovascular events) was modified by baseline stabilizer use (*P*_interaction_ = 0.017). Benefits were observed in those patients not on stabilizers at baseline (rate ratio (RR) = 0.71, 95% confidence interval (CI) 0.54 to 0.93, *P* = 0.012), whereas no benefits were observed in those on stabilizers at baseline (RR = 1.14, 95% CI 0.85 to 1.53, *P* = 0.39). The safety profile of eplontersen was favorable, irrespective of baseline stabilizer use. Treatment with eplontersen versus placebo demonstrated a clinically meaningful benefit among patients not receiving background stabilizers but provided no additional clinical benefit in those on background stabilizer therapy. These findings of a strong treatment effect modification may offer insight for therapeutic decision-making in clinical practice. ClinicalTrials.gov registration: NCT04136171.

## Abstract (web summary)

As presented at the 2026 ESC Congress, in a secondary analysis of the CARDIO-TTRansform trial testing the transthyretin-targeting antisense oligonucleotide eplontersen in patients with transthyretin amyloid cardiomyopathy, a beneficial effect was observed in patients who were not on transthyretin stabilizers at baseline, but not in those who were on stabilizers.

## Main

Transthyretin amyloid cardiomyopathy (ATTR-CM) is a progressive infiltrative cardiomyopathy caused by misfolded monomers or oligomers of the transthyretin (TTR) protein being deposited in the myocardium as amyloid fibrils^1–3^. Deposition of these amyloid fibrils within the myocardial extracellular matrix results in cardiac remodeling with concentric wall thickening and frequent arrhythmias from infiltration of the conduction system^3,4^. Progressive cardiac dysfunction results in heart failure symptoms, accompanied by loss of exercise tolerance, severe reduction in quality of life, frequent hospitalizations and high mortality^5–7^. Before the advent of disease-modifying therapy, ATTR-CM was associated with a median survival of 2–6 years^8,9^.

Current approved disease-modifying therapies for ATTR-CM include the TTR stabilizers tafamidis^10,11^ and acoramidis^12,13^, which bind circulating TTR tetramers to prevent their dissociation into monomers or oligomers and subsequent amyloid formation, and the TTR gene silencer vutrisiran, an RNA interference therapy^14^ that decreases hepatic TTR production. Eplontersen is an antisense oligonucleotide TTR gene silencer that reduces hepatic TTR production, thereby decreasing circulating TTR levels, and has been shown to reduce the progression of polyneuropathy manifestations and improve patients’ quality of life compared with a historical placebo in hereditary TTR amyloidosis^15^.

Eplontersen for the treatment of ATTR-CM was evaluated in the CARDIO-TTRansform trial (NCT04136171), the largest phase 3, randomized, placebo-controlled trial conducted in ATTR-CM. In CARDIO-TTRansform, eplontersen did not reduce the primary composite endpoint of cardiovascular (CV) mortality and recurrent CV events despite achieving substantial TTR suppression and nominal improvements versus placebo in functional capacity and health-related quality of life^16^. The use of TTR stabilizers was permitted with no restrictions throughout the trial, and patients with baseline use of stabilizers were assessed as a prespecified subgroup.

The results of this trial provide an opportunity to further understand the relationship between TTR gene silencing and TTR stabilization on patient outcomes in ATTR-CM in a contemporary patient population. Accordingly, the focus of this prespecified analysis was to elucidate the effects of eplontersen as a monotherapy and with background treatment with TTR stabilizers in the CARDIO-TTRansform trial.

## Results

### Patient characteristics

**Extended Data Fig. 1:**
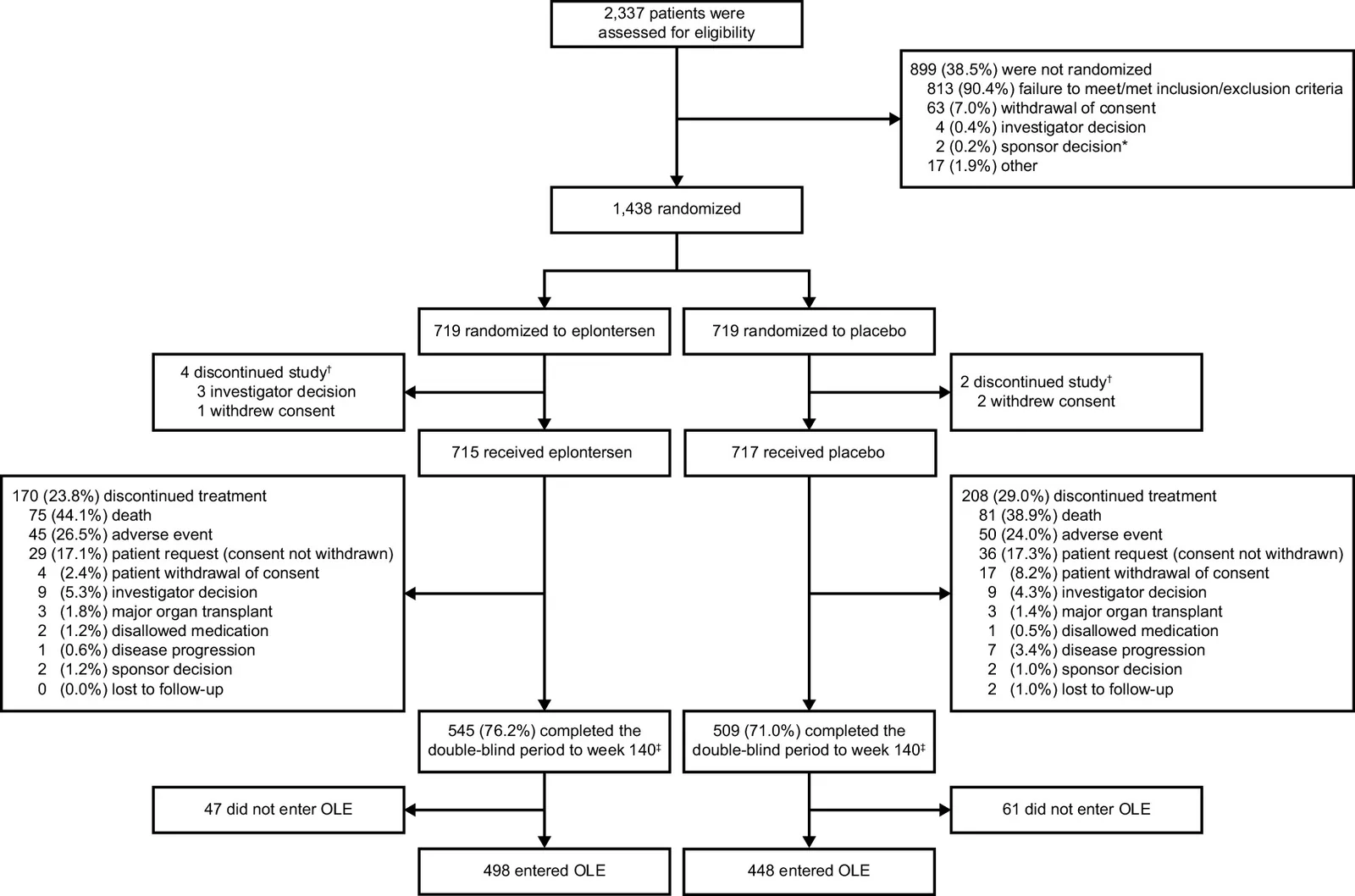
CARDIO-TTRansform CONSORT diagram. CONSORT flow chart of the phases of the CARDIO-TTRansform trial^16^. The percentages of patients who completed treatment and for patients who discontinued treatment are based on the number of treated patients. The percentages for the reasons for treatment discontinuation are based on the number of patients who discontinued treatment. *One patient did not meet the enrollment cut-off deadline; one patient was not able to be randomized within the study timeline. †Three patients withdrew consent before receiving any treatment, and three were removed from the study at their initial visits per investigator decision. No data/follow-up are available for these patients. ‡Patients who completed the double-blind period and who did not enroll in the OLE study were eligible to complete an additional 20-week follow-up period (up to week 160). OLE denotes open-label extension.

**Extended Data Fig. 2:**
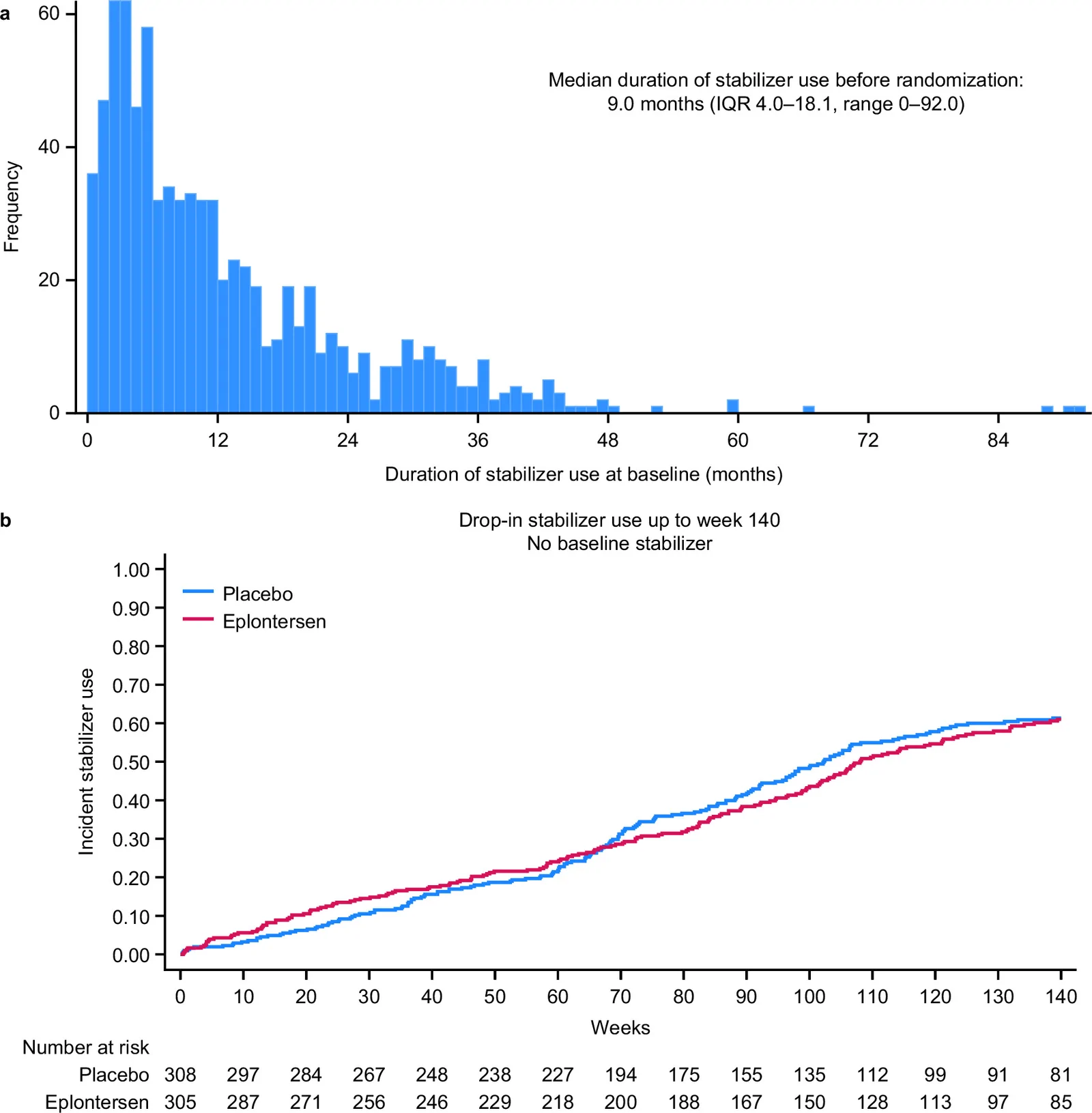
Median duration of stabilizer therapy before randomization and drop-in use of TTR stabilizers during the trial. **a**,**b**, Prior TTR stabilizer use (**a**) and drop-in use of TTR stabilizers during the trial in patients not receiving TTR stabilizer therapy at baseline (**b**). Patients were censored at time of death or last visit prior to Week 140 IQR, interquartile range; TTR, transthyretin.

Of the 1,432 patients who were randomized (Extended Data Fig. 1) and received at least one dose of treatment, 819 (57.2%) were receiving TTR stabilizers at baseline (all tafamidis), including 410 out of 715 (57.3%) in the eplontersen arm and 409 out of 717 (57.0%) in the placebo arm. Demographic and clinical characteristics were generally similar between treatment arms among patients receiving and not receiving stabilizers at baseline (Table 1). The duration between ATTR-CM diagnosis and enrollment was 0.5 (interquartile range (IQR) 0.3–1.1) years in those not on baseline stabilizers and 1.1 (IQR 0.6–2.4) years in those on baseline stabilizers (Extended Data Table 1). The median duration of stabilizer therapy before randomization was 9.0 months (IQR 4.0–18.1, range 0–92.0) (Extended Data Fig. 2).

**Table 1:** Baseline demographic and clinical characteristics, stratified by use of TTR stabilizer at baseline

Characteristic	No baseline stabilizer			Baseline stabilizer			Overall	
	Placebo (*n* = 308)	Eplontersen (*n* = 305)	*P* value	Placebo (*n* = 409)	Eplontersen (*n* = 410)	*P* value	Placebo (*N* = 717)	Eplontersen (*N* = 715)
Age, years								
Mean ± s.d.	77.7 ± 6.2	77.5 ± 6.7	0.77	75.8 ± 7.5	75.2 ± 7.4	0.25	76.6 ± 7.0	76.2 ± 7.2
Median (IQR)	78 (74–82)	79 (73–82)	0.95	77 (71–81)	76 (71–80)	0.16	78.0 (41–90)	77.0 (46–91)
Male sex, *n* (%)	277 (89.9%)	266 (87.2%)	0.29	384 (93.9%)	370 (90.2%)	0.05	661 (92.2%)	636 (89.0%)
Race, *n* (%)			0.97			0.90		
White	288 (93.5%)	287 (94.1%)		349 (85.3%)	347 (84.6%)		637 (88.8%)	634 (88.7%)
Black	14 (4.5%)	12 (3.9%)		43 (10.5%)	42 (10.2%)		57 (7.9%)	54 (7.6%)
Asian	2 (0.6%)	3 (1.0%)		13 (3.2%)	17 (4.1%)		15 (2.1%)	20 (2.8%)
Multiple/other	4 (1.2%)	3 (0.9%)		4 (1.0%)	4 (1.0%)		8 (1.1%)	7 (1.0%)
Hispanic or Latino	22 (7.1%)	33 (10.8%)	0.11	21 (5.1%)	21 (5.1%)	0.99	43 (6.0%)	54 (7.6%)
Region, *n* (%)			0.67			0.44		
North America	15 (4.9%)	16 (5.2%)		253 (61.9%)	270 (65.9%)		268 (37.4%)	286 (40.0%)
Europe	229 (74.4%)	217 (71.1%)		123 (30.1%)	107 (26.1%)		352 (49.1%)	324 (45.3%)
Rest of the world	64 (20.8%)	72 (23.6%)		33 (8.1%)	33 (8.0%)		97 (13.5%)	105 (14.7%)
TTR genotype, *n* (%)								
Wild type	277 (89.9%)	271 (88.9%)	0.66	336 (82.2%)	337 (82.2%)	0.99	613 (85.5%)	608 (85.0%)
Variant	31 (10.1%)	34 (11.1%)		73 (17.8%)	73 (17.8%)		104 (14.5%)	107 (15.0%)
Time since diagnosis of ATTR-CM, years, median (IQR)	0.5 (0.3–1.1)	0.5 (0.3–1.1)	0.78	1.0 (0.5–2.2)	1.2 (0.6–2.6)	0.13	0.8 (0.4–1.8)	0.8 (0.4–1.9)
NYHA functional class, *n* (%)^a^			0.05			0.91		
Class I	47 (15.3%)	27 (8.9%)		61 (14.9%)	57 (13.9%)		108 (15.1%)	84 (11.7%)
Class II	212 (68.8%)	228 (74.8%)		276 (67.5%)	281 (68.5%)		488 (68.1%)	509 (71.2%)
Class III	49 (15.9%)	50 (16.4%)		72 (17.6%)	72 (17.6%)		121 (16.9%)	122 (17.1%)
NAC disease stage, *n* (%)^b^			0.20			0.44		
Stage 1	187 (60.7%)	170 (55.7%)		275 (67.2%)	264 (64.4%)		462 (64.4%)	434 (60.7%)
Stage 2	93 (30.2%)	94 (30.8%)		99 (24.2%)	115 (28.0%)		192 (26.8%)	209 (29.2%)
Stage 3	28 (9.1%)	41 (13.4%)		35 (8.6%)	31 (7.6%)		63 (8.8%)	72 (10.1%)
6-min walk distance, m								
Mean ± s.d.	341.4 ± 94.9	332.4 ± 90.1	0.23	363.5 ± 98.1	363.9 ± 96.7	0.95	354.0 ± 97.3	350.5 ± 95.2
KCCQ-OS score, mean ± s.d.	72.4 ± 19.7	69.8 ± 20.7	0.11	72.8 ± 20.3	74.5 ± 19.0	0.23	72.7 ± 20.0	72.5 ± 19.8
eGFR, ml min^−1^ per 1.73 m^2^, mean ± s.d.	63.6 ± 15.5	60.6 ± 16.1	0.017	61.4 ± 16.8	60.7 ± 16.5	0.56	62.4 ± 16.2	60.7 ± 16.3
NT-proBNP, pg ml^−1^, median (IQR)	2,174 (1,331–3,763)	2,348 (1,381–4,144)	0.25	1,780 (1,237–3,000)	1,937 (1,297–3,017)	0.39	1,965 (1,284–3,422)	2,068 (1,323–3,563)
LV wall thickness, mm, mean ± s.d.	18.6 ± 2.7	18.4 ± 2.9	0.36	18.1 ± 2.8	18.4 ± 2.9	0.15	18.3 ± 2.8	18.4 ± 2.9
LV ejection fraction, %, mean ± s.d.	56.2 ± 9.1	56.6 ± 9.5	0.62	55.6 ± 9.9	55.4 ± 10.4	0.71	55.9 ± 9.6	55.9 ± 10.0
Baseline TTR stabilizer use, *n* (%)	0	0		409 (100.0%)	410 (100.0%)		409 (57.0%)	410 (57.3%)
Duration of stabilizer use before randomization, months, median (IQR)	NA	NA		8.4 (3.7–16.6)	9.8 (4.2–19.0)	0.046	8.4 (3.7–16.6)	9.6 (4.1–19.0)
Drop-in stabilizer use after randomization through week 160, *n* (%)^c^	174 (56.5%)	174 (57.0%)		NA	NA		174 (24.3%)	174 (24.3%)
Tafamidis	174 (56.5%)	173 (56.7%)		NA	NA		174 (24.3%)	173 (24.2%)
Acoramidis	1 (0.3%)	2 (0.7%)		NA	NA		1 (0.1%)	2 (0.3%)
Other concomitant medications, *n* (%)^d^								
Anticoagulants	187 (60.7%)	177 (58.0%)	0.50	247 (60.4%)	264 (64.4%)	0.24	434 (60.5%)	441 (61.7%)
Antiplatelets	60 (19.5%)	68 (22.3%)	0.39	95 (23.2%)	107 (26.1%)	0.34	155 (21.6%)	175 (24.5%)
Diuretic	281 (91.2%)	280 (91.8%)	0.80	364 (89.0%)	361 (88.0%)	0.67	645 (90.0%)	641 (89.7%)
Loop diuretic	276 (89.6%)	270 (88.5%)	0.67	346 (84.6%)	351 (85.6%)	0.68	622 (86.8%)	621 (86.9%)
Beta-blocker	146 (47.4%)	144 (47.2%)	0.96	157 (38.4%)	167 (40.7%)	0.49	303 (42.3%)	311 (43.5%)
ACEi	70 (22.7%)	67 (22.0%)	0.82	47 (11.5%)	58 (14.1%)	0.26	117 (16.3%)	125 (17.5%)
ARB	71 (23.1%)	76 (24.9%)	0.59	106 (25.9%)	97 (23.7%)	0.45	177 (24.7%)	173 (24.2%)
ARNi	17 (5.5 %)	22 (7.2%)	0.39	23 (5.6%)	23 (5.6%)	0.99	40 (5.6%)	45 (6.3%)
MRA	120 (39.0%)	134 (43.9%)	0.21	195 (47.7%)	169 (41.2%)	0.06	315 (43.9%)	303 (42.4%)
SGLT2i	63 (20.5%)	64 (21.0%)	0.87	67 (16.4%)	71 (17.3%)	0.72	130 (18.1%)	135 (18.9%)

Percentages may not total 100 owing to rounding. Multiplicity correction was not performed. Baseline characteristics were summarized using descriptive statistics stratified by baseline stabilizer status. Continuous variables were reported as means with standard deviations or medians with IQRs and ranges, as appropriate; categorical variables were summarized as counts and percentages. For between-group comparisons, two-sided *P* values of ≤ 0.05 were considered to indicate statistical significance.

ACEi, angiotensin-converting enzyme inhibitor; ARB, angiotensin II receptor blocker; ARNi, angiotensin receptor-neprilysin inhibitor; LV, left ventricular; MRA, mineralocorticoid receptor antagonist; NA, not applicable; NAC, National Amyloidosis Centre; SGLT2i, sodium-glucose transport 2 inhibitor.

^a^NYHA functional classes were defined as follows: class I, no limitation of physical activity; class II, slight limitation of physical activity, with ordinary activity causing symptoms; class III, marked physical limitations, with less-than-ordinary activity causing symptoms; and class IV, symptoms at rest or with any activity.

^b^NAC stages were defined as follows: stage 1, NT-proBNP ≤ 3,000 pg ml^−1^ and estimated glomerular filtration rate (eGFR) ≥ 45 ml min^−1^ per 1.73 m^2^; stage 2, NT-proBNP > 3,000 pg ml^−1^ or eGFR < 45 ml min^−1^ per 1.73 m^2^; and stage 3, NT-proBNP > 3,000 pg ml^−1^ and eGFR < 45 ml min^−1^ per 1.73 m^2^.

^c^All three patients who had drop-in acoramidis were not on stabilizers at baseline. Two of the three patients with drop-in acoramidis had initiated tafamidis before acoramidis. The patient who had acoramidis but not tafamidis was in the eplontersen arm.

^d^Concomitant medications were defined as medication use starting on or before the day of randomization and concluding on or after the day of randomization.

Compared with patients not receiving stabilizers at baseline, those receiving stabilizers at baseline were younger; were more likely to be male, Black, non-Hispanic and located in North America; had a longer disease duration; and were less likely to have wild-type ATTR-CM (Extended Data Table 1). Those on stabilizers at baseline also demonstrated greater 6-min walk test (6MWT) distance, higher Kansas City Cardiomyopathy Questionnaire-Overall Summary (KCCQ-OS) score and less severe disease, reflected by the lower proportion of patients at higher National Amyloidosis Centre stages and a lower median N-terminal pro-B-type natriuretic peptide (NT-proBNP) level, and more often had a permanent pacemaker (Extended Data Table 1). Of the patients who were receiving stabilizers at baseline, 25 out of 410 (6.1%) and 26 out of 409 (6.4%) stopped stabilizer therapy during the trial in the eplontersen and placebo arms, respectively. Of the patients who were not receiving stabilizers at baseline, 174 out of 305 (57.0%) and 174 out of 308 (56.5%) started stabilizer therapy during the trial in the eplontersen and placebo arms, respectively.

### Primary and secondary endpoints—interaction with baseline stabilizer use

In the overall study, treatment with eplontersen did not significantly reduce the primary composite endpoint (rate ratio (RR) = 0.89, 95% confidence interval (CI) 0.73 to 1.09, *P* = 0.277). The use of stabilizers at baseline was a treatment effect modifier with interactions directionally favoring greater clinical benefit for eplontersen in participants not on baseline stabilizers for the following endpoints: primary endpoint (*P*_interaction_ = 0.017), change in KCCQ-OS score from baseline at week 140 (*P*_interaction_ = 0.002) and recurrent CV events up to week 140 (*P*_interaction_ = 0.009), but not for change in 6MWT at week 140 (*P*_interaction_ = 0.085) and all-cause (*P*_interaction_ = 0.44) or CV mortality (*P*_interaction_ = 0.74) up to week 160 (Table 2). There were also beneficial interactions favoring eplontersen in participants not receiving baseline stabilizers for mean change from baseline at week 140 in NT-proBNP (*P*_interaction_ = 0.003) (Table 2).

**Table 2:** Efficacy results for prespecified endpoints stratified by use of TTR stabilizer at baseline

Prespecified endpoints	No stabilizer (monotherapy) subgroup		Stabilizer subgroup		*P* value for interaction
	*N* = 613		*N* = 819		
	Efficacy versus placebo (95% CI)	*P* value	Efficacy versus placebo (95% CI)	*P* value	
Primary (CV mortality and recurrent CV events up to week 140)	RR = 0.71 (0.54 to 0.93)	0.012	RR = 1.14 (0.85 to 1.53)	0.39	0.017
6-min walk difference from baseline at week 140	LSM = 29.90 (14.3 to 45.5)	<0.001	LSM = 12.00 (−1.0 to 25.0)	0.07	0.085
KCCQ-OS difference from baseline at week 140	LSM = 7.6 (4.2 to 11.0)	<0.001	LSM = 1.1 (−1.3 to 3.6)	0.37	0.002
Recurrent CV events up to week 140	RR = 0.67 (0.50 to 0.90)	0.008	RR = 1.16 (0.85 to 1.58)	0.35	0.009
All-cause mortality up to week 160	HR = 0.85 (0.62 to 1.16)	0.30	HR = 0.70 (0.49 to 1.01)	0.06	0.44
CV mortality up to week 160	HR = 0.90 (0.61 to 1.32)	0.59	HR = 0.83 (0.55 to 1.25)	0.37	0.74
Placebo-corrected geometric mean change in NT-proBNP from baseline at week 140	−26.3% (−34.7 to −16.9)	<0.001	−7.6% (−15.4 to 0.9)	0.08	0.003

Treatment effect of the primary endpoint (composite of CV mortality and recurrent CV clinical events) was estimated using the LWYY method. Changes from baseline at week 140 for the continuous 6MWT and KCCQ-OS endpoints were analyzed in the full analysis set using an analysis of covariance (ANCOVA) model. For between-group comparisons, two-sided *P* values of ≤ 0.05 were considered to indicate statistical significance.

LSM, least-squares mean.

### Clinical endpoints by subgroup of TTR stabilizer use at baseline

#### Eplontersen in patients without stabilizer use at baseline

**Fig. 1:**
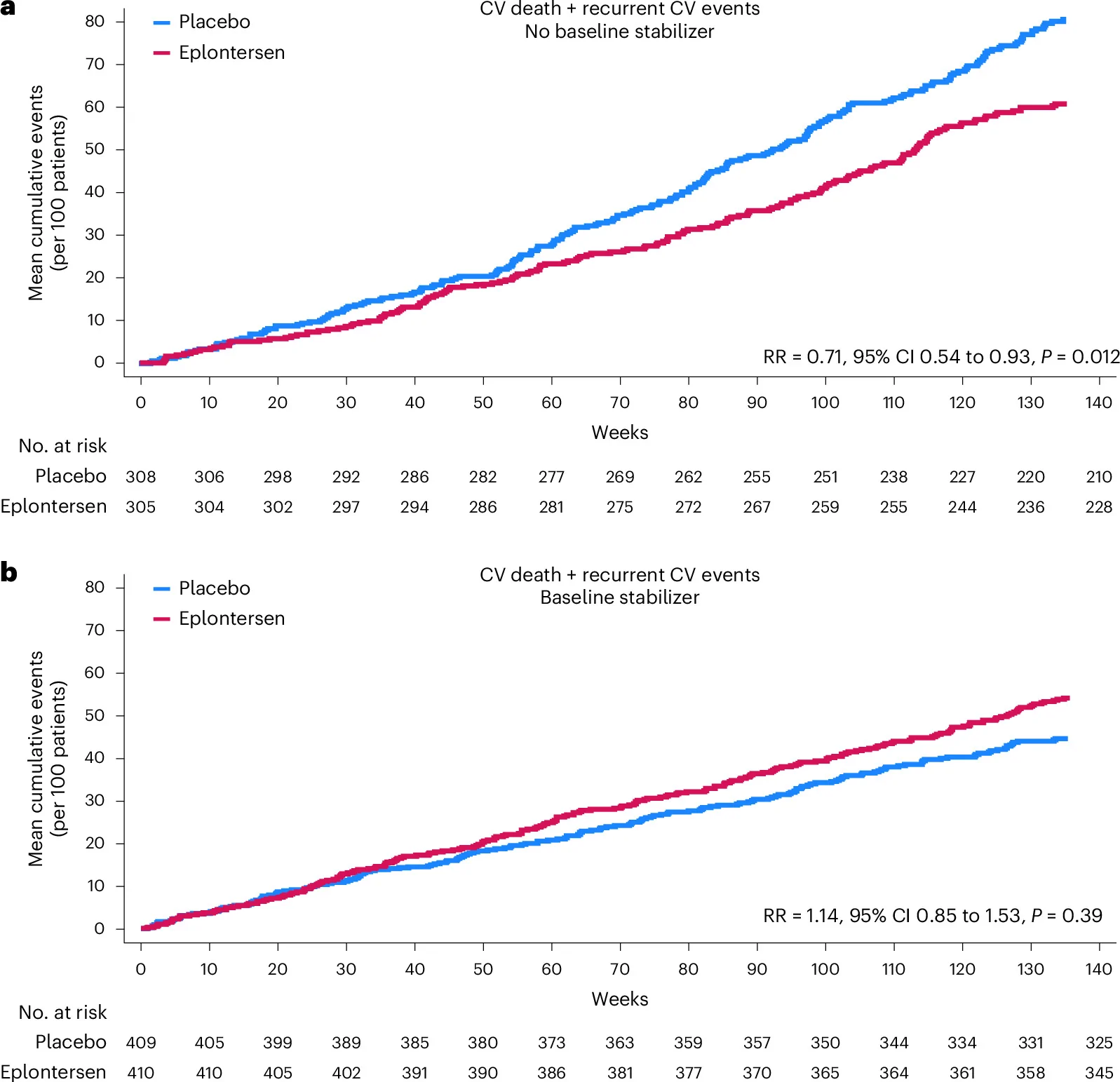
Primary endpoint events. **a**,**b**, The mean cumulative event rate (per 100 patients) for primary endpoint events (CV deaths and recurrent CV events) up to week 140 in patients without stabilizer use (**a**; *P* = 0.012) and with stabilizer use (**b**; *P* = 0.39)^16^ at baseline. RRs and 95% CIs for primary endpoint events and CV events were calculated with the use of the LWYY model. For between-group comparisons, two-sided *P* values of ≤ 0.05 were considered to indicate statistical significance.

**Fig. 2:**
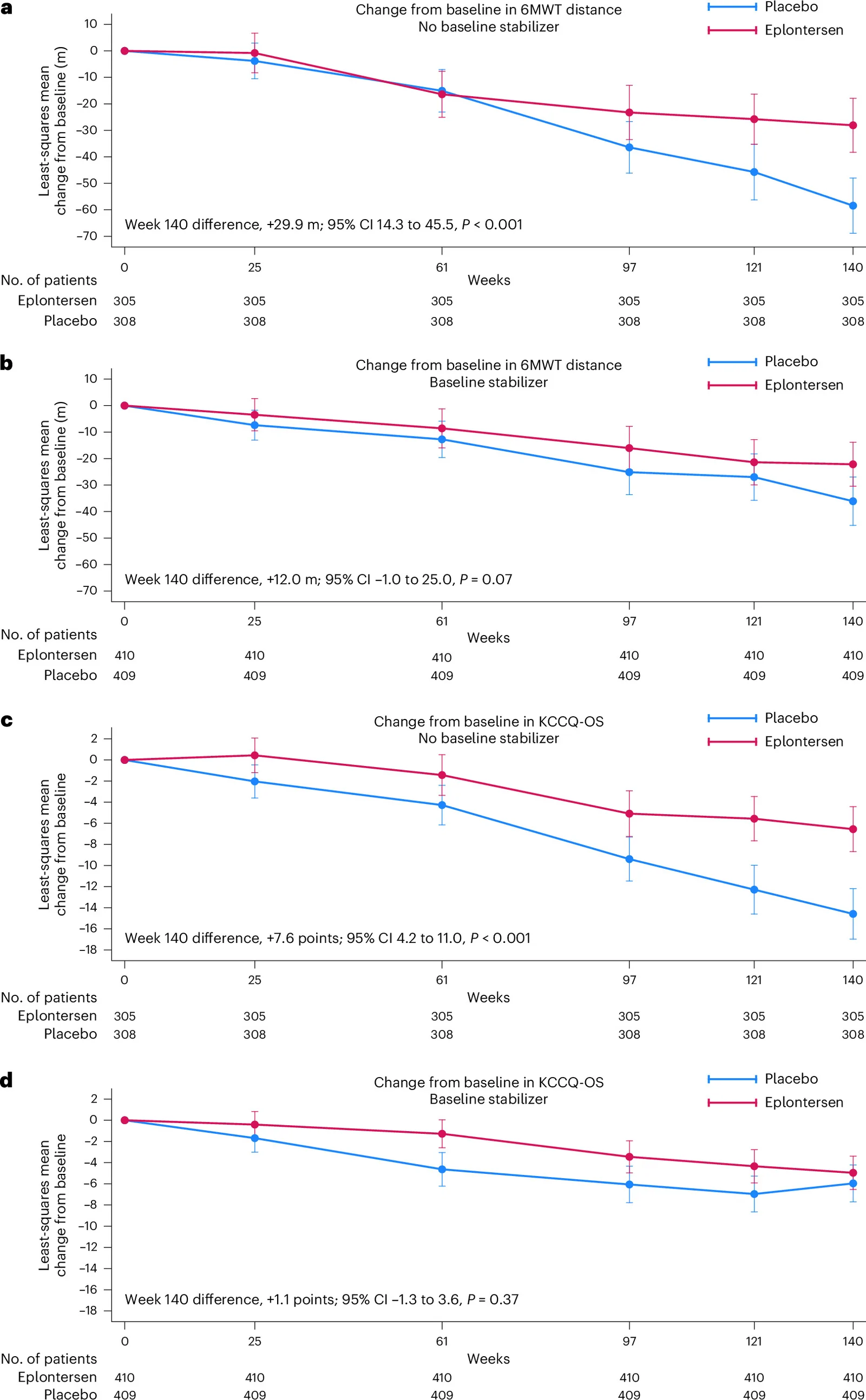
Change from baseline in functional and quality of life assessments for patients without and with tafamidis therapy at baseline. **a**,**b**, The least-squares mean change from baseline in distance (meters) in the 6MWT in patients without (**a**; *P* < 0.001) and with (**b**; *P* = 0.07) stabilizer use at baseline. **c**,**d**, The least-squares mean change from baseline scores in KCCQ-OS in patients without (**c**; *P* < 0.001) and with (**d**; *P* = 0.37) stabilizer use at baseline. Error bars indicate 95% CIs. Changes from baseline at week 140 and 95% CIs for the continuous 6MWT and KCCQ-OS endpoints were calculated using an analysis of covariance (ANCOVA) model. For between-group comparisons, two-sided *P* values of ≤ 0.05 were considered to indicate statistical significance.

**Fig. 3:**
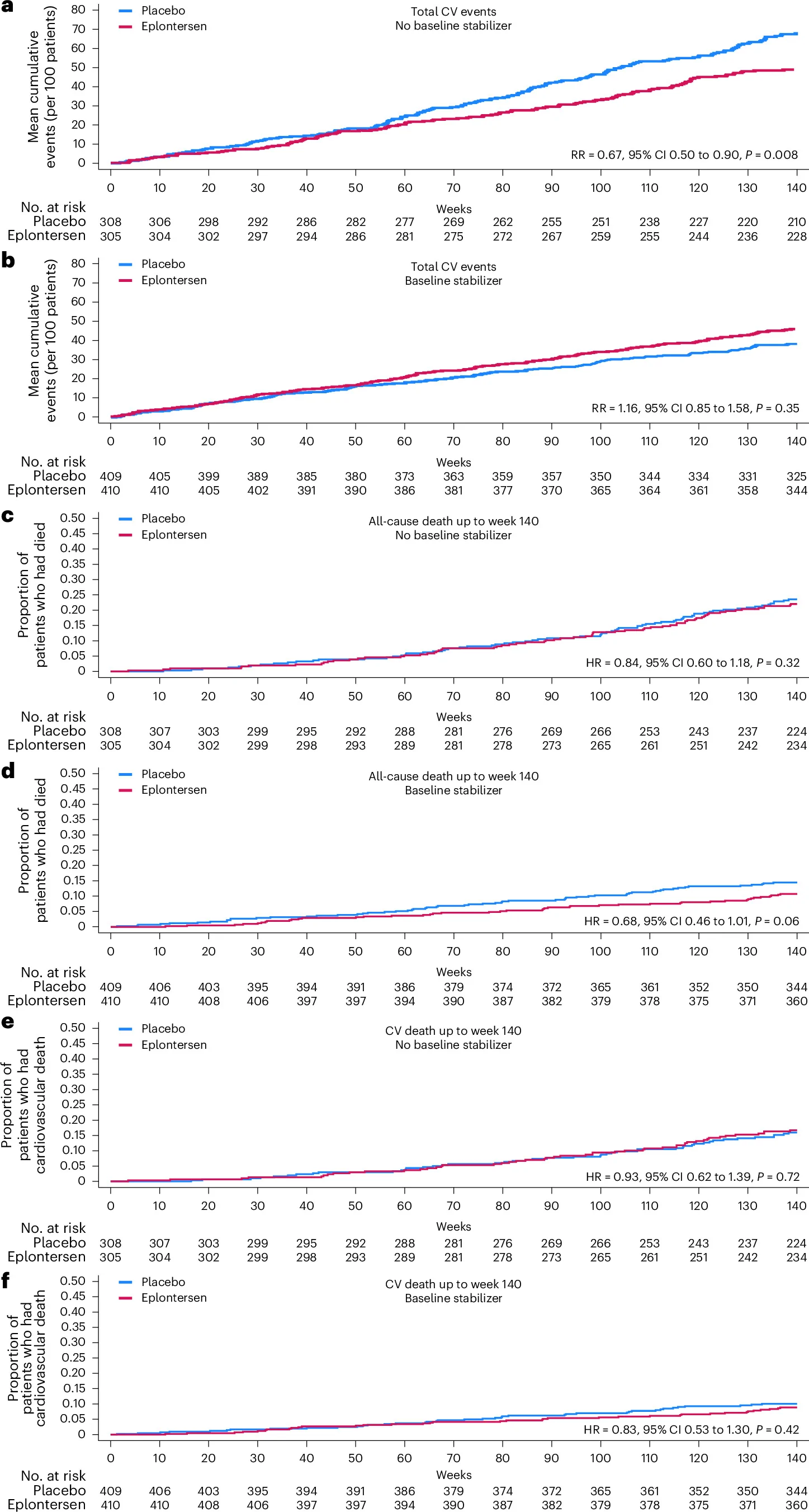
CV events, all-cause death and CV death. **a**,**b**, The mean cumulative event rate (per 100 patients) for recurrent CV events up to week 140 in patients without (**a**) and with (**b**) stabilizer use at baseline. **c**,**d**, The risk of all-cause death up to week 140 in patients without (**c**) and with (**d**) stabilizer use at baseline. **e**,**f**, The risk of CV death up to week 140 in patients without (**e**) and with (**f**) stabilizer use at baseline. HRs and 95% CIs were calculated with the use of the LWYY model. For between-group comparisons, two-sided *P* values of ≤ 0.05 were considered to indicate nominal statistical significance.

**Extended Data Fig. 3:**
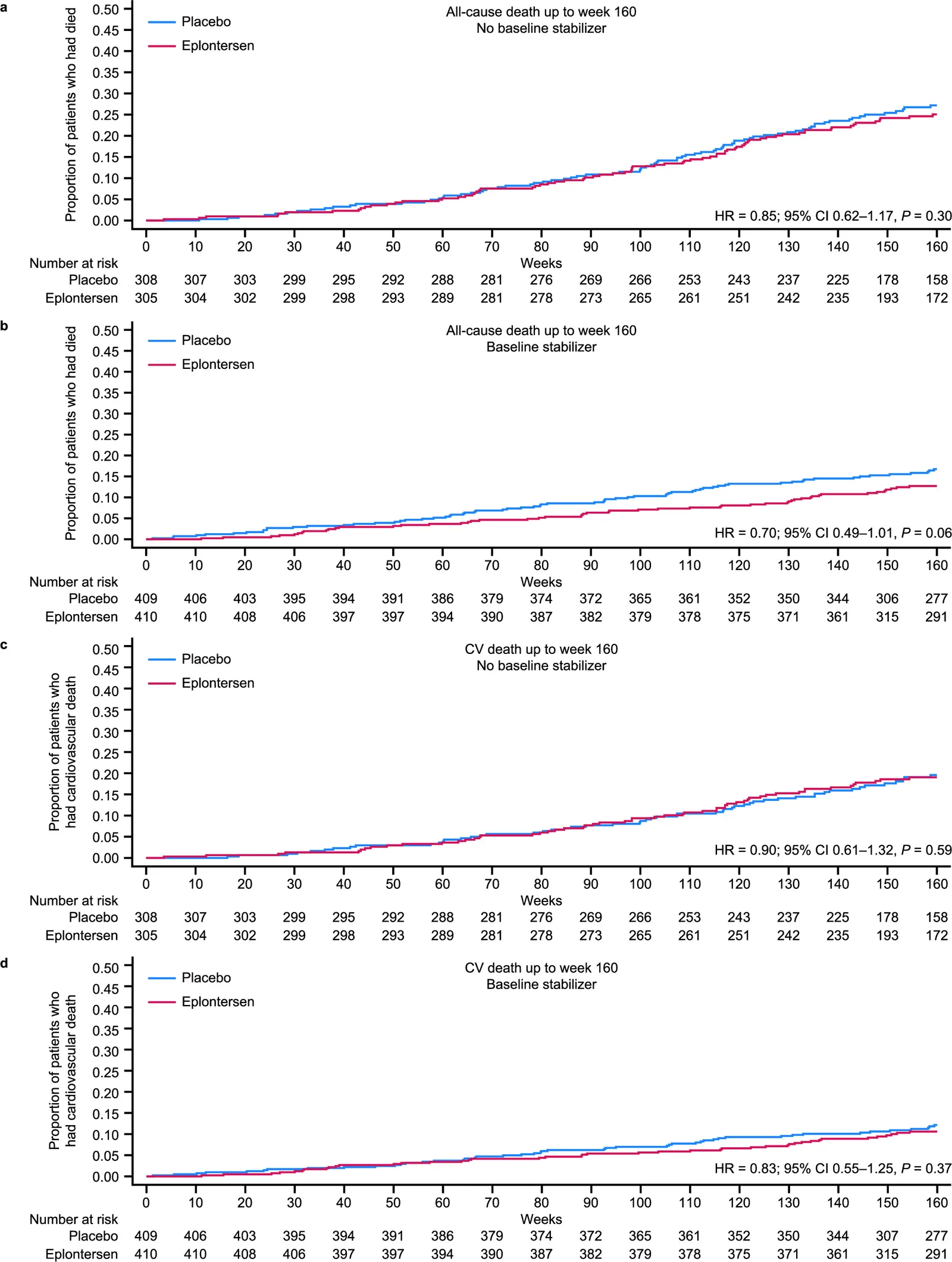
All-cause mortality and CV mortality in patients without and with stabilizer use at baseline up to week 160. Panels **a** and **b** show the risk of all-cause mortality over time up to week 160 in patients without (*P* = 0.30) and with stabilizer at baseline (*P* = 0.05), respectively. Panels **c** and **d** show the risk of CV mortality over time up to week 160 in patients without (*P* = 0.59) and with stabilizer at baseline (*P* = 0.37), respectively. HRs and 95% CIs were calculated with the use of the LWYY model. For between-group comparisons, two-sided P values of ≤ 0.05 were considered to indicate nominal statistical significance CI, confidence interval; CV, cardiovascular; HR, hazard ratio.

**Extended Data Fig. 4:**
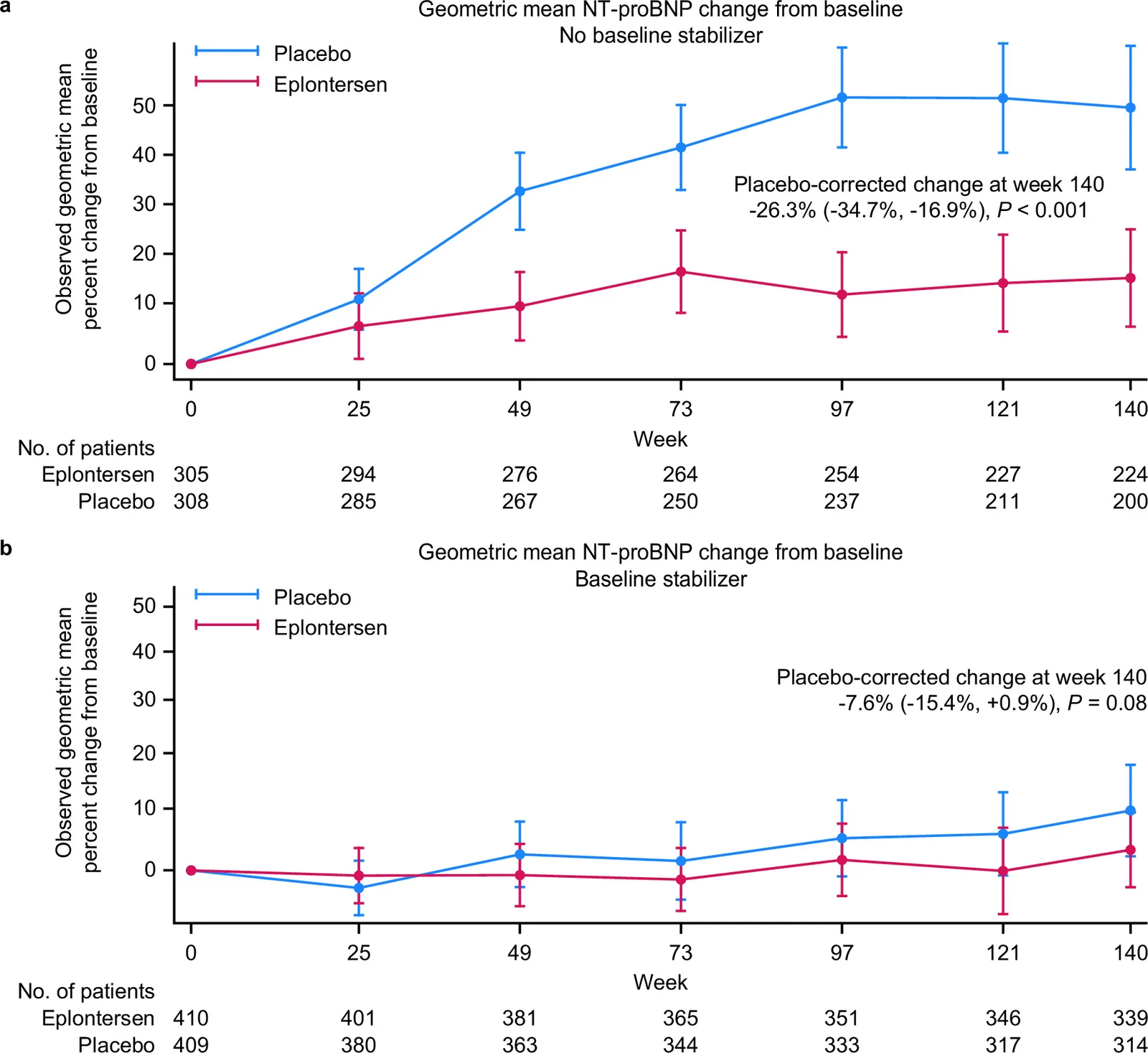
Change from baseline in NT-proBNP in patients without and with stabilizer use at baseline. **a**,**b**, Change in NT-proBNP from baseline at week 140 in patients without (**a**) and with (**b**) stabilizer use at baseline. Error bars indicate 95% confidence intervals. *P* < 0.001 for Change from baseline in NT-proBNP in patients without baseline stabilizer. *P* = 0.08 for Change from baseline in NT-proBNP in patients with baseline stabilizer. NT-proBNP, N-terminal prohormone of brain natriuretic peptide.

In the subgroup of patients not receiving stabilizers at baseline (*n* = 613), initiation of stabilizers after randomization through week 160 was similar in those assigned to eplontersen and placebo, occurring in 174 (57.0%) and 174 (56.5%) patients, respectively (Table 1). There were 172 primary endpoint events among those treated with eplontersen (*n* = 305) versus 221 in those treated with placebo (*n* = 308) (RR = 0.71, 95% CI 0.54 to 0.93, *P* = 0.012) (Fig. 1a). Eplontersen reduced the decline in 6MWT distance (least-squares mean difference (LSMD) = 29.9 m, 95% CI 14.3 to 45.5, *P* < 0.001) and KCCQ-OS score (LSMD = 7.6 points, 95% CI 4.2 to 11.0, *P* < 0.001) at week 140 compared with placebo (Fig. 2a,c). Among the components of the primary endpoint, treatment with eplontersen reduced CV events (RR = 0.67, 95% CI 0.50 to 0.90, *P* = 0.008) (Fig. 3a) but did not reduce CV mortality up to week 140 (hazard ratio (HR) = 0.93, 95% CI 0.62 to 1.39, *P* = 0.72) (Fig. 3e). Eplontersen did not reduce all-cause mortality up to week 140 (HR = 0.84, 95% CI 0.60 to 1.18, *P* = 0.32) (Fig. 3c). CV and all-cause mortality analyses up to week 160 are shown in Extended Data Fig. 3 and did not show differences among patients receiving eplontersen and those receiving placebo. Lastly, eplontersen treatment mitigated the increase in serum NT-proBNP levels compared with placebo, with differences observed from 49 weeks onward (Extended Data Fig. 4a).

#### Eplontersen in patients with stabilizer use at baseline

In patients receiving stabilizers at baseline (*n* = 819), those receiving eplontersen (*n* = 410) experienced 209 primary events compared with 171 events in those receiving placebo (*n* = 409) (RR = 1.14, 95% CI 0.85 to 1.53, *P* = 0.39) (Fig. 1b). Eplontersen treatment did not prevent decline in 6MWT distance (LSMD = 12.0 m, 95% CI −1.0 to 25.0, *P* = 0.07) or KCCQ-OS score (LSMD = 1.1 points, 95% CI −1.3 to 3.6, *P* = 0.37) (Fig. 2b,d). In the subgroup with TTR stabilizer at baseline, treatment with eplontersen did not reduce the components of the primary endpoint with regard to CV events up to week 140 (RR = 1.16, 95% CI 0.85 to 1.58, *P* = 0.35) (Fig. 3b) or CV mortality (HR = 0.83, 95% CI 0.53 to 1.30, *P* = 0.42) (Fig. 3f). HR for all-cause mortality up to week 140 was 0.68 (95% CI 0.46 to 1.01, *P* = 0.06) among those receiving eplontersen treatment with a stabilizer at baseline (Fig. 3d). CV and all-cause mortality analyses up to week 160 are shown in Extended Data Fig. 3 and showed similar results among patients receiving eplontersen and those receiving placebo. Lastly, among those receiving TTR stabilizers at baseline, NT-proBNP levels were similar in patients receiving eplontersen and placebo through 140 weeks (Extended Data Fig. 4b).

### Reduction in serum TTR according to baseline stabilizer use

At the start of the trial, the median absolute serum TTR concentration in participants randomized to eplontersen was 22.0 mg dl^−1^ in the subgroup of participants without stabilizer at baseline and 30.7 mg dl^−1^ in the subgroup of patients with stabilizer at baseline. At the end of the trial, the median absolute serum TTR concentration in those randomized to eplontersen was similar in both subgroups: 6.5 mg dl^−1^ in the subgroup of participants without stabilizer at baseline and 7.1 mg dl^−1^ in the subgroup of patients with stabilizer at baseline.

Using an on-treatment analysis, at the end of the trial, the median absolute serum TTR concentration in those randomized to eplontersen was 6.3 mg dl^−1^ in the subgroup of patients without stabilizer at baseline and 6.9 mg dl^−1^ in the subgroup of patients with stabilizer at baseline.

### Safety

Eplontersen was safe and well tolerated compared with placebo in both subgroups of patients receiving and not receiving baseline stabilizers, with similar rates of serious treatment-emergent adverse events and adverse events relating to study drug discontinuation (Extended Data Table 2).

## Discussion

In this prespecified analysis of the CARDIO-TTRansform trial, we observed treatment effect modification by baseline TTR stabilizer use, such that patients receiving eplontersen as a monotherapy had clinically meaningful and nominally significant benefits that were consistent across multiple endpoints, including reductions in the primary endpoint of CV mortality and recurrent CV events, less decline in functional capacity and quality of life, and smaller increases in NT-proBNP, despite high rates of stabilizer drop-in use in this subgroup. By contrast, no additional treatment benefit was observed with eplontersen in patients receiving stabilizers at baseline despite consistent reduction in TTR.

Patients with ATTR-CM can progress despite TTR stabilizer therapy^10^, and aggressive reduction of amyloidogenic TTR substrate may mitigate ‘amyloid seeding’, whereby existing amyloid fibrils can enucleate and accelerate subsequent fibril formation from circulating TTR^17,18^. Accordingly, the CARDIO-TTRansform trial tested the hypothesis that adding silencer therapy—either as first line or as add-on to stabilizers—would improve clinical outcomes in patients with ATTR-CM. The CARDIO-TTRansform trial enrolled the largest and most clinically diverse cohort of patients with ATTR-CM to date, providing an opportunity to evaluate the effectiveness of TTR gene silencing therapy according to concomitant TTR stabilizer therapy use across the ATTR-CM disease spectrum. The use of stabilizers, which became widely available during this study, was not restricted in the CARDIO-TTRansform trial in order to evaluate TTR gene silencing in a contemporary representative patient population, given that tafamidis was rapidly becoming the standard of care. This is different from the HELIOS-B study^14^ in which baseline stabilizer use was capped at 40% and patients not receiving a stabilizer at baseline were excluded if there was an active plan to initiate stabilizer therapy within the first 12 months after randomization. In HELIOS-B, TTR stabilizer therapy was initiated in 22% and 21% of the patients not taking stabilizers at baseline in the vutrisiran and placebo groups, respectively, which was considerably less than in the current study^14^. In CARDIO-TTRansform, stabilizer use at baseline was a treatment effect modifier, with notable heterogeneity in the composite primary endpoint that was nominally significant (RR = 0.71 with no stabilizer use, RR = 1.14 with stabilizer use; *P*_interaction_ = 0.017). Among patients receiving stabilizer therapy at baseline, there were fewer events of all-cause death (HR = 0.68) and CV death (HR = 0.83) but a higher number of CV events (RR = 1.16) in the eplontersen arm. This may be attributable to patients with advanced cardiac involvement living longer but with an increased susceptibility to recurrent CV events. This is similar to what was observed in the ATTR-ACT trial with tafamidis, in which patients with New York Heart Association (NYHA) class III heart failure lived longer but had an increased risk of CV hospitalizations^10^. The results of CARDIO-TTRansform allow comparison of outcomes in patients receiving eplontersen or placebo with or without baseline stabilizer therapy, guiding future therapeutic decisions in the field and addressing the potential value of combining medications that act at different points of the same biological pathway.

The results of this analysis in a prespecified subgroup as part of the formal testing show that the treatment effect of eplontersen was strongly modified by the presence of a TTR stabilizer at baseline, with significant interaction *P* values for the primary composite endpoint and several key secondary endpoints. In addition to the primary endpoint, an interaction effect with baseline stabilizer use was also found for health-related quality of life, reflected by change in KCCQ-OS scores. Despite TTR stabilizer and TTR gene silencing therapies having different specific mechanisms of action, these therapies act at different places within the same biological pathway in the amyloidogenic cascade by limiting the available precursor protein for ongoing TTR amyloid formation, likely accounting for the absence of additional benefit of eplontersen on top of background stabilizer therapy. Notably, the absolute serum TTR concentration in those randomized to eplontersen at week 140 did not differ between those on stabilizer and not on stabilizer at baseline, suggesting no difference in the pharmacologic effect of eplontersen based on background stabilizer use. Thus, differences in TTR reduction are unlikely to account for differences in the treatment effect of eplontersen between the stabilizer and non-stabilizer subgroups^14^. Although no additional benefit was observed with eplontersen for the primary outcome in patients on baseline TTR stabilizers, we observed apparent clinically meaningful benefits in patients not on stabilizers at baseline.

Notably, TTR stabilizer drop-in use during the trial was common in patients who did not receive stabilizer at baseline, with approximately 57% starting stabilizer therapy (predominantly tafamidis) in both the eplontersen and placebo groups. The similar rates of stabilizer drop-in between treatment groups suggest that access to commercially available stabilizers, rather than clinical factors such as disease progression or deterioration, was the primary driver of treatment initiation during the trial.

Different treatment effects according to baseline stabilizer use seem to explain the contrasting overall results of CARDIO-TTRansform and HELIOS-B^14^. Although there was no upper NT-proBNP limit in CARDIO-TTRansform, there were otherwise only modest differences in baseline characteristics between the two studies, except for the use of stabilizers and sodium-glucose transport 2 inhibitors^19^; in addition, placebo-adjusted TTR knockdown seemed comparable, but direct comparisons are limited by assay, sampling timepoint and population differences between the trials, as well as the differential rates of TTR stabilizer use, which causes TTR levels to increase. For participants not receiving stabilizers at baseline, both studies observed benefits with TTR gene silencing on the composite primary endpoint, functional capacity and quality of life, despite greater stabilizer drop-in use in the eplontersen trial. By contrast, for participants receiving a stabilizer at baseline, CARDIO-TTRansform showed no clear benefit of gene silencing, whereas HELIOS-B showed a non-significant trend favoring vutrisiran, albeit with wide confidence intervals^20^. In HELIOS-B^20^, the RR (vutrisiran versus placebo) for the composite endpoint of CV mortality and recurrent CV events in the baseline stabilizer subgroup was 0.78 (95% CI 0.50 to 1.22, *P* = 0.28, *n* = 259)^20^. Although the point estimate favored vutrisiran, the wide confidence intervals resulted in uncertainty regarding the treatment effect. The additional data from CARDIO-TTRansform, with a much larger sample size (*n* = 819), suggest that the incremental benefit of gene silencing with eplontersen in patients receiving background stabilizer therapy is not evident.

The study had some potential limitations. First, estimation of a true monotherapy effect of eplontersen is limited by the drop-in use of stabilizers during the trial. However, this substantial drop-in use of stabilizers in patients who were not receiving them at baseline would be expected to drive the interaction toward the null and underestimate the true monotherapy effect of eplontersen. Second, the analyses of combination therapy address only the sequence of adding a gene silencer to a background stabilizer, and not vice versa. Our analyses were conducted on subpopulations of the main trial, in which chance effects and residual confounding may occur owing to smaller sample sizes. However, the differential effects of eplontersen according to baseline stabilizer use were consistent across multiple endpoints, making this unlikely. Lastly, tafamidis was almost the only TTR stabilizer used in this study. As such, assessment of the effects of other stabilizers such as acoramidis was not possible.

In conclusion, our findings demonstrate that treatment effects of eplontersen were modified by the use of stabilizers at baseline. Eplontersen seemed to produce clinically meaningful benefits as a monotherapy but not when added to a TTR stabilizer.

## Methods

### Inclusion criteria

- Provided written informed consent and able to comply with all study requirements
- Aged 18 to 90 years (inclusive) at screening
- Women must have been non-pregnant and non-lactating and at least one of the following:
  1. Surgically sterile (for example, have undergone tubal occlusion, hysterectomy, bilateral salpingectomy or bilateral oophorectomy)
  2. Post-menopausal (defined as having 12 months of spontaneous amenorrhea in women 55 years of age or older, or having 12 months of spontaneous amenorrhea without an alternative medical cause and follicle-stimulating hormone levels in the post-menopausal range for those ≤55 years of age)
  3. Abstinent
  4. Agree to use one highly effective contraceptive method from the time of signing the informed consent form until ≥24 weeks after the last dose of study intervention, if engaged in sexual relations of childbearing potential

- Men must have been at least one of the following:
  1. Surgically sterile
  2. Abstinent
  3. Agree to use an acceptable contraceptive method from the time of signing the informed consent form until ≥24 weeks after the last dose of study intervention, if engaged in sexual relations with a woman of childbearing potential

- Were willing to be genetically tested for mutations in the TTR gene during screening
- Had amyloid deposits in cardiac or non-cardiac tissue confirmed by Congo Red (or equivalent) staining or technetium scintigraphy (^99m^Tc-3,3-diphosphono-1,2-propanodicarboxylic acid, ^99m^Tc-pyrophosphate or ^99m^Tc-hydroxymethylene-diphosphonate) with centrally confirmed grade 2 or 3 cardiac uptake in the absence of abnormal light chain ratio
- Had an end-diastolic interventricular septum thickness of >12 mm at screening (evaluated by echocardiogram)
- Had a medical history of heart failure secondary to hereditary or wild-type ATTR-CM with either previous hospitalization for heart failure, which could include either hospitalization for arrhythmia or pacemaker/implantable cardioverter defibrillator placement, or symptoms and signs of volume overload or elevated intracardiac pressure that required treatment with diuretics other than mineralocorticoid receptor antagonists
- NT-proBNP levels of ≥600 pg ml^−1^ at screening (≥1,200 pg ml^−1^ for patients with atrial fibrillation)
- Were classified as NYHA class I to III
- Had a 6MWT distance of ≥100 m at screening
- If treated for heart failure, stayed on a stable dosage regimen for 2 weeks before randomization
- Were willing to adhere to a vitamin A supplementation regimen

### Exclusion criteria

- A history of acute coronary syndrome, unstable angina, stroke, transient ischemic attack, coronary revascularization, cardiac device implantation, cardiac valve repair or major surgery within 3 months of screening.
- Had a hospitalization or urgent emergency visit for worsening of heart failure with discharge date within 4 weeks before or during screening.
- Had uncontrolled hypertension (defined as systolic blood pressure (BP) > 160 mmHg or diastolic BP > 100 mmHg).
- Had an uncontrolled clinically relevant cardiac arrhythmia or uncorrected cardiac valvular disease, per investigator’s assessment.
- Had cardiomyopathy not primarily caused by ATTR-CM.
- Had laboratory results that were out of the bounds of the following or any other clinically relevant abnormalities in screening laboratory values that would render a patient unsuitable for inclusion:
  1. Alanine aminotransferase/aspartate aminotransferase > two times the upper limit of normal (ULN).
  2. Total bilirubin ≥ 2 times the ULN (patients with total bilirubin ≥ two times ULN may have been allowed if only indirect bilirubin was elevated, alanine aminotransferase/aspartate aminotransferase was not greater than the ULN and there was a history of Gilbert’s disease, or if in the opinion of the investigator the bilirubin abnormality was not clinically notable).
  3. Platelets < 125 × 10^9^ l^−1^.
  4. Urine protein creatinine ratio ≥ 750 mg g^−1^, with possible confirmation by random spot testing.
  5. Positive test for blood (including trace) on urinalysis that was subsequently confirmed via urine microscopy showing >five red blood cells per high power field and was related to glomerulopathies.
  6. eGFR < 30 ml min^−1^ per 1.73 m^2^ (<45 ml min^−1^ per 1.73 m^2^ in France, Spain and Denmark).
  7. Abnormal thyroid function tests with clinical significance, per investigator judgment.
  8. Serum retinol level below the lower limit of normal (excepting those with variant ATTR-CM due to mutation at position 84).
  9. Hemoglobin A1c > 9.5%.

- Had monoclonal gammopathy of undetermined significance and/or alterations in immunoglobulin free light chain ratio. For patients with chronic kidney disease and without the presence of monoclonal protein in blood and urine, the acceptable ratio was 0.26–2.25.
- Had an active infection requiring systemic antiviral or antimicrobial therapy that was not completed before study day 1.
- History of or positive test for HIV, hepatitis C or hepatitis B.
- History of bleeding, diathesis or coagulopathy (for example, liver cirrhosis, hematologic malignancy, antiphospholipid antibody syndrome, congenital disorders such as hemophilia A and B, and von Willebrand disease).
- If receiving oral anticoagulants (except vitamin K antagonists), the dose must have been stable for 4 weeks before the first dose of study intervention and has been regularly monitored.
- Had malignancy within 5 years, except for basal or squamous cell carcinoma of the skin, melanoma in situ, prostate carcinoma grade 1, breast ductal carcinoma in situ or carcinoma in situ of the cervix. Patients with a history of other malignancies were eligible per investigator judgment.
- Had a previous liver or heart transplant, and/or left ventricular assist device or anticipated liver transplant or left ventricular assist device within 1 year after randomization.
- Had a Karnofsky performance status of ≤50%.
- Were contraindicated owing to immunosuppressive therapy, per investigator discretion.
- Had known light chain/primary amyloidosis, leptomeningeal amyloidosis or history of multiple myeloma.
- Received treatment with another investigational drug and/or biological agent within 1 month of screening, or 5 half-lives of investigational agent, whichever was longer.
- Current or previous treatment with inotersen, patisiran or other oligonucleotide or RNA therapeutic (including short interfering RNA (siRNA); did not apply to COVID-19 mRNA vaccinations).
- Current treatment with diflunisal, doxycycline with or without ursodeoxycholic acid, and/or non-dihydropyridine calcium-channel blocker (for example, verapamil and diltiazem) without a wash-out period of 14 days before randomization.
- Unwillingness or inability to comply with study procedures, including follow-up, as specified by this protocol, or unwillingness to cooperate fully with the investigator.
- Other physical, social or psychological conditions including illicit drug or alcohol use, which, in the opinion of the investigator, would have made the patient unsuitable for inclusion or could have interfered with the patient participating in or completing the trial.

### Trial design and oversight

CARDIO-TTRansform was a randomized, double-blind, placebo-controlled, multicenter clinical trial conducted in patients with ATTR-CM. Patients were randomized in a 1:1 ratio to receive eplontersen or matching placebo in addition to guideline-directed medical therapy, which could include use of any approved TTR stabilizer at baseline or subsequent initiation (‘drop-in’) during the trial. Institutional review boards or ethics committees at each participating site approved the trial protocol. All patients provided written informed consent. The design of the CARDIO-TTRansform trial and baseline characteristics of the trial population have been previously reported in detail^19,21^.

### Trial participants

Inclusion and exclusion criteria have already been published^19,21^. In brief, patients aged 18–90 years who had mild or moderate heart failure (NYHA functional class I–III) due to variant or wild-type ATTR-CM, as evidenced by a history of at least one heart failure hospitalization, or heart failure signs and symptoms requiring loop diuretic therapy, were eligible for enrolment. The diagnosis of ATTR-CM was established by the presence of TTR amyloid deposits in cardiac or non-cardiac tissue confirmed by Congo Red (or equivalent) staining, or technetium scintigraphy with grade 2 or 3 cardiac uptake in the absence of an abnormal light chain ratio that was centrally confirmed. Patients were also required to have confirmed cardiac involvement based on left ventricular end-diastolic interventricular septum thickness > 12 mm on a screening echocardiogram and NT-proBNP concentration of ≥600 pg ml^−1^ (or ≥1,200 pg ml^−1^ if the patient was in atrial fibrillation) and a demonstrated ability to walk at least 100 m on the 6MWT. Key exclusion criteria included NYHA functional class IV, cardiomyopathy not primarily caused by ATTR-CM and an eGFR < 30 ml min^−1^ per 1.73 m^2^.

### Trial procedures

Patients who met eligibility requirements were randomly assigned in a 1:1 ratio to receive eplontersen 45 mg or matching placebo by subcutaneous injection once every 4 weeks for 140 weeks. Randomization was stratified by NYHA functional class (I/II versus III), ATTR variant status (variant versus wild type), 6MWT distance (≤350 m versus >350 m) and treatment with TTR stabilizer at the time of randomization (yes versus no). Following completion of the 140-week double-blind treatment period, patients elected to either enroll in an open-label extension study (NCT05667493) or end their participation with a 20-week post-treatment evaluation period (during which time no study drug was administered). Concomitant administration of locally standard-of-care TTR stabilizer therapy for ATTR-CM (for example, tafamidis free acid, tafamidis meglumine and acoramidis) was allowed per investigator’s recommendation.

### Outcomes

The primary endpoint of the CARDIO-TTRansform trial was a composite of CV mortality and recurrent CV clinical events (that is, total CV clinical events) assessed up to week 140. CV clinical events in the composite endpoint included hospitalizations due to heart failure or urgent visits requiring intravenous diuretics for heart failure, myocardial infarction, stroke, transient ischemic attack and arrhythmia-related hospitalizations. Deaths from CV or undetermined causes, heart transplantation, or intermediate or long-term cardiac mechanical circulatory support (that is, left ventricular assist device implantation) were counted as CV deaths. Secondary endpoints included a change in 6MWT distance and KCCQ-OS score from baseline at week 140, CV events up to week 140, and all-cause and CV mortality up to week 160 and week 140. The primary efficacy composite endpoint of CARDIO-TTRansform was planned to be formally tested in the subgroup of patients receiving a TTR stabilizer at baseline as a secondary endpoint in the statistical testing hierarchy.

In this analysis, we evaluated the treatment effect of eplontersen versus placebo on the primary and secondary endpoints in patients who were and were not receiving TTR stabilizer therapy at baseline. These were prespecified subgroup analyses that were included in the statistical analysis plan finalized before database lock.

### Statistical analysis

The interaction between treatment with TTR stabilizer therapy at baseline and primary and secondary endpoints of the trial, along with change in NT-proBNP levels, was assessed. The statistical models used to evaluate the primary and secondary endpoints in patients receiving or not receiving baseline TTR stabilizer were identical to those used for evaluation in the overall trial population (with the exception that baseline tafamidis use was not included as a covariate), as previously described in detail^19,21^. Analyses were conducted using the full analysis set (all randomized patients who received at least one dose of study drug or placebo and who had at least one post-baseline primary efficacy assessment or one post-baseline study visit (that is, post-baseline CV clinical event, post-baseline study visit and vital status), receiving or not receiving TTR stabilizer therapy at baseline). The treatment effect of the primary endpoint (composite of CV mortality and recurrent CV clinical events) was estimated using the semi-parametric proportional rates method (Lin, Wei, Yang and Ying (LWYY))^22^ and shown using cumulative event curves via the Nelson–Aalen method. Changes from baseline at week 140 for the continuous 6MWT and KCCQ-OS endpoints were analyzed in the full analysis set using an analysis of covariance model including treatment; baseline values; the stratification factors of NYHA functional class (I/II versus III), ATTR variant status (variant versus wild type) and 6MWT distance (≤350 m versus >350 m); and the interaction of current treatment with TTR stabilizer (yes versus no) at baseline times treatment as covariates. The relative change in serum NT-proBNP between baseline and week 140 was assessed using mixed models for repeated measures, which did not include any random effects but included fixed effects for treatment group, visit, stratification factors, a treatment-by-visit interaction and baseline value. Serum TTR was assessed using mean change from baseline.

Baseline characteristics were summarized using descriptive statistics stratified by baseline stabilizer status. Continuous variables were reported as means with standard deviations or medians with IQRs and ranges, as appropriate; categorical variables were summarized as counts and percentages. Safety analyses were descriptive and included patients who received at least one dose of study drug. Treatment-emergent adverse events (serious, fatal or leading to treatment discontinuation) were summarized by treatment group.

For between-group comparisons, two-sided *P* values of ≤0.05 were considered to indicate statistical significance. As the trial did not meet its primary endpoint, all reported *P* values <0.05 were considered nominally significant and hypothesis generating, but this did not formally indicate statistical significance. Statistical analyses were performed using STATA (version 19).

The protocol and statistical analysis plan are available in the Supplementary Information.

### Reporting summary

Further information on research design is available in the Nature Portfolio Reporting Summary linked to this article.

## Online content

Any methods, additional references, Nature Portfolio reporting summaries, source data, extended data, supplementary information, acknowledgements, peer review information; details of author contributions and competing interests; and statements of data and code availability are available at https://doi.org/10.1038/s41591-026-04670-6.

## Supplementary information

Supplementary Information Protocol and statistical analysis plan.

Reporting Summary

## Data Availability

Data requests from qualified researchers will be considered once all three of the following criteria are met: (1) 12 months from marketing approval of the study drug in both the USA and the European Union, (2) 18 months from conclusion of the study, and (3) 6 months from the publication of this article. Data access is managed to uphold scientific rigor and merit, protect patient data and avoid potential conflicts of interest or competitive concerns. Data requests are reviewed and processed in a timely manner. For additional information, conditions of data access and contact details for access requests, visit https://vivli.org/ourmembers/ionis.

## Extended data

**Extended Data Table 1:** Comparison of baseline demographic and clinical characteristics in patients without and with stabilizers at baseline

	No baseline stabilizer *n* = 613	Baseline stabilizer *n* = 819	*P* value
Age, years, mean ± SD	77.6 ± 6.4	75.5 ± 7.5	< 0.001
Male sex, *n* (%)	543 (88.6%)	754 (92.1%)	0.026
Modified BMI (kg/m^2^), mean ± SD	1,111.7 ± 178.3	1,122.2 ± 192.7	0.29
Race, *n* (%)			< 0.001
Asian	5 (0.8%)	30 (3.7%)	
Black or African American	26 (4.2%)	85 (10.4%)	
Multiple	2 (0.3%)	3 (0.4%)	
Other	5 (0.8%)	5 (0.6%)	
White	575 (93.8%)	696 (85.0%)	
Hispanic or Latino	55 (9.0%)	42 (5.1%)	0.004
Region, n (%)			< 0.001
North America	31 (5.1%)	523 (63.9%)	
Europe	446 (72.8%)	230 (28.1%)	
Rest of world	136 (22.2%)	66 (8.1%)	
Duration between diagnosis and enrolment, years	0.5 (0.3–1.1)	1.1 (0.6–2.4)	< 0.001
Stabilizer termination during the trial and after first dose of study drug, *n* (%)^a^	0 (0)	51 (6.2%)	
6MWT distance, m, mean ± SD	336.9 ± 92.6	363.7 ± 97.4	< 0.001
KCCQ-OS score, mean ± SD	71.1 ± 20.2	73.7 ± 19.6	0.018
NYHA functional class, *n* (%)^b^			0.78
Class I	74 (12.1%)	118 (14.4%)	
Class II	440 (71.8%)	557 (68.0%)	
Class III	99 (16.2%)	144 (17.6%)	
NAC disease stage, *n* (%)^c^			0.002
Stage I	357 (58.2%)	539 (65.8%)	
Stage II	187 (30.5%)	214 (26.1%)	
Stage III	69 (11.3%)	66 (8.1%)	
ATTR-CM wild-type from CRF *n* (%)	548 (89.4%)	673 (82.2%)	< 0.001
TTR genotype – Variant (hereditary) *n* (%)			0.39
Val122lle (p.Val142Ile)	39 (63.9%)	91 (70.5%)	
Ile68Leu (p.Ile88Leu)	7 (11.5%)	11 (8.5%)	
Thr60Ala (p.Thr80Ala)	5 (8.2%)	9 (7.0%)	
Val30Met (p.Val50Met)	10 (16.4%)	18 (14.0%)	
Other	4 (6.2%)	17 (11.6%)	
NT-proBNP (pg/ml)	2,237 (1,356–3,966)	1,856 (1,263–3,017)	< 0.001
NT-proBNP, pg/ml > 8,500, *n* (%)	34 (5.5%)	17 (2.1%)	< 0.001
Troponin, pg/ml	54 (38–73)	47 (34–64)	< 0.001
eGFR (ml min^−1^per 1.73 m^2^), mean ± SD	62.1 ± 15.8	61.1 ± 16.6	0.23
eGFR < 60 (ml min^−1^ per 1.73 m^2^) *n* (%)	289 (47.1%)	412 (50.3%)	0.24
eGFR < 45 (ml min^−1^ per 1.73 m^2^) *n* (%)	90 (14.7%)	139 (17.0%)	0.24
LV ejection fraction, %, mean ± SD	56.4 ± 9.3	55.5 ± 10.1	0.08
LV wall thickness, mm, mean ± SD	18.5 ± 2.8	18.2 ± 2.9	0.08
Global longitudinal strain, %, mean ± SD	–14.7 ± 3.0	–14.4 ± 3.2	0.09
History of atrial fibrillation, *n* (%)	370 (60.4%)	537 (65.6%)	0.043
History of permanent pacemaker, *n* (%)	86 (14.0%)	187 (22.8%)	< 0.001
History of ICD, *n* (%)	29 (4.7%)	86 (10.5%)	< 0.001
Hypertension, *n* (%)	388 (63.3%)	519 (63.4%)	0.98
History of type 1 diabetes mellitus, *n* (%)	1 (0.2%)	4 (0.5%)	0.30
History of type 2 diabetes mellitus, *n* (%)	110 (17.9%)	129 (15.8%)	0.27
Diuretics (any), *n* (%)	561 (91.5%)	725 (88.5%)	0.06
High-ceiling (loop) diuretic, *n* (%)	546 (89.1%)	697 (85.1%)	0.028
Concomitant medications			
Anticoagulants/antithrombotics, *n* (%)	363 (59.2%)	511 (62.4%)	0.22
Beta-blocker, *n* (%)	290 (47.3%)	324 (39.6%)	0.003
MRA, *n* (%)	254 (41.4%)	364 (44.4%)	0.26
ACEi, *n* (%)	137 (22.3%)	105 (12.8%)	0.001
ARB, *n* (%)	147 (24.0%)	203 (24.8%)	0.73
ARNi, *n* (%)	39 (6.4%)	46 (5.6%)	0.55
SGLT2i, *n* (%)	127 (20.7%)	138 (16.8%)	0.06
GLP-1 receptor agonist, *n* (%)	4 (0.7%)	6 (0.7%)	0.86

Percentages may not total 100 due to rounding.

Baseline characteristics were summarized using descriptive statistics stratified by baseline stabilizer status. Continuous variables were reported as means with standard deviations or medians with interquartile ranges and ranges, as appropriate; categorical variables were summarized as counts and percentages.

For between-group comparisons, two-sided P values of ≤ 0.05 were considered to indicate nominal statistical significance

^a^Percentages based on the number of patients with stabilizer at baseline.

^b^NYHA functional classes were defined as: class I, no limitation of physical activity; class II, slight limitation of physical activity, with ordinary activity causing symptoms; class III, marked physical limitations, with less-than-ordinary activity causing symptoms; class IV: symptoms at rest or with any activity.

^c^NAC stages were defined as: stage 1, NT-proBNP ≤ 3,000 pg/ml and eGFR ≥ 45  ml min^−^^1^ per 1.73 m^2^; stage 2, NT-proBNP > 3,000 pg/ml or eGFR < 45  ml min^−1^ per 1.73 m^2^; stage 3, NT-proBNP > 3,000 pg/ml and eGFR < 45 ml min^−1^ per 1.73 m^2^.

ACEi, angiotensin-converting enzyme inhibitor; ARB, angiotensin II receptor blocker; ARNI, angiotensin receptor-neprilysin inhibitor; ATTR-CM, transthyretin amyloid cardiomyopathy; BMI, body mass index; CRF, case report form; eGFR, estimated glomerular filtration rate; GLP-1, glucagon-like peptide-1; ICD, Implantable Cardioverter-Defibrillator; IQR, interquartile range; KCCQ-OS, Kansas City Cardiomyopathy Questionnaire-Overall Summary; MRA, mineralocorticoid receptor antagonist; NAC, National Amyloidosis Centre; NT-proBNP, N-terminal pro-B-type natriuretic peptide; NYHA, New York Heart Association; SGLT2i, sodium-glucose transport 2 inhibitor; TTR, transthyretin.

**Extended Data Table 2:** Adverse events^a^, stratified by stabilizer use at baseline

Characteristic	No stabilizer at baseline		Stabilizer at baseline	
	Placebo (*n* = 308)	Eplontersen (*n* = 305)	Placebo (*n* = 409)	Eplontersen (*n* = 410)
Any treatment-emergent adverse event, n (%)	305 (99.0)	302 (99.0)	400 (97.8)	406 (99.0)
Any serious treatment-emergent adverse event	194 (63.0)	193 (63.3)	232 (56.7)	220 (53.7)
Treatment-emergent adverse event leading to study drug discontinuation	24 (7.8)	21(6.9)	29 (7.1)	25 (6.1)
Any adverse event of special interest	2 (0.6)	0 (0.0)	2 (0.5)	1 (0.2)

^a^All adverse events were classified as treatment-emergent adverse events and were recorded at any time on or after the date of first dose administration.

Safety analyses were descriptive and included patients who received at least one dose of study drug.

CI, confidence interval; RR, rate ratio.
