# Supplementary material for: Eplontersen with and without background transthyretin stabilizers in transthyretin amyloid cardiomyopathy: secondary analysis of a phase 3, randomized controlled trial
Source: Nat Med. 2026 Aug 30;32(11):4047–55. doi: 10.1038/s41591-026-04670-6 (PMC13768182; doi:10.1038/s41591-026-04670-6)
Supplement: Supplementary file 1 [file 41591_2026_4670_MOESM1_ESM.pdf]

nature medicine

Supplementary information https://doi.org/10.1038/s41591-026-04670-6

Eplontersen with and without background transthyretin stabilizers in transthyretin amyloid cardiomyopathy: secondary analysis of a phase 3, randomized controlled trial

In the format provided by the authors and unedited

IONIS PHARMACEUTICALS, INC.

ION-682884-CS2

A Phase 3 Global, Double-Blind, Randomized, Placebo-Controlled Study to Evaluate the Efficacy and Safety of ION-682884 in Patients with Transthyretin-Mediated Amyloid Cardiomyopathy (ATTR-CM)

Protocol Amendment 10 – 21 April 2025

EudraCT No: 2019-002835-27

EU CT No: 2024-514434-20-00

Strategy to Improve Patient Outcomes by Silencing TTR Formation in ATTR-CM

Trial Sponsor

Ionis Pharmaceuticals
2855 Gazelle Court
Carlsbad, CA 92010
Telephone Number: +01 760 931 9200
Fax Number: +01 760 603 2700

Key Sponsor Contact

[redacted]
2855 Gazelle Ct, Carlsbad, CA 92010
[redacted]

ION-682884-CS2

Protocol Amendment 10

EudraCT No: 2019-002835-27

EU CT No: 2024-514434-20-00

Clinical Phase: 3

A Phase 3 Global, Double-Blind, Randomized, Placebo-Controlled Study to Evaluate the Efficacy and Safety of ION-682884 in Patients with Transthyretin-Mediated Amyloid Cardiomyopathy (ATTR-CM)

Strategy to Improve Patient Outcomes by Silencing TTR Formation in ATTR-CM

This protocol applies to the following regions: Argentina, Australia, Brazil, Canada, Israel, Japan, United Kingdom, and United States

Protocol History:

**Argentina, Australia, Brazil, Israel:**	**USA:**
Original Protocol: 28 August 2019	Original: 28 August 2019
Amendment 1: 28 August 2019	Amendment 1: 18 October 2019
Amendment 2: 18 October 2019	Amendment 2: 13 March 2020
Amendment 3: 21 February 2020	Amendment 3: 10 November 2020
Amendment 4: 02 August 2021	Amendment 4: 30 June 2021
Amendment 5: 06 May2022	Amendment 5: 04 May 2022
Amendment 6: 13 September 2022	Amendment 6: 15 September 2022

Canada: Refer to Protocol History in Addendum 1

Japan: Refer to Protocol History in Addendum 2

United Kingdom: Refer to Protocol History in Addendum 3

Consolidated Amendment: Amendment 9: 20 November 2023

ION-682884-CS2

Protocol Amendment 10

EudraCT No: 2019-002835-27

EU CT No: 2024-514434-20-00

Clinical Phase: 3

A Phase 3 Global, Double-Blind, Randomized, Placebo-Controlled Study to Evaluate the Efficacy and Safety of ION-682884 in Patients with Transthyretin-Mediated Amyloid Cardiomyopathy (ATTR-CM)

cardio TTRansform

Strategy to Improve Patient Outcomes by Silencing TTR Formation in ATTR-CM

Sponsor

Ionis Pharmaceuticals, Inc.
Carlsbad, CA 92010

See electronic signature and date attached at end of document

[redacted]

Confidentiality Statement

This document contains confidential information of Ionis Pharmaceuticals, Inc. that must not be disclosed to anyone other than the recipient study staff and members of the independent ethics committee, institutional review board, or authorized regulatory agencies. This information cannot be used for any purpose other than the evaluation or conduct of the clinical investigation without the prior written consent of Ionis Pharmaceuticals, Inc.

PROTOCOL SIGNATURE PAGE

**Protocol Number:**	ION-682884-CS2
**Protocol Title:**	A Phase 3 Global, Double-Blind, Randomized, Placebo-Controlled Study to Evaluate the Efficacy and Safety of ION-682884 in Patients with Transthyretin-Mediated Amyloid Cardiomyopathy (ATTR-CM)
**Amendment:**	10
**Date:**	21 April 2025

I hereby acknowledge that I have read and understood the attached clinical protocol, entitled "A Phase 3 Global, Double-Blind, Randomized, Placebo-Controlled Study to Evaluate the Efficacy and Safety of ION-682884 in Patients with Transthyretin-Mediated Amyloid Cardiomyopathy (ATTR-CM) ," dated 21 April 2025, and agree to conduct the study as described herein.

I agree to comply with the International Conference on Harmonization Tripartite Guideline on Good Clinical Practice.

I agree to ensure that the confidential information contained in this document will not be used for any purpose other than the evaluation or conduct of the clinical investigation without the prior written consent of Ionis Pharmaceuticals, Inc.

____

Investigator's Signature

____ ____________________________

Investigator's Name *(please print)* Date (DD Month YYYY)

TABLE OF CONTENTS

PROTOCOL AMENDMENT 11
PROTOCOL SYNOPSIS 14
STUDY SCHEMA 23
SCHEMATIC PATIENT FLOW THROUGH THE STUDY 24
STUDY GLOSSARY 25
1. OBJECTIVES AND ENDPOINTS 28
1.1. Objectives 28
1.1.1. Primary Objectives 28
1.1.2. Secondary Objectives 28
1.1.3. Safety Objectives 28
1.1.4. Exploratory Objectives 29
1.2. Study Endpoints 29
1.2.1. Primary Endpoints 29
1.2.2. Secondary Endpoints 29
1.2.3. Safety Assessments 30
1.2.4. Exploratory Endpoints 30
2. BACKGROUND AND RATIONALE 31
2.1. Overview of Disease 31
2.2. Therapeutic Rationale 34
2.3. ION-682884 35
2.3.1. Mechanism of Action 35
2.3.2. Chemistry 35
2.3.3. Nonclinical Information 35
2.3.3.1. Nonclinical Pharmacology 36
2.3.3.2. Pharmacokinetics and Product Metabolism in Animals 36
2.3.3.3. Toxicology and Safety Pharmacology 36
2.3.4. Clinical Information 37
2.3.4.1. Clinical Pharmacology 38
2.3.4.2. Clinical Pharmacodynamics 39
2.3.4.3. Clinical Efficacy 40
2.3.4.4. Clinical Safety 40

2.4. Rationale for Dose and Schedule of Administration 42
2.5. Benefit-Risk Assessment 43
2.5.1. Benefit Assessment 43
2.5.2. Risk Assessment 44
2.5.3. Overall Assessment of Benefit:Risk 45
3. EXPERIMENTAL PLAN 46
3.1. Study Design 46
3.2. Number of Study Centers 46
3.3. Number of Patients 46
3.4. Overall Study Duration and Follow-up 46
3.4.1. Screening 46
3.4.2. Treatment Period 47
3.4.3. Post-Treatment Evaluation Period 47
3.5. End-of-Study 47
3.6. Data and Safety Monitoring Board 47
4. PATIENT ENROLLMENT 47
4.1. Screening 47
4.2. Randomization 48
4.3. Replacement of Patients 48
4.4. Unblinding of Treatment Assignment 48
5. PATIENT ELIGIBILITY 48
5.1. Inclusion Criteria 49
5.2. Exclusion Criteria 50
6. STUDY PROCEDURES 52
6.1. Study Schedule 52
6.1.1. Screening 52
6.1.2. Day 1/Randomization 52
6.1.3. Treatment Period 53
6.1.4. Week 140 54
6.1.5. Post-Treatment Evaluation Period 54
6.2. Laboratory Assessments 54
6.3. Other Study Procedures 54

6.4. Restriction on the Lifestyle of Patients 55
6.4.1. Contraception Requirements 55
7. STUDY DRUG 56
7.1. ION-682884 / Placebo 56
7.2. Packaging and Labeling 56
7.3. Study Drug Accountability 56
8. TREATMENT OF PATIENTS 57
8.1. Study Drug Administration 57
8.2. Other Protocol-Required Drugs 57
8.3. Other Protocol-Required Treatment Procedures 57
8.4. Treatment Precautions 57
8.5. Safety Monitoring Rules 57
8.5.1. Safety Monitoring Rules for Liver Chemistry Tests 58
8.5.2. Safety Monitoring Rules for Platelet Count Results 59
8.5.3. Safety Monitoring for Minor Bleeding Events 61
8.5.4. Safety Monitoring for Ocular Effects 61
8.5.5. Safety Monitoring Rules for Renal Function 61
8.6. Stopping Rules 61
8.6.1. Stopping Rules for Liver Chemistry Elevations 61
8.6.2. Stopping Rules for Renal Function Test Results 61
8.6.3. Stopping Rule for Platelet Count Results 62
8.6.4. Stopping Rule for Ocular Effects 64
8.7. Adjustment of Dose and/or Treatment Schedule 64
8.8. Discontinuation of Study Drug Administration 64
8.9. Discontinuation of Study Participation 65
8.9.1. Lost to Follow-up 65
8.10. Concomitant Therapy and Procedures 66
8.10.1. Concomitant Therapy 66
8.10.2. Concomitant Procedures 67
8.11. Treatment Compliance 67
9. SERIOUS AND NON-SERIOUS ADVERSE EVENT REPORTING 67
9.1. Sponsor Review of Safety Information 67

9.2. Regulatory Requirements 67
9.3. Definitions 68
9.3.1. Adverse Event....68
9.3.2. Adverse Drug Reaction and Suspected Unexpected Adverse Drug Reaction....68
9.3.3. Serious Adverse Event....69
9.3.4. Adverse Event of Special Interest....69
9.4. Monitoring and Recording Adverse Events 69
9.4.1. Serious Adverse Events 70
9.4.2. Non-Serious Adverse Events....70
9.4.3. Evaluation of Adverse Events (Serious and Non-Serious)....70
9.4.3.1. Relationship to the Study Drug....70
9.4.3.2. Severity 71
9.4.3.3. Action Taken with Study Drug....71
9.4.3.4. Treatment Given for Adverse Event....71
9.4.3.5. Outcome of the Adverse Event....72
9.4.3.6. Follow-up of Adverse Event....72
9.5. Procedures for Handling Special Situations 73
9.5.1. Abnormalities of Laboratory Tests....73
9.5.2. Prescheduled or Elective Procedures or Routinely Scheduled Treatments 73
9.5.3. Dosing Errors....73
9.5.4. Contraception and Pregnancy 74
9.5.5. Reporting Serious Breaches....75
10. STATISTICAL CONSIDERATIONS 75
10.1. Stratification, Subsets, and Covariates 75
10.2. Sample Size Considerations 76
10.3. Populations 76
10.4. Definition of Baseline....76
10.5. Interim Analysis and Multiplicity....76
10.6. Planned Methods of Analysis....76
10.6.1. Demographic and Baseline Characteristics 77
10.6.2. Efficacy Analysis....77
10.6.2.1. Primary Endpoint Analysis....77

10.6.2.2. Secondary Endpoints Analysis 77
10.6.2.3. Safety Assessments Analysis 78
10.6.2.4. Exploratory Endpoints Analysis 78
10.6.3. Exploratory Additional Analyses 78
10.6.3.1. Pharmacokinetic Analysis 78
10.6.3.2. Immunogenicity Analysis 78
11. INVESTIGATOR'S REGULATORY OBLIGATIONS 79
11.1. Informed Consent 79
11.2. Ethical Conduct of the Study 79
11.3. Independent Ethics Committee/Institutional Review Board 79
11.4. Patient Confidentiality 80
11.5. Publication Policy 80
12. ADMINISTRATIVE AND LEGAL OBLIGATIONS 81
12.1. Protocol Amendments 81
12.2. Study Termination 81
12.3. Study Documentation and Storage 81
12.4. Study Monitoring 82
12.5. Language 82
12.6. Compensation for Injury 83
13. REFERENCES 84
14. APPENDICES 88
APPENDIX A. SCHEDULE OF PROCEDURES 89
APPENDIX B. LIST OF LABORATORY ANALYTES 97
APPENDIX C. GRADING SCALE FOR ADVERSE EVENTS RELATING TO LABORATORY ABNORMALITIES 98
ADDENDUM 1 CANADA SPECIFIC PROTOCOL 101
ADDENDUM 2 JAPAN SPECIFIC PROTOCOL 103
ADDENDUM 3 UNITED KINGDOM SPECIFIC PROTOCOL 104

LIST OF TABLES

Table 1: Summary of Adverse Reactions per Frequency Categories 44

Table 2: Study Drug Characteristics 56

Table 3: Study Drug Dosing Information 57

Table 4: Additional Labs to be Potentially Performed in the Event of a Platelet Count $< 75 \times 10^9$/L, after Discussion with Study Medical Monitor 60

Table 5: Actions in Patients with Low Platelet Count 63

LIST OF FIGURES

Figure 1: Design of ION-682884, a GalNAc-Conjugated Chimeric 2′-MOE Phosphorothioate/Phosphate Oligonucleotide (MOE Gapmer).................................35

PROTOCOL AMENDMENT

Protocol Number: ION-682884-CS2

Protocol Title: A Phase 3 Global, Double-Blind, Randomized, Placebo-Controlled Study to Evaluate the Efficacy and Safety of ION-682884 in Patients with Transthyretin-Mediated Amyloid Cardiomyopathy (ATTR-CM)

Amendment: 10

Date: 21 April 2025

The main purpose of this amendment is to revise the secondary and exploratory objectives and endpoints of the study to provide a more thorough report of the efficacy and safety of eplontersen. In addition, this amendment removed text regarding possible early study closure for the primary endpoint analysis as this scenario is no longer being considered by the Sponsor.

Minor changes (not included in the list of changes below) have been made throughout the protocol to correct errors, provide definitions for abbreviations, and/or to improve the overall clarity of the Original Protocol but these changes do not impact subject safety, exposure, or the overall study design. The following table provides a summary list of major changes to the protocol. Where applicable, bold indicates additions and cross-through indicates deletions.

Protocol Section	Description of Change	Rationale
Throughout	**Changed:**<br>~~end of study~~ **Week 140**	This amendment removed scenarios for early study closure for primary endpoint analysis. End of study is now changed to as Week 140 (which is end of treatment).
Protocol Synopsis<br>Section 1.1.2<br>Section 1.2.2	Revised secondary objectives & endpoints:<br>**Added:**<br>All-cause death up to Week 160<br>Primary endpoint in subgroup of patients treated with tafamidis<br>CV death up to Week 160<br>**Changed:**<br>Changes from Baseline in 6MWT and KCCQ at ~~Week 121~~ **Week 140**	To provide a more thorough report of the efficacy and safety of eplontersen
Protocol Synopsis<br>Section 1.1.4<br>Section 1.2.4	Revised exploratory objectives & endpoints:<br>**Added:**<br>mBMI<br>Immunogenicity	To provide a more thorough report of the efficacy and safety of eplontersen

Protocol Section	Description of Change	Rationale
Protocol Synopsis<br>Patient Flow Diagram<br>Section 8.8<br>Section 8.9	Follow up instructions for patients who withdraw from the study were revised for clarity.	Clarification
Throughout	**Added:**<br>Instructions for collection of vital status at Week 160 and up to the Primary Completion Date.	To provide data for analysis of additional secondary endpoint analysis
Protocol Synopsis<br>Primary Endpoints<br>Section 1.2.1	**Changed:**<br>Hospitalization for arrhythmia~~s and/or an arrhythmia event requiring medical treatment in outpatient facility or ER~~	Treatment of arrhythmias on an outpatient basis are prone to subjectivity and local practice patterns and may not accurately reflect treatment effects
Protocol Synopsis<br>Safety Assessment<br>Section 1.2.3	**Deleted:**<br>~~Norfolk QOL-DN~~	Correction. Norfolk QOL-DN is included as an exploratory endpoint
Throughout	Removed text related to possible early study closure for efficacy	See main rationale
Section 2.3.4 and subsections<br>Section 2.5 and subsections	Sections revised	To be consistent with information provided in current Investigator's Brochure
Section 6.3 Electrocardiograms	**Deleted:**<br>~~The exam should be conducted before vital signs assessment and blood collection.~~	Not required
Throughout	Removed instruction for consultation with Medical Monitor prior to reinitiation of Study Drug administration	Removed per GCP ICH 4.3.1
Section 8.1	Added home healthcare provider for Study Drug administration	Clarification
Section 8.9.1	Added entire section	To provide guidance to help prevent participants from being lost to follow-up
Section 8.10.1<br>Allowed Concomitant Therapy	**Added:**<br>acoramidis, if marketing is locally approved	To add newly approved, non-RNA therapeutic to allowed standard of care
Section 8.10.1<br>Disallowed Concomitant Therapy	**Added:**<br>Amvuttra® (vutrisiran)	To add newly approved RNA therapeutic to disallowed concomitant therapy

**Protocol Section**	**Description of Change**	**Rationale**
Section 9.4.1<br>Section 9.4.2	**Added:**<br>For patients enrolled in the OLE, collection of SAEs (AEs) in ION-682884-CS2 will stop once the patient receives their first dose of eplontersen in the extension study.	To prevent duplicate collection of adverse events in the EDC for the Index Study and OLE
Section 9.4.3.5	**Changed:**<br>~~AE Persists~~ **Ongoing:**~~Patient terminates from the trial and~~ AE continues	Correction to be consistent with CRF
Section 9.5.1	**Added:**<br>These abnormal laboratory tests must be reported as AEs in accordance with Section 9.4.	Clarification
Section 9.5.5	Added entire section	Added section to be compliant with EU CTR
Section 10	Revised	Updated to be consistent with protocol revisions and revised SAP
Section 10.6.3.1	Added entire section	To provide information in the protocol for pharmacokinetic analysis
Appendix A	Deleted X for abbreviated monitoring panel at Week 137	Incorporated corrections from PCL
Appendix A	Added row for vital status and column for collection of vital status up to Primary Completion Date<br>Added footnotes 21 and 22	To provide instruction to collect vital status data for efficacy analysis; see main rationale
Appendix A	Added footnote 20	To allow for remote collection of questionnaires for patients who are unable to attend a mandatory in-clinic visit.
Appendix B	Added description about early restricted unblinded population pharmacokinetic access (ERUPA) Team to footnote 1	To add explanation about how unblinded pharmacokinetic data will be handled to prevent unblinding of the study team.
Country specific Addendum 2 Japan	Incorporated updates from PCL	Corrections
Country specific Addendum 3 UK	Incorporated updates from PCL	Corrections

PROTOCOL SYNOPSIS

**Protocol Title**	A Phase 3 Global, Double-Blind, Randomized, Placebo-Controlled Study to Evaluate the Efficacy and Safety of ION-682884 in Patients with Transthyretin-Mediated Amyloid Cardiomyopathy (ATTR-CM)
**Study Phase**	3
**Indication**	Treatment of ATTR-CM
**Primary Objectives**	• To evaluate the effect of treatment with ION-682884 compared to placebo up to Week 140 on the composite endpoint of cardiovascular (CV) death and recurrent CV clinical events in patients with ATTR-CM receiving available standard of care (SoC)
**Secondary Objectives**	• To evaluate the effect of treatment with ION-682884 compared to placebo at Week 140 Study Visit on exercise tolerance (assessed by the 6-minute walk test [6MWT]) in patients with ATTR-CM receiving available SoC<br>• To evaluate the effect of treatment with ION-682884 compared to placebo at Week 140 Study Visit on patient reported outcome (Kansas City Cardiomyopathy Questionnaire [KCCQ]) in patients with ATTR-CM receiving available SoC<br>• To evaluate the effect of treatment with ION-682884 compared to placebo up to Week 140 on recurrent CV clinical events in patients with ATTR-CM receiving available SoC<br>• To evaluate the effect of treatment with ION-682884 compared to placebo up to Week 160 on all-cause death in patients with ATTR-CM receiving available SoC (survival follow up data between Week 140 and up to Week 160 may originate during participation in CS2 post-treatment evaluation period or CS12 open-label extension study)<br>• To evaluate the effect of treatment with ION-682884 compared to placebo up to Week 140 on all-cause death in patients with ATTR-CM receiving available SoC<br>• To evaluate the effect of treatment with ION-682884 compared to placebo up to Week 140 on the composite endpoint of CV death and recurrent CV clinical events in patients with ATTR-CM when administered with tafamidis at Baseline<br>• To evaluate the effect of treatment with ION-682884 compared to placebo up to Week 160 on CV death in patients with ATTR-CM receiving available SoC (survival follow up data between Week 140 and up to Week 160 may originate during participation in CS2 post-treatment evaluation period or CS12 open label extension study)<br>• To evaluate the effect of treatment with ION-682884 compared to placebo up to Week 140 on CV death in patients with ATTR-CM receiving available SoC

PROTOCOL SYNOPSIS (CONTINUED)

**Safety Objectives**	To evaluate the safety and tolerability of ION-682884 compared to placebo in patients with ATTR-CM receiving available SoC
**Additional / Exploratory Objectives**	• To evaluate the effect of treatment with ION-682884 compared to placebo on echocardiography parameters of interest<br>• To evaluate the effect of treatment with ION-682884 compared to placebo on renal function and disease biomarkers<br>• To evaluate the effect of treatment with ION-682884 compared to placebo on the change from Baseline in NYHA class<br>• To evaluate the effect of treatment with ION-682884 compared to placebo on patient-reported outcomes questionnaires and disease scores related to quality of life (QOL) and burden of ATTR<br>• To evaluate the effect of treatment with ION-682884 compared to placebo on lower limb function test (LLF) and 10-meter walk test patients with hereditary ATTR-CM<br>• To examine the ION-682884 pharmacokinetics (PK) in patients with ATTR-CM<br>• To evaluate the immunogenicity (IM) of ION-682884<br>• To evaluate the effect of treatment with ION-682884 compared to placebo on mBMI
**Study Design**	Randomized, double-blind, placebo-controlled study of ION-682884 vs. placebo administered by SC injection once every 4 weeks for up to 140 weeks in patients with ATTR-CM receiving available background SoC.<br>Patients will be stratified at Randomization based on NYHA Classification (I and II vs. III), ATTR mutation status (mutated vs. wild-type), 6MWT (≤ 350 meters vs. > 350 meters), and current treatment with tafamidis (Yes vs. No).<br>Study Drug (ION-682884 or placebo) will be administered for up to 140 weeks (Treatment Period) followed by an additional 20 weeks of Post-Treatment Evaluation Period.<br>Concomitant administration of SoC therapies for ATTR is allowed, per Investigator's discretion, if locally approved (including compassionate access, extended access, and other), accessible, and consistent with local clinical practice (except ASO and siRNA compounds). Patients will also administer daily supplemental doses of the RDA of vitamin A.<br>An independent Data Safety Monitoring Board (DSMB) will be implemented for the study, will have access to unblinded data and will operate under a prespecified charter. An independent Clinical Adjudication Committee (CAC), blinded to treatment allocation, will adjudicate all deaths and CV clinical events. Central Readers will review cardiac imaging (echocardiography and 99m technetium scintigraphy).

PROTOCOL SYNOPSIS (CONTINUED)

**Number of Patients**	Approximately 1400 patients randomized 1:1 to either ION-682884 or placebo from multiple sites worldwide.
**Study Population**	**Inclusion Criteria**<br>To be eligible to participate in this study patients must meet the following eligibility criteria within 10 weeks prior to Study Day 1, or at the time point specified in the individual eligibility criterion listed.<br>The Medical Monitor may be consulted if any questions arise regarding the Inclusion or Exclusion criteria.<br>1. Must have given written informed consent (signed and dated) and any authorizations required by local law and be able to comply with all study requirements<br>2. 18 to 90 years of age (inclusive)<br>3. Females: must be non-pregnant and non-lactating and either:<br>a. surgically sterile (e.g., tubal occlusion, hysterectomy, bilateral salpingectomy, bilateral oophorectomy)<br>b. post-menopausal (defined as 12 months of spontaneous amenorrhea in females > 55 years of age or, in females ≤ 55 years, 12 months of spontaneous amenorrhea without an alternative medical cause <u>and</u> follicle-stimulating hormone (FSH) levels in the post-menopausal range for the laboratory involved)<br>c. abstinent* or,<br>d. if of childbearing potential and engaged in sexual relations, agree to use 1 highly effective contraceptive method from the time of signing the informed consent form until at least 24 weeks after the last dose of Study Drug (ION-682884 or placebo). See Section 6.4.1 for more details<br>Males must be surgically sterile or abstinent*; if engaged in sexual relations with a female of childbearing potential, the patient must be using an acceptable contraceptive method from the time of signing the informed consent form until at least 24 weeks after the last dose of Study Drug.<br>* Abstinence is only acceptable as true abstinence, i.e., when this is in line with the preferred and usual lifestyle of the patient. Periodic abstinence (e.g., calendar, ovulation, symptothermal, post-ovulation methods), declaration of abstinence for the duration of a trial and withdrawal are not acceptable methods of contraception<br>4. Willing to be genetically tested for mutations in the TTR gene during screening, if it was not done before<br>5. Amyloid deposits in cardiac or non-cardiac tissue confirmed by Congo Red (or equivalent) staining OR technetium scintigraphy (99mTc -3,3-diphosphono-1,2-propanodicarboxylic acid [DPD-Tc], 99m Tc-pyrophosphate [PYP-Tc], or 99mTc-hydroxymethylene-diphosphonate [HMDP-Tc]) with Grade 2 or 3 cardiac uptake in the absence of abnormal light chains ratio, centrally confirmed

PROTOCOL SYNOPSIS (CONTINUED)

**Study Population (Continued)**	**Inclusion Criteria (Continued)**<br>6. End-diastolic interventricular septum thickness of > 12 mm on Screening echocardiogram<br>7. Medical history of HF secondary to hereditary or wild-type ATTR-CM with at least: A) 1 prior hospitalization for HF, which may include hospitalization for arrhythmia or pacemaker/ICD (implantable cardioverter defibrillator) placement, OR B) symptoms and signs of volume overload or elevated intracardiac pressure that requires treatment with diuretics other than mineralocorticoid receptor antagonists (MRA) for clinical stabilization<br>8. Screening N-terminal prohormone of brain natriuretic peptide (NT-proBNP) ≥ 600 pg/mL (≥ 70 pmol/L) by central lab. For patients in atrial fibrillation at Screening the NT-proBNP value is ≥ 1200 pg/mL (≥ 140 pmol/L)<br>9. New York Heart Association (NYHA) class I-III<br>10. 6MWT ≥ 100 meters<br>11. If on medical treatment for HF, stable medication regimen for at least 2 weeks prior to randomization<br>12. Willingness to adhere to vitamin A supplementation per protocol<br>**Exclusion Criteria**<br>1. Acute coronary syndrome, unstable angina, stroke, transient ischemic attack (TIA), coronary revascularization, cardiac device implantation, cardiac valve repair, or major surgery within 3 months of Screening<br>2. Hospitalization or urgent visit to emergency department/emergency room (ED/ER) for worsening of HF within 4 weeks prior to or during Screening<br>3. Uncontrolled hypertension (systolic blood pressure [BP] > 160 or diastolic BP > 100 mm Hg)<br>4. Uncontrolled clinically significant cardiac arrhythmia, per Investigator's assessment (e.g., no pacemaker, although indicated)<br>5. Severe, uncorrected, cardiac valvular disease<br>6. Cardiomyopathy not primarily caused by ATTR-CM, for example, cardiomyopathy due to hypertension, valvular heart disease, or ischemic heart disease<br>7. Screening laboratory results as follows, or any other clinically significant abnormalities in Screening laboratory values that would render a patient unsuitable for inclusion<br>a. Alanine aminotransferase/aspartate aminotransferase (ALT/AST) > 2.0 × upper limit of normal (ULN)<br>b. Total bilirubin ≥ 2 × ULN (patients with total bilirubin ≥ 2 × ULN may be allowed on study if indirect bilirubin only is elevated, ALT/AST is not greater than the ULN, and known to have Gilbert's disease OR if, in the opinion of the Investigator, the bilirubin abnormality is deemed not clinically significant, pending local work-up and discussion with Medical Monitor.)<br>c. Platelets < 125 × 10^9/L<br>d. Urine protein creatinine ratio (UPCR) ≥ 750 mg/g. In the event of UPCR above this threshold, ineligibility may be confirmed by a repeat random spot UPCR ≥ 750 mg/g

PROTOCOL SYNOPSIS (CONTINUED)

**Study Population (Continued)**	**Exclusion Criteria (Continued)**<br>e. Positive test for blood (including trace) for blood on urinalysis. In the event of a positive test ineligibility may be that is subsequently confirmed with urine microscopy showing > 5 red blood cells per high power field and is related to glomerulopathies. In women, this exclusion criterion must be assessed outside of menstrual period. If in the opinion of the Investigator the hematuria is not considered related to glomerulopathies the patient may be considered eligible, pending proper follow up and a discussion with the medical monitor. Patients with history of bladder cancer must have been treated with curative intent and have not presented recurrence within the prior 5 years<br>f. Estimated glomerular filtration rate (eGFR) < 30 mL /min/1.73 m^2 at Screening [CKD-EPI formula; (Levey *et al.* 2009)]. If the eGFR is thought to be underestimated, the CKD-EPI creatinine-cystatin C equation can be used for confirmation (Inker *et al.* 2012)<br>g. Abnormal thyroid function tests with clinical significance per Investigator judgement<br>h. Serum retinol level at Screening < LLN (this criterion does not apply to ATTRv-CM patients with mutation at the position 84 [e.g., Ile84Ser])<br>i. Hemoglobin A1c (HbA1c) > 9.5%<br>8. Monoclonal gammopathy of undetermined significance (MGUS) and/or alterations in immunoglobulin free light chain (FLC) ratio, unless fat, bone marrow, or heart biopsy confirming the absence of light chain and the presence of TTR protein by mass spectrometry or immunoelectron microscopy. For patients with CKD and without presence of monoclonal protein in blood and urine, the acceptable FLC ratio is 0.26-2.25. Results different from that may be discussed with local hematologist and Investigator and Medical Monitor if the risks associated with the biopsy outweigh the benefits<br>9. Active infection requiring systemic antiviral or antimicrobial therapy that will not be completed prior to Study Day 1<br>10. Known history of or positive test for human immunodeficiency virus (HIV) (as evidenced by positive tests for HIV antibody and HIV RNA), hepatitis C (as evidenced by positive tests for HCV antibody and HCV RNA) or hepatitis B (as evidenced by a positive test for hepatitis B surface antigen)<br>11. History of bleeding, diathesis, or coagulopathy (e.g., liver cirrhosis, hematologic malignancy, antiphospholipid antibody syndrome, congenital disorders such as hemophilia A, B, and Von Willebrand disease)<br>12. If receiving oral anticoagulants, (except vitamin K antagonists) the dose must have been stable for 4 weeks prior to the first dose of Study Drug and regular monitoring must be performed, per clinical practice, during the study. If the patient is receiving vitamin K antagonists (e.g., warfarin) INR should be in therapeutic range, as established by the Investigator, for 4 weeks prior to the first dose

PROTOCOL SYNOPSIS (CONTINUED)

**Study Population (Continued)**	**Exclusion Criteria (Continued)**<br>13.Malignancy within 5 years, except for basal or squamous cell carcinoma of the skin, melanoma *in situ*, prostate carcinoma grade group 1, breast ductal carcinoma *in situ*, or carcinoma *in situ* of the cervix Patients with a history of other malignancies who have been treated with curative intent and without recurrence within 5 years may also be eligible per Investigator judgment<br>14. Prior liver or heart transplant, and/or Left Ventricular Assist Device (LVAD) or anticipated liver transplant or LVAD within 1 year after randomization<br>15. Karnofsky performance status of ≤ 50%<br>16. Contraindication for immunosuppressive therapy, per Investigator's discretion<br>17. Known Light chain/Primary Amyloidosis (AL)<br>18. Known leptomeningeal amyloidosis<br>19. Known history of multiple myeloma<br>20. Treatment with another investigational drug and/or biological agent within 1 month of Screening, or 5 half-lives of investigational agent, whichever is longer<br>21. Current or previous treatment with Tegsedi® (inotersen) or Onpattro® (patisiran) or other oligonucleotide or RNA therapeutic (including siRNA; does not apply to COVID-19 mRNA vaccinations)<br>22. Current treatment with diflunisal, doxycycline with or without ursodeoxycholic acid, and/or non-dihydropyridine calcium-channel blocker (e.g., verapamil, diltiazem). Patients receiving any of these agents must respect a Wash-out Period of 14 days before randomization<br>23. Unwillingness or inability to comply with study procedures, including follow-up, as specified by this protocol, or unwillingness to cooperate fully with the Investigator<br>24. Other physical, social, or psychological conditions including illicit drug or alcohol use, which, in the opinion of the Investigator would make the patient unsuitable for inclusion, or could interfere with the patient participating in or completing the study
**Treatment Groups**	Patients will be randomized to receive either ION-682884 45 mg or placebo SC once every 4 weeks at a 1:1 ratio
**Study Drug Dosage and Administration**	Study Drug (0.8 mL vial of ION-682884 150 mg/mL, or placebo) will be supplied by the Sponsor. All Study Drug injections will be administered SC either in the clinic or at home by patient, caregiver, or Sponsor designee.
**Rationale for Dose and Schedule Selection**	The dose was chosen based on the pharmacodynamic and safety analysis of the ION-682884-CS1 study in healthy volunteers.
**Adjustment of Dose and/or Treatment Schedule**	Patients will receive ION-682884 or placebo SC every 4 weeks. Dose frequency adjustments, including dose interruptions, may be allowed for safety or tolerability, per discussion with Medical Monitor.

PROTOCOL SYNOPSIS (CONTINUED)

**Study Visit Schedule and Procedures**	The study will consists of the following periods: up to 10 weeks of Screening Period, up to 140 weeks of Treatment Period, and 20 weeks for Post-Treatment Evaluation Period.<br>Patients who had Study Drug administration paused or discontinued permanently for any reason (i.e., due to adverse events [AE], protocol requirement, patient request, or Sponsor decision) other than withdrawal of consent from study participation, should continue their participation in the study by attending visits and procedures, if possible, and by providing vital status at the time the patient was expected to reach the end of their Treatment Period (Week 140) at the end of their Post Treatment Evaluation Period (Week 160), and up to the Primary Completion Date of the study. This is the preferred follow-up approach, and **Investigators should encourage participants to continue in the study as described above** (Section 8.8).<br>Patients who withdraw consent to participate in the study should be encouraged to complete the Early Termination Visit at the time of withdrawal (Appendix A). These patients will be removed from further treatment and study observation immediately upon the date of request. However, vital status may be obtained through indirect means if permitted by local laws of jurisdiction (Section 8.9).<br>The Primary Completion Date, defined as "the date on which the last patient in the clinical study was examined or received an intervention/treatment to collect final data for the primary outcome measures" is the last patient's last visit of the Treatment Period (Week 140).<br>Following the Treatment Period and Week 140 visit, patients will enter the 20-week Post-Treatment Evaluation Period. This period consists of 2 in-Clinic Study visits on Weeks 150 and 160 as outlined in the Schedule of Procedures (Appendix A).<br>Alternatively, after completion of the Treatment Period, eligible patients may elect to receive ION-682884 in an open-label extension (OLE) study, pending study approval by the Institutional review Board/Independent Ethics Committee (IRB/IEC) and the appropriate regulatory authority. In this case, patients may not participate in any or only some of the Post-Treatment Evaluation Period assessments. All participating patients in the OLE study will receive ION-682884 SC once every month.<br>The vital status up to the Primary Completion Date of all study participants exposed to Study Drug will be determined where possible and regardless of the patient's End-of Study status except those participants active in this study or in the OLE at the time of determination and except those participants already with known date of death.

PROTOCOL SYNOPSIS (CONTINUED)

<table>
<tr><td>Primary Endpoints</td><td>Composite endpoint of CV death and recurrent CV clinical events comparing the 2 study arms up to Week 140 of study.
Cardiovascular clinical events include:
• Hospitalization for myocardial infarction (MI)
• Hospitalization for HF exacerbation
• Hospitalization for arrhythmia
• Hospitalization for stroke/TIA
• HF urgent, nonscheduled, visits to ED/ER or HF clinics requiring administration of intravenous (IV) diuretics for improvement
All deaths and CV clinical events will be adjudicated by an independent CAC using the ‘2017 Cardiovascular and Stroke Endpoint Definitions for Clinical Trials’ (Hicks et al. 2018).</td></tr>
<tr><td>Secondary Endpoints</td><td>• Changes from Baseline in the 6MWT distance at Week 140 Study Visit.
• Changes from Baseline in KCCQ overall summary scores at Week 140 Study Visit
• Recurrent CV clinical events up to Week 140
• All-cause death up to Week 160
• All-cause death up to Week 140
• Primary endpoint in subgroup of patients treated with tafamidis at Baseline
• CV death up to Week 160
• CV death up to Week 140</td></tr>
<tr><td>Safety Assessments</td><td>Safety laboratory tests (e.g., serum creatinine, UPCR, hematology including platelet count, thyroid function), vitals, ECG, AEs.</td></tr>
</table>

PROTOCOL SYNOPSIS (CONTINUED)

**Additional/ Exploratory Endpoints**	• Changes from Baseline in echocardiography parameters of interest including, but not limited to, Global Longitudinal Strain, Interventricular Septum Thickness, Posterior Wall Thickness, Mean Left Ventricle Wall Thickness, Stroke Volume, Ejection Fraction, Left Ventricular Mass, Left Ventricular End Diastolic Diameter (LVEDD), Left Ventricle End Diastolic Volume (LVEDV), Diastolic parameters, Right Ventricle parameters<br>• Changes from Baseline in renal function (eGFR) and biomarkers (NT-proBNP, high sensitivity cardiac troponin T [hs-cTnT], CK-MB, Galectin-3, soluble interleukin-1 receptor-like 1 [sST2], TTR)<br>• Change from Baseline in NYHA class<br>• Changes from Baseline of patient-reported outcomes questionnaires and disease scores<br>– Karnofsky performance status, SF-36 survey, EQ 5D-5L, Norfolk QOL-DN, Patient Global Impression of Severity (PGIS), and Patient Global Impression of Change (PGIC) will be measured in the overall population<br>– Polyneuropathy Disability (PND) score and Composite Autonomic Symptom Score (COMPASS-31), will be measured only in patients with hereditary ATTR-CM<br>• Changes from Baseline in lower limb function test (LLF) and 10-meter walk test in patients with hereditary ATTR-CM<br>• Evaluation of ION-682884 PK in patients with ATTR-CM<br>• Evaluation of immunogenicity (IM) in patients with ATTR-CM<br>• Changes from Baseline on mBMI
**Statistical Considerations**	The sample size calculation assumes treatment effect of 20% reduction in the relative risk of CV death and recurrent CV clinical events of treatment arm compared with placebo over a period up to 140 weeks. Using a two-sided test with an alpha level of 0.05, a sample size of approximately 1400 will provide a power of ~85% with treatment duration of 140 weeks considering a drop-out of 5–10%.
**Sponsor**	Ionis Pharmaceuticals

STUDY SCHEMA

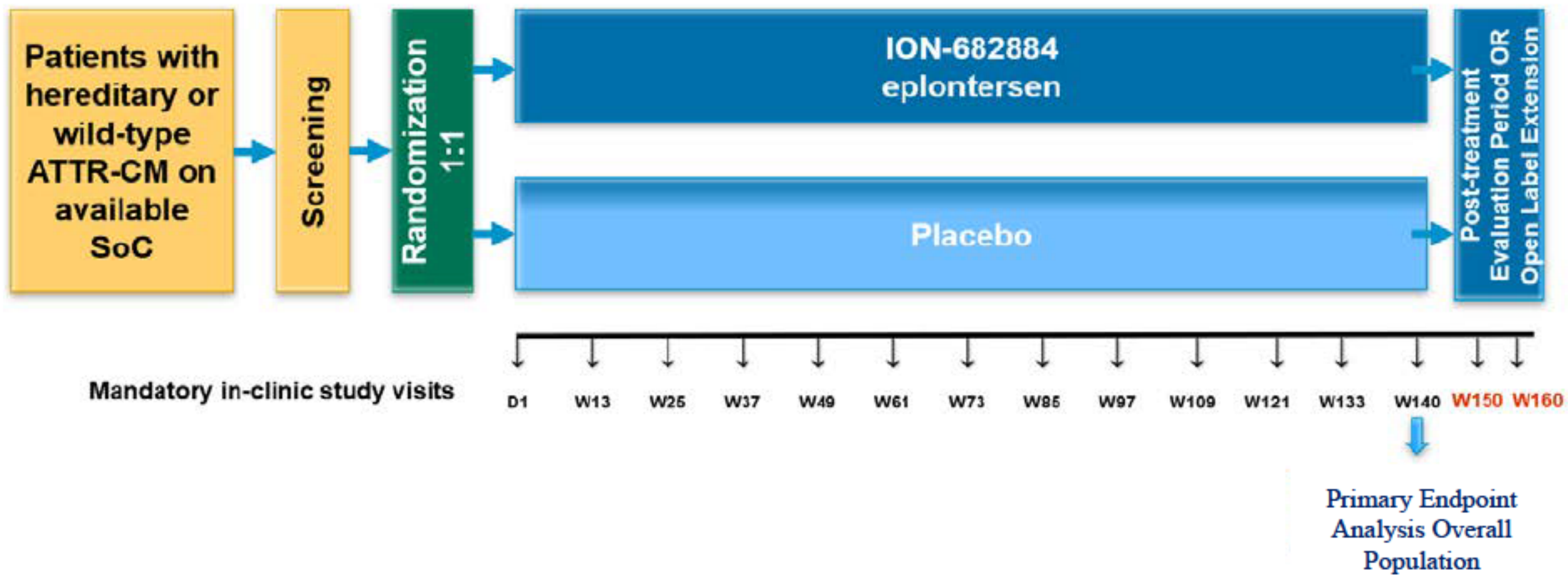

SCHEMATIC PATIENT FLOW THROUGH THE STUDY

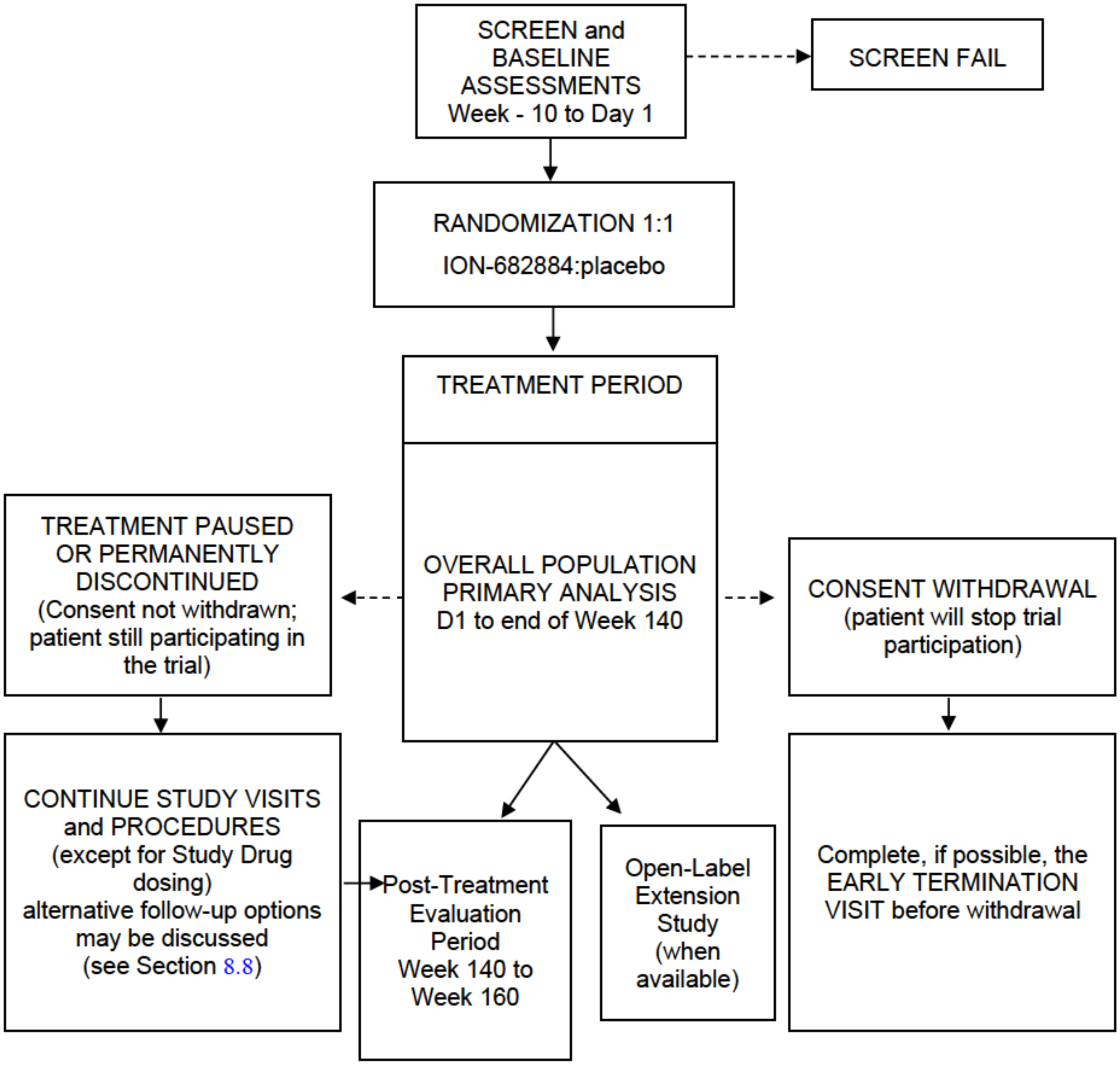

STUDY GLOSSARY

Abbreviation	Definition
2′-MOE	2′-*O*-(2-methoxyethyl)
6MWT	6-minute walk test
ADA	anti-drug antibodies
AE	adverse event
AESI	adverse event of special interest
AL	light chain amyloidosis
ALP	alkaline phosphatase
ALT	alanine aminotransferase (SGPT)
ANA	antinuclear antibody
Anti-PF4	heparin-induced platelet antibody
aPTT	activated partial thromboplastin time
ASO	antisense oligonucleotide
AST	aspartate aminotransferase (SGOT)
ATTR-CM	Transthyretin-Mediated Amyloid Cardiomyopathy
ATTR-PN	Transthyretin-Mediated Amyloid Polyneuropathy
ATTRv	Hereditary Transthyretin-mediated amyloidosis
ATTRwt	Wild type Transthyretin-mediated amyloidosis
Bb	complement factor Bb (activated complement split product)
BE	bio-equivalence
βhCG	beta-subunit of human chorionic gonadotropin (pregnancy test)
BP	blood pressure
C5a	complement factor C5a (activated complement split product)
CAC	Clinical Adjudication Committee
CBC	complete blood count
CIR	copy increment from reference
CKD-EPI	Chronic Kidney Disease Epidemiology Collaboration
CK-MB	creatine kinase-muscle/brain fraction
CMV	cytomegalovirus
CRNMB	clinically relevant non-major bleeding
CRP	C-reactive protein
CSR	Clinical study report
CTCAE	Common Terminology Criteria for Adverse Events
CV	cardiovascular
DSMB	Data and Safety Monitoring Board
ECG	electrocardiogram
eCRF	electronic case report form
ED/ER	emergency department/emergency room
EDC	electronic data capture
eGFR	estimated glomerular filtration rate
eplontersen	nonproprietary name for ION-682884
ERUPA	early restricted unblinded population pharmacokinetic access
ETT	Early Treatment Termination
FSH	follicle-stimulating hormone
FLC	free light chain

Abbreviation	Definition
GCP	Good Clinical Practice
GDPR	General data protection regulation
HAV	hepatitis A virus
HbA1c	hemoglobin A1c
HBsAg	hepatitis B surface antigen
HBV	hepatitis B virus
HCV	hepatitis C virus
HF	heart failure
HIV	human immunodeficiency virus
hsCRP	CRP measured by high sensitivity assay
Hs-cTnT	high sensitivity cardiac troponin T
IA	interim analysis
IB	Investigator Brochure
ICD	implantable cardioverter defibrillator
ICH	International Conference on Harmonization
IDMC	Independent Data Monitoring Committee
IEC	Independent Ethics Committee
IgM	immunoglobulin M
INR	international normalized ratio
ION-682884	Sponsor compound code for eplontersen (antisense inhibitor of transthyretin)
IRB	Institutional Review Board
IV	intravenous(ly)
IVRT	Isovolumic Relaxation Time
IXRS	interactive voice/internet response system
KCCQ	Kansas City Cardiomyopathy Questionnaire
LLF	lower limb function
LLN	lower limit of normal
LSM	Least square mean
LVEDD	Left Ventricular End Diastolic Diameter
LVEDV	Left Ventricle End Diastolic Volume
mBMI	modified body mass index (BMI × serum albumin g/L)
MCH	mean corpuscular hemoglobin
MCHC	mean corpuscular hemoglobin concentration
MCV	mean corpuscular volume
MedDRA	Medical Dictionary for Regulatory Activities
MI	myocardial infarction
MGUS	monoclonal gammopathy of undetermined significance
MRA	mineralocorticoid receptor antagonist
mRNA	messenger ribonucleic acid
NCS	not clinically significant
NOAEL	no observed adverse effect level
NSAID	non-steroidal anti-inflammatory drug
NT-proBNP	n-terminal prohormone of brain natriuretic peptide
NYHA	New York Heart Association
OAEI	other adverse events of interest

Abbreviation	Definition
OLE	open-label extension
OLT	orthotopic liver transplantation
on study	The patient is ‘on study’ from signing of the informed consent until their last study visit
PCL	Protocol Clarification Letter
PD	pharmacodynamic(s)
PGIC	Patient Global Impression of Change
PGIS	Patient Global Impression of Severity
pH	measure of the acidity or basicity of a solution
PK	pharmacokinetic(s)
PND	polyneuropathy disability score
PT	prothrombin time
QoL	quality of life
RBP4	retinol binding protein 4
RDA	recommended daily allowance
rINN	recommended international nonproprietary name
RNase H1	an ubiquitous endonuclease that specifically hydrolyzes the RNA strand in RNA/DNA hybrids
RoW	Rest of the World
SAE	serious adverse event
SAP	Statistical Analysis Plan
SC	subcutaneous(ly)
siRNA	small interfering ribonucleic acid
SoC	standard of care
sST2	soluble interleukin 1 receptor like 1
Study Day 1	defined as the first day Study Drug product is administered to the patient
Study Drug	ION-682884 or placebo
SUSAR	suspected unexpected serious adverse reaction
TEAE	treatment-emergent adverse event
TIA	transient ischemic attack
TSH	thyroid stimulating hormone
ULN	upper limit of normal
UPCR	urine protein creatinine ration
USAN	United States accepted name
WBC	white blood cell

1. OBJECTIVES AND ENDPOINTS

1.1. Objectives

1.1.1. Primary Objectives

- To evaluate the effect of treatment with ION-682884 compared to placebo up to Week 140 of the study on the composite endpoint of cardiovascular (CV) death and recurrent CV clinical events in patients with transthyretin-mediated amyloid cardiomyopathy (ATTR-CM) receiving available standard of care (SoC)

1.1.2. Secondary Objectives

- To evaluate the effect of treatment with ION-682884 compared to placebo at Week 140 Study Visit on exercise tolerance (6MWT) in patients with ATTR-CM receiving available SoC
- To evaluate the effect of treatment with ION-682884 compared to placebo at Week 140 Study Visit on patient reported outcome (KCCQ) in patients with ATTR-CM receiving available SoC
- To evaluate the effect of treatment with ION-682884 compared to placebo up to Week 140 on recurrent CV clinical events in patients with ATTR-CM receiving available SoC
- To evaluate the effect of treatment with ION-682884 compared to placebo up to Week 160 on all-cause death in patients with ATTR-CM receiving available SoC (survival follow up data between Week 140 and up to Week 160 may originate during participation in CS2 post-treatment evaluation period or CS12 open-label extension study)
- To evaluate the effect of treatment with ION-682884 compared to placebo up to Week 140 on all-cause death in patients with ATTR-CM receiving available SoC
- To evaluate the effect of treatment with ION-682884 compared to placebo up to Week 140 on the composite endpoint of CV death and recurrent CV clinical events in patients with ATTR-CM when administered with tafamidis at Baseline
- To evaluate the effect of treatment with ION-682884 compared to placebo up to Week 160 on CV death in patients with ATTR-CM receiving available SoC (survival follow up data between Week 140 and up to Week 160 may originate during participation in CS2 post-treatment evaluation period or CS12 open-label extension study)
- To evaluate the effect of treatment with ION-682884 compared to placebo up to Week 140 on CV death in patients with ATTR-CM receiving available SoC

1.1.3. Safety Objectives

- To evaluate the safety and tolerability of ION-682884 compared to placebo in patients with ATTR-CM receiving available SoC

1.1.4. Exploratory Objectives

- To evaluate the effect of treatment with ION-682884 compared to placebo on echocardiography parameters of interest
- To evaluate the effect of treatment with ION-682884 compared to placebo on renal function and disease biomarkers
- To evaluate the effect of treatment with ION-682884 compared to placebo on the change from Baseline in NYHA class
- To evaluate the effect of treatment with ION-682884 compared to placebo on patient reported outcomes questionnaires and disease scores related to quality of life (QOL) and burden of ATTR
- To evaluate the effect of treatment with ION-682884 compared to placebo on lower limb function test (LLF) and 10-meter walk test patients with hereditary ATTR-CM
- To examine ION-682884 pharmacokinetics (PK) in patients with ATTR-CM
- To evaluate the immunogenicity (IM) of ION-682884
- To evaluate the effect of treatment with ION-682884 compared to placebo on mBMI

1.2. Study Endpoints

1.2.1. Primary Endpoints

Composite endpoint of CV-death and recurrent CV clinical events comparing the 2 study arms up to Week 140. Cardiovascular clinical events include:

- Hospitalization for myocardial infarction (MI)
- Hospitalization for heart failure (HF)
- Hospitalization for arrhythmias
- Hospitalization for stroke/ Transient Ischemic Attack (TIA)
- HF urgent visits to Emergency Department/Emergency Room (ED/ER) or HF clinics requiring administration of intravenous (IV) diuretics for improvement.

All deaths and CV clinical events will be adjudicated by an independent Clinical Adjudication Committee (CAC) using the '2017 Cardiovascular and Stroke Endpoint Definitions for Clinical Trials' (Hicks *et al.* 2018).

1.2.2. Secondary Endpoints

- Changes from Baseline in the 6MWT distance at Week 140 Study Visit
- Changes from Baseline in the Kansas City Cardiomyopathy Questionnaire (KCCQ) overall summary score at Week 140 Study Visit
- Recurrent CV clinical events up to Week 140
- All-cause death up to Week 160

- All-cause death up to Week 140
- Primary endpoint in subgroup of patients treated with tafamidis at Baseline
- CV death up to Week 160
- CV death up to Week 140

1.2.3. Safety Assessments

Safety laboratory tests (e.g., serum creatinine, urine protein creatinine ratio [UPCR], hematology including platelet count, thyroid function), vital signs, electrocardiogram (ECG) parameters, and AEs.

1.2.4. Exploratory Endpoints

- Changes from Baseline in echocardiography parameters of interest including, but not limited to, Global Longitudinal Strain, Interventricular Septum Thickness, Posterior Wall Thickness, Mean Left Ventricle Wall Thickness, Stroke Volume, Ejection Fraction, Left Ventricular Mass, Left Ventricular End Diastolic Diameter (LVEDD), Left Ventricle End Diastolic Volume (LVEDV), Diastolic parameters, Right Ventricle parameters
- Changes from Baseline in renal function (eGFR) and biomarkers (N-terminal prohormone of brain natriuretic peptide [NT-proBNP], high sensitivity cardiac troponin T [hs-cTnT], creatine kinase-muscle/brain fraction [CK-MB], Galectin-3, soluble interleukin-1 receptor like 1 [sST2], TTR)
- Change from Baseline in NYHA class
- Changes from Baseline in patient-reported outcomes questionnaires and disease scores
  - Karnofsky performance status, SF-36 survey, EQ-5D-5L, and Norfolk QOL-DN, Patient Global Impression of Severity (PGIS), and Patient Global Impression of Change (PGIC) will be assessed periodically in the overall population
  - Polyneuropathy Disability (PND) and Composite Autonomic Symptom Score (COMPASS-31) will be assessed periodically only in patients with hereditary ATTR-CM
- Changes from Baseline in LLF and 10-meter walk test in patients with hereditary ATTR-CM
- Evaluation of ION-682884 PK in patients with ATTR-CM
- Evaluation of immunogenicity (IM) on ION-682884
- Changes from Baseline in mBMI

2. BACKGROUND AND RATIONALE

2.1. Overview of Disease

Transthyretin amyloidosis cardiomyopathy (ATTR-CM) is a progressive, irreversible, and often fatal condition characterized by extracellular amyloid deposits derived from plasma transthyretin (TTR) into the myocardium. Two (2) forms of ATTR-CM have been described: sporadic, caused by deposition of wild-type (non-mutant) TTR (ATTRwt-CM) and hereditary (ATTRv-CM), associated with autosomal dominant mutations of TTR gene that destabilize the typical tetrameric structure of the TTR protein, causing its dissociation into free monomers. These monomers misfold and subsequently aggregate into insoluble, extracellular amyloid fibers deposits, causing cell degeneration and death (Plante-Bordeneuve & Said 2011; Quintas *et al.* 2001; Ruberg *et al.* 2012). Due to the multiple organ deposition of amyloid, the disease has a broad spectrum of clinical manifestations. However, historically, hereditary ATTR (ATTRv) has been divided into 2 major phenotypes: ATTRv with polyneuropathy (ATTRv-PN), when the peripheral nerves, including the autonomic nervous system, and gastrointestinal tract are predominantly involved, and ATTRv-CM, when the heart is predominantly involved.

Hereditary ATTR-CM has been estimated to occur in 80% of ATTRv amyloidosis with the most common TTR mutations being Val122Ile (V122I; occurring in ~3.5% of African American) and Leu111Met (L111M). A mixed phenotype of polyneuropathy and cardiomyopathy is more frequently seen with the E89Q and T60A mutations (Rapezzi *et al.* 2009). In ATTRwt patients the prevalence of cardiomyopathy increases with advancing age, and the disease is typically diagnosed in men aged > 70 years, hence the previous name of "senile systemic amyloidosis" (Falk 2011; Ng *et al.* 2005). The infiltration of the myocardium by non-mutated TTR amyloid fibers has been reported in up to 25% of elderly people in autopsy studies (Cornwell *et al.* 1983). Epidemiological data suggest that up to 13% of patients with HF with preserved ejection fraction (HFpEF) above 60 years old have TTRwt-CM as an undiagnosed condition (González-López *et al.* 2015). However, in the recent years the diagnosis of ATTRwt-CM has become more straightforward due to the repurposing of bone scintigraphy (technetium scintigraphy using 99mTc -3,3-diphosphono-1,2-propanodicarboxylic acid [DPD-Tc] or 99m Tc-pyrophosphate [PYP-Tc]) towards the diagnosis of this condition. These tests are non-invasive, very sensitive, and have been supporting the increase of awareness of ATTRwt-CM among cardiologists (Gillmore *et al.* 2016).

There is no widely accepted method for measuring progression of cardiac disease in ATTR-CM. However, recent studies have shown that strain imaging with echocardiography or MRI can detect changes within a 1-year time frame (Benson *et al.* 2017; Falk 2011). New staging systems have been proposed to provide better risk stratification and prognosis prediction. The staging systems based on the association of NT-proBNP and eGFR (Gillmore *et al.* 2018) and NT-proBNP and cardiac troponin-T (Grogan *et al.* 2016) have been able to stratify patients with higher mortality risk.

Because the pathology of ATTRwt-CM has no association with genetic mutations, its geographical distribution is expected to be worldwide. The actual prevalence and incidence of ATTRwt-CM are unknown.

Irrespective of whether due to mutant or wild type, ATTR-CM presents as restrictive cardiomyopathy, which can lead to cardiac hypertrophy, HFpEF, and ultimately, heart transplant or death (Castaño *et al.* 2015). Conduction abnormalities and arrhythmias are also common, and many patients require pacemaker and/or defibrillator insertion. ATTR-CM significantly impacts patients' functional capacity and QOL (Dungu *et al.* 2012). As expected, given the severity of ATTR, there is a significant impact on caregivers' QOL (Conceição *et al.* 2016; Stewart *et al.* 2013).

Overview of Target – Transthyretin

The name TTR is derived from transporter of thyroxine and retinol and was formerly known as prealbumin. The human *ttr* gene is over 7 kb in length, has 4 exons, and is found on chromosome *18q12.1*. It is synthesized primarily in the liver and, to a lesser extent, by the choroid plexus and retina. It is secreted as a tetramer by the liver into the plasma as a 55 kDa protein composed of 4 identical subunits of 14 kDa each. A major function of TTR in the plasma is to transport retinol to tissues through its association with retinol binding protein 4 (RBP4). Retinol is the major circulating form of vitamin A. About 70% of total body retinol is stored in the liver and mobilized into the blood stream bound to RBP4 (D'Ambrosio *et al.* 2011). The retinol-RBP4 complex in turn binds to TTR, which prevents the complex from being excreted through the kidney. The ratio RBP4:TTR in plasma is around 0.3 in healthy individuals, indicating that most of the circulating TTR remains free of RBP4 ligand (Buxbaum & Reixach 2009). Besides transporting the retinol-RBP4 complex, TTR also transports ~15% of plasma thyroxine with the majority of thyroxine transported by either thyroxine binding globulin or albumin.

Recent evidence has suggested that TTR may also have a neuroprotective function in the brain. Brain TTR is produced primarily by the choroid plexus and has been proposed to help with nerve regeneration and to play a protective role in the Alzheimer's disease (Buxbaum & Reixach 2009; Fleming *et al.* 2009). It has been demonstrated that mice treated systemically with a TTR-specific ASO had decreased hepatic TTR messenger ribonucleic acid (mRNA) levels and serum TTR concentration but did not show changes in the expression of TTR in the choroid plexus (Benson *et al.* 2006; 2017).

Current Therapies

Most of the current therapeutic strategies to treat ATTR are indicated to the polyneuropathy phenotype and include orthotopic liver transplant (OLT) or pharmacotherapy with Vyndaqel® (tafamidis), Tegsedi™ (inotersen), Onpattro® (patisiran), Amvuttra® (vutrisiran) or off-label use of diflunisal (Adams *et al.* 2016; 2018; 2018; Benson *et al.* 2018; Berk *et al.* 2013; Coelho *et al.* 2012).

Because most of the amyloidogenic-mutated TTR is secreted by the liver, OLT results in rapid disappearance of mutant TTR protein from the serum. However, wild-type TTR protein continues to be produced by the donor liver and can deposit in the pre-existing amyloid deposits in the tissues after transplantation, leading to continual disease progression and, in some cases, accelerating heart disease (Liepnieks *et al.* 2010; Liepnieks & Benson 2007; Yazaki *et al.* 2000; 2007). Younger patients with early ATTRv-PN disease, Val30Met (V30M) mutation, and mild symptoms (typically Stage 1) generally experience better outcomes with OLT. Stage 2 patients are often not candidates for OLT due to advanced age, cardiac involvement, or other health reasons (Herlenius *et al.* 2004; Stangou & Hawkins 2004).

Regarding currently approved medical therapies for ATTRv-PN, Onpattro® (patisiran), a double-stranded small interfering RNA (siRNA) product which also works by inhibiting the production of TTR protein, is approved in the United States for the treatment of the polyneuropathy of ATTRv in adults. This drug is given by IV infusion every 3 weeks after patients are premedicated with dexamethasone, acetaminophen, and anti-histaminic agents to reduce the infusion-related adverse reactions. Tegsedi™ (inotersen), an antisense oligonucleotide (ASO) administered subcutaneously (SC) once a week, is authorized in the European Union, the US and Canada for the treatment of Stage 1 or Stage 2 polyneuropathy in adult patients with ATTRv. This agent is given SC every week. Both agents do not have an approved indication for ATTR-CM.

Vyndaqel® (tafamidis), which is a TTR stabilizer that works by preventing dissociation of the tetramer into amyloid-forming monomers, is indicated for the treatment of ATTRv-PN in adult patients with Stage 1 polyneuropathy to delay peripheral neurological impairment, and is currently approved for use in Europe, Japan, and several other countries (polyneuropathy phenotype indication only). The ATTR-ACT trial (Maurer *et al.* 2018) evaluated the effect of administration of tafamidis or placebo in patients with ATTR-CM (~25% hereditary) for 30 months and demonstrated a statistically significant difference in the primary composite endpoint of all-cause death and CV hospitalizations. Based on these data, Vyndaqel® (tafamidis) was approved for the treatment of ATTR-CM by the FDA in May 2019 and is expected to be approved for this indication in other countries soon. Vyndaqel® (tafamidis) is the first agent with an approved indication for the treatment of hereditary or wild-type ATTR-CM.

Diflunisal is a non-steroidal anti-inflammatory drug (NSAID) that also acts as a TTR tetramer stabilizer and is not currently approved for the treatment of ATTR. Off-label use has been reported in patients with Stage 1 and Stage 2 ATTRv-PN (Adams *et al.* 2016); however, the known CV and renal side effects associated with the NSAID drug class may limit the use of this drug in older patients with ATTR-CM or mixed phenotype.

2.2. Therapeutic Rationale

ION-682884 is an antisense drug targeted to human TTR mRNA and its hybridization to the cognate TTR mRNA results in the RNase H1-mediated degradation of the TTR mRNA, thus preventing production of the TTR protein (see Section 2.3). The strategy of treating ATTR patients with ION-682884 is to reduce the amount of mutated and wild-type TTR protein secreted by the liver, a primary organ for ASO distribution after systemic delivery. It should be noted that ASOs are highly charged hydrophilic molecules that do not cross the blood brain barrier (Levin *et al.* 2008) and thus systemic treatment with ION-682884 is not predicted to decrease levels of TTR in the brain.

It is predicted that decreasing the amount of liver-derived TTR protein circulating in the plasma by treatment with ION-682884 will result in a decrease in the formation of TTR amyloid fibril deposits, and thus slow, halt or even reverse disease progression. This strategy is a similar strategy to OLT, with the exception that ION-682884 reduces wild-type protein in addition to the mutated protein. Given that wild-type TTR can continue to deposit as amyloid after liver transplant, this distinction represents a potential therapeutic advantage.

The primary objective of this study is to evaluate the treatment effect of ION-682884 on mortality and morbidity in a population of wild-type and hereditary ATTR-CM patients. Functional endpoints, patient-reported outcomes, disease scores, and clinical surrogate markers will be evaluated as secondary and/or exploratory objectives. The selection of hard clinical endpoints for the primary analyses is consistent with the clinical benefits already presented by Vyndaqel® (tafamidis) in the ATTR-ACT trial.

Clinical trials in patients with ATTRv-PN generally use derivatives of the NIS, such as the NIS Lower Limb (NIS-LL) or the NIS+7, to assess progression of neuropathy, and the Norfolk QOL-DN to assess disease specific changes in the patients' perceived QOL associated with neuropathy. Because this study will enroll patients with ATTRv-CM, who may present a mixed phenotype, LLF, 10-meter walk test and COMPASS-31 will be periodically assessed in all patients with ATTRv-CM enrolled in the study, and Norfolk QOL-DN will be periodically assessed in the overall study population, regardless of the presence of neuropathy.

The clinical benefits seen with Vyndaqel® (tafamidis) in the ATTR-ACT trial (Maurer *et al.* 2018) were clear and suggested that treatments targeting the stabilization or synthesis of the ATTR protein can be successful. However, despite the significant difference in the primary composite endpoint of all-cause mortality and CV hospitalizations, the ATTR-ACT trial demonstrated that in both treatment and control groups the disease continued progressing with consistent worsening of the distance of the 6MWT and KCCQ scores over time.

Consequently, there continues to be an unmet medical need for more effective and convenient treatments for patients with ATTR-CM.

2.3. ION-682884

2.3.1. Mechanism of Action

ION-682884 is the Sponsor compound code for a second-generation ASO drug targeted to TTR, that has been covalently bound to triantennary *N*-acetyl galactosamine (GalNAc), a high-affinity ligand for the hepatocyte-specific asialoglycoprotein receptor (ASGPR), to form an ASO-GalNAc conjugate. This GalNAc-conjugate approach results in enhanced ASO delivery to hepatocytes vs. non-parenchymal cells and has increased ASO potency at least 10-fold in mice (Prakash *et al.* 2014). The ASO portion of ION-682884 shares the same base sequence as inotersen and is complementary to a region within the 3′ untranslated region (3′ UTR) of the TTR pre-mRNA and binds to the mRNA by Watson and Crick base pairing. Thus, it is expected that once the GalNAc moiety is cleaved from the molecule, the unconjugated ION-682884 behaves the same way as inotersen in its mechanism of action. ION-682884 is also known by the nonproprietary name *eplontersen* (USAN, rINN). For more details regarding ION-682884/eplontersen, please consult the Investigator Brochure (IB).

2.3.2. Chemistry

Chemically, ION-682884 is a synthetic oligomer of 20 nucleotides (i.e., a 20-mer) that are connected sequentially by phosphorothioate and phosphate diester internucleotide linkages, with a triantennary cluster of GalNAc sugars linked to the 5′ end of the oligonucleotide via a phosphodiester/phosphate linkage (Figure 1).

Figure 1: Design of ION-682884, a GalNAc-Conjugated Chimeric 2′-MOE Phosphorothioate/Phosphate Oligonucleotide (MOE Gapmer)

	2′-MOE	2-Deoxy (RNase H1 sensitive)	2′-MOE
GalNAc	${}_{o}{}^{Me}U_{s}{}^{Me}C_{o}{}^{Me}U_{o}{}^{Me}U_{o}G_{o}$	$G_{s}T_{s}T_{s}A_{s}{}^{Me}C_{s}A_{s}T_{s}G_{s}A_{s}A_{s}$	$A_{o}{}^{Me}U_{o}{}^{Me}C_{s}{}^{Me}C_{s}{}^{Me}C$

GalNAc = trishexylamino GalNAc cluster
2′-MOE = 2′-methoxyethyl RNA
2′-Deoxy = DNA
${}^{Me}C$ = 5-methylcytidine
${}^{Me}U$ = 5-methyluridine
s = phosphorothioate linkage
o = phosphate linkage

2.3.3. Nonclinical Information

Detailed information concerning the nonclinical studies conducted with eplontersen (ION-682884) can be found in the Eplontersen Investigator's Brochure, Revision 5, dated 26 July 2024. A summary is included below.

2.3.3.1. Nonclinical Pharmacology

The activity of eplontersen was evaluated in human hepatocytes, human *TTR* transgenic mice, and monkeys as it exhibits full complementarity to the human and monkey *TTR* mRNA sequence. Reductions in hepatic *TTR* expression were demonstrated in all 3 nonclinical models with eplontersen treatment, which resulted in corresponding reductions in plasma TTR protein in transgenic mice and monkeys. When compared to inotersen (ISIS 420915), an unconjugated 2′-MOE ASO of the same sequence, eplontersen exhibited superior potency in human hepatocytes, human *TTR* transgenic mice, and monkeys due to GalNAc conjugation.

2.3.3.2. Pharmacokinetics and Product Metabolism in Animals

The toxicokinetics (TK) and exposure measures confirmed continuous and generally dose-dependent exposure to eplontersen evaluated up to 3 months in the sub-chronic mouse and monkey toxicity studies, and up to 6 months and 9 months in the chronic mouse and monkey toxicity studies, respectively. After subcutaneous (SC) administration, eplontersen was cleared within hours from plasma due to distribution to tissues. Eplontersen was highly bound to plasma proteins (> 96%) in mouse, monkey, and human plasma, which limits glomerular filtration and urinary excretion. The liver half-life of approximately 2 to 4 weeks for eplontersen observed in monkeys supports the monthly clinical dosing regimen. Pharmacokinetics observed in monkeys for eplontersen generally predicted the observed plasma (and expected tissue) exposure levels in humans on the basis of mg/kg equivalent doses.

2.3.3.3. Toxicology and Safety Pharmacology

The toxicity of eplontersen was evaluated up to 6 months and 9 months of dosing in repeat-dose toxicology studies in mice and monkeys, respectively. ION-1184986 (a mouse specific TTR inhibitor) was also included in the 26-week study in mice to evaluate any potential toxicity associated with TTR inhibition. ION-1184986 has the same phosphorothioate/phosphodiester (PS/PO) backbone modifications and is conjugated to the same triantennary GalNAc moiety as eplontersen. Additionally, the preclinical toxicology program for eplontersen included a single-dose safety pharmacology study in the monkey (cardiovascular [CV], respiratory, and central nervous systems [CNS] assessment), *in vitro* hERG assay, an *in vitro* bacterial mutation and chromosomal aberration assays, an *in vivo* mouse micronucleus study, a carcinogenicity study in Tg.rasH2 mice (6 months), a combined fertility and embryo-fetal development study in mice, a dose range-finding study in SD rats and an impurity qualification study in mice.

Most of the findings observed in mice and monkeys following eplontersen treatment were, in general, non-specific class effects related to the uptake and accumulation of 2′-MOE ASO (Henry *et al.* 2008). The intended pharmacologic effect of dose-dependent reduction in hepatic TTR mRNA levels (up to ~62% and 82% reductions) was achieved in monkeys and mice, respectively, with subsequent decreases in TTR plasma protein levels (up to 70% reduction) in (Henry *et al.* 2017) monkeys. There were no toxicologically relevant findings considered related to the pharmacologic inhibition of TTR expression.

In the 13-week monkey study, the only eplontersen-related adverse change consisted of a severely decreased platelet (PLT) count (thrombocytopenia: PLT < 25 × 10^9/L) in 1 female monkey (1 out of 12 animals) administered 24 mg/kg/wk, the highest dose tested. However, no observations of bleeding or hemorrhage were noted in this animal. A moderate decrease in PLT

count (202 × 10^9/L: ~50% decrease from Baseline) was first noted on Day 57 in this monkey and further decreased by Days 73 and 84 to 26 × 10^9/L and 3 × 10^9/L, respectively. The PLT count recovered following steroid administration and dosing holiday. PLT reductions were not observed in any other animals in the 24 mg/kg/wk dose group or in any of the remaining dose groups for 13 weeks. This type of marked platelet change has occasionally been observed in monkeys treated with conjugated or unconjugated ASOs of similar chemistry, where a small proportion of individual animals appear to be susceptible to marked effects on platelets following ASO treatment at higher doses (Henry *et al.* 2017). The plasma AUC at 24 mg/kg/wk (96 mg/kg/month) in monkeys was > 100-fold higher than that for the clinical dose at 45 mg/month eplontersen.

Most of the findings observed in the chronic studies were similar to those observed in the 13-week studies and were related to the uptake and accumulation of ASO in tissues which were not considered adverse. Following a 13-week Recovery Period, no eplontersen-related findings were noted. Furthermore, there were no platelet reductions observed- in the 9-month monkey study at doses up to 25 mg/kg/month (~123-fold higher plasma AUC than the clinical dose at 45 mg/month [approximately 0.64 mg/kg/month assuming a 70-kg patient]).

Eplontersen caused no untoward effects in safety pharmacology studies (*in vivo* and *in vitro*) and was not genotoxic (*in vitro* and *in vivo*). Eplontersen had no effects on fertility or embryo-fetal development in the mouse.

Eplontersen, at doses up to 1500 mg/kg/month, did not produce any evidence of a carcinogenic effect in the Tg.rasH2 mouse model system.

The safety margins between the NOAEL in monkeys and the clinically relevant exposure are based on cumulative plasma AUC measurements. The NOAELs at 6 mg/kg/wk (equivalent to 24 mg/kg/month; 13-week monkey study) and 25 mg/kg/month (9-month monkey study) are ~73 and ~123-fold higher, respectively, than the monthly clinical dose at 45 mg (0.64 mg/kg/month) in humans. The data from the 9-month monkey study supports the Phase 3 clinical evaluation of eplontersen in ATTR patients.

2.3.4. Clinical Information

Detailed information concerning the clinical studies conducted with eplontersen can be found in the Eplontersen Investigator's Brochure, Revision 5, dated 26 July 2024. A summary is included below.

Eplontersen was evaluated in a Phase 1, double-blind, placebo-controlled clinical study (ION-682884-CS1) in healthy volunteers to assess the tolerability, safety, PK, and pharmacodynamic (PD) of single and multiple doses of eplontersen. Based on the results of this study, the clinical dosing regimen selected for Phase 3 studies is 45 mg (as eplontersen free acid, equivalent to ~47 mg eplontersen sodium) administered once every 4 weeks, which is approximately 25-fold lower than the inotersen dosing regimen of 300 mg (as inotersen sodium, equivalent to 284 mg inotersen free acid) administered once weekly, with equal or greater PD effects observed. An additional Phase 1, double-blind, placebo-controlled single ascending dose ethnobridging study (ION-682884-CS20) was conducted to assess safety, PK, and PD in healthy volunteers of Japanese descent.

In a Phase 1, single-dose, randomized, open label, 3-period crossover study (Study ION-682884-CS21) in healthy adult subjects, the bio-equivalence (BE) of 3 drug presentations used to administer 45 mg of eplontersen (vial and syringe, prefilled syringe with a safety device, and prefilled syringe with autoinjector) was evaluated. This study was conducted to support bridging from the single-dose vial and syringe presentation to the use of the prefilled syringe with a safety device or the prefilled syringe with an autoinjector.

One (1) pivotal Phase 3 study of eplontersen is ongoing: a randomized, double-blind, placebo-controlled study in hereditary and wild-type TTR-mediated amyloid cardiomyopathy (ATTR-CM) patients (ION-682884-CS2, also known as CARDIO-TTRansform). One (1) pivotal Phase-3-study has been completed: a randomized, open-label, historically-controlled study in hereditary TTR-mediated amyloid polyneuropathy (ATTRv-PN) patients (ION-682884-CS3, also known as NEURO-TTRansform). Participants who successfully complete the treatment period in these index studies are eligible to enter the corresponding ongoing Phase 3 treatment extension study, ION-682884-CS12 for ATTR-CM and ION-682884-CS13 for ATTRv-PN.

The primary objective of Study ION-682884-CS3 was to evaluate the effect of eplontersen with regards to mNIS+7, Norfolk QoL-DN, and the serum TTR protein concentration over a 65-week external placebo-controlled period. The objective was achieved by performing placebo-controlled evaluations at 2 time points, at an interim analysis when all patients reached Week 35, and at a final analysis when all patients reached Week 65/66. The eplontersen-treatment period for patients in ION-682884-CS3 continued to Week 85 of the study.

2.3.4.1. Clinical Pharmacology

In the Phase 1 multiple and single ascending dose study ION-682884-CS1, eplontersen was absorbed rapidly into the systemic circulation with median time to maximum plasma concentrations (T_{max}) ranging from 1 to 6 hours following SC administration. Mean peak (C_{max}) and total exposure (AUC) were dose-dependent and slightly higher than dose-proportional over the studied SC dose range. After reaching C_{max}, mean plasma concentrations of eplontersen declined in a bi-phasic fashion with time, with an initial rapid distribution phase that dominated the plasma clearance, followed by a slower elimination phase with terminal half-life of 2 to 4 weeks. This result is consistent with the slow elimination of eplontersen observed in monkeys, with terminal elimination half-life of approximately 2 to 4 weeks determined in monkey plasma and tissues, and consistent with the comparatively long half-lives observed for this chemical class.

There was little or no increase in mean plasma C_{max} or AUC after 4 repeated monthly SC injections as compared to the first dose, consistent with the expected result of little or no plasma accumulation and time-invariant plasma kinetics. In the Phase 1 single ascending dose study in healthy volunteers of Japanese descent, ION-682884-CS20, the C_{max} and AUC were 43% and 24% higher, respectively, as compared to heathy volunteers of non-Japanese descent (ION-682884-CS1) when comparing the plasma exposure following the same site of injection (i.e., abdomen). These C_{max} and AUC differences are consistent with the modest body weight (BW) effects on PK, where higher exposures (AUC, C_{max}) were seen with lower BW (both as BW and lean body mass [LBM]).

In patients with ATTRv-PN, eplontersen was rapidly absorbed following SC administration, with a median (range) T_{max} of approximately 2 hours (range 1 to 6 hours). No increase in C_{max} or AUC from time 0 to 6 hours post-dose (AUC_{0-6h}) was observed, as compared to Day 1, on Days 225 and 449, after repeated Q4W administrations, which is consistent with the expected little or no plasma accumulation and time-invariant plasma kinetics. However, accumulation in trough concentration (C_{trough}) was observed with Q4W SC administration. Time to steady state, based on C_{trough} in patients who tested negative for anti-drug antibody (ADA), appeared to be reached by Day 169. Of note, immunogenicity had no impact on PK parameters except plasma C_{trough}.

The intended commercial drug product presentation of PFS with AI is supported by the BE study (Study ION-682884-CS21), together with the population PK, PK/PD, and exposure-response (E-R) analyses.

2.3.4.2. Clinical Pharmacodynamics

In the Phase 1 multiple and single ascending dose study, ION-682884-CS1, and the Phase 1 single ascending dose ethnobridging study, ION-682884-CS20, subcutaneous administration of eplontersen resulted in statistically significant, dose-dependent reductions in serum TTR protein and retinol binding protein 4 (RBP4) compared to placebo. The results for placebo-treated subjects showed little variation over time.

In study ION-682884-CS1, the percent change from Baseline in serum TTR protein at Day 29 after a single dose of eplontersen was -54.3 with 45 mg, -78.8 with 60 mg, -81.5 with 90 mg and -86.3 with 120 mg. The corresponding percent change from Baseline in RBP4 for the 4 cohorts were -52.8, -65.0, -71.7 and -77.4, respectively. In study ION-682884-CS20, the percent change from Baseline in serum TTR protein at Day 29 were similar to those seen in subjects of non-Japanese descent at -59.6 with 45 mg, -65.5 with 60 mg, and -77.2 with 90 mg. The corresponding percent change from Baseline in RBP4 for the 3 cohorts were -53.6, -68.1, and -69.2, respectively.

In the multiple-dose cohorts (ION-682884-CS1), the percent change from Baseline in serum TTR protein was -85.7, -90.5 and -93.8 for the 45, 60 and 90 mg multiple-dose cohorts, respectively. The corresponding percent change from Baseline in RBP4 for the 3 cohorts were -77.3, -79.2 and -84.0, respectively.

In study ION-682884-CS3, at the Week 35 interim analysis time point, mean percent change from Baseline in serum TTR protein was -81.2 (1.7) in the eplontersen group and -14.8 (2.0) in the external placebo group. The least square means (LSM) difference for percent change from Baseline at Week 35 between eplontersen and external placebo was statistically significant: 66.4 (95 % confidence interval [CI] -71.4, -61.5; $p < 0.0001$). At the Week 66 final analysis, the reduction in serum TTR was maintained. The LSM (SE) percent changes from Baseline were -81.7 (1.6) and -11.2 (1.9), respectively, and the difference in LSM was -70.4 (95% CI -84.8 to -78.5, $p < 0.0001$). Mean percent change from Baseline in the eplontersen group was maintained through Week 85.

2.3.4.3. Clinical Efficacy

The efficacy assessments in the ION-682884-CS3 study were percent change from Baseline in serum TTR concentration, change from Baseline in mNIS+7, a composite score of polyneuropathy impairment and change from Baseline in Norfolk QoL-DN, a patient-reported assessment that evaluates the subjective experience of neuropathy (quality of life). All 3 assessments were evaluated at 2 time points comparing to the external placebo, at an interim analysis when all patients reached Week 35, and at a final efficacy evaluation when all patients reached Week 65/66. These assessments were also to be performed at Week 85 (without comparison to the external placebo).

TTR

Eplontersen was superior to the external placebo in the co-primary endpoint of percent change from Baseline in serum TTR concentration.

mNIS+7

Eplontersen was superior to the external placebo in the co-primary endpoint mNIS+7 change from Baseline at Week 35. The LSMs for mNIS+7 change from Baseline were 0.2 (1.9) in the eplontersen group and 9.2 (1.9) in the external placebo group. The difference in the LSM in change from Baseline between eplontersen 45 mg Q4W and placebo was 9.0 (95 % CI -13.5 to -4.5; $p < 0.0001$). At Week 66, the LSM (SE) of mNIS+7 change from Baseline in the eplontersen-treated patients remained minimal at 0.3 (2.4) whereas the LSM (SE) of mNIS+7 in placebo-treated patients worsened to 25.1 (2.4). The difference in LSM between eplontersen and placebo groups at Week 66 was -24.8 (95% CI -31.0 to -18.6, $p < 0.0001$). The benefit of eplontersen treatment on mNIS+7 was maintained through to Week 85 where mean (SE) change was -2.9 (1.9).

Norfolk QoL-DN

Eplontersen was superior to the external placebo in quality of life as assessed by the Norfolk QoL-DN total score. The LSM for Norfolk QoL-DN change from Baseline in the ION-682884-CS3 eplontersen group was -3.1 (2.1) and in the external placebo group was 8.7 (2.1), indicating, on average, no worsening of quality of life in patients receiving eplontersen, and worsening of quality of life in patients receiving placebo. The difference in the LSM for change from Baseline to Week 35 in Norfolk QoL-DN between eplontersen 45 mg Q4W and placebo was -11.8 (95 % CI -16.8 to -6.8; $p < 0.0001$). At Week 66, the LSM (SE) Norfolk QoL-DN total score of the eplontersen-treated patients improved to -5.5 (2.3) and worsened to14.2 (2.3) for the placebo-treated patients. The benefit of eplontersen treatment on Norfolk QoL-DN was maintained through to Week 85 where mean (SE) change was -6.2 (1.7).

2.3.4.4. Clinical Safety

Eplontersen safety profile was summarized/characterized from the completed Phase 1 multiple and single dose escalation studies in healthy volunteers, ION-682884-CS1 and ION-682884-CS20, the completed Phase 1 BE study in healthy volunteers, ION-682884-CS21, the completed Phase 3 pivotal study ION-682884-CS3 (NEURO-TTRansform),the ongoing Phase 3 pivotal study ION-682884-CS2 (CARDIO-TTRansform), and the 2 ongoing Phase 3 OLE studies, ION-682884-CS13 for ATTRv-PN and ION-682884-CS12 for ATTR-CM.

As of 13 May 2024, an estimated total of 1,088 unique subjects have been exposed to at least 1 dose of the Study Drug/treatment in clinical studies. For further details on exposure, please refer to Section 5.4.1 Extent of Exposure of the current IB.

The clinical safety of eplontersen was initially evaluated in the completed Phase 1 healthy volunteer studies ION-682884-CS1 and ION-682884-CS20. Eplontersen was well tolerated in both of these studies. There were no serious adverse events (SAEs) nor deaths or events that led to discontinuation in these studies. Laboratory, vital signs, and ECG data showed no clinically relevant changes except that reversible ALT increases (> 3 × ULN) were observed in 2 subjects in the ION-682884-CS1 90-mg multiple dose cohort without increases in total bilirubin or any clinical symptoms.

Clinical safety was also evaluated in ION-682884-CS21, a completed Phase 1 BE study of a 45 mg dose of eplontersen from 3 SC presentations in healthy volunteers. In the completed study, eplontersen was well tolerated. Safety results were comparable among the 3 treatment drug product presentations and consistent with the previous studies of eplontersen in healthy volunteers (Studies ION-682884-CS1 and ION-682884-CS20), with no new signal detected. No deaths nor serious treatment-emergent adverse events (TEAEs) were reported. The injection site reactions were comparable between treatment drug product presentations: 1 patient had TEAEs of injection site pain (pre-filled syringe [PFS] with autoinjector [AI]; probably related to eplontersen and not related to study device) and injection site paraesthesia (PFS with safety device; unlikely related to eplontersen and related to study device).

Clinical safety was also evaluated in ION-682884-CS3, a completed Phase 3, open-label study in which eplontersen was administered to 168 ATTRv-PN patients, including 137 patients exposed to at least 12 months of eplontersen treatment.

No clinically significant, grade 3/4 or serious thrombocytopenia events were observed in the study. None of the events lead to any Study Drug interruption and all recovered without any treatment given. Additionally, no concurrent bleeding-related adverse events (AEs) were reported. The percentage of subjects in the eplontersen group who experienced platelet counts $< 100 \times 10^9$/L at any time post-Baseline was low (2.8%) and similar to that in the external placebo group (3.3%). The mean platelet counts were generally stable over time in the eplontersen group and similar to the external placebo group.

There were no clinically meaningful imbalances, or any observable trends seen in renal and hepatic OAEIs with eplontersen.

In the end-of-treatment analysis of the study, the cumulative data as of 07 April 2023, demonstrated that eplontersen 45 mg SC, q4wk, had an acceptable safety and tolerability profile compared with external placebo and historical inotersen in an ATTRv-PN population.

As of 13 May 2024, 151 ATTRv-PN patients (146 rolled-over from the CS3 study and 5 enrolled from an investigator-sponsored trial of inotersen) received eplontersen in the open-label extension (OLE) study ION-682884-CS13. Vomiting, injection site erythema, injection site pain, injection site pruritus, and vitamin A decreased have been identified as adverse drug reactions. For further details on the safety of subjects under the CS3 study please refer to the current IB.

As of 13 May 2024, 1432 ATTR-CM patients have been randomized in the ongoing, blinded ION-682884-CS2 study and 187 patients have been enrolled in the OLE study ION-682884-CS12.

As Study ION-682884-CS2 is ongoing and remains blinded, the safety evaluation of eplontersen is limited. For further details on safety of subjects under CS2 study, please refer to current version of the IB.

There are no identified risks considered important for eplontersen. The most frequent adverse reactions during treatment with eplontersen observed in ≥ 5% of patients were vomiting and vitamin A decrease. Ocular AEs due to vitamin A deficiency have been identified as an important potential risk for eplontersen. For further details, please refer to Section 6 of the current IB.

In Studies ION-682884-CS1 and ION-682884-CS3, 5 of 39 (12.8%) healthy subjects and 58 of 144 (40.3%) patients with ATTRv-PN developed treatment-emergent ADA with Q4W administration of eplontersen for up to 13 and 85 weeks, respectively. Of note, by the data cutoff date of 07 April 2023, the mean treatment duration was 540.8 days in Study ION-682884-CS3. ADA had no impact on the peak (C_{max}) and total (AUC) plasma exposures, albeit a higher C_{trough} was observed in ADA-positive subjects. There was no clinically meaningful impact of ADA positivity on PD, efficacy, or safety.

Eplontersen was approved in the US on 21 December 2023 for the treatment of polyneuropathy of hereditary transthyretin-mediated amyloidosis (ATTRv-PN) in adults.

Overall, the safety profile of eplontersen has been acceptable and manageable. The benefit/risk profile of eplontersen remains favorable and supports continued development (CM indication) and commercialization (PN indication) of this drug.

2.4. Rationale for Dose and Schedule of Administration

Due to the predictability of the PK of ASOs and the cumulative experience with other drugs in this class, estimation of clinical doses for an antisense drug is less difficult than for most drugs in preclinical development. The PK and disposition of many drugs in this class have been characterized and there are remarkable similarities from sequence to sequence and across species. In particular, the plasma PK for ASOs in monkeys has been highly predictive of those in man. Because distribution to tissues is the dominant mechanism for plasma clearance, it can be assumed that plasma PK similarities translate into similarities in tissue distribution. This assumption is supported by the knowledge that patterns of tissue distribution for oligonucleotides are similar in mice, rats, rabbits, dogs and monkeys. Thus, it is possible to predict dose regimens on the basis of concentrations at the primary site of activity, the liver. Additionally, ASOs are hydrophilic and distribute predominantly to the lean body mass and in our experience, they should be administered as a fixed dose rather than by total body weight.

In ATTRv-PN, both wild-type and mutant proteins will deposit in the tissue as amyloid. TTR mutant reductions ≥ 90% have been observed post-liver transplantation (Adams *et al.* 2000) and have been shown to have a therapeutic effect. Additionally, 2 Phase 3 clinical trials have shown that median plasma concentration TTR reduction from Baseline of 79% (Benson *et al.* 2018) and 81% (Adams *et al.* 2018) is associated with clinical benefit, measured by clinical endpoints (mNIS+7 and Norfolk-QOL-DN). Therefore, the therapeutic goal of

ION-682884 is to reduce both wild-type and mutant plasma TTR concentration as much as possible using a dose level that will not cause unacceptable toxicity.

In the ION-682884-CS1 study, the 45 mg once-monthly dose level showed satisfactory safety profile and a substantial PD effect (85.7% mean reduction in plasma TTR concentration).

The prolonged half-life of ION-682884 in liver of approximately 4 weeks observed in monkeys supports the once every 4 weeks dosing regimens used in ION-682884-CS1 and planned in this study. Moreover, this dosing schedule has been employed safely in previous clinical studies with a number of other conjugated, 2′-MOE ASOs. Kidney, liver, and adipose tissue concentrations of ION-682884 collected after 13 weeks of dosing during a monkey toxicology study indicate that tissue distribution for this compound is consistent with previously administered ASOs. Post-distribution plasma concentrations (i.e., terminal elimination phase) of ION-682884 are expected to be in equilibrium with tissue concentrations in humans, as was observed in monkeys, mice, and rats for similar 2′-MOE gapmers with and without GalNAc-conjugation (Geary *et al.* 2003; Yu *et al.* 2016; 2016).

Safety data from the toxicology studies support a once every 4 weeks dose strategy. In the toxicology studies with ION-682884 in mice and monkeys, no overt toxicities were observed after both subchronic and chronic studies (Study Nos. 682884-AS01, -AS02, -AS04, and -AS05; for details, refer to the ION-682884 IB).

The No Adverse Effect Level (NOAEL) for ION-682884 was determined to be 6 mg/kg/wk (24 mg/kg/month) in a weekly dosing study with a duration of 13 weeks in monkeys; and 25 mg/kg/month in a monthly dosing study with a duration of 39 weeks, also in monkeys. Pharmacokinetics observed in monkeys for ASOs predict the expected exposures in humans on the basis of mg/kg-equivalent doses (FDA 2005; Geary *et al.* 2003; Yu *et al.* 2007) and not on the basis of surface area. This PK behavior is class-related and has been shown for all ASOs examined to date in the same chemical class as ION-682884. Therefore, the dose of ION-682884 chosen for this study, 45 mg/month (leading to an actual plasma $AUC_{0\text{-}48h}$ on Day 85 of 1.4 µg.hr/mL) corresponds to 94- and 142-fold below the NOAEL based on the 13-weeks study and the 39-weeks study, respectively; this dose is considered a safe dose for this trial.

In addition to the clinical and nonclinical experience of ION-682884, the safety of this dose level is supported by the preclinical and clinical experience of several other 2′-MOE-modified ASOs that have been safely administered IV and SC in multiple clinical studies at doses up to 1000 mg (Kwoh 2008) and for treatment durations that exceed 24 months.

2.5. Benefit-Risk Assessment

2.5.1. Benefit Assessment

Based on all data to date, including the Week 35, Week 66, Week 85, and end-of-study analyses of the Phase 3 study ION-682884-CS3, eplontersen 45 mg given Q4W is well-tolerated and has an acceptable safety profile in healthy volunteers and adult patients with ATTRv-PN.

The Week 65/66 final placebo-controlled analysis of Study ION-682884-CS3 (Neuro-TTRansform) demonstrated that eplontersen 45 mg Q4W given to adults with ATTRv-PN was superior to placebo for the (1) reduction of serum TTR, (2) stabilization of

neuropathy impairment (mNIS+7), and (3) improvement of patient-reported quality of life (Norfolk QoL-DN).

Notably, analyses suggest that eplontersen may reverse neuropathy impairment (mNIS+7) and/or improve quality of life (Norfolk QoL-DN) for many patients. At Week 66, 53.1 % of subjects treated with eplontersen displayed improvement in signs of neuropathy (mNIS+7), and 64.8% displayed improvement in patient-reported quality of life (Norfolk QoL-DN).

Administration of ION-682884 to patients may reduce the levels of mutated and wild-type TTR protein secreted by the liver, the primary source of TTR production and a primary organ for GalNAc-conjugated ASO distribution after systemic delivery. By decreasing the amount of liver-derived TTR protein circulating in the plasma, ION-682884 treatment is expected to decrease CV mortality and morbidity.

2.5.2. Risk Assessment

There are no important identified risks for eplontersen.

Key potential risks were identified prior to completion of the Phase 3 study ION-682884-CS3 based on the known safety profile of inotersen, an earlier generation 2′-MOE ASO. Severe reductions in platelet count $< 50 \times 10^9$/L accompanied by a MB or CRNMB, or platelet count of $< 25 \times 10^9$/L independent of a MB or CRNMB event are considered as AEs of special interest. Monitoring rules and stopping rules for platelet count reduction, bleeding, ocular, renal, and liver events remain in place and will continue to be enforced.

The safety data described below reflects exposure to eplontersen in 151 patients with polyneuropathy caused by ATTRv (ATTRv-PN) randomized to eplontersen and who received at least one dose of eplontersen. Of these, 141 patients received at least 6 months of treatment, and 137 patients received at least 12 months of treatment. The mean duration of treatment was 541 days (range: 57 to 582 days).

Per the current IB revision 5.0, the most frequent adverse reactions during treatment with eplontersen observed in ≥ 5% of patients were vomiting and vitamin A decreased. Table 1 presents a summary of ADRs (that include vomiting, injection site erythema, injection site pain and injection site pruritus) per frequency category (see below). Ocular events due to vitamin A deficiency are important potential risks for eplontersen.

Table 1: Summary of Adverse Reactions per Frequency Categories

System Organ Class	Adverse Reaction	Frequency*
**Gastrointestinal disorders**	Vomiting	Common
**General disorders and administration site conditions**	Injection site erythema	Common
	Injection site pain	Common
	Injection site pruritus	Common
**Investigations**	Vitamin A decreased	Very Common

* Frequencies of occurrence of adverse reactions are defined as: very common (≥ 1/10); common (≥ 1/100 to < 1/10); uncommon (≥ 1/1,000 to < 1/100); rare (≥ 1/10,000 to < 1/1000); very rare (< 1/10,000) and not known (cannot be estimated from available data)

Eplontersen is an ASO drug targeted to human TTR mRNA. Hybridization to the cognate TTR mRNA results in the ribonuclease H1-mediated degradation of the mRNA, inhibiting production of the TTR protein. A known function of TTR in the plasma is to transport retinol (vitamin A) to tissues through its association with RBP4. The retinol-RBP4 complex in turn binds to TTR, which prevents the complex from being excreted through the kidney. Other mechanisms also exist for vitamin A transport to tissues. A decrease in serum concentration of vitamin A is therefore an expected consequence of the mechanism of action of eplontersen.

Based on results from the ION-682884-CS3 study up to Week 66 (end of placebo-controlled treatment period), mean serum vitamin A concentrations at Baseline were similar in the eplontersen, external placebo, and historical inotersen groups (52.2, 53.1, and 50.0 µg/dL, respectively). Mean vitamin A values decreased in the eplontersen 45 mg q4wk group and remained lower throughout the study compared with the external placebo group. As expected, a higher incidence of vitamin A values below the lower limit of normal at any time post-baseline was observed in the eplontersen treated population (96.5%) compared with the external placebo group (3.3%). No patients in any treatment group had post-baseline vitamin A value > ULN. Treatment with eplontersen in combination with vitamin A supplementation does not appear to increase the risk of symptomatic vitamin A deficiency.

The incidence of subjects with ocular AEs potentially related to vitamin A deficiency was 27.1% in the eplontersen treated population compared with 15% in the external placebo group and 18.8% in the historical inotersen group. Excluding the TEAEs with PT of vitamin A deficiency and PT vitamin A decreased, the incidence of ocular AEs potentially related to vitamin A deficiency AESI was similar between the eplontersen group (16.7%) and the external placebo group (15.0%). Collectively, review of ocular AEs continues to show that cases were generally mild in severity and does not suggest a relationship to eplontersen.

Clinical consequences of vitamin A deficiency can include ocular AEs such as reduced night vision or night blindness and persistent dry eyes.

In this study all patients will be required to take oral supplementation of the recommended daily allowance (RDA) of vitamin A (approximately 3000 IU vitamin A per day). In addition, patients will complete periodically an “ocular questionnaire” about signs and symptoms of vitamin A deficiency.

2.5.3. Overall Assessment of Benefit:Risk

Taking into account the potential benefits in CV mortality and morbidity provided by the treatment with ION-682884 and the measures taken to minimize the potential risks to patients participating in this study, including regular safety monitoring, supplementation with vitamin A, and allowing the concomitant administration of approved therapies, the enrollment of patients with hereditary or wild-type ATTR-CM into this study is justified.

3. EXPERIMENTAL PLAN

3.1. Study Design

This is a Phase 3 global, double-blind, randomized, placebo-controlled, study to evaluate the efficacy and safety of ION-682884 (eplontersen) in patients with hereditary or wild-type ATTR-CM.

Approximately 1400 patients will be randomized 1:1 to receive 45 mg of ION-682884 or placebo SC once every 4 weeks. The Treatment Period is up to 140 weeks. Dose frequency adjustments, including dose interruptions, are allowed for reasons of safety or tolerability, per discussion with Medical Monitor.

Patients will be stratified at Randomization based on NYHA Classification (I and II vs. III), ATTR mutation status (mutated vs. wild-type), 6MWT distance (6MWT ≤ 350 meters vs. > 350 meters), and current treatment with tafamidis (Yes vs. No).

Patients must meet all eligibility criteria to be enrolled in this trial. Concomitant administration of SoC therapies is allowed, per Investigator's discretion, if locally approved (including compassionate access, extended access, and other), accessible, and consistent with local clinical practice (except ASO and siRNA compounds). Patients will also take daily supplemental doses of the RDA of vitamin A.

3.2. Number of Study Centers

This study will be conducted at multiple centers worldwide.

3.3. Number of Patients

Approximately 1400 patients will be randomized 1:1 to receive 45 mg of ION-682884 or placebo SC once every 4 weeks for a total of up to 140 weeks.

3.4. Overall Study Duration and Follow-up

This study will consist of the following periods:

- Up to 10 weeks for Screening and Baseline Assessment Period
- Up to 140-week Treatment Period
- A 20-week Post-Treatment Evaluation Period

Following the Treatment Period, eligible patients may elect to enroll in an open-label extension (OLE) study pending study approval by the Institutional Review Board/Independent Ethics Committee (IRB/IEC) and the appropriate regulatory authorities. All participating patients in the OLE study will receive ION-682884 once every month. In this case, patients possibly will not participate in some or all of the Post-Treatment Evaluation Period assessments.

3.4.1. Screening

Patient eligibility for the study will be determined within 10 weeks prior to study entry. Sites can perform part or all of the consenting process remotely, if locally acceptable.

3.4.2. Treatment Period

The Treatment Period is up to 140 weeks (from Day 1/Randomization to end of Week 140). Enrolled patients will visit the clinical research site at regular intervals through the 140-week Treatment Period. During this period ION-682884 or placebo will be administered SC every 4 weeks.

3.4.3. Post-Treatment Evaluation Period

The Post-Treatment Evaluation Period comprises 20 weeks that follow the Treatment Period (Week 140 to Week 160). Patients will visit the clinical research site at regular intervals through the 20-week Post-Treatment Evaluation Period.

Alternatively, after completion of the Treatment Period, eligible patients may elect to enroll in an OLE study where they will receive ION-682884 SC every month, pending study approval by the IRB/IEC and appropriate regulatory authority. In this case, patients may not participate in any or only some of the Post-Treatment Evaluation Period.

3.5. End-of-Study

The Primary Completion Date, defined as "the date on which the last participant in a clinical study was examined or received an intervention/treatment to collect final data for the primary outcome measures (primary endpoint assessments)" is the last patient's last visit of the Treatment Period.

The vital status (i.e., dead including date of death; alive including last date known alive; unknown) up to the Primary Completion Date of all study participants exposed to Study Drug will be determined where possible and regardless of the patient's End-of-Study status except those participants active in this study or in the OLE at the time of determination and except those participants already with known date of death.

The End-of-Study is the last patient last visit.

3.6. Data and Safety Monitoring Board

An independent Data and Safety Monitoring Board (DSMB) will be assembled to review safety, tolerability and efficacy (as needed) of unblinded data collected during the trial. Details on the safety assessments, frequency of review, meeting schedules and controlled access to unblinded data are outlined in the DSMB Charter and Statistical Analysis Plan (SAP).

4. PATIENT ENROLLMENT

4.1. Screening

Before patients may be enrolled into the study, the Sponsor requires a copy of the Study Center's written independent IEC/IRB approval of the protocol, informed consent form, and all other patient-facing information and/or recruitment material.

Patients must sign the consent form before any screening tests or assessments, including fasting prior to screening blood draws, are performed. At the time of consent, the patient will be

considered enrolled in the study and will be assigned a unique screening number before any study procedures, including Screening procedures, are performed. At the time of randomization, patients will be assigned a unique patient identification number. This number will be used to identify the patient throughout the trial and must be used on all study documentation related to that patient. The patient identification number must remain constant throughout the entire trial. In the event the patient is re-consented and re-screened the patient must be given a new screening number. Screening numbers and patient identification numbers, once assigned, will not be re-used.

4.2. Randomization

Patients will be randomized after all Screening assessments have been completed and after the Investigator has verified that they are eligible per criteria in Sections 5.1 and 5.2. No patient may begin treatment prior to randomization and assignment of a unique patient identification number.

Using an Interactive Voice/Web-Response System (IXRS), eligible patients will be randomized 1:1 to receive ION-682884 or placebo.

To ensure treatment arms will be balanced, patients will be stratified at randomization by:

- NYHA Classification (I and II vs. III)
- ATTR mutation status (mutated vs. wild-type)
- 6MWT distance (6MWT $\leq$ 350 meters vs. > 350 meters)
- Current treatment with tafamidis (Yes vs. No)

4.3. Replacement of Patients

No patients will be replaced in this study.

4.4. Unblinding of Treatment Assignment

The Sponsor and all patients, monitors, and Study Center personnel will be blinded about treatment assignment throughout the study. However, if a patient has suffered a SAE (as defined in Section 9.3.3) and when knowledge of the treatment assignment will impact the clinical management of the patient, the Investigator will have the ability to unblind the treatment assignment for that patient using the automated IXRS. Investigators are encouraged to discuss with Medical Monitor before unblinding, if possible. An unblinded randomization schema will be maintained securely at the Sponsor's designated vendor.

5. PATIENT ELIGIBILITY

To be eligible to participate in this study candidates must meet the following eligibility criteria within 10 weeks of Study Day 1, or at the time point specified in the individual eligibility criterion listed.

The Medical Monitor may be consulted if any questions arise regarding the Inclusion or Exclusion criteria.

5.1. Inclusion Criteria

1. Must have given written informed consent (signed and dated) and any authorizations required by local law and be able to comply with all study requirements
2. 18 to 90 years of age (inclusive)
3. Females: must be non-pregnant and non-lactating and either:
   a. surgically sterile (e.g., tubal occlusion, hysterectomy, bilateral salpingectomy, bilateral oophorectomy)
   b. post-menopausal (defined as 12 months of spontaneous amenorrhea in females > 55 years of age or, in females ≤ 55 years, 12 months of spontaneous amenorrhea without an alternative medical cause and follicle-stimulating hormone (FSH) levels in the post-menopausal range for the laboratory involved)
   c. abstinent* or,
   d. if engaged in sexual relations of childbearing potential, agree to use 1 highly effective contraceptive method from the time of signing the informed consent form until at least 24 weeks after the last dose of Study Drug (ION-682884 or placebo). See Section 6.4.1 for more details.

   Males must be surgically sterile or abstinent*; if engaged in sexual relations with a female of childbearing potential, the patient must be using an acceptable contraceptive method from the time of signing the informed consent form until at least 24 weeks after the last dose of Study Drug

   * Abstinence is only acceptable as true abstinence, i.e., when this is in line with the preferred and usual lifestyle of the patient. Periodic abstinence (e.g., calendar, ovulation, symptothermal, post-ovulation methods), declaration of abstinence for the duration of a trial and withdrawal are not acceptable methods of contraception.
4. Willing to be genetically tested for mutations in the TTR gene during screening, if it was not done before
5. Amyloid deposits in cardiac or non-cardiac tissue confirmed by Congo Red (or equivalent) staining OR technetium scintigraphy (99mTc -3,3-diphosphono-1,2-propanodicarboxylic acid [DPD-Tc], 99m Tc-pyrophosphate [PYP-Tc], or 99mTc-hydroxymethylene-diphosphonate [HMDP-Tc]) with Grade 2 or 3 cardiac uptake in the absence of abnormal light chains ratio, centrally confirmed
6. End-diastolic interventricular septum thickness of > 12 mm on Screening echocardiogram
7. Medical history of HF secondary to hereditary or wild-type ATTR-CM with at least: A) prior hospitalization for HF, which may include hospitalization for arrhythmia or pacemaker/ICD (implantable cardioverter defibrillator) placement, OR B) symptoms and signs of volume overload or elevated intracardiac pressure that requires treatment with diuretics other than mineralocorticoid receptor antagonists (MRA) for clinical stabilization
8. Screening N-terminal prohormone of brain natriuretic peptide (NT-proBNP) ≥ 600 pg/mL (≥ 70 pmol/L) by central lab. For patients in atrial fibrillation at screening the NT-proBNP values ≥ 1200 pg/mL (≥ 140 pmol/L)

9. New York Heart Association (NYHA) class I-III
10. 6MWT ≥ 100 meters
11. If on medical treatment for HF on stable dosage regimen for 2 weeks prior to randomization
12. Willingness to adhere to vitamin A supplementation per protocol

5.2. Exclusion Criteria

1. Acute coronary syndrome, unstable angina, stroke, TIA, coronary revascularization, cardiac device implantation, cardiac valve repair, or major surgery within 3 months of Screening
2. Hospitalization or urgent visit to ER/ED for worsening of HF with discharge date within 4 weeks prior to or during Screening
3. Uncontrolled hypertension (systolic blood pressure [BP] > 160 or diastolic BP > 100 mmHg)
4. Uncontrolled clinically significant cardiac arrhythmia, per Investigator's assessment (e.g., no pacemaker, although indicated)
5. Severe uncorrected cardiac valvular disease
6. Cardiomyopathy not primarily caused by ATTR-CM, for example, cardiomyopathy due to hypertension, valvular heart disease, or ischemic heart disease
7. Screening laboratory results as follows, or any other clinically significant abnormalities in Screening laboratory values that would render a patient unsuitable for inclusion
    a. Alanine aminotransferase/aspartate aminotransferase (ALT/AST) > 2.0 × upper limit of normal (ULN)
    b. Total bilirubin ≥ 2 × ULN (patients with total bilirubin ≥ 2 × ULN may be allowed on study if indirect bilirubin only is elevated, ALT/AST is not greater than the ULN and known to have Gilbert's disease OR if, in the opinion of the Investigator, the bilirubin abnormality is deemed not clinically significant, pending local workup and discussion with Medical Monitor)
    c. Platelets < 125 × 10^9/L
    d. Urine protein creatinine ratio (UPCR) ≥ 750 mg/g. In the event of UPCR above this threshold ineligibility may be confirmed by a repeat random spot UPCR ≥ 750 mg/g
    e. Positive test for blood (including trace) on urinalysis that is subsequently confirmed with urine microscopy showing > 5 red blood cells per high power field and is related to glomerulopathies. In women, this exclusion criterion must be assessed outside of menstrual period. If in the opinion of the Investigator the haematuria is not considered related to glomerulopathies the patient may be considered eligible, pending proper follow-up and a discussion with the Medical Monitor. Patients with history of bladder cancer must have been treated with curative intent and have not presented recurrence within the prior 5 years

f. Estimated glomerular filtration rate (eGFR) < 30 mL/min/1.73 m^2 at Screening [Chronic Kidney Disease Epidemiology Collaboration [CKD-EPI] formula;(Levey *et al.* 2009)]. If the eGFR is thought to be underestimated, the CKD-EPI creatinine-cystatin C equation can be used for confirmation (Inker *et al.* 2012)
g. Abnormal thyroid function tests with clinical significance per Investigator judgement
h. Serum retinol level at Screen < LLN (this criterion does not apply to ATTRv-CM patients with mutation at the position 84 [e.g., Ile84Ser])
i. Hemoglobin A1c (HbA1c) > 9.5%

8. Monoclonal gammopathy of undetermined significance (MGUS) and/or alterations in immunoglobulin free light chain (FLC) ratio, unless fat, bone marrow, or heart biopsy confirming the absence of light chain and the presence of TTR protein by mass spectrometry or immunoelectron microscopy. For patients with CKD and without presence of monoclonal protein in blood and urine, the acceptable FLC ratio is 0.26-2.25. Results different from that may be discussed with local hematologist, Investigator and Medical Monitor if the risks associated with the biopsy outweigh the benefits
9. Active infection requiring systemic antiviral or antimicrobial therapy that will not be completed prior to Study Day 1
10. Known history of or positive test for human immunodeficiency virus (HIV) (as evidenced by positive tests for HIV antibody and HIV RNA), hepatitis C (as evidenced by positive tests for HCV antibody and HCV RNA) or hepatitis B (as evidenced by a positive test for hepatitis B surface antigen)
11. History of bleeding, diathesis or coagulopathy (e.g., liver cirrhosis, hematologic malignancy, antiphospholipid antibody syndrome, congenital disorders such as hemophilia A, B, and Von Willebrand disease)
12. If receiving oral anticoagulants (except vitamin K antagonists), the dose must have been stable for 4 weeks prior to the first dose of Study Drug and regular monitoring must be performed, per clinical practice during the study. If the patient is receiving vitamin K antagonists (e.g., warfarin) INR should be in therapeutic range, as established by the Investigator, for 4 weeks prior to the first dose
13. Malignancy within 5 years, except for basal or squamous cell carcinoma of the skin, melanoma *in situ*, prostate carcinoma grade group 1, breast ductal carcinoma *in situ*, or carcinoma *in situ* of the cervix. Patients with a history of other malignancies who have been treated with curative intent and without recurrence within 5 years may also be eligible per Investigator judgment
14. Prior liver or heart transplant, and/or Left Ventricular Assist Device (LVAD) or anticipated liver transplant or LVAD within 1 year after randomization
15. Karnofsky performance status of ≤ 50%
16. Contraindication for immunosuppressive therapy, per Investigator's discretion
17. Known Light chain/Primary Amyloidosis (AL)

18. Known leptomeningeal amyloidosis
19. Known history of multiple myeloma
20. Treatment with another investigational drug and/or biological agent within 1 month of Screening, or 5 half-lives of investigational agent, whichever is longer
21. Current or previous treatment with Tegsedi® (inotersen) or Onpattro® (patisiran) or other oligonucleotide or RNA therapeutic (including siRNA; does not apply to COVID-19 mRNA vaccinations)
22. Current treatment with diflunisal, doxycycline with or without ursodeoxycholic acid, and/or non-dihydropyridine calcium-channel blocker (e.g., verapamil, diltiazem). Patients receiving any of these agents must respect a Wash-out Period of 14 days before randomization
23. Unwillingness or inability to comply with study procedures, including follow-up, as specified by this protocol, or unwillingness to cooperate fully with the Investigator
24. Other physical, social, or psychological conditions including illicit drug or alcohol use, which, in the opinion of the Investigator would make the patient unsuitable for inclusion, or could interfere with the patient participating in or completing the Study

6. STUDY PROCEDURES

6.1. Study Schedule

All required study procedures are outlined in Appendix A, Appendix B and Appendix C.

6.1.1. Screening

Before any study-specific procedures or evaluations are initiated, patients must sign and date the informed consent form. Up to 10-week period is given to perform the Screening evaluations and Baselines assessments which must be completed before randomization at Study Day 1. TTR genotyping is only conducted if previous results are not available. An abnormal screening result may be retested if Investigator believes and documents that the results are artifactual, except for NT-proBNP < 600 pg/dL (< 70 pmol/L), which leads to automatic screen failure. These patients may be rescreened in the future if the PI believes the condition has advanced.

The study site will record patient's basic personal details, including name, contact details, gender, height, weight, date of birth, age, and self-reported ethnicity and racial origin (to be used only for clinical purposes).

6.1.2. Day 1/Randomization

During the Day 1 study visit patients will report to the Study Center for evaluations, randomization, and ION-682884 or placebo administration (first dosing should be the last procedure of Day 1 study visit).

The Baseline assessments include, but are not limited to, medical history, concomitant medication, height, weight, physical exam, vital signs, ECG, pregnancy test, Norfolk QOL-DN,

SF-36 Survey, NYHA class, Karnofsky performance status, KCCQ, 6MWT, transthoracic ECHO, and laboratory tests.

Patients with hereditary ATTR-CM should also complete the COMPASS-31 questionnaire. The LLF, 10-meter walk test and PND Score will be assessed in all ATTRv-CM patients by the Investigator or designee.

6.1.3. Treatment Period

The Treatment Period starts at Day 1 after the first Study Drug dosing. After Day 1, the Study Drug will be administered SC once every 4 weeks.

There will be a total of 36 visits during the 140 weeks of the Treatment Period: (13 mandatory in-clinic visits and 23 visits that can be done either in-clinic or completed at home by home-health care) (see Schedule of Procedures in Appendix A). The time window for all study visits during the Treatment Period is of ± 4 days. Echocardiogram has its own time window (± 10 days); see Schedule of Procedures in Appendix A). In the event that a visit does not occur or is delayed, all subsequent visits should be calculated based on the time elapsed since Day 1 rather than from the date of the previous visit. For clinical study visits where a blood draw is required, the blood samples should be drawn prior to Study Drug administration. For Study Drug dosing occurring out of a scheduled mandatory clinic/site study visit, the Study Drug administration can be supported by either Study Center personnel (in which case additional clinic visits will occur) or at home by the patient/caregiver (See Section 8.1). Dosing instructions and training will be provided to the patient on Day 1 and to any caregiver, as needed.

In addition to the Study Drug, patients will also take supplemental doses of the RDA of vitamin A (approximately 3000 IU vitamin A or similar dose as available in the region in which the patient resides). Vitamin A may be taken as either a single vitamin A supplement, or as part of a multivitamin. The vitamin A supplement should be taken throughout the treatment and Post-Treatment Evaluation Period.

Vitamin A deficiency will be monitored by having the patient periodically complete a vitamin A deficiency screening questionnaire (called “ocular questionnaire”). In cases of suspected vitamin A deficiency, a consultation with ophthalmologist may be requested by the Investigator, if deemed necessary, after discussing with the Medical Monitor.

During the Treatment Period, laboratory analyte samples will be collected during study visits approximately every 4 weeks (± 4 days) for the first 6 months and every 3 months thereafter for the remainder of the treatment period according to the Schedule of Procedures (see Appendix A) and List of Laboratory Analytes (see Appendix B).

If the results are uninterpretable (e.g., missing sample, hemolysis) the Investigator should request a new test as soon as possible.

For patients who have Study Drug administration paused or permanently discontinued and/or who withdraw consent to participate in the study, please refer to Section 8.8 and Section 8.9, respectively, for further details.

6.1.4. Week 140

The Week 140 visit occurs at the end of 140 weeks of treatment exposure (Treatment Period) and is considered the last study visit for collection of final data for the primary outcome measures (primary endpoint assessments).

6.1.5. Post-Treatment Evaluation Period

After completion of the Treatment Period and Week 140, patients will enter the 20-week Post-Treatment Evaluation Period. This period consists of 2 in-clinic study visits on Weeks 150 and 160, as outlined in the Schedule of Procedures (Appendix A). Alternatively, after completion of the Treatment Period, eligible patients may elect to receive ION-682884 in an OLE study, pending study approval by the IRB/IEC and the appropriate regulatory authority. In this case, patients may not participate in any or only some of the Post-Treatment Evaluation Period.

6.2. Laboratory Assessments

Laboratory analyte samples will be collected during study visits throughout the study approximately every 4 weeks during the initial 6 months and every 3 months thereafter. A list of these analytes is contained in Appendix B. During the Treatment Period, general blood chemistry and urine samples do not require fasting, with the exception of the assessment of retinyl palmitate (Screening, Weeks 25, 49, 97, 140) which requires fasting for at least 8 hours. During this time the patient can drink water.

If the results of these assessments are uninterpretable (e.g., missing sample, hemolysis) the Investigator should request a new test as soon as possible.

The TTR protein is also known as "prealbumin" and has been used in assessments of nutritional status in critically ill patients and other conditions coursing with severe malnutrition. Because the assessment of TTR or prealbumin is influenced by the administration of ION-682884 we require the Investigators not to assess prealbumin levels in trial participants.

Other than prealbumin, RBP4 and vitamin A, Investigators are allowed to request any test deemed necessary to provide the best care for the patient, per local clinical practice.

6.3. Other Study Procedures

Electrocardiograms

Single 12-lead ECGs will be performed locally per Schedule of Procedures (see Appendix A). The patient should remain in supine position for 5 minutes before recording the ECG, if possible.

ECG parameters should include PR, RR, QRS, QT and QTc intervals, heart rate, cardiac rhythm, and overall clinical interpretation. The results should be recorded in the specific electronic Case Report form (eCRF) section. The original report should be kept in the patient's study source records.

Echocardiograms

Echocardiography will be performed locally per Schedule of Procedures (see Appendix A). The results will be interpreted (for data analysis purposes only) by an independent central laboratory blinded to patient's treatment allocation. The parameters to be analyzed include, but not limited to, Global Longitudinal Strain, Interventricular Septum Thickness, Posterior Wall Thickness, Mean Left Ventricle Wall Thickness, Stroke Volume, Ejection Fraction, Left Ventricular Mass, LVEDD, LVEDV, Diastolic parameters, Right Ventricle parameters. Sites will not receive the interpretations performed by the central echocardiogram laboratory.

The site's analysis of the end-diastolic interventricular septum in the echocardiogram performed during Screening should inform the patient's eligibility per Inclusion Criterion Number 6 before randomization.

Detailed instructions for echocardiogram conduct will be provided.

6.4. Restriction on the Lifestyle of Patients

6.4.1. Contraception Requirements

Male patients must either be abstinent[†] or, if engaged in sexual relations with a woman of childbearing potential (WOCBP), the patient or the patient's non-pregnant female partner must use a highly effective contraception method from the time of signing the informed consent form until at least 24 weeks after their last dose of Study Drug. Male patients with partners that are pregnant must use condoms to ensure that the fetus is not exposed to the Study Drug.

All male patients and WOCBP must either be abstinent[†] or use highly effective contraception from the time of signing the informed consent form until at least 24 weeks after their last dose of study treatment

For the purposes of this study, WOCBP is defined as any female who has experienced menarche, and who does <u>not</u> meet 1 of the following conditions:

- Post-menopausal: 12 months of spontaneous amenorrhea in females > 55 years of age or, in females ≤ 55 years, 12 months of spontaneous amenorrhea without an alternative medical cause <u>and</u> FSH levels in the postmenopausal range for the laboratory involved
- 6 weeks after surgical bilateral oophorectomy with or without hysterectomy
- Post-hysterectomy

For the purposes of the study, highly effective contraception is defined as follows:

For male patients:

- Highly effective male contraception includes a vasectomy with negative semen analysis at follow-up, or the non-pregnant female partner of WOCBP uses a highly effective contraceptive method (defined below)

For female patients and female partners of male patients, highly effective female contraception methods comprise:

- Surgical sterilization (e.g., bilateral tubal occlusion), hormonal contraception associated with inhibition of ovulation (combined estrogen and progestogen containing, or progestogen-only), intrauterine contraception device or intrauterine hormone-releasing system (IUS)

†Note: Abstinence (i.e., refraining from heterosexual intercourse throughout the duration of study participation) is only acceptable as true abstinence, i.e., when this is in line with the preferred and usual lifestyle of the patient. Periodic abstinence (e.g., calendar, ovulation, symptothermal, post-ovulation methods), declaration of abstinence for the duration of a trial and withdrawal are not acceptable methods of contraception.

Note: A female condom and a male condom should not be used together as friction between the two can result in either or both products failing.

7. STUDY DRUG

7.1. ION-682884 / Placebo

Study Drug (ION-682884 [eplontersen] or Placebo) characteristics are listed in Table 2.

The Study Drug is contained in 2-mL stoppered glass vials. The Study Drug and its storage and preparation instructions will be provided by the Sponsor or designee. The Study Drug must be stored securely at 2–8 °Celsius and be protected from light.

Table 2: Study Drug Characteristics

**Study Drug**	**ION-682884**	**Placebo**
**Strength**	150 mg/mL	Not Applicable
**Volume/Formulation**	0.8 mL solution per vial	0.8 mL solution per vial
**Route of Administration**	Subcutaneous	Subcutaneous

7.2. Packaging and Labeling

The Sponsor will provide the Investigator with packaged Study Drug labeled in accordance with specific country regulatory requirements.

7.3. Study Drug Accountability

The study staff is required to document the receipt, dispensing, and return/destruction, according to Sponsor instruction and in accordance with institutional policy of Study Drug supplies provided by the Sponsor.

8. TREATMENT OF PATIENTS

8.1. Study Drug Administration

ION-682884 or placebo will be provided in 2-mL stoppered glass vials with 0.8 mL solution per vial. Vials are for single use only. ION-682884 or placebo will be administered by SC injection once every 4 weeks.

ION-682884 or placebo can be administered in the clinic during clinic visits per protocol or administered at home by the patient/caregiver/home healthcare provider after clinic visits. For Study Drug dosing occurring out of a scheduled mandatory clinic/site study visit the Study Drug administration can be supported by either Study Center personnel (in which case additional clinic visits will occur) or at home by the patient/caregiver/home healthcare provider. Dosing instructions and training will be provided to the patient on Day 1 and to any caregiver, as needed.

Please refer to the Study Drug Manual provided by the Sponsor or designee for more detailed instructions for Study Drug (ION-682884 or placebo) preparation and administration.

Table 3: Study Drug Dosing Information

Study Drug	Volume to Administer	Total Dose	Frequency
**ION-682884**	0.30 mL	45 mg	Every 4 weeks
**Placebo**	0.30 mL	N/A	Every 4 weeks

N/A = not applicable

8.2. Other Protocol-Required Drugs

All study participants will take oral supplemental doses of the RDA of vitamin A (approximately 3000 IU vitamin A or the closest approximate dose as available in the region in which the patient resides). Patients may take vitamin A as either a single vitamin A supplement, or as part of a multivitamin. The vitamin A supplement should be taken throughout the treatment and Post-Treatment Evaluation Period. Vitamin A supplement at dose more than the RDA is not recommended.

8.3. Other Protocol-Required Treatment Procedures

There are no other protocol-required treatment procedures.

8.4. Treatment Precautions

There are no specific treatment precautions required for this study.

8.5. Safety Monitoring Rules

Please refer also to the ‘Guidance for Investigator’ section of the IB.

For the purposes of safety monitoring for liver function, renal function and platelet count Baseline is defined as:

- The average of the pre-dose test closest to Day 1 and Day 1

In addition to the standard monitoring of clinical safety parameters, the following guidelines are provided for the monitoring of selected parameters chosen based on preclinical and clinical observations.

Confirmation Guidance: At any time during the study (Treatment or Post-Treatment Periods), the initial clinical laboratory results meeting the safety monitoring criteria presented below (renal function, liver function, platelet count) **must be confirmed** by performing measurements (ideally in the same laboratory that performed the initial measurement) on new specimens. All new specimen collections should take place as soon as possible (ideally within 3 days of the initial collection). For stopping rules, if the initial laboratory result is observed during the Treatment Period, the results from the retest **must be available** prior to administering the next dose of Study Drug (ION-682884 or placebo).

Re-dosing Guidance: Patients with initial laboratory test values that reach a stopping rule must not be re-dosed until the re-test results are available. In general, patients who do not meet the stopping rules based upon retest may continue dosing. However, the Investigator and the Medical Monitor (or appropriately qualified designee) should confer as to whether additional close monitoring of the patient is appropriate. If any of the stopping criteria described below (refer to Section 8.6.1 to Section 8.6.4) are met, the patient must discontinue Study Drug administration, evaluated fully as outlined below and in consultation with the Medical Monitor or appropriately qualified designee, and will be followed up in accordance with Section 8.6 of the protocol. The reinitiation of Study Drug administration, when appropriate and in accordance with Section 8.6, will be at the Investigator's discretion.

8.5.1. Safety Monitoring Rules for Liver Chemistry Tests

The following rules are adapted from the draft guidance for industry, "Drug-Induced Liver Injury: Premarketing Clinical Evaluation," issued by the U.S. Department of Health and Human Services, Food and Drug Administration, July 2009. For a definition of Baseline, please refer to guidance in Section 8.5 above.

In the event of an ALT or AST measurement that is > 3 × ULN (or 2 × baseline value if the baseline value was > ULN) at any time during the study (Treatment or Post-Treatment Period), repeat testing should be performed within 48 to 72 hours of liver chemistry tests (ALT, AST, alkaline phosphatase [ALP], and total bilirubin) to confirm the abnormalities and to determine if they are increasing or decreasing. There also should be inquiry made about symptoms. Additional, confirmatory measurements should also be performed if ALT or AST levels increase to 5 × ULN.

If new onset symptoms of anorexia, nausea, fatigue, right upper abdominal discomfort, vomiting present with unclear etiology, liver enzymes should be measured.

Frequency of Repeat Measurements: Patients with confirmed ALT or AST levels > 3 × ULN (or 2 × baseline value if the baseline value was > ULN) should have their liver chemistry tests (ALT, AST, ALP, international normalized ratio [INR] and total bilirubin, direct and indirect bilirubin) retested 2 or 3 times weekly. Frequency of retesting can decrease to once a week or less if abnormalities stabilize or the trial drug has been discontinued and the patient is asymptomatic (see below).

Further Investigation into Liver Chemistry Elevations: For patients with confirmed ALT or AST levels > 3 × ULN (or 2 × baseline value if the baseline value was > ULN), the following evaluations should be included in the work up:

- Obtain a more detailed history of symptoms and prior and concurrent diseases
- Obtain further history for concomitant drug use (including nonprescription medications, herbal and dietary supplement preparations), alcohol use, recreational drug use, and special diets
- Obtain a history for exposure to environmental chemical agents and travel
- Serology for viral hepatitis (hepatitis A virus [HAV] immunoglobulin M [IgM], hepatitis B surface antigen [HBsAg], hepatitis C virus [HCV] antibody, cytomegalovirus [CMV] IgM, and EBV antibody panel)
- Serology for autoimmune hepatitis (e.g., antinuclear antibody [ANA], antismooth muscle antibody, type 1 anti-liver kidney microsomal antibody)

Additional liver evaluations, including gastroenterology/hepatology consultations, hepatic CT or MRI scans, may be performed at the discretion of the Investigator, in consultation with the Medical Monitor. Repetition of the above evaluations should be considered if a patient's ALT and/or AST levels reach 5 × ULN.

8.5.2. Safety Monitoring Rules for Platelet Count Results

Please refer also to Table 4 and Table 5.

Platelet count will be monitored every 4 weeks (± 4 days) for the initial 6 months and every 3 months (± 4 days), thereafter during the Treatment Period. The Investigator or designee should review all platelet count results within 4 days of receipt. If there is suspicion of EDTA mediated platelet clumping, a repeat platelet count should be collected in a sodium citrate tube as soon as possible. In the event of confirmed platelet count declines to values < 100×10^9/L, more frequent monitoring will be implemented. If a patient's platelet count falls to < 75×10^9/L, then the Study Drug administration should be paused, additional laboratory investigations should be conducted (Table 4), and the platelet monitoring rule defined in Stopping Rules (Section 8.6.3) should be followed. If a patient's platelet count falls to < 50×10^9/L, the Investigator must communicate to the Sponsor within 24 hours of receiving the platelet result.

Treatment should be held if there is no evaluable platelet count from the prior scheduled safety assessment. Any unreportable platelet count result must be rechecked and determined not to have met a stopping rule before dosing can continue.

Table 4: Additional Labs to be Potentially Performed in the Event of a Platelet Count < 75 × 10^9/L, after Discussion with Study Medical Monitor

**To Be Performed at Local Lab**
Citrated sample for platelets<br>Peripheral smear (should be performed locally, fixed and sent to central lab for review)
Fibrinogen split products or D-dimer on fresh blood
Helicobacter pylori (IgG serum test or alternative per local availability)
**To Be Performed at Central Lab**
Coagulation panel (PT/INR, aPTT)
CBC with reticulocytes and mean platelet volume (MPV)
Serum B12 and folate
Fibrinogen
von Willebrand factor
Total globulins, total IgA, IgG and IgM
Complement: total C3, total C4, Bb, C5a
hsCRP
Serology for:
HBV, HCV, HIV (if not done for screening)
Rubella
CMV
EBV
Parvo B19
Auto-antibody screen:
Antiphospholipid
Rheumatoid factor
Anti-dsDNA
Anti-thyroid
**To Be Performed at Specialty Lab**
Antiplatelet antibodies and Anti-PF4 assay
Anti-ASO antibody

Note: The above labs may change as additional data is assessed, and sites will be updated regarding any changes.

8.5.3. Safety Monitoring for Minor Bleeding Events

Minor bleeding events are those that do not fulfill the criteria for major bleeding (MB) or clinically-relevant, non-major bleeding events (which are defined in Section 8.6.3), for example excess bruising, petechiae, gingival bleeding on brushing teeth. If a minor bleeding event occurs, additional testing of coagulation parameters (aPTT, prothrombin time [PT], INR) and platelet count should be performed.

8.5.4. Safety Monitoring for Ocular Effects

Periodic assessment of vitamin A deficiency questionnaire (ocular questionnaire) will be conducted to all patients during the trial. A consultation with ophthalmologist may be requested by the Investigator, if deemed necessary, after discussing with the Medical Monitor.

8.5.5. Safety Monitoring Rules for Renal Function

Urine protein-creatinine ratio and eGFR will be assessed every 4 weeks (± 4 day) for the initial 6 months and every 3 months (± 4 days), thereafter. See Section 8.6.2 for further instructions.

8.6. Stopping Rules

For the purposes of the stopping rules, Baseline is defined as: average of the pre-dose test closest to Day 1 and Day 1.

8.6.1. Stopping Rules for Liver Chemistry Elevations

In the event of laboratory results meeting the following criteria, **and the event is without an alternative explanation as discussed with the Medical Monitor,** dosing of a patient with Study Drug will be stopped permanently; values that are not confirmed due to failure to retest or missing lab values will be presumed confirmed:

1. ALT or AST > 8 × ULN, which is confirmed
2. ALT or AST > 5 × ULN, which is confirmed and persists for ≥ 2 weeks
3. ALT or AST > 3 × ULN (or the greater of 2 × baseline value or 3 × ULN if the baseline value was > ULN), which is confirmed **and** total bilirubin > 2 × ULN or INR > 1.5
4. ALT or AST > 3 × ULN (or the greater of 2 × baseline value or 3 × ULN if the baseline value was > ULN), which is confirmed, **and** the new appearance (i.e., onset coincides with the changes in hepatic enzymes) of fatigue, nausea, vomiting, right upper quadrant pain or tenderness, fever, rash, and/or concomitant eosinophilia (> ULN)

8.6.2. Stopping Rules for Renal Function Test Results

Dosing should be interrupted in the event of an alteration observed and confirmed, for either of the 2 criteria below:

1. A decrease in eGFR (mL/min/1.73 mm^2) > 30% compared to Baseline [eGFR should be calculated using the CKD-EPI creatinine equation (Levey *et al.* 2009)]; if the eGFR is thought to be underestimated, the CKD-EPI creatinine-cystatin C equation should be used to confirm (Inker *et al.* 2012). This criterion does not apply for events which the Investigator considers not related to Study Drug administration. A discussion with Medical Monitor is needed and a reassessment of the event must be conducted if the

relationship with Study Drug changes, per Investigator's opinion. Such events must be followed and managed as AE/SAE per Section 9.4

2. Proteinuria: Absolute UPCR value ≥ 1000 mg/g (confirmed by a repeat random spot UPCR ≥ 1000 mg/g)

If Study Drug administration is paused per the rules above, once eGFR returns to the prior value, UPCR decreases to below 1000 mg/g, or the underlying cause of the decline in renal function is corrected, dosing may be reinitiated. In the case of UPCR of 2000 mg/g or higher, further evaluation for renal disease should be performed. If acute glomerulonephritis is confirmed, treatment should be permanently discontinued.

The follow-up schedule for any events meeting either of these criteria will be determined by the Investigator **in consultation with** the Medical Monitor or designee. Re-initiation of dosing will be at the discretion of the Investigator.

8.6.3. Stopping Rule for Platelet Count Results

In the event of a confirmed platelet count $< 75 \times 10^9$/L the Study Drug administration will be interrupted, the platelet count monitoring will be increased to weekly, and additional laboratory investigations will be conducted. If the platelet count decreases to $< 50 \times 10^9$/L, the monitoring should be increased to 2–3 assessments per week and the Medical Monitor must be contacted within 24 hours to discuss the case. A hematologist consultation may be requested, per Investigator's discretion. If the platelet count decreases to $< 25 \times 10^9$/L, the monitoring should be increased to daily and a hematologist consultation to discuss treatment with corticosteroids is mandatory. In the event of a platelet count $< 10 \times 10^9$/L the patient should be hospitalized and be treated with corticosteroids and platelet transfusion, per hematologist's discretion (see Table 5).

The Study Drug administration should be permanently discontinued in patients whose platelet count reached values $< 25 \times 10^9$/L. For the patients who temporarily paused Study Drug administration due to platelet counts $< 75 \times 10^9$/L and $\geq 25 \times 10^9$/L the re-challenge can occur after 2 consecutive assessments results $\geq 100 \times 10^9$/L, per Investigator's discretion.

The suitability of the patient for continued dosing will be determined by the Investigator in consultation with the Medical Monitor and will be based on the rules above and factors such as the original rate of decline in the patient's platelet count, whether any bleeding events were experienced, and the speed of recovery of platelet count after interruption of dosing. Any unreportable platelet count result must be rechecked and determined not to have met a stopping rule before dosing can continue.

Definition of Major Bleeding Events (Schulman & Kearon 2005)

1. Fatal bleeding, and/or
2. Symptomatic bleeding in a critical area or organ, such as intracranial, intraspinal, intraocular, retroperitoneal, intraarterial or pericardial, or intramuscular with compartment syndrome
3. Clinically overt bleeding leading to transfusion of ≥ 2 units of packed red blood cells or whole blood or a fall in hemoglobin of 2.0 mg/dL (1.24 mmol/L) or more within 24 hours

Definition of Clinically Relevant Non-Major Bleeding Events

Clinically relevant non-major bleeding (CRNMB) is defined as overt bleeding not meeting the criteria for major bleeding (MB) but that resulted, for example, in medical examination, intervention, or had clinical consequences for a patient.

Definition of Minor Bleeding Events

Minor bleeding events are those that do not fulfill the criteria for MB or clinically relevant, non-major bleeding events (defined above), for example excess bruising, petechiae, gingival bleeding on brushing teeth.

Table 5: Actions in Patients with Low Platelet Count

If the subsequent test confirms the initial test result, then monitoring frequency and dosing should be adjusted as recommended in the table.

**Platelet Count ($\times 10^9$/L)**	**Monitoring Frequency**	**Treatment**
<125 - ≥ 100	Monthly	Investigator's discretion
< 100 – ≥ 75	Every 2 weeks	Investigator's discretion
< 75 – ≥ 50	Weekly	Treatment must be paused until 2 consecutives values ≥ 100 × 10^9/L
< 50 – ≥ 25	Twice weekly until 2 consecutives values ≥ 75 × 10^9/L, then weekly monitoring until ≥ 100 × 10^9/L, then per Schedule of Procedures<br>Consider more frequent monitoring if additional risk factors for bleeding are present<br>**Note: if a patient's platelet count falls to < 50 × 10^9/L, the Investigator must communicate to the Sponsor within 24 hours of receiving the platelet result**	Treatment should be paused until 2 consecutives values ≥ 100 × 10^9/L. Consider haematologist consultation and corticosteroids administration if additional risk factors for bleeding are present
< 25 – ≥ 10[‡*]	Daily until 2 consecutives values ≥ 25 × 10^9/L, then monitor twice weekly until 2 consecutives values ≥ 75 × 10^9/L, then weekly monitoring until ≥ 100 × 10^9/L, then per Schedule of Procedures	Treatment should be permanently discontinued.<br>Haematologist consultation is mandatory.
< 10[*]	Patient should be hospitalized	Treatment should be permanently discontinued.<br>Haematologist consultation is mandatory.

‡ Additional risk factors for bleeding include age > 60 years, receiving anticoagulant or antiplatelet medicinal products, and /or prior history of MB events

* It is strongly recommended that, unless corticosteroids are contraindicated, the patient receives glucocorticoid therapy to reverse the platelet decline as recovery in platelet count may be accelerated by administration of high dose steroids. Treatment guidelines for immune thrombocytopenia (Provan *et al.* 2010) recommend Dexamethasone 40 mg daily for 4 days every 2–4 weeks for 1–4 cycles; Prednis(ol)one 0.5–2 mg/kg/d for 2-4 weeks then taper; or Methylprednisolone 30 mg/kg/day for 7 days (note: may require continuation with oral steroids after methylprednisolone)

8.6.4. Stopping Rule for Ocular Effects

A patient should be permanently discontinued from Study Drug administration if he presents clear signs of vitamin A deficiency as confirmed by ophthalmologist consultation and discussed with the Investigator.

8.7. Adjustment of Dose and/or Treatment Schedule

Dose frequency adjustments, including dose interruptions, are allowed for reasons of safety or tolerability, per discussion with Medical Monitor.

8.8. Discontinuation of Study Drug Administration

The reasons for permanent discontinuation of Study Drug administration are:

- Patient request (no consent withdrawal for study participation; not related to AE)
- Patient withdrawal of consent for study participation (not related to AE)
- The patient experiences an AE that necessitates permanent discontinuation of study treatment
  - At the discretion of the Investigator due to a safety concern
  - At the patient's request due to an adverse event
  - The patient presents laboratory test abnormalities that meet any of the stopping rules listed in Section 8.6.1 to Section 8.6.4
  - The patient becomes pregnant. Report the pregnancy according to instructions in Section 9.5.4
- Protocol specific requirements
  - The patient receives major organ transplantation or LVAD insertion
  - The patient starts disallowed medications specified in Section 8.10.1
- Investigator decision (not related to AE, e.g., non-compliant patient)
- Sponsor decision (not related to AE)
- Disease progression (not related to AE, e.g., hospice admission)

The reason for discontinuation of Study Drug administration must be recorded in the eCRF and source documentation.

Patients who had Study Drug administration stopped for any reason (i.e., Investigator decision, protocol requirement, patient request, or Sponsor decision) other than withdrawal of consent for study participation, should continue their participation in the study by attending visits and procedures as outlined in the Schedule of Procedures (Appendix A), if possible, and by providing vital status at the time the patient was expected to reach the end of their Treatment Period (Week 140), the end of their Post-Treatment Evaluation Period (Week 160), and up to the Primary Completion Date of the study. This is the preferred follow-up approach, and Investigators should encourage participants to continue in the study as described above.

If the patient declines or is unable to attend study visits, the Investigator should clarify what type of follow-up the patient is agreeable to: in person, by phone/mail, through family/friends, via correspondence/communication with other physicians, and/or from review of the medical records, and as allowed per local regulations. Wherever possible, these patients should be encouraged to continue to be followed up via the agreed means to collect information on AE, concomitant medications and vital status. At the very least, the patient's vital status should be ascertained and documented wherever possible by the time the patient was expected to reach the end of their Treatment Period (Week 140), the end of their Post Treatment Evaluation Period (Week 160), and up to the Primary Completion Date of the study. The agreed means of follow-up will be documented in the patient records and notified to the Sponsor.

8.9. Discontinuation of Study Participation

The reasons for discontinuing patient participation in the study are:

- Patient withdrawal of consent for study participation
- At the discretion of the Investigator or Sponsor for noncompliance or significant protocol deviation

All efforts will be made to complete and report the observations as thoroughly as possible up to the date of withdrawal. All information, including the reason for withdrawal from study, must be recorded in the eCRF.

Full withdrawal of consent: Any patient who withdraws consent to participate in the study will be removed from further treatment and study observation immediately upon the date of request. These patients should be encouraged to complete the Early Termination Visit at the time of withdrawal (Appendix A). Any data including stored samples or imaging collected up to the time of withdrawal of consent will still be included, analyzed, and reported as part of the study data in order to preserve the scientific integrity of the research being conducted and to comply with legal obligations of the Sponsor (data controller). Patients who withdraw consent may have vital status follow-up if permitted by local laws of jurisdiction.

8.9.1. Lost to Follow-up

To prevent participants being lost to follow-up, their contact details, including next of kin contacts, should be collected, and updated regularly by the site staff or representative. The Investigator should educate the participant on the importance of contact with Investigator throughout the study.

Repeated attempts will be made to locate and obtain pertinent medical information for participants who are initially lost to follow-up. A participant will be classified as lost to follow-up only if he/she has failed to return for the required study visits and his/her vital status remains unknown despite multiple attempts to contact him/her via telephone, fax email, certified letter, or through patient locator agencies (if allowed by national regulation). Where permissible by local law, an Informed Consent Document will include language to grant the option to employ outside companies to assist in obtaining updated contact information or ascertainment of vital status of lost participants using publicly available source.

8.10. Concomitant Therapy and Procedures

The use of concomitant therapies or procedures defined below must be recorded on the patient's eCRF. Adverse events related to administration of these therapies or procedures must also be documented on the appropriate eCRF. If a patient withdraws consent, all efforts should be made upon receipt of the withdrawal communication to obtain current status of all medication being taken.

8.10.1. Concomitant Therapy

A concomitant therapy is any non-protocol specified drug or substance (including over-the-counter medications, herbal medications and vitamin supplements) administered between signing of informed consent and the last study visit.

Patients are required to take RDA supplemental doses of vitamin A during the treatment and Post-Treatment Evaluation Periods. The vitamin A supplements may be provided by the Study Center or designee or patients may choose to supplement with their own multivitamin containing RDA vitamin A only after reviewed and approved by the Investigator. Additional vitamin A supplements (other than those described above) are not allowed at any time during the study unless approved by the Investigator (this includes multivitamin supplements that contain vitamin A).

Allowed Concomitant Therapy

Any medications deemed necessary by the Investigator are allowed except those listed in the disallowed concomitant therapy.

The use of tafamidis (and acoramidis, if marketing is locally approved) is allowed as a concomitant medication at the locally approved dosage regimen for ATTR-CM or ATTR-PN (including compassionate access, extended access, and other), per physician discretion.

All concomitant medication should be recorded in the proper section of the eCRF.

Disallowed Concomitant Therapy

Current or previous treatment with Tegsedi® (inotersen) or Onpattro® (patisiran) or Amvuttra® (vutrisiran) or other oligonucleotide or RNA therapeutic (including siRNA) is not allowed. The initiation of any of these treatments will require Study Drug permanent discontinuation.

Non-dihydropyridine calcium channel blockers (e.g., verapamil, diltiazem) are contraindicated in patients with ATTR-CM and are not allowed.

The use of diflunisal and/or doxycycline with or without ursodeoxycholic acid is not approved for the treatment of ATTR-CM and is not allowed as concomitant therapy in this trial. Patients receiving these agents must respect a Wash-out Period of 14 days before randomization.

The use of doxycycline for an indication other than for the treatment of ATTR-CM is an allowed concomitant medication (e.g., if used as an antibiotic for a short period of time [5-30 days average] while the patient is on study). The same applies for non-ATTR-CM indications for the use of ursodeoxycholic acid, such as if used for the treatment of gallbladder stones and some severe dyslipidemias.

8.10.2. Concomitant Procedures

A concomitant procedure is any therapeutic intervention (e.g., surgery/biopsy, physical therapy) or diagnostic assessment (e.g., blood gas measurement, bacterial cultures) performed between signing of informed consent and last study visit.

Patient participation in another non-interventional trial concurrent with this trial is allowed, as long as the study does not include another investigational agent (e.g., natural history study) and does not interfere with the schedule of procedures for this trial.

8.11. Treatment Compliance

Compliance with treatment dosing is monitored and recorded in the eCRF by Study Center staff.

Patients will record treatment in a dosing diary that will be reviewed periodically by Study Center staff and the Clinical Monitor.

9. SERIOUS AND NON-SERIOUS ADVERSE EVENT REPORTING

9.1. Sponsor Review of Safety Information

Safety information will be collected, reviewed, and evaluated by the Sponsor in accordance with the applicable Ionis and/or designee Standard Operation Procedures (SOP) throughout the conduct of the clinical trial.

9.2. Regulatory Requirements

The Sponsor is responsible for regulatory submissions and reporting to the Investigators of SAEs including suspected unexpected serious adverse reactions (SUSARs) per the International Conference on Harmonization (ICH) guidelines E2A and ICH GCP. Country-specific regulatory requirements will be followed in accordance with local country regulations and guidelines.

Institutional Review Boards/Independent Ethics Committees will be notified of any SAE according to applicable regulations. The DSMB will be notified of any SAE as specified in the DSMB charter.

In addition to the Investigator’s assessment of relatedness, the Sponsor will evaluate the available information and perform an independent assessment of all reported SAEs and determine if there is a reasonable possibility that the Study Drug (ION-682884 or placebo) is causally related to a reported SAE. While the Sponsor may upgrade an Investigator’s decision; however, it is not permissible to downgrade the Investigator’s opinion for the purposes of determining whether the SAE meets the definition of a SUSAR.

Appropriate personnel at the Sponsor or designee will unblind SUSARs for the purpose of regulatory reporting. The Sponsor or designee will submit SUSARs to Regulatory Agencies in blinded or unblinded fashion according to local law. The Sponsor will submit SUSARs to Investigators in a blinded fashion.

For the purpose of regulatory reporting of SUSARs, there are no "expected" AEs related to the Study Drug. The ATTR-CM population in this study is expected to experience a substantial rate of CV clinical events. These events will be reported as SUSARs if considered related to Study Drug.

9.3. Definitions

9.3.1. Adverse Event

An adverse event (AE) can be any unfavorable and unintended sign (including an abnormal laboratory finding, for example), symptom, or disease temporally associated with the use of medicinal (investigational) product, whether or not the AE is considered related to the medicinal (investigational) product.

An AE can therefore be any of the following:

- Any unfavorable and unintended sign (including an abnormal laboratory finding), symptom, or disease temporally associated with the use of a medicinal product, whether or not considered related to the medicinal product
- Any new disease or exacerbation of an existing disease (a worsening in the character, frequency, or severity of a known condition)
- Recurrence of an intermittent medical condition (e.g., headache) not present at Baseline
- Any deterioration in a laboratory value or other clinical test (e.g., ECG, X-ray) that is associated with symptoms or leads to a change in study treatment or concomitant treatment or discontinuation from Study Drug
- Adverse events that are related to a protocol-mandated intervention, including those that occur prior to assignment of study treatment (e.g., screening invasive procedures such as biopsies)

9.3.2. Adverse Drug Reaction and Suspected Unexpected Adverse Drug Reaction

Adverse Drug Reaction (ADR)

In the *pre-approval* clinical experience with a new medicinal product or its new usages, particularly as the therapeutic dose(s) may not have been established, ADR is defined as follows:

- All noxious and unintended responses to a medicinal product related to any dose should be considered adverse drug reactions.

The phrase "responses to a medicinal product" means that a causal relationship between the medicinal product and the AE has been determined by the Sponsor as at least a reasonable possibility, i.e., the relationship cannot be ruled out.

Suspected Unexpected Adverse Drug Reaction

A suspected unexpected ADR is any ADR, the nature or severity of which is not consistent with the applicable product information, e.g., IB for an unapproved medicinal (investigational) product.

A suspected adverse reaction implies a lesser degree of certainty about causality than an adverse reaction.

9.3.3. Serious Adverse Event

A SAE is any AE that in the view of either the Investigator or Sponsor, meets any of the following criteria:

- Results in death
- Is life threatening: that is, poses an immediate risk of death at the time of the event
- An AE or suspected adverse reaction is considered "life-threatening" if, in the view of either the Investigator or Sponsor, its occurrence places the patient at immediate risk of death. It does not include an AE or suspected adverse reaction that, had it occurred in a more severe form, might have caused death
- Requires inpatient hospitalization or prolongation of existing hospitalization
- Hospitalization is defined as an admission of greater than 24 hours to a medical facility and does not always qualify as an AE
- Results in a persistent or significant incapacity or substantial disruption of the ability to conduct normal life functions
- Results in a congenital anomaly or birth defect in the offspring of the patient (whether the patient is male or female)
- <u>Important medical events</u> that may not result in death, are not life-threatening, or do not require hospitalization may also be considered serious when, based upon appropriate medical judgment, they may jeopardize the patient and may require medical or surgical intervention to prevent 1 of the outcomes listed in this definition. Examples of such medical events include allergic bronchospasm requiring intensive treatment in an emergency room or at home, blood dyscrasias or convulsions that do not result in inpatient hospitalization, or the development of drug dependency or drug abuse

The terms "severe" and "serious" are not synonymous. Severity refers to the intensity of an AE (e.g., rated as mild, moderate, or severe, or according to National Cancer Institute Common Terminology Criteria for Adverse Events [NCI CTCAE]; OR Toxicity Grading Scale for Healthy Adult and Adolescent Volunteers Enrolled in Preventive Vaccine Clinical Trials; the event itself may be of relatively minor medical significance (such as severe headache without any further findings).

Severity and seriousness need to be independently assessed for each AE recorded on the eCRF.

9.3.4. Adverse Event of Special Interest

For the purpose of this study, severe reductions in platelet count $< 50 \times 10^9$/L accompanied by a MB or CRNMB, or platelet count of $< 25 \times 10^9$/L independent of a MB or CRNMB event are considered as AEs of special interest and are subject to 15-day expedited reporting by the Sponsor to the regulatory agencies.

9.4. Monitoring and Recording Adverse Events

Any pre-existing conditions or signs and/or symptoms present in a patient prior to the start of the study (i.e., before informed consent) should be recorded as Medical History and not recorded as AEs unless the pre-existing condition worsened. The Investigator should always group signs and

symptoms into a single term that constitutes a **single unifying diagnosis** if possible. Before a diagnosis is confirmed, all symptoms should be reported as separate AEs.

9.4.1. Serious Adverse Events

In the interest of patient safety, and in order to fulfill regulatory requirements**,** all SAEs (regardless of their relationship to Study Drug) should be reported to the Sponsor within 24 hours of the Study Center's first knowledge of the event. The collection of SAEs will begin after the patient signs the informed consent form and stops at the end of the patient's Post-Treatment Evaluation Period which is defined as study visit Week 160, or at 20 weeks after the patient's last dose, whichever occurs first. For patients enrolled in the OLE, collection of SAEs in ION-682884-CS2 will stop once the patient receives their first dose of eplontersen in the extension study. SAEs should be reported using an electronic SAE submission from whenever possible. In situations where the electronic SAE submission is unavailable or if an SAE occurs during the Screening Period, paper Initial Serious Adverse Event Form should be completed, and a copy should be faxed or emailed to the Sponsor. The SAE reporting instruction, including the fax number and email address can be found in the Investigator site file for the study.

Detailed information should be actively sought and included as follow-up when submitting electronically or on Follow-Up Serious Adverse Event Forms if reporting by paper as soon as additional information becomes available. All SAEs will be followed until resolution. SAEs that remain ongoing past the patient's last protocol-specified follow-up visit will be evaluated by the Investigator and Sponsor. If the Investigator and Sponsor agree the patient's condition is unlikely to resolve, the Investigator and Sponsor will determine the follow-up requirement.

9.4.2. Non-Serious Adverse Events

The recording of non-serious AEs will begin after the patient signs the informed consent form and will stop at the end of the patient's Post-Treatment Evaluation Period, which is defined as study visit Week 160, or at 20 weeks after the patient's last dose, whichever occurs first. For patients enrolled in the OLE, collection of AEs in ION-682884-CS2 will stop once the patient receives their first dose of eplontersen in the extension study. The Investigator will monitor each patient closely and record all observed or volunteered AEs on the Adverse Event Case Report Form.

9.4.3. Evaluation of Adverse Events (Serious and Non-Serious)

The Investigator's opinion of the following should be documented on the Adverse Event Case Report Form.

9.4.3.1. Relationship to the Study Drug

The event's relationship to the Study Drug (ION-682884 or placebo) is characterized by 1 of the following:

- **Related:** There is clear evidence that the event is related to the use of Study Drug, e.g., confirmation by positive re-challenge test

- **Possible:** The event cannot be explained by the patient's medical condition, concomitant therapy, or other causes, and there is a plausible temporal relationship between the event and Study Drug (ION-682884 or placebo) administration
- **Unlikely/Remote:** An event for which an alternative explanation is more likely (e.g., concomitant medications or ongoing medical conditions) or the temporal relationship to Study Drug ION-682884 or placebo) administration and/or exposure suggests that a causal relationship is unlikely (for reporting purposes, Unlikely/Remote will be grouped together with Not Related)
- **Not Related:** The event can be readily explained by the patient's underlying medical condition, concomitant therapy, or other causes, and therefore, the Investigator believes no relationship exists between the event and Study Drug

9.4.3.2. Severity

The severity of AEs and SAEs relating to laboratory tests and AE at the injection site will be graded based on criteria from the Common Terminology Criteria for Adverse Events (CTCAE) Version 5.0, November 2017 (refer to Appendix C). Any AE not listed in Appendix C will be graded as follows:

- **Mild:** The event is easily tolerated by the patient and does not affect the patient's usual daily activities
- **Moderate:** The event causes the patient more discomfort and interrupts the patient's usual daily activities
- **Severe:** The event is incapacitating and causes considerable interference with the patient's usual daily activities

If the event is an SAE, then all applicable seriousness criteria must be indicated (criteria listed in Section 9.3.3).

9.4.3.3. Action Taken with Study Drug

Action taken with Study Drug (ION-682884 or placebo) due to the event is characterized by 1 of the following.

- **None:** No changes were made to Study Drug (ION-682884 or placebo) administration and dose
- **Not Applicable:** SAE/AE was reported during Screening Period prior to Study Drug administration
- **Permanently Discontinued:** Study Drug was discontinued and not restarted
- **Temporarily Interrupted, Restarted – Same Dose:** Dosing and/or dosing frequency was temporarily interrupted/changed or delayed due to the AE and restarted at the same dose

9.4.3.4. Treatment Given for Adverse Event

Any treatment (e.g., medications or procedures) given for the AE should be recorded on the Adverse Event Case Report Form. Treatment should also be recorded on the concomitant treatment or ancillary procedures eCRF, as appropriate.

9.4.3.5. Outcome of the Adverse Event

If the event is a non-serious AE, then the event's outcome is characterized by 1 of the following:

- **Ongoing:** AE continues
- **Recovered:** Patient recovered completely from the AE
- **Became Serious:** The event became serious (the date that the event became serious should be recorded as the Resolution Date of that AE and the Onset Date of the corresponding SAE)
- **Change in Severity (if applicable):** AE severity changed

If the event is an SAE, then the event's outcome is characterized by 1 of the following:

- **Ongoing:** SAE continuing
- **Persists (as non-serious AE):** Patient has not fully recovered but the event no longer meets serious criteria and should be captured as an AE on the non-serious AE eCRF (the SAE resolution date should be entered as the date of onset of that AE)
- **Recovered:** Patient recovered completely from the SAE (the date of recovery should be entered as the SAE resolution date)
- **Recovered with Sequelae:** The signs/symptoms of the reported SAE have improved but not completely resolved, and a new baseline for the patient is established since full recovery is not expected
- **Fatal:** Patient died (the date of death should be entered as the SAE resolution date)
- **Unknown:** The outcome of the reported SAE is not available, e.g., patient is lost to follow-up

9.4.3.6. Follow-up of Adverse Event

Investigator Follow-Up

During the Study Period, the Investigator should follow each AE until the event has resolved to baseline grade or better, the event is assessed as stable, the patient is lost to follow-up, or the patient withdraws consent. Every effort should be made to follow all SAEs considered to be related to Study Drug or related to study procedures until a final outcome can be reported.

In the event a patient indicates a desire to withdraw consent from study participation, all efforts should be made to collect information regarding new AEs and updates regarding previously reported AEs.

Resolution of AE (with dates) should be documented on the Adverse Event eCRF and in the patient's medical record to facilitate source data verification.

Investigator should follow-up or support the Sponsor's effort to follow up with all pregnancies reported during the study from either the study patient or the female partner of male study patient until pregnancy outcome is available.

Sponsor Follow-Up

For AESI and pregnancy cases in patients who has completed or terminated study, the Sponsor or a designee should follow-up by telephone, fax, email, and/or a monitoring visit to obtain additional case details and outcome information (e.g., from hospital discharge summaries, consultant reports, autopsy reports) in order to perform an independent medical assessment of the reported case.

9.5. Procedures for Handling Special Situations

9.5.1. Abnormalities of Laboratory Tests

Clinically significant abnormal laboratory test results may, in the opinion of the Investigator, constitute or be associated with an AE. Examples of these include abnormal laboratory results that are associated with symptoms, or require treatment, e.g., bleeding due to thrombocytopenia, tetany due to hypocalcemia, or cardiac arrhythmias due to hyperkalemia. These abnormal laboratory tests must be reported as AEs in accordance with Section 9.4. Whenever possible, the underlying diagnosis should be listed as the AE terms in preference to abnormal laboratory values. Clinically significant abnormalities will be monitored by the Investigator until the parameter returns to its baseline value or until agreement is reached between the Investigator and Medical Monitor. Laboratory abnormalities deemed not clinically significant (NCS) by the Investigator should not be reported as AEs. Similarly, laboratory abnormalities reported as AEs by the Investigator should not be deemed NCS on the laboratory sheet.

The Investigator is responsible for reviewing and signing all laboratory reports. The signed clinical laboratory reports will serve as source documents and should include the Investigator’s assessment of clinical significance of out of range/abnormal laboratory values.

9.5.2. Prescheduled or Elective Procedures or Routinely Scheduled Treatments

A prescheduled or elective procedure or a routinely scheduled treatment will not be considered an SAE, even if the patient is hospitalized; the Study Center must document all of the following:

- The prescheduled or elective procedure or routinely scheduled treatment was scheduled (or was on a waiting list to be scheduled) prior to obtaining the patient’s consent to participate in the study
- The condition that required the prescheduled or elective procedure or routinely scheduled treatment was present before and did not worsen or progress in the opinion of the Investigator between the patient’s consent to participate in the study and the timing of the procedure or treatment
- The prescheduled or elective procedure or routinely scheduled treatment is the sole reason for the intervention or hospital admission

9.5.3. Dosing Errors

Study Drug (ION-682884 or placebo) errors (including overdose, underdose, and administration errors) should be documented as Protocol Deviations, subcategory Dosing Error. A brief description should be provided in the deviation, including whether the patient was symptomatic (list symptoms) or asymptomatic, and whether the event was accidental or intentional.

Dosing details should be captured on the Dosing Case Report Form. If the patient takes a dose of Study Drug (ION-682884 or placebo) that exceeds protocol specifications and the patient is symptomatic, then the symptom(s) should be documented as an AE related to Study Drug administration error and be reported per Section 9.4.

An overdose is the accidental or intentional use of a drug in an amount higher than the dose being studied. An overdose or incorrect administration of study treatment is not itself an AE, but it may result in an AE. Any overdose or incorrect administration of Study Drug should be noted on the Study Drug Administration Electronic Case Report Form (eCRF). Adverse events associated with an overdose or incorrect administration of Study Drug should be recorded on the Adverse Event eCRF. If the associated AE fulfills seriousness criteria, the event should be reported to the Sponsor immediately (i.e., no more than 24 hours after learning of the event).

Should an overdose occur, the Investigator or designee should refer to the Guidance to Investigator's section of the IB and contact the Sponsor or designee within 24 hours, regardless the presence or not of an AE.

9.5.4. Contraception and Pregnancy

Male patients and female patients of childbearing potential must continue to use appropriate contraception with their partners, or refrain from sexual activity, as described in Section 6.4.1.

If a patient becomes pregnant or a pregnancy is suspected, or if a male patient makes or believes that he has made someone pregnant during the study, then the Study Center staff must be informed immediately. An Initial Pregnancy Form should be submitted via FAX or email to the Sponsor **within 24 hours** of first learning of the occurrence of pregnancy. Follow-up information including delivery or termination is reported by designating as 'Follow-up' on the Pregnancy Forms and reported within 24 hours.

Payment for all aspects of obstetrical care, child or related care will be the patient's responsibility.

<u>Female patients</u>: If a suspected pregnancy occurs while on the study (including follow-up), a pregnancy test will be performed. The patient with a confirmed pregnancy will be immediately withdrawn from treatment with Study Drug. However, the patient will be encouraged to complete the Post-Treatment Evaluation Period of the study to the extent that study procedures do not interfere with the pregnancy. Regardless of continued study participation, the study physician will assist the patient in getting obstetrical care and the progress of the pregnancy will be followed until the outcome of the pregnancy is known (i.e., delivery, elective termination, or spontaneous abortion). If the pregnancy results in the birth of a child, the Study Center and Sponsor may require access to the mother and infant's medical records to obtain additional information relevant to the pregnancy progress and outcome. A longer follow-up may be required if a newborn child experiences a medical condition. Follow-up will be performed to the extent permitted by the applicable regulations and privacy considerations; e.g., pregnancy informed consent form may be required.

<u>Male patients</u>: The progress of the pregnancy of a male patient's partner should be followed until the outcome of the pregnancy is known (i.e., delivery, elective termination, or spontaneous abortion). If the pregnancy results in the birth of a child, **the Study Center and Sponsor may follow-up with the mother and may request access to the mother and infant's medical**

records to obtain additional information relevant to the pregnancy progress and outcome. A longer follow-up may be required if a newborn child experiences a medical condition. Follow-up will be performed to the extent permitted by the applicable regulations and privacy considerations; e.g., partner informed consent form may be required.

9.5.5. Reporting Serious Breaches

Regulatory Reporting Requirements for Serious Breach:

- Prompt notification by the Investigator to the Sponsor of any (potential) serious breach of the protocol or regulations is essential so that legal and ethical obligations are met.
  - A ‘serious breach’ means a breach likely to affect to a significant degree the safety and rights of a participant or the reliability and robustness of the data generated in the clinical trial.
- If any (potential) serious breach occurs in the course of the study, Investigators or other site personnel will inform the appropriate Ionis representatives immediately but no later than 24 hours of when they become aware of it.
- In certain regions/countries, the Sponsor has a legal responsibility to notify both the local regulatory authority and other regulatory agencies about such breaches.
  - The Sponsor will follow country-specific regulatory requirements relating to serious breach reporting
- The Investigator should have a process in place to ensure that:
  - The site staff or service providers delegated by the Investigator/institution are trained and able to identify the occurrence of a (potential) serious breach.
  - A (potential) serious breach is promptly reported to the Sponsor or delegated party, through the contacts provided by the study team.

10. STATISTICAL CONSIDERATIONS

10.1. Stratification, Subsets, and Covariates

The stratification factors include:

- NYHA Classification (I and II vs. III)
- ATTR mutation status (mutated vs. wild-type)
- 6MWT distance (6MWT ≤ 350 meters vs. > 350 meters)
- Current treatment with tafamidis (Yes vs. No) at Baseline

Prespecified subgroup analysis for primary and secondary efficacy endpoints will include, but not limited to, NYHA Classification, ATTR mutation status, current treatment with tafamidis (Yes vs. No), time on tafamidis treatment, region (North America, Europe, and ROW), gender, and age. Details of the prespecified subgroup analyses will be provided in the SAP.

In general, the covariates will include stratification factors and baseline measurements (where applicable). Log-transformed baseline NT-proBNP will be included as a covariate in the analysis for primary endpoint, and secondary endpoints including all-cause death, CV death, recurrent CV clinical events. The details will be provided in Section 10.6.2 and in the SAP.

10.2. Sample Size Considerations

The sample size calculation assumes treatment effect of 20% reduction in the relative risk of CV death and recurrent CV clinical events of treatment arm compared with placebo over a period up to 140 weeks. Using a two-sided test with an alpha level of 0.05, a sample size of approximately 1400 will provide a power of ~ 85% with treatment duration of 140 weeks considering a drop-out of 5-10%.

10.3. Populations

Full Analysis Set: all randomized patients who received at least 1 injection of ION-682884 or placebo and who have at least 1 post-Baseline efficacy assessment (i.e., post-Baseline CV clinical event, post-Baseline study visit, date of death).

Safety Population: All patients who are randomized and receive at least 1 dose of Study Drug.

PK Population: All patients who are randomized and receive at least 1 dose of ION-682884.

10.4. Definition of Baseline

For analyses purposes, baseline laboratory assessments including PD will be defined as the average of all non-missing pre-dose assessments.

For the purposes of safety monitoring and the stopping rules (see Section 8.5), baseline is defined as: average of the pre-dose test closest to Day 1 and Day 1.

Baseline for all other assessments will be defined as the last non-missing value prior to the first dose of the Study Drug.

10.5. Interim Analysis and Multiplicity

An Interim Analysis is not planned for this study.

In order to control the Type I error rate at 0.05 at final analyses for the testing of primary and secondary efficacy endpoints, a hierarchical ranking strategy in a pre-defined testing sequence for primary and secondary endpoints will be applied. Details will be provided in the Statistical Analysis Plan (SAP).

10.6. Planned Methods of Analysis

Descriptive summary statistics including n, mean, median, standard deviation, standard error, interquartile range (25th percentile, 75th percentile), and range (minimum, maximum) for continuous variables, and counts and percentages for categorical variables will be used to summarize most data by treatment group. Where appropriate, p-values will be reported.

All primary and secondary endpoints will be assessed on the Full Analysis Set. All safety assessments will be performed on the Safety Set.

10.6.1. Demographic and Baseline Characteristics

Demographic and Baseline characteristics will be summarized using descriptive statistics by treatment group. Patient randomization will be summarized by treatment group. The patient disposition will be summarized. All patients enrolled will be included in a summary of patient disposition.

10.6.2. Efficacy Analysis

10.6.2.1. Primary Endpoint Analysis

The primary analysis is to compare the composite endpoint of CV-death and recurrent CV clinical events after up to 140 weeks of exposure using the Andersen-Gill method (Andersen & Gill 1982). The treatment effect will be estimated with a robust variance estimator using Lin, Wei, Yang and Ying (LWYY) method (Lin *et al.* 2000).

Patients, who discontinue for transplantation (i.e., heart transplantation and combined heart and liver transplantation) or for implantation of an intermediate-or long-term mechanical circulatory support (i.e., ventricular assist device, total artificial heart), will be treated as CV death in the primary efficacy analysis.

Details of analysis method and sensitivity analyses for primary endpoints will be described in the SAP.

10.6.2.2. Secondary Endpoints Analysis

The primary analyses for secondary endpoints will be performed as follows:

- Change from Baseline at Week 140 Study Visit in the 6MWT will be compared between ION-682884 and placebo treatment groups in the FAS using an ANCOVA model including treatment, stratification factors, baseline value, and the interaction of current tafamidis use (Y/N) at Baseline times treatment as covariates. Patients with missing endpoint will be imputed using the multiple imputation method based on a pattern mixture model. More details for missing data imputation and analysis method will be described in the SAP.
- Change from Baseline at Week 140 Study Visit in the KCCQ overall summary score will also be analyzed in the same way as for change from Baseline at Week 140 Study Visit in the 6MWT.
- Recurrent CV clinical events will be analyzed using the similar analysis model as for the primary analysis.
- All-cause death will be analyzed using a Cox proportional hazards model with treatment, stratification factors as factors, and log-transformed baseline NT-proBNP as covariates.
- Composite endpoint of CV-death and recurrent CV clinical events up to Week 140 in subgroup of patients treated with tafamidis at Baseline will be analyzed using the same way as primary analysis.
- CV death will be analyzed in the same way as for all-cause death.

Details of analysis methods and sensitivity analyses for secondary endpoints will be described in the SAP.

10.6.2.3. Safety Assessments Analysis

Treatment duration and amount of ION-682884 or placebo received will be summarized by treatment group, as well as reasons for any withdrawals from ION-682884.

All treatment-emergent adverse events (TEAE) and SAEs, including deaths, AESI and OAEIs (Other Adverse Events of Interest, described in Statistical Analysis Plan) will be summarized for each treatment group using the MedDRA™ coding system, by system organ class, preferred term, relationship to ION-682884 or placebo, and severity. Narratives of deaths, SAEs, including early withdrawals from ION-682884 or placebo and from study due to AE, will also be provided.

Laboratory tests to ensure patient safety including chemistry panel, complete blood count (CBC) with differential, coagulation panel, etc., will be summarized by study visits for each treatment group. These safety variables will also be presented as change and percent change from Baseline over time after ION-682884 or placebo administration, as appropriate.

Vital sign and ECG measures will be tabulated. In addition, the number of patients who experience abnormalities in clinical laboratory evaluations will be summarized.

10.6.2.4. Exploratory Endpoints Analysis

Details of the exploratory analyses will be described in the SAP.

10.6.3. Exploratory Additional Analyses

10.6.3.1. Pharmacokinetic Analysis

Plasma trough and post-treatment concentrations of ION-682884 will be summarized using descriptive statistics, with and without stratification by patient level ADA data. Additional details regarding the PK analysis will be described in the SAP.

10.6.3.2. Immunogenicity Analysis

Anti-drug Antibody (ADA) will be determined in a multi-tiered approach that includes screening, confirmation, and titration (if confirmed positive) assays at Baseline, during treatment, and at the end of Follow-up Period for all patients (receiving either ION-682884 or placebo) in the study. Immunogenicity results (screen positive/negative, confirmed positive/negative or not evaluable, and when applicable, titer of ADA) before, during, and after treatment with ION-682884 or placebo (i.e., sample ADA status) will be listed. Patient ADA status (positive/negative or not evaluable) for all evaluable patients, along with the study day that the first positive immunogenicity status emerged (T_{first}), i.e., onset of treatment-emergent ADA, the last positive immunogenicity status observed (T_{last}), the last ADA sample collection day, and peak titer if applicable, will be listed by their treatment and study day. Patients with positive ADA status may be further classified (where applicable) as being either ‘persistent’, ‘transient’, or ‘not determinable’, if deemed appropriate.

Additionally, the sample and patient immunogenicity incidence (number) and incidence rate (percent) will be summarized as the total number and percent of evaluated patients with antibody

negative, positive, and unknown status by treatment and dose. Furthermore, onset, titer over time, and peak titer of the ADA response, if applicable, will be summarized as median, quartiles (25% and 75%), and range by treatment and dose.

Potential effect of immunogenicity on efficacy and safety will be evaluated. Additional details regarding the IM data analysis will be described in the SAP.

11. INVESTIGATOR'S REGULATORY OBLIGATIONS

11.1. Informed Consent

The written informed consent document should be prepared in the language(s) of the potential patient population, based on an English version provided by the Sponsor.

Before a patient's participation in the trial, the Investigator is responsible for obtaining written informed consent from the patient after adequate explanation of the aims, methods, anticipated benefits, and potential hazards of the study and before any protocol-specific screening procedures or any Study Drug (ION-682884 or placebo) are administered. The patient must be given sufficient time to consider whether to participate in the study.

A legally authorized representative (LAR) signature is not allowed for the inclusion of the subject in the Screening Period, however, allowed for reconsent when applicable.

The acquisition of informed consent and the patient's agreement or refusal to notify his/her primary care physician should be documented in the patient's medical records and the informed consent form should be signed and personally dated by the patient and by the person who conducted the informed consent discussion (not necessarily an Investigator). The original signed informed consent form should be retained in the Study Master File and in any other locations required by institutional policy, and a copy of the signed consent form should be provided to the patient.

11.2. Ethical Conduct of the Study

All applicable regulations and guidelines of current Good Clinical Practice (GCP) as well as the demands of national drug and data protection laws and other applicable regulatory requirements must be followed. These include, US Code of Federal Regulations, Title 21, and Regulation (EU) No 536/2014.

11.3. Independent Ethics Committee/Institutional Review Board

A copy of the protocol, proposed informed consent form, other written patient information, and any proposed advertising material must be submitted to the IEC/IRB for written approval.
A copy of the written approval of the protocol and informed consent form must be received by the Sponsor before recruitment of patients into the study and shipment of Study Drug. A copy of the written approval of any other items/materials that must be approved by the Study Center or IEC/IRB must also be received by the Sponsor before recruitment of patients into the study and shipment of Study Drug. The IB must be submitted to the IEC/IRB for acknowledgement.

The Investigator must submit to and, where necessary, obtain approval from the IEC/IRB, for all subsequent protocol amendments and changes to the informed consent document. The Investigator should notify the IEC/IRB of deviations from the protocol in accordance with ICH GCP and/or per the IEC/IRB reporting requirements. The Investigator should also notify the IEC/IRB of SAEs occurring at the Study Center and other AE reports received from the Sponsor in accordance with local procedures.

The Investigator will be responsible for obtaining annual IEC/IRB approval/renewal throughout the duration of the study. Copies of the Investigator's reports, all IEC/IRB submissions and the IEC/IRB continuance of approval must be sent to the Sponsor or designee.

11.4. Patient Confidentiality

The Investigator must ensure that the patient's confidentiality is maintained. On the case report forms or other documents submitted to the Sponsor, patients should be identified by a patient identification number only. Documents that are not for submission to the Sponsor (e.g., signed informed consent forms) should be kept in strict confidence by the Investigator.

In compliance with Federal and local regulations/ICH GCP Guidelines, it is required that the Investigator and institution permit authorized representatives of the company, of the regulatory agency(s), and the IEC/IRB direct access to review the patient's original medical records for verification of study-related procedures and data. Direct access includes examining, analyzing, verifying, and reproducing any records and reports that are important to the evaluation of the study. The Investigator is obligated to inform and obtain the consent of the patient to permit named representatives to have access to his/her study-related records without violating the confidentiality of the patient.

Sponsor, and companies working with Sponsor, will implement technical and organizational measures for protection of personal information, and will ensure appropriate protections are in place when transferring personal data in accordance with applicable law (e.g., GDPR, LGPD). For example, transfers from the European Union will comply with GDPR, including appropriate contractual arrangements such as standard contractual clauses. Furthermore, study data is pseudonymized and the pseudonymization key is maintained at the clinical sites and not provided to the Sponsor, which will mitigate potential adverse effects from a data breach.

11.5. Publication Policy

Upon completion of the study, a CSR will be submitted to the IEC/IRB as appropriate per country guidelines. In addition, another complete and accurate summary of the results will be made publicly available at relevant country clinical trial websites. The CSR, the summary, and any other summaries that may be generated will adhere to accepted guidelines for clinical reporting, e.g., will include any inconclusive or negative results.

12. ADMINISTRATIVE AND LEGAL OBLIGATIONS

12.1. Protocol Amendments

Protocol amendments must be made only with the prior approval of the Sponsor. Agreement from the Investigator must be obtained for all protocol amendments and amendments to the informed consent document. The regulatory authority and IEC/IRB must be informed of all amendments and give approval for any amendments likely to affect the safety of the patients or the conduct of the trial. The Investigator **must** send a copy of the approval letter from the IEC/IRB to the Sponsor.

12.2. Study Termination

The Sponsor reserves the right to terminate the study. The Investigator reserves the right to terminate their participation in the study, according to the terms of the site contract. The Investigator should notify the IEC/IRB in writing of the trial's completion or early termination and send a copy of the notification to the Sponsor.

12.3. Study Documentation and Storage

An eCRF utilizing an Electronic Data Capture (EDC) application will be used for this study.

The Investigator should ensure that all appropriately qualified persons to whom he/she has delegated trial duties are recorded on a Sponsor-approved Delegation of Site Responsibilities Form.

Source documents are original documents, data, and records from which the patient's case report form data are obtained. These include but are not limited to hospital records, clinical and office charts, laboratory and pharmacy records, diaries, imaging, and correspondence.

The Investigator and Study Center staff are responsible for maintaining a comprehensive and centralized filing system of all study-related (essential) documentation in accordance with ICH GCP, suitable for inspection at any time by representatives from the Sponsor and/or applicable regulatory authorities. Elements should include:

- Patient files containing completed case report forms, informed consents, and supporting copies of source documentation
- Study files containing the protocol with all amendments, IB, copies of pre-study documentation and all correspondence to and from the IEC/IRB and the Sponsor
- If drug supplies are maintained at the Study Center, proof of receipt, Study Drug Product Accountability Record, Return of Study Drug Product for Destruction, final Study Drug product reconciliation, and all drug-related correspondence

In addition, all original source documents supporting entries in the case report forms must be maintained and be readily available.

No study document should be destroyed without prior written agreement between the Sponsor and the Investigator. Should the Investigator wish to assign the study records to another party or move them to another location, he/she must notify the Sponsor.

12.4. Study Monitoring

The Sponsor representative and regulatory authority inspectors are responsible for contacting and visiting the Investigator for the purpose of inspecting the facilities and, upon request, inspecting the various records of the trial (e.g., case report forms and other pertinent data) provided that patient confidentiality is respected.

The study monitor is responsible for inspecting the case report forms at regular intervals throughout the study to verify adherence to the protocol; completeness, accuracy, and consistency of the data; and adherence to local regulations on the conduct of clinical research. The study monitor should have access to patient medical records and other study-related records needed to verify the entries on the case report forms. The access to these records may include viewing your medical records remotely, from a location outside of the study center (where allowed locally).

The Sponsor, Investigator and patients will be kept blinded to laboratory results which can jeopardize the blinding of treatment allocation, alter Investigator's clinical practice, or are part of study endpoints to avoid bias in the conduct of the study. The Investigators will have unblinded access to safety monitoring laboratory results and can request locally any test, exam, or procedure, per clinical practice, to provide the best care for the patient (with the exception of prealbumin assessment).

The Investigator agrees to cooperate with the monitor to ensure that any problems detected in the course of these monitoring visits, including delays in completing case report forms, are resolved.

In accordance with ICH GCP and the Sponsor's audit plans, this study may be selected for audit by representatives from the Sponsor's Clinical Quality Assurance Department. Inspection of Study Center facilities (e.g., pharmacy, drug storage areas, laboratories) and review of study-related records will occur to evaluate the trial conduct and compliance with the protocol, ICH GCP, and applicable regulatory requirements.

To ensure the quality of clinical data a clinical data management review will be performed on patient data received by the Sponsor. During this review, patient data will be checked for consistency, omissions, and any apparent discrepancies. In addition, the data will be reviewed for adherence to the protocol and GCP. To resolve any questions arising from the clinical data management review process, data queries and/or Study Center notifications will be sent to the Study Center for completion and return to Sponsor.

Periodically during the study, and at the end of the study, the Principal Investigator will sign and date the indicated places on the case report form. These signatures will indicate that the Principal Investigator inspected or reviewed the data on the case report form, the data queries, and the Study Center notifications, and agrees with the content.

12.5. Language

Case report forms must be completed in English. Generic names and trade names are acceptable for concomitant medications. Combination medications should be recorded using their trade name.

All written information and other material to be used by patients and investigative staff must use vocabulary and language that are clearly understood.

12.6. Compensation for Injury

The Sponsor maintains appropriate insurance coverage for clinical trials and will follow applicable local compensation laws. Patients will be treated and/or compensated for any study-related illness/injury in accordance with the information provided in the Compensation for Injury section of the Informed Consent document.

13. REFERENCES

Adams, D., Gonzalez-Duarte, A., O'Riordan, W. D., … Suhr, O. B. (2018). Patisiran, an RNAi Therapeutic, for Hereditary Transthyretin Amyloidosis. *N Engl J Med*, **379**(1), 11–21.

Adams, D., Hawkins, P. N., & Polydefkis, M. (2018). Oligonucleotide Drugs for Transthyretin Amyloidosis. *N Engl J Med*, **379**(21), 2086.

Adams, D., Samuel, D., Goulon-Goeau, C., … Said, G. (2000). The course and prognostic factors of familial amyloid polyneuropathy after liver transplantation. *Brain*, **123 (Pt 7)**, 1495–504.

Adams, D., Suhr, O. B., Hund, E., … Coelho, T. (2016). First European consensus for diagnosis, management, and treatment of transthyretin familial amyloid polyneuropathy. *Curr Opin Neurol*, **29 Suppl 1**(NA;), S14-26.

Andersen, P. K., & Gill, R. D. (1982). Cox's regression model for counting processes: a large sample study. *The Annals of Statistics*, **10**, 1100–1120.

Benson, M. D., Dasgupta, N. R., Rissing, S. M., Smith, J., & Feigenbaum, H. (2017). Safety and efficacy of a TTR specific antisense oligonucleotide in patients with transthyretin amyloid cardiomyopathy. *Amyloid*, **24**(4), 217–223.

Benson, M. D., Kluve-Beckerman, B., Zeldenrust, S. R., … Sloop, K. W. (2006). Targeted suppression of an amyloidogenic transthyretin with antisense oligonucleotides. *Muscle & Nerve*, **33**(5), 609–618.

Benson, M. D., Waddington-Cruz, M., Berk, J. L., … Coelho, T. (2018). Inotersen treatment for patients with hereditary transthyretin Amyloidosis. *N Engl J Med*, **379**(1), 22–31.

Berk, J. L., Suhr, O. B., Obici, L., … Dyck, P. J. (2013). Repurposing diflunisal for familial amyloid polyneuropathy: a randomized clinical trial. *Jama*, **310**(24), 2658–67.

Buxbaum, J. N., & Reixach, N. (2009). Transthyretin: the servant of many masters. *Cell Mol Life Sci*, **66**(19), 3095–101.

Castaño, A., Drachman, B. M., Judge, D., & Maurer, M. S. (2015). Natural history and therapy of TTR-cardiac amyloidosis: emerging disease-modifying therapies from organ transplantation to stabilizer and silencer drugs. *Heart Failure Reviews*, **20**(2), 163–178.

Coelho, T., Maia, L. F., Silva, A. M. da, … Grogan, D. R. (2012). Tafamidis for transthyretin familial amyloid polyneuropathy: a randomized, controlled trial. *Neurology*, **79**(8), 785–92.

Conceição, I., González-Duarte, A., Obici, L., … Amass, L. (2016). "Red-flag" symptom clusters in transthyretin familial amyloid polyneuropathy. *Journal of the Peripheral Nervous System*, **21**(1), 5–9.

Cornwell, G. G., Murdoch, W. L., Kyle, R. A., Westermark, P., & Pitkänen, P. (1983). Frequency and distribution of senile cardiovascular amyloid A clinicopathologic correlation. *The American Journal of Medicine*, **75**(4), 618–623.

D'Ambrosio, D. N., Clugston, R. D., & Blaner, W. S. (2011). Vitamin A metabolism: an update. *Nutrients*, **3**(1), 63–103.

Dungu, J. N., Anderson, L. J., Whelan, C. J., & Hawkins, P. N. (2012). Cardiac transthyretin amyloidosis. *Heart*, **98**(21), 1546–54.

Falk, R. H. (2011). Cardiac Amyloidosis. *Circulation*, **124**(9), 1079–1085.

FDA. (2005). Guidance for industry: estimating the maximum safe starting dose in initial clinical trials for therapeutics in adult healthy volunteers. Retrieved from https://www.fda.gov/downloads/Drugs/GuidanceComplianceRegulatoryInformation/Guidances/UCM078932.pdf.

Fleming, C. E., Nunes, A. F., & Sousa, M. M. (2009). Transthyretin: More than meets the eye. *Progress in Neurobiology*, **89**(3), 266–276.

Geary, Yu, R. Z., Watanabe, T., … Levin, A. A. (2003). Pharmacokinetics of a Tumor Necrosis Factor-α Phosphorothioate 2′-O-(2-Methoxyethyl Modified Antisense Oligonucleotide: Comparison Across Species. *Drug Metabolism and Disposition*, **31**(11), 1419–1428.

Gillmore, J. D., Damy, T., Fontana, M., … Hawkins, P. N. (2018). A new staging system for cardiac transthyretin amyloidosis. *Eur Heart J*, **39**(30), 2799–2806.

Gillmore, J. D., Maurer, M. S., Falk, R. H., … Hawkins, P. N. (2016). Nonbiopsy Diagnosis of Cardiac Transthyretin Amyloidosis. *Circulation*, **133**(24), 2404–2412.

González-López, E., Gallego-Delgado, M., Guzzo-Merello, G., … Garcia-Pavia, P. (2015). Wild-type transthyretin amyloidosis as a cause of heart failure with preserved ejection fraction. *European Heart Journal*, **36**(38), 2585–2594.

Grogan, M., Scott, C. G., Kyle, R. A., … Dispenzieri, A. (2016). Natural History of Wild-Type Transthyretin Cardiac Amyloidosis and Risk Stratification Using a Novel Staging System. *Journal of the American College of Cardiology*, **68**(10), 1014–1020.

Henry, Kim, T. W., Kramer-Stickland, K., Zanardi, T. A., Fey, R. A., & Levin, A. A. (2008). Chapter 12: Toxicologic Properties of 2 -O-Methoxyethyl Chimeric Antisense Inhibitors in Animals and Man. *Antisense Drug Technology: Principles, Strategies, and Application (CRC Press: Boca Raton)*, 327–363.

Henry, Narayanan, P., Shen, L., Bhanot, S., Younis, H. S., & Burel, S. A. (2017). Assessment of the Effects of 2’-Methoxyethyl Antisense Oligonucleotides on Platelet Count in Cynomolgus Nonhuman Primates. *Nucleic Acid Ther*, **27**(4), 197–208.

Herlenius, G., Wilczek, H. E., Larsson, M., & Ericzon, B. G. (2004). Ten years of international experience with liver transplantation for familial amyloidotic polyneuropathy: results from the Familial Amyloidotic Polyneuropathy World Transplant Registry. *Transplantation*, **77**(1), 64–71.

Hicks, K. A., Mahaffey, K. W., Mehran, R., … Trials, I. S. D. C. for C. (2018). 2017 Cardiovascular and Stroke Endpoint Definitions for Clinical Trials. *Circulation*, **137**(9), 961–972.

Inker, L. A., Schmid, C. H., Tighiouart, H., … Investigators, C.-E. (2012). Estimating Glomerular Filtration Rate from Serum Creatinine and Cystatin C. *The New England Journal of Medicine*, **367**(1), 20–29.

Kwoh. (2008). *Antisense Drug Technology Principles, Strategies, and Applications: Chapter 13: An Overview of the Clinical Safety Experience of First- and Second- Generation Antisense Oligonucleotides*. (S. T. Crooke, Ed.), 2nd edn, Boca Raton: CRC Press. doi:10.1201/9780849387951.ch13

Levey, A. S., Stevens, L. A., Schmid, C. H., … Ckd, E. P. I. (2009). A new equation to estimate glomerular filtration rate. *Ann Intern Med*, **150**(9), 604–12.

Levin, A. A., Yu, R. Z., & Geary, R. S. (2008). Chapter 7: Basic Principles of the Pharmacokinetics of Antisense Oligonucleotide Drugs, 2nd edn, Boca Raton: CRC Press, pp. 183–25.

Liepnieks, J. J., & Benson, M. D. (2007). Progression of cardiac amyloid deposition in hereditary transthyretin amyloidosis patients after liver transplantation. *Amyloid*, **14**(4), 277–282.

Liepnieks, J. J., Zhang, L. Q., & Benson, M. D. (2010). Progression of transthyretin amyloid neuropathy after liver transplantation. *Neurology*, **75**(4), 324–327.

Lin, D. Y., Wei, L., Yang, I., & Ying, Z. (2000). Semiparametric regression for the mean and rate functions of recurrent events. *Journal of the Royal Statistical Society Series B*, **62**(4), 711–730.

Maurer, M. S., Schwartz, J. H., Gundapaneni, B., … Investigators, A.-A. S. (2018). Tafamidis Treatment for Patients with Transthyretin Amyloid Cardiomyopathy. *N Engl J Med*, **379**(11), 1007-1016.

Ng, B., Connors, L. H., Davidoff, R., Skinner, M., & Falk, R. H. (2005). Senile Systemic Amyloidosis Presenting With Heart Failure: A Comparison With Light Chain–Associated Amyloidosis. *Archives of Internal Medicine*, **165**(12), 1425–1429.

Plante-Bordeneuve, V., & Said, G. (2011). Familial amyloid polyneuropathy. *Lancet Neurol*, **10**(12), 1086–97.

Prakash, T. P., Graham, M. J., Yu, J., … Seth, P. P. (2014). Targeted delivery of antisense oligonucleotides to hepatocytes using triantennary N-acetyl galactosamine improves potency 10-fold in mice. *Nucleic Acids Research*, **42**(13), 8796–8807.

Provan, D., Stasi, R., Newland, A. C., … Kuter, D. J. (2010). International consensus report on the investigation and management of primary immune thrombocytopenia. *Blood*, **115**(2), 168–86.

Quintas, A., Vaz, D. C., Cardoso, I., Saraiva, M. J., & Brito, R. M. (2001). Tetramer dissociation and monomer partial unfolding precedes protofibril formation in amyloidogenic transthyretin variants. *J Biol Chem*, **276**(29), 27207–13.

Rapezzi, C., Merlini, G., Quarta, C. C., … Perlini, S. (2009). Systemic cardiac amyloidoses: disease profiles and clinical courses of the 3 main types. *Circulation*, **120**(13), 1203–12.

Ruberg, F. L., Maurer, M. S., Judge, D. P., … Grogan, D. R. (2012). Prospective evaluation of the morbidity and mortality of wild-type and V122I mutant transthyretin amyloid cardiomyopathy: the Transthyretin Amyloidosis Cardiac Study (TRACS). *Am Heart J*, **164**(2), 222-228.e1.

Schulman, S., & Kearon, C. (2005). Definition of major bleeding in clinical investigations of antihemostatic medicinal products in non-surgical patients. *Journal of Thrombosis and Haemostasis*, **3**(4), 692–694.

Stangou, A. J., & Hawkins, P. N. (2004). Liver transplantation in transthyretin-related familial amyloid polyneuropathy. *Current Opinion in Neurology*, **17**(5), 615–620.

Stewart, M., Lenderking, W., & Loftus, J. (2013). Evaluating the quality of life and burden of illness in an ultra-rare disease in the U.S.: transthyretin familial amyloid polyneuropathy (TTR-FAP) patients & caregivers. Abstract presented at: the IXth International Syposium on Familial Amyloidotic Polyneuropathy (ISFAP); November 10-13, 2013; Rio de Janeiro, Brazil. Abstract E16.

Yazaki, M., Mitsuhashi, S., Tokuda, T., … Ikeda, S. I. (2007). Progressive wild-type transthyretin deposition after liver transplantation preferentially occurs onto myocardium in FAP patients. *Am J Transplant*, **7**(1), 235–42.

Yazaki, M., Tokuda, T., Nakamura, A., … Ikeda, S. (2000). Cardiac amyloid in patients with familial amyloid polyneuropathy consists of abundant wild-type transthyretin. *Biochem Biophys Res Commun*, **274**(3), 702–6.

Yu, Kim, Hong, Watanabe, Gaus, & Geary. (2007). Cross-Species Pharmacokinetic Comparison from Mouse to Man of a Second-Generation Antisense Oligonucleotide, ISIS 301012, Targeting Human Apolipoprotein B-100. *Drug Metabolism and Disposition*, **35**(3), 460–468.

Yu, R. Z., Graham, M. J., Post, N., … Henry, S. (2016). Disposition and Pharmacology of a GalNAc3-conjugated ASO Targeting Human Lipoprotein (a) in Mice. *Molecular Therapy - Nucleic Acids*, **5**(5), e317.

Yu, R. Z., Gunawan, R., Post, N., … Henry, S. (2016). Disposition and Pharmacokinetics of a GalNAc3-Conjugated Antisense Oligonucleotide Targeting Human Lipoprotein (a) in Monkeys. *Mol Ther Nucleic Acids*, **5**(5), 372–380.

14. APPENDICES

APPENDIX A. SCHEDULE OF PROCEDURES

Shaded columns denote Mandatory in-Clinic visits

D1 to Week 89

	Screening	D1 Visit	Treatment Period (Day 1 to End of Week 140)																					
**Study Week**	N/A	1	5	9	13	17	21	25	29	33	37	41	45	49	53	57	61	65	69	73	77	81	85	89
**Study Day**	-70	1	29	57	85	113	141	169	197	225	253	281	309	337	365	393	421	449	477	505	533	561	589	617
**Mandatory in-Clinic Visit**[20]	X	X			X			X			X			X			X			X			X	
**Site/Clinic or Home Visit**			X	X		X	X		X	X		X	X		X	X		X	X		X	X		X
**Mandatory Discussion (face-to-face or phone call)***			X	X		X	X																	
**Visit Window (± days)**	N/A	N/A	4	4	4	4	4	4	4	4	4	4	4	4	4	4	4	4	4	4	4	4	4	4
Informed Consent	X																							
Randomization[1]		X[1]																						
Eligibility criteria		X																						
Medical History	X																							
Concomitant Medications	X	X	→																					
Adverse Events	X	X	→																					
TTR Genotyping[2]	X																							
Biopsy and/or Scintigraphy[2]	X																							
SPEP, UPEP, serum free light chain ratio[3]	X																							
Vital Signs, weight, height[4]	X	X			X			X			X			X			X			X			X	
Physical Exam[5]	X	X			X			X			X			X			X			X			X	

Appendix A Schedule of Procedures (Continued)

D1 to Week 89 (continued)

	**Screening**	**D1 Visit**	**Treatment Period (Day 1 to End of Week 140)**																					
**Study Week**	**N/A**	**1**	**5**	**9**	**13**	**17**	**21**	**25**	**29**	**33**	**37**	**41**	**45**	**49**	**53**	**57**	**61**	**65**	**69**	**73**	**77**	**81**	**85**	**89**
**Study Day**	**-70**	**1**	**29**	**57**	**85**	**113**	**141**	**169**	**197**	**225**	**253**	**281**	**309**	**337**	**365**	**393**	**421**	**449**	**477**	**505**	**533**	**561**	**589**	**617**
**Mandatory in-Clinic Visit[20]**	**X**	**X**			**X**			**X**			**X**			**X**			**X**			**X**			**X**	
**Site/Clinic or Home Visit**			**X**	**X**		**X**	**X**		**X**	**X**		**X**	**X**		**X**	**X**		**X**	**X**		**X**	**X**		**X**
**Mandatory Discussion (face-to-face or phone call)***			**X**	**X**		**X**	**X**																	
**Visit Window (± days)**	**N/A**	**N/A**	**4**	**4**	**4**	**4**	**4**	**4**	**4**	**4**	**4**	**4**	**4**	**4**	**4**	**4**	**4**	**4**	**4**	**4**	**4**	**4**	**4**	**4**
Single ECG 12-Lead[6]	**X**				**X**			**X**						**X**						**X**				
HIV, Hepatitis B and C	**X**																							
FSH[7]	**X**																							
Pregnancy Test[8]	**X**	**X**	**X**	**X**	**X**	**X**	**X**	**X**			**X**			**X**			**X**			**X**			**X**	
Chemistry panel, hematology, urinalysis	**X**	**X**			**X**			**X**			**X**			**X**			**X**			**X**			**X**	
Abbreviated monitoring panel[9]			**X**	**X**		**X**	**X**																	
Thyroid panel, PT, INR, aPTT	**X**							**X**						**X**										
PD panel, PK sampling Immunogenicity Testing[10]		**X**			**X**			**X**			**X**			**X**			**X**			**X**			**X**	
Cardiac Biomarkers	**X**							**X**						**X**						**X**				
Retinol/Retinyl Palmitate[11]	**X**							**X**						**X**										
Ocular questionnaire		**X**			**X**			**X**			**X**			**X**			**X**			**X**			**X**	
Archived Serum Sample[12]		**X**			**X**			**X**			**X**			**X**			**X**			**X**			**X**	

Appendix A Schedule of Procedures (Continued)

D1 to Week 89 (Continued)

	**Screening**	**D1 Visit**	**Treatment Period (Day 1 to End of Week 140)**																					
**Study Week**	**N/A**	**1**	**5**	**9**	**13**	**17**	**21**	**25**	**29**	**33**	**37**	**41**	**45**	**49**	**53**	**57**	**61**	**65**	**69**	**73**	**77**	**81**	**85**	**89**
**Study Day**	**-70**	**1**	**29**	**57**	**85**	**113**	**141**	**169**	**197**	**225**	**253**	**281**	**309**	**337**	**365**	**393**	**421**	**449**	**477**	**505**	**533**	**561**	**589**	**617**
**Mandatory in-Clinic Visit[20]**	**X**	**X**			**X**			**X**			**X**			**X**			**X**			**X**			**X**	
**Site/Clinic or Home Visit**			**X**	**X**		**X**	**X**		**X**	**X**		**X**	**X**		**X**	**X**		**X**	**X**		**X**	**X**		**X**
**Mandatory Discussion (face-to-face or phone call)***			**X**	**X**		**X**	**X**																	
**Visit Window (± days)**	**N/A**	**N/A**	**4**	**4**	**4**	**4**	**4**	**4**	**4**	**4**	**4**	**4**	**4**	**4**	**4**	**4**	**4**	**4**	**4**	**4**	**4**	**4**	**4**	**4**
6-Minute Walk Test	**X**							**X**									**X**							
Echocardiogram[13]	**X**																**X**							
KCCQ, SF-36, EQ-5D-5L		**X**						**X**						**X**			**X**			**X**				
Norfolk-QoL-DN		**X**									**X**						**X**						**X**	
PGIS, PGIC[14]		**X**															**X**							
NYHA class	**X**							**X**			**X**			**X**			**X**			**X**			**X**	
Karnofsky perf. Status	**X**																**X**							
PND, COMPASS-31, LLF, 10-meter walk test[14]		**X**									**X**						**X**						**X**	
Study Drug administration[16]		**X**	**X**	**X**	**X**	**X**	**X**	**X**	**X**	**X**	**X**	**X**	**X**	**X**	**X**	**X**	**X**	**X**	**X**	**X**	**X**	**X**	**X**	**X**
Study Drug return and dosing review[17]					**X**			**X**			**X**			**X**			**X**			**X**			**X**	

Appendix A Schedule of Procedures (Continued)

Week 93 to End of Study

	Treatment Period (Day 1 to End of Week 140)													Post-Treatment Evaluation Period[18]		Early Termination Visit [19]	Study Primary Completion Date[21]
**Study Week**	93	97	101	105	109	113	117	121	125	129	133	137	140	150	160	n/a	n/a
**Study Day**	645	673	701	729	757	785	813	841	869	897	925	953	980	1050	1120	n/a	n/a
**Mandatory in-Clinic Visit[20]**		X			X			X			X		X	X	X	X	
**Site/Clinic or Home Visit**	X		X	X		X	X		X	X		X					
**Visit Window (± days)**	4	4	4	4	4	4	4	4	4	4	4	4	7	7	7	n/a	
Vital Signs, weight, height[4]		X			X			X			X		X	X	X	X	
Physical Exam[5]		X			X			X			X		X	X	X	X	
Concomitant Medications	→																
Adverse Events	→																
Single ECG 12-Lead[6]		X						X					X		X	X	
Pregnancy Test[8]		X			X			X			X		X	X	X	X	
Chemistry panel, hematology, urinalysis		X			X			X			X		X	X	X	X	
Abbreviated monitoring panel[9]																	
Thyroid panel, PT, INR, aPTT		X						X					X		X	X	

Appendix A Schedule of Procedures (Continued)

Week 93 to End of Study (Continued)

	Treatment Period (Day 1 to End of Week 140)													Post-Treatment Evaluation Period[18]		Early Termination Visit [19]	Study Primary Completion Date[21]
**Study Week**	93	97	101	105	109	113	117	121	125	129	133	137	140	150	160	n/a	n/a
**Study Day**	645	673	701	729	757	785	813	841	869	897	925	953	980	1050	1120	n/a	n/a
**Mandatory in-Clinic Visit[20]**		X			X			X			X		X	X	X	X	
**Site/Clinic or Home Visit**	X		X	X		X	X		X	X		X					
**Visit Window (± days)**	4	4	4	4	4	4	4	4	4	4	4	4	7	7	7	n/a	
PD panel, PK sampling, Immunogenicity Testing[10]		X			X			X			X		X	X	X	X	
Cardiac Biomarkers		X						X					X		X	X	
Retinol/Retinyl Palmitate[11]		X											X		X	X	
Ocular questionnaire		X			X			X			X		X	X	X	X	
Archived Serum Sample[12]		X			X			X			X		X		X	X	
6-Minute Walk Test		X						X					X		X	X	
Echocardiogram[13]		X											X			X	
KCCQ, SF-36 Survey, EQ-5D-5L		X						X					X		X	X	
Norfolk-QoL-DN								X					X		X	X	
PGIS, PGIC[14]													X		X	X	

Appendix A Schedule of Procedures (Continued)

Week 93 to End of Study (Continued)

	Treatment Period (Day 1 to End of Week 140)													Post-Treatment Evaluation Period[18]		Early Termination Visit[19]	Study Primary Completion Date[21]
**Study Week**	93	97	101	105	109	113	117	121	125	129	133	137	140	150	160	n/a	n/a
**Study Day**	645	673	701	729	757	785	813	841	869	897	925	953	980	1050	1120	n/a	n/a
**Mandatory in-Clinic Visit[20]**		X			X			X			X		X	X	X	X	
**Site/Clinic or Home Visit**	X		X	X		X	X		X	X		X					
**Visit Window (±-days)**	4	4	4	4	4	4	4	4	4	4	4	4	7	7	7	n/a	
NYHA class		X			X			X			X		X	X	X	X	
Karnofsky perf. Status													X		X	X	
PND, COMPASS-31, LLF, 10-meter walk test[15]								X					X		X	X	
Study Drug administration[16]	X	X	X	X	X	X	X	X	X	X	X	X					
Study Drug return and dosing review[17]		X			X			X			X		X			X	
Vital Status[22]													X		X		X

Legend

*The visits at Weeks 5, 9, 17, and 21 are not mandatory in-clinic visits, therefore, for those visits, when not conducted in-clinic, it is required (mandatory) that the PI, or designee contact (i.e., phone call, video call) the patient to discuss the occurrence of AEs/SAEs, changes in concomitant medications, and their general health.

1 Patients must have all screening eligibility procedures conducted and reviewed by the Investigator to be randomized. Randomization and assessment of some baseline measurements occur during D1 (the completion of procedures can take more than one day). First Study Drug dosing should be the last procedure of D1.

2 For determination of patient eligibility, only if not conducted before, or appropriate documentation is not available. For biopsy, location per local practice.

3 SPEP, UPEP with immunofixation, serum free light chain ratio.

4 Blood Pressure (BP), heart rate (HR), respiratory rate (RR), temperature. Height to be measured at Screening visit only.

5 Full physical exam to be given at Screening and abbreviated physical exam to be given during Treatment and Follow-up Period as indicated to assess changes from Screening.

6 Single 12-lead ECG to be performed with local machine. Results to be recorded in the specific eCRF section.

7 For confirmation of menopause (non-childbearing potential) in woman less than 55 years old and with > 12 months of amenorrhea, and not surgically sterile, per inclusion criterion.

8 For women who are not surgically sterile or post-menopausal per protocol (childbearing potential) only. Serum test.

9 The abbreviate monitoring panel includes total bilirubin; direct (conjugated) bilirubin; indirect (unconjugated) bilirubin; ALT; AST; creatinine; potassium, sodium, BUN, eGFR (CKD-EPI formula); red blood cells; hemoglobin; hematocrit; mean corpuscular volume (MCV); mean corpuscular hemoglobin (MCH); mean corpuscular hemoglobin concentration (MCHC); RDW; MPV; Platelets; White blood cells; UPCR. These assessments will be conducted every 4 weeks (± 4 days) for the initial 6 months and every 3 months (± 4 days), thereafter during the Treatment Period. Patients will have their samples collected at the study site or at home by a home health provider (if approved locally), or the use of a laboratory local to the patient upon Investigator approval. If the abbreviate monitoring panel results are uninterpretable (e.g., due to clumping, hemolysis or quantity not sufficient) or missing, a repeat blood or urine specimen should be re-drawn as soon as possible and ensure interpretable values on retest before dosing. If there is suspicion of EDTA mediated platelet clumping, a repeat platelet count should be collected in a sodium citrate tube (or an alternative tube) as soon as possible.

10 Blood collection should be conducted before the Study Drug dosing.

11 Requires fasting for at least 8 hours.

12 Collection expected unless prohibited by local regulations. Stored as per the laboratory manual for follow up exploration of laboratory findings and/or AE (e.g., measurement of cytokine and/or chemokine levels, measurement of additional markers of kidney function, measurement of antibodies, etc.) in this or subsequent clinical studies of ION-682884. Not for genetic research.

13 The time window for the echocardiogram is ± 10 days from the visit date. This does not apply for Screening Period.

14 PGIC not to be assessed at D1 (no baseline).

15 COMPASS-31 should be administered ONLY to patients with hereditary ATTR-CM. LLF, 10-meter walk test and PND Score to be assessed ONLY in patients with hereditary ATTR-CM (the time window for LLF and 10-meter walk test assessment is ± 10 days from the visit date. This does not apply for Screening Period).

Appendix A Schedule of Procedures (Continued)

Legend (Continued)

16 Study Drug administration should occur after conducting all procedures scheduled for that day. Study Drug can be administered in the clinic during clinic visits per protocol or administered at home by the patient/caregiver/home health provider after clinic visits. For Study Drug dosing occurring out of a scheduled mandatory site/clinic study visit the Study Drug administration can be supported by either Study Center personnel (in which case additional clinic visits will occur) or at home by the patient/caregiver/home health provider. The dosing visit has a flexibility of ± 4 days. Delayed dosing can occur up to +18 days from the scheduled visit date if delays to access reliable results from safety monitoring occur. Study Drug administrations must be at least 10 days apart.

17 Study Drug dosing review of prior administrations (patient diary).

18 The Post Treatment Evaluation Period is 20 weeks with study visits at Week 150 and Week 160 (or 10 and 20 weeks after after last dose respectively, if the last dose occurs prior to Week137). Patients enrolled in the Open Label Extension Study (when available) are not required to complete the Post-Treatment Evaluation Period.

19 For patients who withdraw full consent for study participation, the completion of the Early Termination Visit should be encouraged. It does not apply for Study Drug pauses or discontinuation for reasons other than full consent withdrawal.

20 Questionnaires (KCCQ, SF-36 Survey, EQ-5D-5L, Norfolk QOL-DN, PGIS, PGIC, COMPASS-31 and ocular questionnaire), may be completed remotely, for patients who are unable to attend a mandatory in-clinic visit.

21 Study Primary Completion Date is defined as the date of the last patient's last visit of the Treatment Period. Vital status collection may be initiated 12 weeks prior to the anticipated Study Primary Completion Date. The vital status assessment for Week 160 may be performed at the time of regulatory and IRB/IEC approval of this protocol amendment if the approval is granted after the Week 160 time point.

22 For patients who discontinue early, vital status will be assessed by the time the patient was expected to reach the end of their Treatment Period (Week 140) and the end of their Post Treatment Evaluation Period (Week 160). For patients who complete Week 140 and enroll in CS12, vital status will be assessed in CS12 by the time that corresponds to when the patient would have reached Week 160 had they remained in CS2. Vital status of all study participants up to the Study Primary Completion Date, except for those patients that receive dosing in CS12, are currently active in CS2, or have a known date of death, will be determined where possible and regardless of the patient's End-of Study status. If the patient has withdrawn consent, vital status may be obtained through indirect means if permitted by local laws of jurisdiction.

APPENDIX B. LIST OF LABORATORY ANALYTES

Based on emerging data from this or future studies, additional tests not listed below may be performed on stored samples to better characterize the profile of ION-682884 or other similar oligonucleotides.

Chemistry Panel

- Sodium
- Potassium
- Chloride
- Bicarbonate
- Total protein
- Albumin
- Calcium
- Magnesium
- Phosphorus
- Glucose (non-fasting)
- BUN
- Creatinine
- eGFR (CKD-EPI formula)
- Uric Acid
- Total bilirubin
- Direct (conjugated) bilirubin
- Indirect (unconjugated) bilirubin
- ALT
- AST
- Alkaline phosphatase
- Creatine kinase
- GGT
- Cystatin C

Pregnancy Test

- Serum βhCG

Screening Tests

- Hepatitis B surface antigen
- Hepatitis C antibody and HCV RNA
- HIV antibody and HIV RNA
- FSH
- Serum βhCG
- SPEP, UPEP with immunofixation
- Serum free light chain ratio
- TTR genotyping

PD Panel

- TTR*
- RBP4*

Cardiac Biomarkers

- NT-ProBNP*
- hs cTnT*
- CK-MB*
- Soluble ST2*
- Galectin-3*

Pharmacokinetics[1]

- ION-682884 in plasma*

Immunogenicity

- Anti-ION-682884 antibodies*

Hematology

- HbA1c
- Red blood cells
- Hemoglobin
- Hematocrit
- MCV, MCH, MCHC
- RDW, MPV
- Platelets
- White blood cells
- WBC Differential (% and absolute)
  - Neutrophils
  - Eosinophils
  - Basophils
  - Lymphocytes
  - Monocytes

Thyroid Panel

- TSH
- Free T4
- Total T3

Coagulation

- aPTT (sec)
- PT (sec)
- INR

Other tests

- Retinol/Retinyl Palmitate (fasting)*

Urinalysis

- Color
- Appearance
- Specific gravity
- pH
- UPCR
- UACR
- Protein
- Blood
- Ketones
- Urobilinogen
- Glucose
- Bilirubin
- Leukocyte esterase
- Nitrate
- Microscopic examination[2]

Abbreviated Monitoring Panel Includes:

- Total bilirubin; direct (conjugated) bilirubin; indirect (unconjugated) bilirubin; ALT; AST; creatinine; potassium, sodium, BUN, eGFR (CKD-EPI formula);
- Red blood cells; hemoglobin; hematocrit; MCV; MCH; MCHC; RDW; MPV; Platelets; White blood cells
- UPCR

* After Study Drug dosing on Study Day 1, these results will be kept blinded to participants, Sponsor and Investigators until the end of the study.

[1] Plasma PK samples may also be used for profiling of drug binding proteins, bioanalytical method validation purposes, stability assessments, metabolite assessments, immunogenicity testing (or possibly for purposes of immunogenicity assay development and/or validation) or to assess other actions of ION-682884 with plasma constituents. An early restricted unblinded population pharmacokinetic access (ERUPA) Team has been appointed for this study. The ERUPA Team is a group of independent Programmers and Scientists who will have early unblinded access to the pharmacokinetic data, and certain laboratory and demographic data that is not associated with the efficacy and safety outcomes of the study. The ERUPA Team will use this information to develop a population pharmacokinetic (PopPK) model before database lock. A charter will document this process and list of individuals in the ERUPA team.

[2] Will be performed on abnormal findings unless otherwise specified.

APPENDIX C. GRADING SCALE FOR ADVERSE EVENTS RELATING TO LABORATORY ABNORMALITIES

The following grading recommendations for AE relating to lab test abnormalities and AE at the injection site are based upon the Common Terminology Criteria for Adverse Events (CTCAE) Version 5.0, November 2017

Adverse Event	Mild	Moderate	Severe
	**Hematology**		
aPTT prolonged	>ULN - 1.5 x ULN	>1.5 - 2.5 x ULN	>2.5 x ULN; bleeding
Eosinophils increased[†]	>ULN and >Baseline	-	Steroids Initiated
Fibrinogen decreased	<1.0 - 0.75 x LLN; if abnormal, <25% decrease from baseline	<0.75 - 0.5 x LLN; if abnormal, 25 - <50% decrease from baseline	<0.5 x LLN; if abnormal, ≥50% decrease from baseline
Hemoglobin decreased (Anemia)	Hemoglobin (Hgb) <LLN - 10.0 g/dL; <LLN - 6.2 mmol/L; <LLN - 100 g/L	Hgb <10.0 - 8.0 g/dL; <6.2 - 4.9 mmol/L; <100 - 80g/L	Hgb <8.0 g/dL; <4.9 mmol/L; <80 g/L; transfusion indicated
Hemoglobin increased**	Increase in >0 - 2 g/dL above ULN or above baseline if baseline is above ULN	Increase in >2 - 4 g/dL above ULN or above baseline if baseline is above ULN	Increase in >4 g/dL above ULN or above baseline if baseline is above ULN
INR increased	>1.2 - 1.5; >1 - 1.5 times above baseline if on anticoagulation	>1.5 - 2.5; >1.5 - 2.5 x baseline if on anticoagulation; monitoring only indicated	>2.5; >2.5 x baseline if on anticoagulation; dose adjustment indicated
Lymphocyte count decreased	<LLN - 800/mm^3; <LLN - 0.8 x 10^9/L	<800 - 500/mm^3; <0.8 - 0.5 x 10^9 /L	<500 /mm^3; <0.5 x 10^9 /L
Lymphocyte count increased	-	>4000/mm^3 - 20,000/mm^3	>20,000/mm^3
Neutrophil count decreased	<LLN - 1500/mm^3; <LLN - 1.5 x 10^9 /L	<1500 - 1000/mm^3; <1.5 - 1.0 x 10^9 /L	<1000/mm^3; <1.0 x 10^9 /L
Platelet count decreased	<LLN - 75,000/mm^3; <LLN - 75.0 x 10^9 /L	<75,000 - 50,000/mm^3; <75.0 - 50.0 x 10^9 /L	<50,000/mm^3; <50.0 x 10^9 /L
White blood cell decreased	<LLN - 3000/mm^3; <LLN - 3.0 x 10^9 /L	<3000 - 2000/mm^3; <3.0 - 2.0 x 10^9 /L	<2000/mm^3; <2.0 x 10^9 /L
	**Chemistry**		
Acidosis	pH <normal, but >=7.3	-	pH <7.3
Alanine aminotransferase increased	>ULN - 3.0 x ULN if baseline normal 1.5 - 3.0 x baseline if baseline abnormal	>3.0 - 5.0 x ULN if baseline normal >3.0 - 5.0 x baseline if baseline abnormal	>5.0 x ULN if baseline normal >5.0 x baseline if baseline abnormal
Alkaline phosphatase increased	>ULN - 2.5 x ULN if baseline normal 2.0 - 2.5 x baseline if baseline abnormal	>2.5 - 5.0 x ULN if baseline normal >2.5 - 5.0 x baseline if baseline abnormal	>5.0 x ULN if baseline normal >5.0 x baseline if baseline was abnormal
Alkalosis	pH >normal, but ≤7.5	-	pH >7.5
Aspartate aminotransferase increased	>ULN - 3.0 x ULN if baseline normal 1.5 - 3.0 x baseline if baseline abnormal	>3.0 - 5.0 x ULN if baseline normal >3.0 - 5.0 x baseline if baseline abnormal	>5.0 x ULN if baseline normal >5.0 x baseline if baseline abnormal
Blood bilirubin increased	>ULN - 1.5 x ULN if baseline normal >1.0 - 1.5 x baseline if baseline abnormal	>1.5 - 3.0 x ULN if baseline normal >1.5 - 3.0 x baseline if baseline abnormal	>3.0 x ULN if baseline normal >3.0 x baseline if baseline abnormal
Cardiac troponin I increased	Levels above the upper limit of normal and below the level of myocardial infarction as defined by the manufacturer	-	Levels consistent with myocardial infarction as defined by the manufacturer

APPENDIX C. GRADING SCALE FOR ADVERSE EVENTS RELATING TO LABORATORY ABNORMALITIES (CONTINUED)

Adverse Event	Mild	Moderate	Severe
Cardiac troponin T increased	Levels above the upper limit of normal and below the level of myocardial infarction as defined by the manufacturer	-	Levels consistent with myocardial infarction as defined by the manufacturer
CD4 lymphocytes decreased	<LLN - 500/mm^3;<br><LLN - 0.5 x 10^9 /L	<500 - 200/mm^3;<br><0.5 - 0.2 x 10^9 /L	<200/mm^3;<br><0.2 x 10^9 /L
CPK increased*	>ULN - <6 ULN	6 - 10 x ULN	>10 x ULN
Creatinine increased**	>ULN - 1.5 x ULN if baseline normal<br>> 1.0 - 1.5 x baseline if baseline abnormal	>1.5 - 3.0 x ULN if baseline normal<br>>1.5 - 3.0 x baseline if baseline abnormal	>3.0 x ULN if baseline normal<br>>3.0 x baseline if baseline abnormal
GGT increased	>ULN - 2.5 x ULN if baseline normal<br>2.0 - 2.5 x baseline if baseline abnormal	>2.5 - 5.0 x ULN if baseline normal<br>>2.5 - 5.0 x baseline if baseline abnormal	>5.0 x ULN if baseline normal<br>>5.0 x baseline if baseline abnormal
Hypercalcemia	Corrected serum calcium of >ULN - 11.5 mg/dL; >ULN - 2.9 mmol/L; Ionized calcium >ULN - 1.5 mmol/L	Corrected serum calcium of >11.5 - 12.5 mg/dL; >2.9 - 3.1 mmol/L; Ionized calcium >1.5 - 1.6 mmol/L; symptomatic	Corrected serum calcium of >12.5 mg/dL; >3.1 mmol/L; Ionized calcium >1.6 mmol/L; hospitalization indicated
Hyperglycemia††	Fasting glucose value ≥126 mg/dL (7.0 mmol/L)	Change in daily management to maintain fasting blood glucose <126 mg/dL (7.0 mmol/L); e.g. addition of oral antiglycemic agent; workup for diabetes	Insulin therapy initiated; hospitalization indicated
Hyperkalemia	>ULN - 5.5 mmol/L	>5.5 - 6.0 mmol/L; intervention initiated	>6.0; hospitalization indicated
Hypermagnesemia	>ULN - 3.0 mg/dL;<br>>ULN - 1.23 mmol/L	-	>3.0 mg/dL;<br>>1.23 mmol/L
Hypernatremia	>ULN - 150 mmol/L	>150 - 155 mmol/L; intervention initiated	>155 mmol/L; hospitalization indicated
Hyperphosphatemia	Laboratory finding only and intervention not indicated	Noninvasive intervention indicated	Severe or medically significant but not immediately life-threatening; hospitalization or prolongation of existing hospitalization indicated
Hyperuricemia	>ULN without physiologic consequences	-	>ULN with physiologic consequences
Hypoalbuminemia	<LLN - 3 g/dL;<br><LLN - 30 g/L	<3 - 2 g/dL;<br><30 - 20 g/L	<2 g/dL;<br><20 g/L
Hypocalcemia	Corrected serum calcium of <LLN - 8.0 mg/dL; <LLN - 2.0 mmol/L; Ionized calcium <LLN - 1.0 mmol/L	Corrected serum calcium of <8.0 - 7.0 mg/dL; <2.0 - 1.75 mmol/L; Ionized calcium <1.0 - 0.9 mmol/L; symptomatic	Corrected serum calcium of <7.0 mg/dL; <1.75 mmol/L; Ionized calcium <0.9 mmol/L; hospitalization indicated
Hypoglycemia‡	≥54 mg/dL - <70 mg/dL<br>≥3.0 mmol/L - <3.9 mmol/L	<54 mg/dL (3.0 mmol/L) AND no assistance required to actively administer carbohydrates, glucagon, or take other corrective actions	Requires assistance of another person to actively administer carbohydrates, glucagon, or take other corrective actions
Hypokalemia	<LLN - 3.0 mmol/L	symptomatic with <LLN - 3.0 mmol/L; intervention indicated	<3.0 mmol/L; hospitalization indicated
Hypomagnesemia	<LLN - 1.2 mg/dL;<br><LLN - 0.5 mmol/L	<1.2 - 0.9 mg/dL;<br><0.5 - 0.4 mmol/L	<0.9 mg/dL;<br><0.4 mmol/L
Hyponatremia	<LLN - 130 mmol/L	125-129 mmol/L and asymptomatic	125-129 mmol/L symptomatic;<br>120-124 mmol/L regardless of symptoms
Hypophosphatemia	Laboratory finding only and intervention not indicated	Oral replacement therapy indicated	Severe or medically significant but not immediately life-threatening; hospitalization or prolongation of existing hospitalization indicated
Lipase increased	>ULN - 1.5 x ULN	>1.5 - 2.0 x ULN;<br>>2.0 - 5.0 x ULN and asymptomatic	>2.0 x ULN with signs or symptoms
Serum amylase increased	>ULN - 1.5 x ULN	>1.5 - 2.0 x ULN;<br>>2.0 - 5.0 x ULN and asymptomatic	>2.0 x ULN with signs or symptoms

APPENDIX C. GRADING SCALE FOR ADVERSE EVENTS RELATING TO LABORATORY ABNORMALITIES (CONTINUED)

Adverse Event	Mild	Moderate	Severe
**Urine**			
Proteinuria			
Adults	1+ proteinuria;<br>urinary protein ≥ULN - <1.0 g/24 hrs	2+ and 3+ proteinuria;<br>urinary protein 1.0 - 3.4 g/24 hrs;	4+ proteinuria;<br>Urinary protein ≥3.5 g/24 hrs;
Children	-	Urine P/C (Protein/Creatinine) ratio 0.5 - 1.9	Urine P/C >1.9
Hematuria	Asymptomatic; clinical or diagnostic observations only; intervention not indicated	Symptomatic; urinary catheter or bladder irrigation indicated	Gross hematuria; transfusion, IV medications or hospitalization indicated; elective invasive intervention indicated
**Adverse Events at the Injection Site**			
Adverse events at the injection site[**]	An event at the injection site (e.g. erythema, tenderness, itching) that is easily tolerated by the subject and does not affect the subject's usual daily activities	- Persistent (>24 hours) pain, phlebitis or edema; OR<br>- Lipodystrophy, hair growth or alopecia, OR<br>- Prolonged (>1 month) hypo/hyperpigmentation	- Ulceration or necrosis; severe tissue damage; operative intervention indicated, OR<br>- Any event at the injection site that is incapacitating

[†] Grading for this parameter is derived from the Toxicity Grading Scale for Healthy Adult and Adolescent Volunteers Enrolled in Preventive Vaccine Clinical Trials, Sept 2007

[*] Grading for this parameter is derived from the Division of AIDS (DAIDS) Table for Grading the Severity of Adult and Pediatric Adverse Events Version 2.0, Nov 2014

[††] Modified for consistency with ADA "Standards of Medical Care in Diabetes - 2018" Diabetes Care 2018;41(Suppl. 1):S13–S27. https://doi.org/10.2337/dc18-S002

[‡] Modified for consistency with ADA "Glycemic Targets: Standards of Medical Care in Diabetes - 2018", Diabetes Care 2018;41(Suppl. 1):S55–S64. https://doi.org/10.2337/dc18-S006

[**] Adapted from the original CTCAE V5.0 scale

ADDENDUM 1 CANADA SPECIFIC PROTOCOL

This addendum describes changes to Study ION-682884-CS2 specific to the conduct of the study in Canada.

The Renal Stopping Rule described in Section 8.6.2 is as follows:

Section 8.6.2 Stopping Rules for Renal Function Test Results

Dosing should be stopped temporarily in the event of an alteration observed and confirmed, for any of the 4 criteria below:

1. **A decrease in eGFR > 50% (eGFR is calculated using the CKD-EPI equation)**
2. **eGFR < 45 mL/min/1.73 m^2 (if Baseline eGFR > 60 mL/min/1.73 m^2)**
3. **eGFR < 30 mL/min/1.73 m^2 (if Baseline eGFR ≤ 60 mL/min/1.73 m^2)**
   a. **Criteria #1, 2, 3: these criteria do not apply for events to which the Investigator considers not related to Study Drug administration. A discussion with Medical Monitor is needed and a reassessment of the event must be conducted if the relationship with Study Drug changes, per Investigator's opinion. Such events must be followed and managed as AE/SAE per Section 9.4**
4. **Proteinuria: Random UPCR > 5 × Baseline AND > 1500 mg/g; or absolute UPCR value ≥ 2000 mg/g (confirmed by a repeat random spot UPCR ≥ 2000 mg/g or a 24-hr UPCR ≥ 2000 mg/g)**

If Study Drug administration is paused per the rules above, once eGFR returns to the prior value, UPCR decreases to below 1000 mg/g, or the underlying cause of the decline in renal function is corrected, dosing may be reinitiated. In the case of UPCR of 2000 mg/g or higher, further evaluation for renal disease should be performed. If acute glomerulonephritis is confirmed, treatment should be permanently discontinued.

Dosing of a patient will be stopped permanently if urinalysis or renal blood tests confirm any of the following values in the absence of an alternative explanation agreed by a consulting nephrologist:

- **urine protein is > 2 g (in 24-hour urine)**
- **creatinine clearance < 45 mL/min/1.73 m^2 (if Baseline CKD-EPI > 60 mL/min/1.73 m^2)**

Irrespective of whether the stopping rule is confirmed or not, the follow-up schedule and frequency of renal function monitoring after the initial event will be determined by the Study Medical Monitor in consultation with the Investigator and a renal consult will be requested.

Protocol History – Canada:

Amendment 2:	25 November 2019
Amendment 3:	24 March 2020
Amendment 4:	9 March 2021
Amendment 5:	2 August 2021
Amendment 6:	10 May 2022
Amendment 7:	11 October 2022

ADDENDUM 2 JAPAN SPECIFIC PROTOCOL

This addendum describes changes to Study ION-682884-CS2 specific to the conduct of the study in Japan.

Patients are stratified at Randomization based on **2 stratification factors: NYHA Classification (I and II vs. III) and 6MWT distance (6MWT ≤ 350 meters vs > 350 meters)**

Entry criteria:

Inclusion 2. **20** to 90 years of age (inclusive)

Inclusion 5. **Amyloid deposits in cardiac or non-cardiac tissue confirmed by Congo Red (or equivalent) staining**.

Section 3.4.1 Screening

3.4.1. If a scintigraphy (99mTc -3,3-diphosphono-1,2-propanodicarboxylic acid [DPD-Tc], 99m Tc-pyrophosphate [PYP-Tc]), or 99mTc-hydroxymethylene-diphosphonate [HMDP-Tc]), new or historical is available, please submit it for central reading, for recording purposes.

Section 6.4.1. Contraception Requirements

Progestogen-only is removed from the list of highly effective female contraception methods.

Appendix A. Schedule of Procedures

- Additional Week 3 study visit
- All study visits are mandatory in-clinic visits
- Vital signs and physical exam are conducted at all study visits

Protocol History – Japan:

Original Protocol:	28 August 2019
Amendment 1:	26 October 2020
Amendment 2:	21 January 2021
Amendment 3:	29 March 2022
Amendment 4:	18 May 2022
Amendment 5:	25 May 2022
Amendment 6:	14 October 2022

ADDENDUM 3 UNITED KINGDOM SPECIFIC PROTOCOL

This addendum describes changes to Study ION-682884-CS2 specific to the conduct of the study in United Kingdom.

Excludes patients with:

- **Known diagnosis of moderate or severe chronic obstructive pulmonary disease (as defined by the American Thoracic Society/European Respiratory Society) or severe restrictive lung disease, in the opinion of the Investigator**

The Renal Stopping Rule described in Section 8.6.2 is as follows:

Section 8.6.2 Stopping Rules for Renal Function Test Results

Dosing should be stopped temporarily in the event of an alteration observed and confirmed, for any of the 4 criteria below:

1. **A decrease in eGFR > 50% (eGFR is calculated using the CKD-EPI equation)**
2. **eGFR < 45 mL/min/1.73 m^2 (if Baseline eGFR > 60 mL/min/1.73 m^2)**
3. **eGFR < 30 mL/min/1.73 m^2 (if Baseline eGFR ≤ 60 mL/min/1.73 m^2)**
    a. **Criteria #1, 2, 3: these criteria do not apply for events to which the Investigator considers not related to Study Drug administration. A discussion with Medical Monitor is needed and a reassessment of the event must be conducted if the relationship with Study Drug changes, per Investigator's opinion. Such events must be followed and managed as AE/SAE per Section 9.4**
4. **Proteinuria: Random UPCR > 5 × Baseline AND > 1500 mg/g; or absolute UPCR value ≥ 2000 mg/g (confirmed by a repeat random spot UPCR ≥ 2000 mg/g or a 24-hr UPCR ≥ 2000 mg/g)**

If Study Drug administration is paused per the rules above, once eGFR returns to the prior value, UPCR decreases to below 1000 mg/g, or the underlying cause of the decline in renal function is corrected, dosing may be reinitiated. In the case of UPCR of 2000 mg/g or higher, further evaluation for renal disease should be performed. If acute glomerulonephritis is confirmed, treatment should be permanently discontinued.

Dosing of a patient will be stopped permanently if urinalysis or renal blood tests confirm any of the following values in the absence of an alternative explanation agreed by a consulting nephrologist:

- **urine protein is > 2 g (in 24-hour urine)**
- **creatinine clearance < 45 mL/min/1.73 m^2 (if Baseline CKD-EPI > 60 mL/min/1.73 m^2)**

Irrespective of whether the stopping rule is confirmed or not, the follow-up schedule and frequency of renal function monitoring after the initial event will be determined by the Study Medical Monitor in consultation with the Investigator and a renal consult will be requested.

Protocol History – United Kingdom:

Amendment 2:	18 December 2019
Amendment 3:	19 March 2020
Amendment 4:	19 March 2021
Amendment 5:	25 August 2021
Amendment 6:	10 November 2021
Amendment 7:	6 June 2022
Amendment 8:	13 October 2022

Protocol

Version:	1
Version Date:	22 Apr 2025
Title:	682884-CS2-Protocol-Amendment 10 - A Phase 3 Global, Double-Blind, Randomized, Placebo-Controlled Study to Evaluate the Efficacy and Safety of ION-682884 in Patients with Transthyretin-Mediated Amyloid Cardiomyopathy (ATTR-

APPROVALS:

[redacted] 22-Apr-2025 23:21:47 GMT+0000

PAREXEL International

IONIS PHARMACEUTICALS, INC.

ION-682884-CS2

A Phase 3 Global, Double-Blind, Randomized, Placebo-Controlled Study to Evaluate the Efficacy and Safety of ION-682884 in Patients with Transthyretin-Mediated Amyloid Cardiomyopathy (ATTR CM)

Statistical Analysis Plan

Version: 6.0

PAREXEL Project Number: [redacted]

SPONSOR SIGNATURE PAGE

Approved by:

See electronic signature and date at the end of the document

[redacted]
Ionis Pharmaceuticals, Inc.
2855 Gazelle Court
Carlsbad, CA 92010

See electronic signature and date at the end of the document

[redacted]
Ionis Pharmaceuticals, Inc.
2855 Gazelle Court
Carlsbad, CA 92010

See electronic signature and date at the end of the document

[redacted]
Clinical Development
Ionis Pharmaceuticals, Inc.
2855 Gazelle Court
Carlsbad, CA 92010

See electronic signature and date at the end of the document

[redacted]
Ionis Pharmaceuticals, Inc.
2855 Gazelle Court
Carlsbad, CA 92010

PAREXEL SIGNATURE PAGE

Signature(s) below confirm that the Statistical Analysis Plan was developed in accordance with SOP-GDO-WW-019 and that it is approved for release.

This document has been approved and signed electronically on the final page by the following:

	Signatory
**Author**	[redacted]

TABLE OF CONTENTS

SPONSOR SIGNATURE PAGE 2
PAREXEL SIGNATURE PAGE 3
REVISION HISTORY 7
LIST OF ABBREVIATIONS 12
1. INTRODUCTION 15
2. STUDY OBJECTIVES 15
2.1. Primary Objectives 15
2.2. Secondary Objectives 15
2.3. Safety Objective 16
2.4. Exploratory Objectives 16
3. INVESTIGATIONAL PLAN 17
3.1. Overall Study Design and Plan 17
3.2. Study Endpoints 17
3.2.1. Primary Endpoint 17
3.2.2. Secondary Endpoints 18
3.2.3. Safety Variables 18
3.2.4. Exploratory Endpoints 18
4. STATISTICAL METHODS 19
4.1. Data Management and Quality Assurance 19
4.1.1. Laboratory Data 20
4.1.2. Pharmacokinetics and Immunogenicity Data 20
4.1.3. Echocardiogram Data 21
4.1.4. Electrocardiogram Data 21
4.1.5. Adjudicated Data 21
4.2. General Presentation Considerations 21
4.2.1. Baseline 22
4.2.2. Definitions and Computational Formulas 22
4.2.3. Analysis Visit Windows 23
4.3. Software 24
4.4. Study Subjects 25
4.4.1. Disposition of Subjects 25

4.4.2. Protocol Deviations 25
4.5. Analysis Sets 25
4.5.1. Screened Patients 25
4.5.2. Randomized Patients 25
4.5.3. Full Analysis Set 26
4.5.4. Safety Set 26
4.5.5. PK Set 26
4.6. Demographic and Other Baseline Characteristics 26
4.7. Concomitant Medications 27
4.8. Treatment Compliance 28
4.9. Efficacy Evaluation 28
4.9.1. Estimand for Primary and Secondary Outcomes 28
4.9.2. Analysis and Data Conventions 31
4.9.2.1. Adjustments for Covariates 37
4.9.2.2. Handling of Dropouts or Missing Data 37
4.9.2.3. Multiple Imputation Methodology for 6WMT or KCCQ 37
4.9.2.4. Data Summary Plan 38
4.9.2.5. Interim Analysis 39
4.9.2.6. Multiple Comparisons/Multiplicity 39
4.9.2.7. Examination of Subgroups 40
4.9.3. Primary Efficacy Variable 40
4.9.4. Sensitivity, Supplementary, and Exploratory Analyses of Primary Endpoint 42
4.9.5. Secondary Efficacy Variables 44
4.9.6. Sensitivity and Exploratory Analyses of Secondary Endpoints 45
4.9.6.1. Change from Baseline to Week 140 in 6MWT and KCCQ 45
4.9.6.2. CV Clinical Events 46
4.9.6.3. Time to Event Endpoints (All-cause Death and CV Death) 47
4.10. Safety Evaluation 47
4.10.1. Adverse Events 47
4.10.2. Other Significant Adverse Events 49
4.10.2.1. Adverse Events of Special Interest (AESI) 49
4.10.2.2. Other Adverse Events of Interest 50

4.10.3. Clinical Laboratory Evaluation....52
4.10.3.1. Hepatobiliary Laboratory Abnormalities....52
4.10.3.2. Platelets....53
4.10.3.3. Renal Parameters....54
4.10.4. Vital Signs, Physical Findings and Other Observations Related to Safety....55
4.10.5. 12-Lead Electrocardiograms (12-Lead ECG)....56
4.10.6. Ocular Questionnaire....56
4.10.7. Safety Monitoring (Data and Safety Monitoring Board [DSMB])....56
4.11. Exploratory Analyses....57
4.11.1. Exploratory Endpoints Analysis....57
4.11.2. Immunogenicity (IM) Analysis....59
4.11.2.1. Sample Level ADA Data....59
4.11.2.2. Subject Level ADA Data....59
4.11.2.3. Evaluation of IM Impact on PK, PD, Efficacy and Safety....62
4.12. Other Analyses....62
4.12.1. Pharmacokinetics....62
4.13. Determination of Sample Size....63
4.14. Changes in the Conduct of the Study or Planned Analysis....63
5. REFERENCES....64
6. APPENDIX....65
Appendix 6.1 Kansas City Cardiomyopathy Questionnaire....65
Appendix 6.2 Composite Autonomic Symptom Score (COMPASS-31)....68
Appendix 6.3 EQ-5D Health State Index Score Derivation Algorithm....72
Appendix 6.4 eGFR Slope Analysis – SP Model....73

LIST OF TABLES

Table 1: KCCQ Domains and Scoring....65
Table 2: Algorithm of KCCQ Scoring....67
Table 3: COMPASS-31 Scores....68
Table 4: Weight of EQ-5D-5L Response by Region....72

REVISION HISTORY

Version No.	Effective Date	Summary of Change(s)
1.0	19NOV2020	New Document
2.0	17MAR2023	To address FDA's comments in Advice/Information Request under IND 139707 dated 14 Jan 2021 and 19 Jan 2023, the SAP is updated to version 2.0 and major changes are summarized as follows.<br>1. Added mathematical details on primary analysis LWYY model.<br>2. Added sensitivity analyses for primary endpoint (joint frailty model, WLW method, Ghosh-Lin method, time to first event, and negative binomial regression).<br>3. Clarified components of the composite primary endpoint are defined as additional secondary endpoints.<br>4. Modified missing data imputation for 6MWT and KCCQ: Missing data after death and missing after transplantation will be multiply imputed using the Jump to Reference (J2R) approach based on the bottom 50% placebo patients.<br>5. Clarified the blinded power estimation for potential early final analysis in section 4.13.<br>Additional major changes include:<br>1. Updated based on protocol amendment, and major changes include number of patients and treatment duration, and interim analysis was removed.<br>2. Added analysis related to Cardiac MRI Sub-study and Scintigraphy sub-study.
3.0	14DEC2023	The major changes are summarized as follows.<br>1. Updated based on protocol amendment, and major changes include adding additional potential early stop timepoints.<br>2. In section 4.9.1.6, added secondary endpoints including CV events, CV death, and all-cause death to hierarchical testing order to control overall type I error.<br>3. In section 4.9.2, clarified primary analysis methods for secondary endpoints.<br>4. In section 4.10.2.1, removed "Ocular adverse events related to vitamin A deficiency", "Thrombocytopenia", and "Renal Impairment" from AESI to be consistent with the protocol.<br>Added "Vitamin A Decreased", "Ocular events potentially related to vitamin A deficiency", "Renal Impairment", "Thrombocytopenia" and "AV Block" to other AE of interest. The previous definition of "Ocular events related to vitamin A deficiency" is split into two

		categories: "Vitamin A Decreased" with SMQ codes related only to low Vitamin A labs, and "Ocular events potentially related to vitamin A deficiency" with SMQ codes related only to ocular events because the prior definition could be met when patients experienced a Vitamin A decrease in the absence of any ocular event. This second category will not include all ocular events, only ocular events that occurred in setting of low Vitamin A levels will be included.
4.0	28APR2025	The major changes are summarized as follows.<br>1. Updated latest protocol amendment and eCRF versions in Section 1.<br>2. Updated Secondary objectives and primary/secondary endpoints in Section 2 and 3 per protocol amendment updates.<br>3. Removed the blinded power assessment in Section 3.1 and 4.13, and updated trial treatment period per Protocol Amendment updates.<br>4. Removed analysis visit window of PK and IM in Section 4.2.3.<br>5. Removed Per Protocol Set from Section 4.5 and related sections per protocol amendment updates.<br>6. Added estimands for primary and secondary endpoints in Section 4.9.1.<br>7. Added baseline log-transformed NT-proBNP as a covariate in Section 4.9.2.1 and related analysis sections.<br>8. Updated multiple imputation method in Section 4.9.2.3.<br>9. Removed sensitivity analyses using Ghosh-Lin and WLW analyses in Section 4.9.4.<br>10. Added tipping point analysis for 6MWT and KCCQ as sensitivity analysis in Section 4.9.6.1.<br>11. Added competing risk analyses for recurrent CV events and time to CV death in Section 4.9.6.2 and 4.9.6.3.<br>12. Added supplementary analysis for components of CV clinical events in Section 4.9.6.2.<br>13. Added Kaplan-Meier analysis for time to first event for AE and lab abnormalities analysis in Section 4.10.<br>14. Added TEAE and death summary by PT term in Section 4.10.1.<br>15. Provided updates to Section 4.10.2.2 Other Adverse Events of Interest based on currently available data and known safety profile of eplontersen.<br>16. Removed analyses based on "confirmed" value for lab summary in Section 4.10.3.<br>17. Added Vital Signs shift from baseline table and vital signs summary table by category Section 4.10.4<br>18. Removed analysis for Cardiac MRI and Scintigraphy sub-studies from Section 4.11.1 and related sections.

		19. Added logistic regression analysis for PGI-C in Section 4.11.1, and detailed analysis and summary for change in NYHA class.<br>20. Added imputation rule for missing baseline ADA data in Section 4.11.2.2.<br>21. Added definitions for Peak ADA Titer, Time to Peak ADA Titer, and Peak ADA Titer Quartiles in Section 4.11.2.
5.0	17FEB2026	1. Update the study CRF version from v10 to v13 in Section 1<br>2. Added echocardiography parameters in Section 3.2.4<br>3. Updated analysis visit window based on latest protocol amendment in Section 4.2.3<br>4. Clarified the summary and added vital status summary for Week 140 and Week 160 in Section 4.4.1<br>5. Added summary for protocol deviations due to COVID-19 related impact in Section 4.4.2<br>6. Added NAC stage summary and details for baseline characteristics in Section 4.6<br>7. Added summary for change of TTR-stabilizer use in Section 4.7<br>8. Added summary for total number of doses received and updated compliance calculation in Section 4.8<br>9. Updated imputation model for missing data Pattern 2 from CIR to CR in Section 4.9.2.3<br>10. Added clarification for the scope of analysis results reported in CSR and addendum to CSR; Removed “excluding Echo related endpoints” in end of treatment lock in Section 4.9.2.4<br>11. Clarified subgroup will be based on eCRF in Section 4.9.2.7<br>12. Added clarifications for censoring rules and details of how to handle data for patients who discontinue treatment in the primary analysis in Section 4.9.3<br>13. In Section 4.9.4: Clarified sensitivity analysis using negative binomial regression will be conducted as supplementary analysis. Added NT-proBNP group (≤ 3000; >3000 ng/L) as an additional covariate in supplementary analysis 1 and 2 to be consistent with primary analysis and added a weighted log-rank test as supplementary analysis.<br>14. Added clarifications for censoring rule and details of how to handle data for time to all-cause death and time to CV death for patients who discontinue treatment in the primary analysis in Section 4.9.5<br>15. Clarified sensitivity analysis using negative binomial regression for frequency of CV clinical events will be conducted as supplementary analysis, clarified that patients who had missing Week 140 data irrespective of

		the reason will be considered a non-responder in the supplementary analysis 2 (responder analysis), and removed sensitivity analysis by including proxy assessments for KCCQ considering there are minimum data collected by proxy assessment in Section 4.9.6<br>16. Added a weighted log-rank test as supplementary analysis for time to all-cause death and time to CV death endpoints.<br>17. In Section 4.10.1: clarified AE occurred on or after the first administration of Study Drug and leading to study drug discontinuation will be deemed as TEAE. Added KM% with 95% CI at week 140 for AE summary. Added partial date imputation for fatal AE end date and death date.<br>18. Added details for summary of AESI and other AE of interest in Section 4.10.2.1 and 4.10.2.2<br>19. Added continuous parameters for urinalysis parameters in Section 4.10.3<br>20. Added KM% and 95% CI at Week 140 summary for lab abnormalities in Section 4.10.3.1<br>21. Added summary of abnormalities of platelet values by analysis visit and mean platelet values over time plot in Section 4.10.3.2<br>22. Clarified eGFR summary, added summary of eGFR over time and incidence of abnormalities of eGFR by CKD-EPI in Section 4.10.3.3<br>23. Removed summary of QTc stopping rule since it is not captured in CRF<br>24. In section 4.11.1: added parameters for echocardiogram; added MMRM model and joint model for eGFR analyses; added log-transformed analysis for hs-cTnT; added subgroup analyses for TTR; added details for how to handle convergency issue of MMRM model; updated analysis model to CMH method and added imputation method for PGI-C, PGI-S, and PND.
6.0	22APR2026	1. Clarified primary endpoint and secondary endpoint (i.e., recurrent CV events) definition in Section 4.9.3 and 4.9.5. For hospitalization for arrhythmia events, the independent CAC will adjudicate hospitalizations for arrhythmia and/or arrhythmia events requiring medical treatment in outpatient facility or ER, and only unscheduled hospitalization or ED > 24 hours for arrhythmia adjudicated events will be counted as CV clinical events in the primary endpoint and secondary endpoint (i.e., recurrent CV clinical events).<br>2. Clarified sensitivity analysis using joint frailty model will be conducted to evaluate the competing risk of all-cause death on recurrent CV events as supplementary analysis for primary analysis in Section 4.9.4.

		3. Added more details for partial data imputation for fatal AE end date and death date in Section 4.10.1, and last dosing date in Section 4.8. Added details for how to handle missing covariate on eCRF in Section 4.9.2.1.

LIST OF ABBREVIATIONS

Abbreviation / Acronym	Definition / Expansion
6MWT	6-minute walk test
%CV	percent Coefficient of Variation
ADA	Patient anti-drug antibody
ADaM	Analysis Data Model
AE	Adverse event
AESI	Adverse event of special interest
ALT	Alanine aminotransferase
ANCOVA	Analysis of covariance
ASO	Antisense oligonucleotide
AST	Aspartate aminotransferase (SGOT)
ATC	Anatomical Therapeutic Chemical
ATTR	Transthyretin-mediated amyloid
ATTR-CM	Transthyretin-mediated amyloid cardiomyopathy
BMI	Body mass index
BP	Bodily pain
CAC	Clinical Adjudication Committee
CBC	Complete blood count
CI	Confidence interval
CICL	Cardiac Imaging Core Laboratory
CKD-EPI	Chronic Kidney Disease Epidemiology Collaboration
CK-MB	Creatine kinase-muscle/brain fraction
CNS	Central Nervous System
COMPASS-31	Composite Autonomic Symptom Scale Score
CR	Copy reference
CRF	Case report form
CRNMB	Clinically relevant, non-major bleeding
CRO	Contract research organization
CTCAE	Common Terminology Criteria for Adverse Events
CV	Cardiovascular
DCRI	Duke Clinical Research Institute
DSMB	Data and safety monitoring board
ECG	Electrocardiogram
ECHO	Echocardiogram
eCRF	Electronic case report form
EDC	Electronic data capture
ED/ER	Emergency Department/Emergency Room
eGFR	Estimated glomerular filtration rate
EOS	End of study
EOT	End of treatment
EQ-5D-5L	European Quality of Life Five Dimension Five Level Scale

Abbreviation / Acronym	Definition / Expansion
ERUPA	Early Restricted Unblinded Population Pharmacokinetic Access
FAS	Full Analysis Set
GH	General health
HF	Heart failure
HFH	Hospitalization for heart failure
HLT	Higher level term
HRQoL	Health-related quality of life
hs-cTnT	High sensitivity cardiac troponin T
IXRS	Interactive voice/internet response system
IM	immunogenicity
INR	International normalized ratio
ION-682884	Antisense inhibitor of transthyretin
IV	Intravenous
IVRT	Isovolumic Relaxation Time
KCCQ	Kansas City Cardiomyopathy Questionnaire
KPS	Karnofsky Performance Status
LLF	Lower limb function
LLOQ	Lower limit of quantification
LV	Left ventricular
LVEDD	Left ventricular end diastolic diameter
LVEDV	Left ventricle end diastolic volume
MB	Major bleeding
MCS	Mental component summary
MedDRA	Medical Dictionary for Regulatory Activities
MH	Mental Health
MI	Myocardial infarction
MMRM	Mixed Effects Model with Repeated Measures
Norfolk QOL-DN	Norfolk quality of life questionnaire-diabetic neuropathy
NT-proBNP	N-terminal prohormone of brain natriuretic peptide
NYHA	New York Heart Association
P25	25th percentile
P75	75th percentile
PCS	Physical component summary
PD	Pharmacodynamic
PF	Physical functioning
PGIC	Patient Global Impression of Change
PGIS	Patient Global Impression of Severity
PK	Pharmacokinetic
PND	Polyneuropathy disability
PRO	Patient reported outcome
PT	Preferred term
QoL	Quality of life

Abbreviation / Acronym	Definition / Expansion
QTc	QT interval corrected for heart rate
QTcB	QTc interval calculated using Bazett's formula
QTcF	QTc interval calculated using Fridericia's formula
RBP4	Retinol binding protein 4
RE	Role-Emotional
RDA	Recommended daily allowance
RP	Role-Physical
SAE	Serious Adverse Event
SAP	Statistical Analysis Plan
SC	Subcutaneous
SDTM	Study Data Tabulation Model
siRNA	Small interfering ribonucleic acid
SD	Standard deviation
SE	Standard error
SF	Social Functioning
SF-36	Short Form (36) Health Survey
SMQ	Standardized MedDRA query
SOC	System Organ Class
SoC	Standard of care
SS	Safety Set
sST2	Soluble Interleukin-1 Receptor Like 1
Study Drug	ION-682884 or Placebo
TEAE	Treatment emergent adverse event
TIA	Transient Ischemic Attack
TTP	thrombotic thrombocytopenic purpura
TTR	Transthyretin
ULN	Upper limit of normal
UPCR	Urine protein/creatinine ratio
VAS	Visual analog scale
VT	Vitality
WHO-DD	World Health Organization-Drug Dictionary

1. INTRODUCTION

This Statistical Analysis Plan (SAP) details the statistical methodology to be used in analyzing study data and outlines the statistical programming specifications for the Tables, Listings and Figures. It describes the variables and populations, anticipated data transformations and manipulations and other details of the analyses not provided in the Clinical Study Protocol.

The analyses described in this SAP are based upon the following study documents:

- Study Protocol Amendment 10 - ROW (April 21, 2025)
- Study Protocol Amendment 9 – EU (April 22, 2025)
- Electronic Case Report Form (eCRF), Version 13.0 (December 8, 2025)

Section 2 indicates the study objectives. Section 3 provides overall study design and study endpoints. Section 4 presents statistical methods including general guidelines to be followed for all analyses, the analysis population, handling of missing data, definition of Baseline and visit window, discussion for the primary/secondary efficacy, sensitivity analyses, details for pharmacokinetic (PK) and determination of sample size.

2. STUDY OBJECTIVES

2.1. Primary Objectives

To evaluate the effect of treatment with ION-682884 compared to placebo up to Week 140 on the composite endpoint of cardiovascular (CV) death and recurrent CV clinical events in patients with transthyretin-mediated amyloid cardiomyopathy (ATTR-CM) receiving available standard of care (SoC).

2.2. Secondary Objectives

- To evaluate the effect of treatment with ION-682884 compared to placebo at Week 140 Study Visit on exercise tolerance (6MWT) in patients with ATTR-CM receiving available SoC
- To evaluate the effect of treatment with ION-682884 compared to placebo at Week 140 Study Visit on patient reported outcome (KCCQ) in patients with ATTR-CM receiving available SoC
- To evaluate the effect of treatment with ION-682884 compared to placebo up to Week 140 on recurrent CV clinical events in patients with ATTR-CM receiving available SoC
- To evaluate the effect of treatment with ION-682884 compared to placebo up to Week 160 on all-cause death in patients with ATTR-CM receiving available SoC (survival follow-up data between Week 140 and up to Week 160 may originate during participation in CS2 post-treatment evaluation period or CS12 open-label extension study)

- To evaluate the effect of treatment with ION-682884 compared to placebo up to Week 140 on all-cause death in patients with ATTR-CM receiving available SoC
- To evaluate the effect of treatment with ION-682884 compared to placebo up to Week 140 on the composite endpoint of CV death and recurrent CV clinical events in patients with ATTR-CM when administered with tafamidis at baseline
- To evaluate the effect of treatment with ION-682884 compared to placebo up to Week 160 on CV death in patients with ATTR-CM receiving available SoC (survival follow-up data between Week 140 and up to Week 160 may originate during participation in CS2 post-treatment evaluation period or CS12 open-label extension study)
- To evaluate the effect of treatment with ION-682884 compared to placebo up to Week 140 on CV death in patients with ATTR-CM receiving available SoC

2.3. Safety Objective

To evaluate the safety and tolerability of ION-682884 compared to placebo in patients with ATTR-CM receiving available SoC.

2.4. Exploratory Objectives

- To evaluate the effect of treatment with ION-682884 compared to placebo on echocardiography parameters of interest
- To evaluate the effect of treatment with ION-682884 compared to placebo on renal function and disease biomarkers
- To evaluate the effect of treatment with ION-682884 compared to placebo on the change from baseline in NYHA class
- To evaluate the effect of treatment with ION-682884 compared to placebo on patient-reported outcome questionnaires and disease scores related to Quality of Life (QoL) and burden of transthyretin-mediated amyloid (ATTR)
- To evaluate the effect of treatment with ION-682884 compared to placebo on Lower Limb Function (LLF) test and 10-meter walk test in patients with hereditary ATTR-CM
- To examine ION-682884 PK in patients with ATTR-CM
- To evaluate the immunogenicity (IM) of ION-682884
- To evaluate the effect of treatment with ION-682884 compared to placebo on mBMI

3. INVESTIGATIONAL PLAN

3.1. Overall Study Design and Plan

This is a Phase 3 multicenter, global, double-blind, randomized, placebo-controlled study to evaluate the efficacy and safety of ION-682884 in patients with hereditary or wild-type ATTR-CM.

Approximately 1400 patients will be randomized 1:1 to receive 45 mg of ION-682884 or placebo SC once every 4 weeks. The Treatment Period is up to 140 weeks. Dose frequency adjustments, including dose interruptions, are allowed for reasons of safety or tolerability, per discussion with Medical Monitor.

Patients will be stratified at Randomization based on New York Heart Association (NYHA) Classification (I and II vs. III), ATTR mutation status (mutated vs. wild-type), 6MWT distance (6MWT ≤ 350 meters vs. > 350 meters), and current treatment with tafamidis (Yes vs. No).

Patients must meet all eligibility criteria to be enrolled in this trial. Concomitant administration of SoC therapies is allowed, per Investigator's discretion, if locally approved, accessible, and consistent with local clinical practice (except antisense oligonucleotide [ASO] and small interfering ribonucleic acid [siRNA] compounds). Patients will also receive daily supplemental doses of the recommended daily allowance [(RDA]) of vitamin A.

The Post-Treatment Evaluation Period comprises a 20-week follow-up period that occurs after the Treatment Period (Week 140 to Week 160). Patients will visit the clinical research site at regular intervals through the 20-week Post-Treatment Evaluation Period. Alternatively, after completion of the Treatment Period, eligible patients may elect to enroll in an OLE study (ION-682884-C12) where they will receive ION-682884 SC every month, pending study approval by the IRB/IEC and appropriate regulatory authority. In this case, patients may not participate in any or only some of the Post-Treatment Evaluation Period.

The vital status (i.e., dead including date of death; alive including last date known alive; unknown) up to the Primary Completion Date of all study participants exposed to study drug will be determined where possible and regardless of the patient's End-of-Study status except those participants active in this study or in the OLE at the time of determination and except those participants already with known date of death.

3.2. Study Endpoints

3.2.1. Primary Endpoint

Composite endpoint of CV death and recurrent CV clinical events up to Week 140.

Cardiovascular clinical events include:

- Hospitalization for myocardial infarction (MI)
- Hospitalization for heart failure (HF)
- Hospitalization for arrhythmias

- Hospitalization for stroke/ Transient Ischemic Attack (TIA)
- HF urgent visits to Emergency Department/Emergency Room (ED/ER) or HF clinics requiring administration of intravenous (IV) diuretics for improvement.

CV deaths include:

- Death with cardiovascular cause or with undetermined cause
- Transplantation (i.e., heart transplantation or combined heart and liver transplantation)
- Intermediate or long-term cardiac mechanical circulatory support (i.e., ventricular assist device or total artificial heart)

All deaths and CV clinical events will be adjudicated by an independent Clinical Adjudication Committee (CAC) using the '2017 Cardiovascular and Stroke Endpoint Definitions for Clinical Trials' (Hicks *et al.* 2018).

3.2.2. Secondary Endpoints

Secondary Endpoints include:

- Changes from baseline in the 6MWT distance at Week 140
- Change from baseline in Kansas City Cardiomyopathy Questionnaire (KCCQ) overall summary score at Week 140
- Recurrent CV clinical events up to Week 140
- All-cause death up to Week 160
- All-cause death up to Week 140
- Primary endpoint in subgroup of patients treated with tafamidis at baseline
- CV death up to Week 160
- CV death up to Week 140

3.2.3. Safety Variables

Safety laboratory tests (e.g., serum creatinine, renal function [eGFR], urine protein/creatinine ratio [UPCR], hematology including platelet count, thyroid function), vital signs, electrocardiogram (ECG) parameters, and Adverse events (AEs).

3.2.4. Exploratory Endpoints

- Changes from baseline up to Week 140 in echocardiography parameters of interest including, but not limited to, Global Longitudinal Strain, Interventricular Septum Thickness, Posterior Wall Thickness, Mean Left Ventricular (LV) Wall Thicknesses, Stroke Volume, Ejection Fraction, Left Ventricular Mass, Left Ventricular End Diastolic Diameter (LVEDD), Left Ventricle End Diastolic Volume (LVEDV), Average E/e' Ratio, Lateral E/e' Ratio, Septal E/e' Ratio, Right Ventricular (RV)

Free Wall Longitudinal Strain, Right Ventricular End Diastolic Area (RVEDA),Tricuspid Annular Plane Systolic Excursion (TAPSE)

- Changes from baseline in renal function (eGFR) and serum biomarkers (N-terminal prohormone of brain natriuretic peptide [NT-proBNP], high sensitivity cardiac troponin T [hs-cTnT], creatine kinase-muscle/brain fraction [CK-MB], Galectin-3, soluble inerleukin-1 receptor like 1 [sST2], Transthyretin [TTR])
- Change from baseline in NYHA class
- Changes from baseline in patient-reported outcome questionnaires and disease scores:
  - Karnofsky performance status (KPS), Short Form (36) Health Survey (SF-36), European Quality of Life Five Dimension Five Level Scale (EQ-5D-5L), Norfolk quality of life questionnaire-diabetic neuropathy (Norfolk QOL-DN), Patient Global Impression of Severity (PGIS), and Patient Global Impression of Change (PGIC) will be assessed periodically in the overall population.
  - Polyneuropathy Disability (PND) and Composite Autonomic Symptom Score (COMPASS-31) will be assessed periodically only in patients with hereditary ATTR-CM.
- Changes from baseline in LLF and 10-meter walk test in patients with hereditary ATTR-CM
- Evaluation of ION-682884 PK in patients with ATTR-CM
- Evaluation of the immunogenicity (IM) of ION-682884
- Change from baseline in mBMI

4. STATISTICAL METHODS

4.1. Data Management and Quality Assurance

ICON has been contracted to provide Data Management services for this study. As part of the contract, ICON is responsible for creating the Electronic Data Capture (EDC) data entry screens, database and edit checks using definitions developed together with Ionis Pharmaceuticals, Inc. ICON is also responsible for the review, data management querying and locking of the database.

Data is single-entered into the EDC system by the investigator site staff. Programmed edit checks (computer logic that checks the validity of the data entered and also prompt for missing data that are expected to be entered) are run and automatic queries are generated. ICON reviews all data for accuracy and validity and generates additional queries in the EDC system when necessary. The data are corrected or an explanation concerning the query is provided in the EDC system. After all data are entered, reviewed, and all queries are resolved, the database is closed and locked. Database closing and locking will be done prior to final analysis.

The Sponsor and all patients, monitors, and Study Center personnel will be blinded to treatment assignment throughout the study. However, if a patient suffers a Serious Adverse Event (SAE) and when knowledge of the treatment assignment would impact the clinical management of the patient, the Investigator will have the ability to unblind the treatment assignment for that patient using the automated interactive voice/internet response system (IXRS). Investigators are encouraged to discuss with Medical Monitor before unblinding, if possible. An unblinded randomization schema will be maintained securely at Ionis-designated vendor.

4.1.1. Laboratory Data

Ionis Pharmaceuticals, Inc. (Ionis) and ICON are responsible for the format of the laboratory electronic data transfers and the transfer schedule. Central laboratory data results are not stored in the EDC system. ICON and Ionis are responsible for the review of the clinical laboratory data. This process involves reviewing the patient and visit identifiers in the central laboratory data results data against the central lab data identifiers collected in the EDC system. Investigator sites have access to the data via lab reports sent directly from the laboratory or through the laboratory's web portal (in which case Investigators only have access to data from their site).

The Sponsor, Investigator and patients will be kept blinded to post-baseline laboratory results (retinol/ retinyl palmitate, TTR, retinol binding protein 4 [RBP4], NT-ProBNP, hs-cTnT, CK-MB, sST2 and galectin-3) which can jeopardize the blinding of treatment allocation, alter Investigator's clinical practice, or are part of study endpoints to avoid bias in the conduct of the study. The Investigators will have unblinded access to safety monitoring laboratory results and can request locally any test, exam, or procedure, per clinical practice, to provide the best care for the patient (with the exception of prealbumin [TTR] assessment).

4.1.2. Pharmacokinetics and Immunogenicity Data

Ionis is responsible for the management and review of the PK and IM data. This process involves reviewing the patient and visit identifiers with the clinical data collected in the EDC system. The PK and IM data are not stored in the EDC system. Prior to unblinding of the final analysis, any PK concentration and IM datasets provided to Ionis by the bioanalytical labs will be provided without reference to actual patient identifiers to avoid inadvertent or accidental unblinding. The bioanalytical labs may provide Ionis with datasets containing dummy patient identifiers unrelated to the actual identifiers to allow review of the PK and IM data.

An early restricted unblinded population pharmacokinetic access (ERUPA) Team has been appointed for this study. The ERUPA Team is a group of independent Programmers and Scientists who will have early unblinded access to pharmacokinetic data, and certain laboratory and demographic data that is not associated with the efficacy and safety outcomes of the study. The ERUPA Team will use this information to develop a population pharmacokinetic (PopPK) model before database lock. A charter will document this process and list of individuals in the ERUPA team. Robust documentation, IT systems, and where applicable early unblinded team, are required to preserve data integrity.

4.1.3. Echocardiogram Data

The echocardiogram (ECHO) data will be collected, analyzed and stored in a secure database by an independent Contract research organization (CRO) (Cardiac Imaging Core Laboratory [CICL] and Clinical Trials Endpoints Center at the Brigham and Women's Hospital). The sites will upload the ECHO data on a secure web-portal for analysis by CICL. CICL will only inform the site whether the ECHO images are acceptable quality or not. CICL will not provide a formal report, read, or clinical data to the sites. ECHO results data are not stored in the EDC system. Ionis and ICON are responsible for the format of the ECHO electronic data transfers and the transfer schedule. Ionis and ICON are responsible for the data reconciliation of the ECHO results data. This process involves reviewing the patient and visit identifiers in the ECHO results data against the ECHO data collected in the EDC system.

4.1.4. Electrocardiogram Data

All ECG data (machine read) are entered into the Medidata Rave EDC by the sites. The Rave EDC data will be used for ECG summary and analysis.

4.1.5. Adjudicated Data

All deaths and CV clinical events will be adjudicated by an independent Clinical Adjudication Committee (CAC) using the '2017 Cardiovascular and Stroke Endpoint Definitions for Clinical Trials' (Hicks *et al.* 2018). The Duke Clinical Research Institute (DCRI) CAC group is responsible for the conduct of the CAC operations in collaboration with ICON and Ionis pharmaceuticals in a blinded manner. The details of role of the DCRI CAC and adjudication process are provided in the DCRI CAC charter.

4.2. General Presentation Considerations

All tables will present the population frequencies in each treatment group and/or subgroup (where appropriate) and will indicate the number of subjects with non-missing data and the denominators for percentages.

Continuous data will be summarized in terms of the n, mean, standard deviation (SD), median, interquartile range (25th percentile [P25], 75th percentile [P75]), range (minimum, maximum) and number of observations, unless otherwise stated. The minimum and maximum will be reported to the same number of decimal places as the raw data recorded in the database. The mean, median, lower quartile, upper quartile, least squares means and difference in least squares means will be reported to one more decimal place than the raw data recorded in the database. The SD and SE will be reported to one more decimal places than the raw data recorded in the database. In general, the maximum number of decimal places reported shall be four for any summary statistic. All raw values presented in listings will be displayed to the measured precision.

Categorical data will be summarized in terms of the number of subjects providing data at the relevant time point (n), frequency counts and percentages. Any planned collapsing of categories will be detailed in the text and the data displays.

Age will be presented in years. Weight will be presented in kg. Height will be presented in cm. Temperature will be presented in °C. Laboratory values will be summarized using standard units of measure.

Percentages will be presented to one decimal place and not be presented for zero counts. Percentages will be calculated using n as the denominator.

Changes from baseline in categorical data will be summarized using shift tables where appropriate.

P-values greater than or equal to 0.001, in general, will be presented to three decimal places. P-values less than 0.001 will be presented as "<0.001". All statistical tests will be conducted using 2-sided tests with 5% Type I error rates unless otherwise stated.

Confidence intervals (CI) will be presented to one more decimal place than the raw data.

Summaries of plasma drug concentrations and pharmacokinetic parameters will include typical descriptive statistics, such as n, mean, SD, SE, percent Coefficient of Variation (%CV), geometric mean, geometric %CV, median, minimum, and maximum.

All primary and secondary endpoints will be assessed on the Full Analysis Set (FAS). All safety assessments will be performed on the Safety Set (SS). PK endpoints will be assessed in the PK Set as applicable.

Efficacy results will be summarized under the treatment to which patients were randomized. Safety and PK results will be summarized under the treatment which patients actually received. Should there be any cases in which a patient received treatment other than what he/she was randomized, such cases will be discussed in the study report and noted in footnotes where applicable.

All tables and figures containing efficacy endpoints will indicate whether they present raw data or adjusted results from the statistical model, with footnotes indicating the model used and covariates included in the model.

All tables, figures and data listings to be included in the report will be independently checked for consistency, integrity and in accordance with standard Parexel (a CRO that is responsible for pre-programming datasets and TLFs for the Study) procedures.

4.2.1. Baseline

- Baseline for laboratory assessments including pharmacodynamic (PD) and biomarkers will be defined as the average of all non-missing pre-dose assessments.
- Baseline for all other assessments will be defined as the last non-missing value prior to the first dose of the Study Drug.

4.2.2. Definitions and Computational Formulas

- Day 1 will refer to the day of the first dose of study drug.
- BMI will be computed using the formula:

  BMI = (weight in kilograms) / $[\text{height in cm}/100]^2$

- mBMI will be computed from BMI and serum albumin levels by:

  mBMI = BMI * serum albumin (g/L)

4.2.3. Analysis Visit Windows

For the ION-682884-CS2 study, the efficacy and PD data will be assigned to a visit according to the visit windows in the table below. If a patient has discontinued early from the treatment period but attended Early Termination post-treatment follow-up visit to collect assessments, then those assessments collected at Early Termination visit and during post-treatment follow-up period should be mapped to analysis visits as well and included in the analysis and summaries, unless otherwise described. For patients who have multiple visits within a window, the visit nearest the target day will be used unless two visits are equally near, in which case the average value will be used.

**Efficacy/PD measure**	**Nominal Visit (Target Day)**	**Analysis Visit Window (Day)**
6MWT, KCCQ, SF-36, EQ-5D-5L	Week 25 (Day 169)	2-295
	Week 61 (Day 421)	296-547
	Week 97 (Day 673)	548-757
	Week 121 (Day 841)	758-910
	Week 140 (Day 980)	911-1050
	Week 160 (Day 1120)	> 1050
PND, COMPASS-31, LLF, 10-meter walk, Norfolk QOL-DN	Week 37 (Day 253)	2-337
	Week 61 (Day 421)	338-505
	Week 85 (Day 589)	506-715
	Week 121 (Day 841)	716-910
	Week 140 (Day 980)	911-1050
	Week 160 (Day 1120)	> 1050
NYHA class	Week 25 (Day 169)	2-211
	Week 37 (Day 253)	212-295
	Week 49 (Day 337)	296-379
	Week 61 (Day 421)	380-463
	Week 73 (Day 505)	464-547
	Week 85 (Day 589)	548-631
	Week 97 (Day 673)	632-715
	Week 109 (Day 757)	716-799
	Week 121 (Day 841)	800-883
	Week 133 (Day 925)	884-952
	Week 140 (Day 980)	953-1015

Efficacy/PD measure	Nominal Visit (Target Day)	Analysis Visit Window (Day)
	Week 150 (Day 1050)	1016-1085
	Week 160 (Day 1120)	> 1085
PD panel/mBMI	Week 13 (Day 85)	2-127
	Week 25 (Day 169)	128-211
	Week 37 (Day 253)	212-295
	Week 49 (Day 337)	296-379
	Week 61 (Day 421)	380-463
	Week 73 (Day 505)	464-547
	Week 85 (Day 589)	548-631
	Week 97 (Day 673)	632-715
	Week 109 (Day 757)	716-799
	Week 121 (Day 841)	800-883
	Week 133 (Day 925)	884-952
	Week 140 (Day 980)	953-1015
	Week 150 (Day 1050)	1016-1085
	Week 160 (Day 1120)	> 1085
PGIS, PGIC, KPS	Week 61 (Day 421)	2-700
	Week 140 (Day 980)	701-1050
	Week 160 (Day 1120)	> 1050
Biomarkers/NT-proBNP	Week 25 (Day 169)	2-253
	Week 49 (Day 337)	254-421
	Week 73 (Day 505)	422-589
	Week 97 (Day 673)	590-757
	Week 121 (Day 841)	758-910
	Week 140 (Day 980)	911-1050
	Week 160 (Day 1120)	> 1050
ECHO	Week 61 (Day 421)	2-547
	Week 97 (Day 673)	548-826
	Week 140 (Day 980)	> 826

4.3. Software

All report outputs will be produced using SAS® version 9.3 or a later version in a secure and validated environment. SF-36 scoring will be calculated by using Quality Metrics PROCore v2.

4.4. Study Subjects

4.4.1. Disposition of Subjects

The number of patients screened, the number and percentage of patients randomized, the number and percentage of patients dosed, the number and percentage of patients who completed the study, the number and percentage of patients who discontinued from treatment, the number and percentage patients who withdrew from study, including the main reasons for screen failure and reasons for discontinuation/withdrawal, and the number and percentage of patients who enrolled in the OLE study will be summarized by treatment group and overall. These summaries will also be provided for each stratum: NYHA Classification (I and II vs. III), ATTR mutation status (mutated vs. wild-type), current treatment with tafamidis (Yes vs. No), and 6MWT distance (6MWT ≤ 350 meters vs. > 350 meters). The percentage of patients treated will be based on the number of patients randomized, and the percentage of patients who discontinued from treatment and the percentage of patients who withdrew from study will be calculated based on the number of patients treated. The percentage of the reasons for discontinuation from treatment and study will be based on the number of patients who discontinued from treatment and withdrew from study, respectively.

In patients who withdrew from the study or discontinued the study prior to Day 980, vital status at Week 140 (Day 980) will be summarized as Alive, Dead, or Unknown. In patients who withdrew from the study or discontinued the study prior to Day 1120, vital status at Week 160 (Day 1120) will be summarized as Alive, Dead, Unknown, Week 160 not yet reached, or Unknown, Week 160 reached. The participant is to be considered as having reached Week 160 if the participant received first treatment at least 1119 days prior to the report data cutoff date, regardless of whether they had already withdrawn consent or were lost to follow-up at that time.

4.4.2. Protocol Deviations

Protocol deviations will be categorized to major or minor based on study protocol deviation guidance document. Major protocol deviations will be summarized by deviation category and all protocol deviations will be listed. Protocol deviations due to COVID-19 related impact will be summarized as well.

4.5. Analysis Sets

The number of patients in each analysis population will be summarized by treatment group and overall for all randomized patients.

4.5.1. Screened Patients

Screened patients will be defined as those patients who signed an informed consent form.

4.5.2. Randomized Patients

Randomized patients will be defined as those screened patients who received a randomization assignment. Results will be summarized under the treatment to which patients were randomized.

4.5.3. Full Analysis Set

All randomized patients who received at least 1 injection of ION-682884 or placebo and who have at least 1 post-Baseline of primary efficacy assessment or 1 post baseline study visit (i.e., post-Baseline CV clinical event, post-Baseline study visit, vital status).

4.5.4. Safety Set

All patients who are randomized and received at least 1 dose of ION-682884 or placebo.

4.5.5. PK Set

All patients who are randomized, received at least 1 dose of ION-682884, and have at least 1 evaluable PK sample.

4.6. Demographic and Other Baseline Characteristics

Demographic and other baseline characteristics will be descriptively summarized by treatment group for the Randomized Patients, SS, and FAS. Patient's allocation by investigative site and distribution by stratification factor will be tabulated by treatment group and overall for randomized patients.

The following summary and listing will be provided:

Patient demographic characteristics, including age, sex, race, ethnicity, ATTR mutation status, ATTR mutations/variants, duration of disease from ATTR-CM diagnosis (months), duration from onset of ATTR-CM symptoms (months), presence of ATTR-PN (mixed phenotype), current treatment with tafamidis at baseline, and disease family history will be summarized by treatment group and overall. Baseline severity of illness, including the baseline 6MWT, LLF, 10-meter walk, PND score, weight, height, BMI, mBMI, NAC stage, eGFR, and NT-proBNP, will be summarized by treatment group and overall. Measures of quality of life and level of functioning, including the baseline NYHA class, KCCQ overall summary score, PGIS, Norfolk QOL-DN total score, EQ-5D and SF-36 overall score will also be summarized.

NAC stage is defined as:

- I = NT-proBNP < =3000 ng/L and eGFR > =45 mL/min/1.73 m^2
- II = NT-proBNP > 3000 ng/L and eGFR > =45 mL/min/1.73 m^2 OR NT-proBNP < =3000 ng/L and eGFR < 45 mL/min/1.73 m^2
- III = NT-proBNP > 3000 ng/L and eGFR < 45 mL/min/1.73 m^2

The ATTR-CM diagnosis and onset date of ATTR-CM symptoms only collected year and month information in CRF. To facilitate the calculation of duration, the following imputation rules will be applied:

- If year is missing, no imputation will be performed.
- Day will be imputed as 01.

- If month is missing and the recorded year is before the year in informed consent date, the missing month will be imputed to be December; otherwise, it will be imputed as the month from the informed consent date.

A by-patient listing of demographics and baseline characteristics will be presented based on the Randomized Patients. This will include the year of birth, age (in years), sex, race, ethnicity, height (in cm), weight (in kg) and BMI. The body weight will be the measurement obtained at Screening. The baseline characteristics to be listed will include current treatment with Tafamidis, NYHA class, ATTR mutation variants and status, 6MWT (m), NT-proBNP (pmol/L) and PND score.

Medical history will be coded by Medical Dictionary for Regulatory Activities (MedDRA) dictionary Version 25.0 or higher version and will be summarized by system organ class (SOC) and preferred term (PT) by treatment group and overall for the SS. Medical history will also be provided in the data listings.

4.7. Concomitant Medications

Medications will be coded using Anatomical Therapeutic Chemical (ATC) Classification codes by World Health Organization-Drug Dictionary (WHO-DD) version March 2022 or higher version.

Prior and concomitant medications will be summarized by ATC class, Preferred Term by treatment group and overall on SS. Prior and concomitant medications will be defined using the start and stop dates, and ongoing fields recorded in the Case report form (CRF)s relative to the first and last dose dates of randomized investigational product. A prior medication is defined as any medication started prior to the start date of investigational product. A concomitant medication is defined as any medication taken on the same day or after the first dose of Study Drug.

Note that if either of the start dates or stop dates of prior/concomitant medication are missing, the worst, or most conservative, case will be considered when slotting medications (i.e., the medications should slot into all possible phases). If a medication is administered pre-treatment or after first dose of investigation product and no stop date/time is recorded, then usage will be assumed to be ongoing for the remainder of the data collection periods. If a medication is stopped after first dose and no start date/time is recorded it will be assumed that the medication was ongoing from prior to the start of investigational product. If a medication has no start or stop date it will be assumed that the medication was ongoing from prior to the start of investigational product. If a partial date is recorded in the CRF, the following convention will be used to assign the medication:

- If the partial date is a start date, a '01' will be used for the day and 'Jan' will be used for the month
- If the partial date is a stop date, a '28/29/30/31' will be used for the day (dependent on the month and year) and 'Dec' will be used for the month.

The recorded partial date will be displayed in listings.

Tafamidis use prior to Day 1 will be summarized by descriptive summary statistics and categories: >0 - < 6 months vs. ≥ 6 months, and other categories if appropriate. Also, tafamidis use changes during the study will be summarized.

Change of TTR-stabilizer use, including Tafamidis and Acoramidis, after the first dose will be summarized by descriptive summary statistics. For patients who do not use TTR-stabilizer at baseline, number of patients who start using TTR-stabilizer and time since first dose of investigation product to start of TTR-stabilizer will be summarized. For patients using TTR-stabilizer at baseline, number of patients who terminate using TTR-stabilizer will be summarized.

Vitamin A use during the treatment period will be summarized as well.

4.8. Treatment Compliance

Study drug will be administered as subcutaneous (SC) injections. A descriptive summary of treatment compliance variables will be provided:

- Total number of doses received
- Total dose injected (mg)
- Total volume injected (mL)
- Duration of Study Drug exposure (months): Duration will be derived as the difference between the date of the last dose of study drug and the date of the first dose plus one divided by 30.4375.

  If the last dosing date is partially missing, it will be imputed using the most recent prior visit with a complete dosing date, plus the difference in nominal visit weeks × 7 days (corresponding to 28 days between consecutive visits per the 4-week dosing schedule). If the imputed date exceeds the data cutoff date, it will be set to the data cutoff date.
- Overall treatment compliance by groups (%):

  Actual total volume injected (mL) /Expected total volume (mL) to be administered *100. For patients who discontinued treatment, denominator should be based on the total volume expected until discontinuation.
- Treatment compliance by categories (< 80%, 80%- < 100%, 100%, > 100% if available)
- Reason for dose pause/missed dose.

4.9. Efficacy Evaluation

4.9.1. Estimand for Primary and Secondary Outcomes

The primary objective will be assessed through a primary estimand with the following attributes:

- **Treatment:** ION-682884 or placebo on top of SoC.

- **Population:** Patients with transthyretin-mediated amyloid cardiomyopathy as defined by the inclusion and exclusion criteria. Implemented using FAS.
- **Variable:** Composite of recurrent unfavorable events (CV clinical events and CV death) up to Day 980 from the first dose (Week 140).
- **Intercurrent event strategies:**
  - For the intercurrent event of treatment discontinuation, a treatment policy strategy will be used.
  - For the intercurrent event of on-study initiation of TTR stabilizers, a treatment policy strategy will be used.
  - The intercurrent event of Non-CV death will be handled using a hypothetical strategy, by assuming participants cannot die of Non-CV death implemented by censoring at time of death and not imputing data after death.
- **Population-level summary for the variable:** The rate ratio (RR).

The secondary objective to evaluate exercise tolerance will be assessed through a secondary estimand with the following attributes:

- **Treatment:** ION-682884 or placebo on top of SoC.
- **Population:** Patients with transthyretin-mediated amyloid cardiomyopathy as defined by the inclusion and exclusion criteria. Implemented using FAS.
- **Variable:** Change from baseline in the 6MWT distance at Week 140.
- **Intercurrent event strategies:**
  - For the intercurrent event of treatment discontinuation, a treatment policy strategy will be used.
  - For the intercurrent event of on-study initiation of TTR stabilizers, a treatment policy strategy will be used.
  - For the intercurrent event of death, a composite strategy where death is handled as a negative outcome is adopted, see the multiple imputation framework described in Section 4.9.2.2.
  - In addition, missing data caused by intercurrent events are handled in the framework described in Section 4.9.2.2.
- **Population-level summary for the variable:** Difference in means.

The secondary objective to evaluate PRO will be assessed through a secondary estimand with the following attributes:

- **Variable:** Change from baseline in KCCQ overall summary score at Week 140.
- **Treatment, Population, Intercurrent event strategies and Population-level summary** for the variable are same as for exercise tolerance.

The secondary objective to evaluate the occurrence of CV clinical events will be assessed through a secondary estimand with the following attributes:

- **Treatment:** ION-682884 or placebo on top of SoC.
- **Population:** Patients with transthyretin-mediated amyloid cardiomyopathy as defined by the inclusion and exclusion criteria. Implemented using FAS.
- **Variable:** Recurrent unfavourable events (CV clinical events) up to Day 980 from the first dose (Week 140).
- **Intercurrent event strategies:**
  - For the intercurrent event of treatment discontinuation, a treatment policy strategy will be used.
  - For the intercurrent event of on-study initiation of TTR stabilizers, a treatment policy strategy will be used.
  - The intercurrent event of all-cause death will be handled using a hypothetical strategy, by assuming participants cannot die, implemented by censoring at time of death and not imputing data after death.
- **Population-level summary for the variable:** The rate ratio (RR).

The secondary objective to evaluate the occurrence of all-cause death up to Week 160 will be assessed through a secondary estimand with the following attributes:

- **Treatment:** ION-682884 or placebo on top of SoC.
- **Population:** Patients with transthyretin-mediated amyloid cardiomyopathy as defined by the inclusion and exclusion criteria. Implemented using FAS.
- **Variable:** Time to all-cause death up to Day 1120 from first dose (Week 160).
- **Intercurrent event strategies:**
  - For the intercurrent event of treatment discontinuation, a treatment policy strategy will be used.
  - For the intercurrent event of on-study initiation of TTR stabilizers, a treatment policy strategy will be used.
- **Population-level summary for the variable:** The hazard ratio (HR).

The secondary objective to evaluate the occurrence of all-cause death up to Week 140 will be assessed through a secondary estimand with the following attributes:

- **Variable:** Time to all-cause death up to Day 980 from first dose (Week 140).
- **Treatment, Population, Intercurrent event strategies and Population-level summary** for the variable are same as for time to all-cause death up to Day 1120.

The secondary objectives to evaluate the occurrence of composite of CV clinical events and CV death in patients on tafamidis will be assessed through a secondary estimand with the following attributes:

- **Population:** Patients with transthyretin-mediated amyloid cardiomyopathy as defined by the inclusion and exclusion criteria and receiving tafamidis. Implemented using subgroup of patients in FAS with tafamidis at baseline.
- **Treatment, Variable, Intercurrent event strategies and Population-level summary for the variable** are same as primary estimand.

The secondary objectives to evaluate the occurrence of CV death up to Week 160 will be assessed through a secondary estimand with the following attributes:

- **Treatment:** ION-682884 or placebo on top of SoC.
- **Population:** Patients with transthyretin-mediated amyloid cardiomyopathy as defined by the inclusion and exclusion criteria. Implemented using FAS.
- **Variable:** Time to CV death up to Day 1120 from first dose (Week 160).
- **Intercurrent event strategies:**
  - For the intercurrent event of treatment discontinuation, a treatment policy strategy will be used.
  - For the intercurrent event of on-study initiation of TTR stabilizers, a treatment policy strategy will be used.
  - In the analysis of CV death, Non-CV death will be handled using a hypothetical strategy, by assuming participants cannot die, implemented by censoring at time of death and not imputing data after death.
- **Population-level summary for the variable:** The hazard ratio (HR).

The secondary objectives to evaluate the occurrence of CV death up to Week 140 will be assessed through a secondary estimand with the following attributes:

- **Variable:** Time to CV death up to Day 980 from first dose (Week 140).
- **Treatment, Population, Intercurrent event strategies and Population-level summary** for the variable are same as for time to CV death up to Day 1120.

4.9.2. Analysis and Data Conventions

6MWT

The 6MWT is used to assess walking distance in meters (m) at a constant and unhurried pace within 6 minutes. Patients are asked to cover the maximum distance at self-selecting speed. They are instructed that they can slow down and rest if necessary and then start again, if possible. Patients are allowed to use a cane or a walker and/or to perform the test, and the test is performed by trained site staff in accordance with the 6MWT study manual.

KCCQ

The KCCQ is a 23-item self-administered questionnaire developed to independently measure the patient's perception of their health status, which includes heart failure symptoms, impact on physical and social function, and how their heart failure impacts their QoL within a 2-week recall

period. All items are measured on a Likert scale with 5–7 response options. There are five individual subscales, and all, except the Self-efficacy subscale, are aggregated into clinical and overall summary scores. Scores for each subscale are standardized to range from 0 to 100 with higher scores indicating better health status, fewer symptoms, and greater disease-specific Health-related quality of life (HRQoL).

The KCCQ tool quantifies the following 5 distinct domains and 3 summary scores:

- **Symptom Domain (frequency and burden)** measures recent changes in patients' symptoms; their shortness of breath, fatigue or swelling with their frequency 2 weeks ago.
- **Physical Limitation Domain** measures the limitations patients experience, due to their heart failure symptoms, in performing routine activities. Activities are common, gender-neutral, and generalizable across cultures, while also capturing a range of exertional requirements.
- **Self-efficacy Domain** quantifies patients' perceptions of how to prevent heart failure exacerbations and manage complications when they arise. This scale is not included in the summary scores.
- **Quality of Life Domain** is designed to reflect patients' assessment of their quality of life, given the current status of their heart failure.
- **Social Limitation Domain** quantifies the extent to which heart failure symptoms impair patients' ability to interact in a number of gender-neutral social activities.

To facilitate interpretability across several domains, 3 summary scores for the KCCQ were also developed:

- The Total Symptom Score is the mean score of 2 symptom domain scores: Symptom Frequency and Symptom Burden.
- The Clinical Summary Score is the mean of the Total Symptom Score and the Physical Limitation domain score.
- The Overall Summary Score is the mean of 4 scores: the Total Symptom Score, the Physical Limitation domain score, the Social Limitation domain score, and the QoL domain score.

The Overall Summary Score is a comprehensive measure of all of the domains except self-efficacy domain of the KCCQ and will be compared as the secondary endpoint in the primary analysis of KCCQ. The details of KCCQ algorithm is in Appendix 6.1. Only self-administered questionnaire results will be included in the primary analysis of KCCQ. Proxy assessments from caregivers for patients who have been deemed as unreliable self-reporters (e.g., due to cognitive impairment) will not be included in analyses.

NYHA

NYHA classification was first developed in 1928 and has served as a fundamental tool for risk stratification of heart failure and determines clinical trial eligibility and candidacy for drugs and devices. It has been revised from the original. It places patients in 1 of 4 categories based on

degree of limitation during physical activity. The limitations/symptoms regard to normal breathing and varying degrees in shortness of breath and/or anginal pain. NYHA class I represents no symptoms, class II represents symptoms during ordinary activities, class III represents symptoms with minimal exertion, and class IV with symptoms at rest.

COMPASS-31

COMPASS-31 is a 31-item questionnaire that evaluates autonomic function across 6 domains: orthostatic intolerance, vasomotor, secretomotor, gastrointestinal, bladder, and pupillomotor.

Individual questions within domains inquire about symptom frequency (rarely to almost always), symptom severity (mild to severe), and symptom improvement (gotten much worse to completely gone).

Questions include either “yes” or “present” will be scored as 1, “no” or “not present” scored as 0. All questions regarding the frequency of symptoms will be scored as 0 points for “rarely” or “never”, 1 point for “occasionally” or “sometimes”, 2 points for “frequently” or “a lot of the time” and 3 points for “almost always” or “constantly”. All questions regarding the severity of symptoms will be scored as 1 point for “mild”, 2 points for “moderate”, and 3 points for “severe”. When assessing the time course of a symptom, will be scored 0 points for responses of representing improvement such as “gotten/getting somewhat better”, “gotten/getting much better”, “completely gone” and “I have not had any of these symptoms”, 1 point for “stayed about the same”, 2 points for “gotten/getting somewhat worse” and 3 points for “gotten/getting much worse.” The scores for changes in bodily functions depended on the individual question asked. For example, “I get full a lot more quickly now than I used to when eating a meal” will be scored 2 points, “I get full more quickly now than I used to when eating a meal” will be score as 1 point, and “I get full less quickly now than I used to”, “I get full a lot less quickly now than I used to” will be scored 0 points, while the answer “I sweat somewhat more than I used to”, “I haven’t noticed any changes” will be given 0 point, “I sweat much more than I used to” and “I sweat somewhat less than I used to” will be given 1 point and “I sweat much less than I used to” will be scored 2 points. A detailed list of can be found in Appendix 6.2.

Depending on the domain, possible scores in each domain ranged from 6 (vasomotor) to 28 (gastrointestinal). Final score is a sum total of all subscales varying from 0 to 100 with higher scores indicates worsening of autonomic dysfunction.

EQ-5D-5L

EQ-5D-5L is a standardized measure of health status developed by the EuroQoL Group to provide a simple, generic measure of health for clinical and economic appraisal. The EQ-5D-5L consists of the EQ-5D-5L descriptive system and the EQ visual analog scale (VAS).

The EQ-5D-5L descriptive system comprises five dimensions (mobility, self-care, usual activities, pain/discomfort and anxiety/depression), and the response to each dimension is scored as: 1=no problems/pain/anxious, 2=slight problems/pain/anxious, 3=moderate problems/pain/anxious, 4=severe problems/pain/anxious, 5=unable/extreme. Lower score indicates worsening of QoL.

The EQ VAS is used as a quantitative measure of health outcome as judged by the individual respondent. The EQ VAS records a self-rating of health status on a vertical VAS anchored at 100 (best imaginable health state) at the top and 0 (worst imaginable health state) at the bottom.

Additionally, the EQ-5D health state index score reflects how good or bad a health state is according to the preferences of the general population of a country/region will be derived. If any of five dimensions is missing, then the index value will be set as missing. The details of EQ-5D health state index score derivation algorithm is described in Appendix 6.3.

PGI

Patient Global Impression of Severity (PGI-S) is a single-item scale instrument asking patients to rate the severity of their amyloidosis condition on a scale of (1) none, (2) mild, (3) moderate, (4) severe, (5) very severe.

Patient Global Impression of Change (PGI-C) is a single-item scale, which aims to evaluate all aspects of patients' health and determine if there has been an improvement or not in the patients' overall change in amyloidosis condition. Patients rate their change as (1) much better, (2) a little better, (3) no change, (4) a little worse, (5) much worse.

SF-36

The SF-36 comprising 36 items that yield an 8-subscale profile of functional health and well-being, as well as physical and mental health summary measures.

From 35 of the 36 items, 8 subscales and 2 summary measures are constructed. The 8 subscales are:

- Physical functioning (PF)
- Role-Physical (RP)
- Bodily pain (BP)
- General health (GH)
- Vitality (VT)
- Social Functioning (SF)
- Role-Emotional (RE)
- Mental Health (MH)

The 2 summary measures are:

- Physical component summary (PCS)
- Mental component summary (MCS)

Scoring of the PCS and MCS involves three steps: First, 8 subscales (raw scores) are standardized using the means and standard deviations. Second, they are aggregated using weights (factor scores coefficients). Finally, all 8 subscales and aggregate PCS and MCS scores are standardized using a linear T-transformation to have a mean of 50 and a standard deviation of 10. All norms and scoring algorithms used will be based on the US population norms.

SF-36 scores will be computed by using Quality Metrics PROCore v2 using missing score estimation option.

KPS

The KPS ranking describes patient's health status as a comprehensive 11-point scale correlating to percentage values ranging from 0 to 100, with 0 being patient death to 100 being patient in perfect health condition.

LLF

LLF evaluate three domains in both right and left side: walking on toes, walking on heels, and arising from a kneeled position. Result "Normal" will be scored as 0, "Abnormal" will be scored as 1. Total LLF score will be sum of 6 items.

Norfolk QOL-DN

The Norfolk QOL-DN is a self-administered questionnaire, designed to capture and quantify the impact of diabetic neuropathy on the quality of life of individual patients with diabetic neuropathy. It contains 35 scored questions related to five domains as below.

Norfolk QOL-DN Domain	Question Number
physical functioning/large fiber neuropathy	8, 11, 13-15, 24, 27-35
activities of daily living	12, 22, 23, 25, 26
Symptoms	1-7, 9
small fiber neuropathy	10, 16, 17, 18
autonomic neuropathy	19, 20, 21

In general, items 1–7 (Part I) are a simple inventory of symptoms of neuropathy. Each of these questions are assessed for the feet, legs, hands and arms, with presence of a symptom scored as a 1 and absence as a 0. The question score is calculated by summing the scores for the individual sites for the question. Items 8-35 (Part II) pertain to Activities of Daily Life, and most of these are scaled on a 5-point Likert scale ranging from 0 ("Not a problem") to 4 ("Severe problem"). However, Questions 31 and 32 are scored differently. In Question 31, "Good", the middle item, is scored as 0. "Very Good" is scored as -1, Excellent" is scored as -2. "Fair is scored as 1, and "Poor" is scored as 2. In Question 32, "About the Same", the middle item, is scored as 0. "Somewhat better" is scored as -1, "Much better" is scored as -2. "Somewhat worse" is scored as 1, and "Much worse" is scored as 2.

The Total QOL and five domains should be summed as follows:

- Total QOL Σ(1-7, 8-35)
- Physical Functioning/Large Fiber Σ(8, 11, 13-15, 24, 27-35)
- Activities of Daily Living (ADLs) Σ(12, 22, 23, 25, 26)
- Symptoms Σ(1-7, 9)

- Small Fiber Σ(10, 16, 17, 18)
- Autonomic Σ(19, 20, 21)

For each patient at a specific visit (defined by the analysis visit window), if at least 50% of the questions for a domain (physical functioning/large fiber neuropathy, activities of daily living, symptoms, small fiber neuropathy) are not missing or if at least one question is not missing for autonomic domain, the missing questions are imputed as follows: If any question is missing at baseline, the mean value for this question at baseline from the study population (across all treatment groups) will be used to impute the missing baseline question value. For post-baseline visits during the treatment period, any missing question values will be imputed using the last observed or imputed question value (including baseline value). For the symptom domain, in the case that a patient responded on a particular question (Questions 1-7) as not having the symptom but also marked presence of the symptom in their feet, legs, hands, or arm, the question will be set to missing and the imputation rules will be followed. Otherwise, the total for that domain will be set to missing

- The Norfolk QOL-DN total score will be calculated by summing the imputed domain scores. If any domain score after imputation is still missing, then the Norfolk QOL-DN total score will be set to missing.
- The Norfolk QOL-DN individual domain and total scores will only be calculated for visits where the patient had a Norfolk QOL-DN assessment. The individual domain and total scores are set to be missing if a patient misses the visit or does not have a Norfolk QOL-DN assessment at that visit.

10MWT

The 10MWT is used to assess walking speed in meters per second (meter/second [m/s]) over a short distance. It can be employed to determine functional mobility, gait, and vestibular function. The 10MWT can be done in 2 different ways: comfortable gait speed or fast walking speed. Lower score indicates worsening. The time is measured for the middle 6 m to allow for patient acceleration and deceleration: the time is started when any part of the leading foot crosses the plane of the 2-meter mark, and the time is stopped when any part of the leading foot crosses the plane of the 8 meter mark.

The total time taken to ambulate 6 meters (m) is recorded to the nearest hundredth of a second. 6 m is then divided by the total time (in seconds) taken to ambulate. If a patient requires total assistance or is unable to ambulate at all, a score of 0 m/s should be documented.

PND Score

The PND is a 6-stage scoring system: Stage 0: no impairment; Stage 1: sensory disturbances in limbs without motor impairment; Stage 2: difficulty walking without the need of a walking aid; Stage 3A: 1 stick or 1 crutch required for walking; Stage 3B: 2 sticks or 2 crutches needed; Stage 4: wheelchair required or patient confined to bed.

4.9.2.1. Adjustments for Covariates

Generally, the efficacy analysis will be adjusted for the following baseline covariates based on eCRF unless otherwise specified (If a covariate is missing from the eCRF, the corresponding stratum factor at randomization will be used instead, where applicable):

- NYHA Classification (I and II vs. III)
- ATTR mutation status (mutated vs. wild-type)
- Current treatment with tafamidis (Yes vs. No)
- Baseline 6MWT distance (6MWT ≤ 350 meters vs. > 350 meters)
- Baseline value of corresponding efficacy endpoint (if applicable)
- Baseline log-transformed NT-proBNP

For analyses of change from baseline in 6MWT, the covariates will include ATTR mutation status (mutated vs. wild-type), Current treatment with tafamidis (Yes vs. No), NYHA Classification (I and II vs. III), and baseline 6MWT as a continuous covariate in the ANCOVA model. For analysis for change from baseline in 6MWT and KCCQ, baseline log-transformed NT-proBNP will not be included as a covariate.

4.9.2.2. Handling of Dropouts or Missing Data

No imputation will be done for missing cases for the primary analysis of the primary endpoint.

Missing data imputation strategies for missing visit level data for 6MWT

If a patient misses a visit (or the visit is performed outside the analysis visit window), or the 6MWT assessment is not conducted at the scheduled visit, then the 6MWT will be considered as missing at that visit and will be imputed using the multiple imputation method, and the endpoint treatment differences (which are adjusted to take account of missing data) are derived. The multiple imputation method is detailed in Section 4.9.2.3.

Missing data imputation strategies for KCCQ at domain level score and overall level score

If a patient has completed at least part of the KCCQ at a visit, then each domain score of KCCQ will be scored based on the algorithm in Appendix 6.1. If at least 2 domains with missing in the overall score (Physical Limitation, Total Symptom Score, Quality of Life, Social Limitation), then KCCQ overall score will be set to missing, and the missing overall score will be imputed using the same method specified in Section 4.9.2.3.

Imputation of Missing/Partial Dates

The imputation of partial or missing dates for adverse events (AE) and partial death date, and prior/concomitant medications are detailed in Section 4.10.1 and Section 4.7.

4.9.2.3. Multiple Imputation Methodology for 6WMT or KCCQ

Patients with missing 6WMT or KCCQ overall score will be imputed using the multiple imputation method in the secondary analyses, unless otherwise stated.

In the primary analysis, the missing data will be modelled using the mixture pattern as below.

- Pattern 1: Missing data after death and missing after heart transplantation and combined heart and liver transplantation, intermediate or long-term cardiac mechanical circulatory support (i.e., ventricular assist device, total artificial heart) will be multiply imputed based on the bottom 20% of observed values at the same visit in the same treatment group, which are considered worst scores.
- Pattern 2: For patients treated with ION-682884 who discontinue the treatment due to AE or disease progression, their missing 6MWT or KCCQ data after treatment discontinuation (last dose date plus 52 days) will be multiply imputed using the copy reference (CR) approach based on the estimates of the placebo patients only. The assumption is that after treatment discontinuation, missing post-baseline outcomes are assumed to follow the same distribution and trajectory as the patients in the placebo arm.
- Pattern 3: Missing data prior to treatment discontinuation will be multiply imputed based on the distribution of their own arm assuming missing at random. All other missing data and COVID-19 related missing will be multiply imputed in the same way as for missing data prior to treatment discontinuation.

The multiple imputation will be conducted using the Fully Conditional Specification with Regression Predictive Mean Matching (FCS REGPMM) method, implemented through the SAS procedure `PROC MI` with the `FCS REGPMM` statement. The imputation model contains following variables: baseline, post-baseline results, and the 3 randomization stratification factors: NYHA Classification, ATTR mutation status, Current treatment with tafamidis at baseline for 6WMT. For KCCQ, it also will include the stratification factor for 6MWT distance (6MWT ≤ 350 meters vs. > 350 meters).

A total of 100 imputed datasets will be generated. Each of 100 imputed data set will then be analyzed using ANCOVA model at Week 140 and the resulting treatment differences and their standard errors will be combined using Rubin's rules. The ANCOVA model will include main effects for treatment (two levels), and the randomization stratification factors (NYHA Classification, ATTR mutation status, Current treatment with tafamidis at baseline, Baseline 6MWT groups for KCCQ additionally) (each with two levels), the interaction term of treatment group times Current treatment with tafamidis at baseline, and the baseline value as the covariate.

A random seed to be used by a random number generator with value of 259804 will be used to initiate data imputation.

4.9.2.4. Data Summary Plan

The Primary Analysis will be performed after the last patient has completed the treatment period or otherwise discontinued (End of Treatment Lock). The Final Analysis will be conducted after the study is completed, i.e., all patients have either discontinued or completed the study (Final Lock). The table below details what data will be included in the summary and analysis outputs by endpoint type, what outputs are planned for end of treatment lock, and what outputs will be updated for the final database lock. Data summarized/analyzed at end of treatment and final lock will be based on data collected up to the date of the data cut. In select instances, the data for a type of endpoint that is summarized or analyzed may not be consistent with the rules in the

Table; such exceptions will be noted in this document. Analyses performed at the End of Treatment Lock will be reported in the CSR and analyses performed at the Final Lock will be reported in a later addendum to the CSR.

**Endpoint Type**	**End of Treatment Lock**	**Final Lock**	**Comment/Definition**
Efficacy	All efficacy endpoints:<br>-Summary/analysis tables (Based on 6MWT, KCCQ imputed data, otherwise, specify)<br>Listings for primary and secondary endpoints		-Visit defined by analysis visit window<br>-The primary/secondary efficacy inference will be made at end of treatment lock
PD	-Listings<br>-Summary/analysis tables		-Visit defined by analysis visit window
ECG, vital signs (including body weight)	-Listings<br>-Summary tables	-Listings<br>-Summary tables	
Lab	-Summary tables	-Summary tables	
AE	-Listings<br>-Summary tables	-Listings<br>-Summary tables	
Concomitant/ Prior medications	-Listings<br>-Summary tables	-Listings<br>-Summary tables	
IM	-Listings<br>-Summary/analysis tables	-Listings<br>-Summary/analysis tables	
PK	-Summary/analysis tables	-Summary/analysis tables	

4.9.2.5. Interim Analysis

An Interim Analysis is not planned for this study.

4.9.2.6. Multiple Comparisons/Multiplicity

To control the Type I error rate at 0.05 for the testing of primary and secondary efficacy endpoints, a hierarchical ranking strategy will be applied, and the testing sequence is defined as below.

Hierarchical Testing Order for Primary and Secondary Endpoints

- Primary Endpoint: Comparison of the composite of CV-death and recurrent CV clinical events up to Week 140 in the Full Analysis Set
- Secondary Endpoint 1: Comparison of change from baseline to Week 140 in the 6MWT distance between the ION-682884 and placebo treatment groups in the Full Analysis Set

- Secondary Endpoint 2: Comparison of change from baseline to Week 140 in the KCCQ overall score between the ION-682884 and placebo treatment groups in the Full Analysis Set
- Secondary Endpoint 3: Comparison of cardiovascular-related clinical events up to Week 140 between the ION-682884 and placebo treatment groups in the Full Analysis Set
- Secondary Endpoint 4: Comparison of all-cause death at Week 160 between the ION682884 and placebo treatment groups in the Full Analysis Set
- Secondary Endpoint 5: Comparison of all-cause death at Week 140 between the ION682884 and placebo treatment groups in the Full Analysis Set
- Secondary Endpoint 6: Comparison of primary endpoint (the composite of CV-death and recurrent CV clinical events) up to Week 140 in subgroup patients with tafamidis use at baseline
- Secondary Endpoint 7: Comparison of cardiovascular death at Week 160 between the ION682884 and placebo treatment groups in the Full Analysis Set
- Secondary Endpoint 8: Comparison of cardiovascular death at Week 140 between the ION682884 and placebo treatment groups in the Full Analysis Set

4.9.2.7. Examination of Subgroups

Exploratory subgroup analyses will be performed to evaluate the consistency of the primary analysis results across subgroups defined by demographic and baseline characteristics. Analyses will be performed on the primary and secondary endpoints for each subgroup. Subgroups based on eCRF (as appropriate per actual subgroup sample size) include but are not limited to:

- NYHA Classification
- ATTR mutation status
- Current treatment with tafamidis at baseline (Yes vs. No)
- Time on tafamidis treatment prior to Day 1 (Categories based tafamidis use prior to Day 1: < 6 months vs. ≥ 6 months, < median vs. ≥ median time, and other categories if appropriate)
- Region (North America, Europe, and Rest of world)
- Gender
- Age (< 65, 65 - 74, ≥ 75 years)

4.9.3. Primary Efficacy Variable

The primary analysis is to compare the composite of CV-death and recurrent CV clinical events up to Week 140 using the Andersen-Gill method (Andersen & Gill 1982), and the treatment effect will be estimated with a robust variance estimator using Lin, Wei, Yang and Ying (LWYY) method (Lin *et al.* 2000) based on FAS.

Let RR to be the rate ratio between the ION-682884 and placebo in the primary endpoint, the following hypothesis test will be conducted:

$$H_0: RR = 1$$

$$H_1: RR \neq 1$$

The death and CV clinical events in all analyses, unless otherwise specified, will be adjudicated by an independent CAC using the ‘2017 Cardiovascular and Stroke Endpoint Definitions for Clinical Trials’ (Hicks *et al.* 2018). Only adjudicated events that occurred prior to or at Week 140 (Day 980) will be utilized, i.e., for patients who have completed the Week 140 follow up in final analysis, the first dosing date plus 979 days will be used, and for patients who do not complete Week 140 follow up, the last clinical assessment date will be used. For hospitalization for arrhythmia events, the independent CAC will adjudicate hospitalizations for arrhythmia and/or arrhythmia events requiring medical treatment in outpatient facility or ER, and only unscheduled hospitalization or ED > 24 hours for arrhythmia adjudicated events will be counted as CV clinical events in the primary endpoint.

Under the semi-parametric proportional rates model (LWYY model), the individual rate function for the composite endpoint of CV death and total CV events is assumed to be,

$$\lambda_i(t) = Y_i(t)\lambda_0(t)\exp\left(\beta_0 x_i + \beta_1 z_{1i} + \beta_2 z_{2i} + \beta_3 z_{3i} + \beta_4 z_{4i} + \beta_5 z_{5i}\right)$$

where $\lambda_i(t, x_i)$ is the individual rate of primary composite events for subject i, given that the patient has not died due to CV reason at time t, and it is dependent on the time from first dose t and treatment group x_i and covariate $z_{1i} - z_{5i}$. Let $x_i = 1$ if the subject is in the ION-682884 group and $x_i = 0$ if the subject is in the placebo group. $z_{1i} - z_{5i}$ are the covariates for the 4 randomization stratification factors (NYHA Classification (I and II vs. III), ATTR mutation status (mutated vs. wild-type), current treatment with tafamidis (Yes vs. No), and 6MWT distance (6MWT ≤ 350 meters vs. > 350 meters), and baseline log-transformed NT-proBNP. $Y_i(t)$ is the predictable process taking value of 0 or 1, indicating (by the value 1) when the subject i is under observation; $\lambda_0(t)$ is the underlying baseline rate function.

exp (β_0) is the rate ratio of total CV events and CV death in the ION-682884 group relative to the placebo group given the patient has not died due to CV at time t, which is assumed to be constant over time. The model estimate exp (β_0) will be computed by maximization of a partial likelihood score function and a robust variance estimator (sandwich estimator) will be used to account for the dependency of within subject events.

The LWYY model can be carried out using the following sample SAS code:

```
proc phreg data=adttee covs(aggregate);
 model (Tstart, Tstop)*cnsr(0)=trt strat1 strat2 strat3 strat4 logbnp /ties=efron rl;
 id subjid;
run;
```

where adttee is the input dataset including primary composite endpoint, Tstart is the previous event start time and Tstop is the current event start time, cnsr is the censoring variable (0 for

censored and 1 for event), trt is the treatment group, strat1-strat4 are the stratification factors, logbnp is the baseline log-transformed NT-proBNP, and subjid is the subject ID.

Patients who complete the treatment and complete Week 140 follow up and have no adjudicated events defined in the primary endpoint prior to or at Week 140 (i.e., the first dosing date plus 979 days) will be censored at Week 140. Patients who died for unknown reasons will be treated as CV death, and patients who died for reasons other than cardiovascular prior to or at Week 140 will be designated as censored at date of death. Patients who discontinue treatment should be encouraged to remain on-study and complete their follow up based protocol scheduled visits. If patients discontinue treatment and remain on the study for alternate follow up collecting at least AE, concomitant medications and vital status, their follow up data will be used in the primary analysis. If patients discontinue treatment and do not agree with alternate follow up but agree with survival follow up at Week 140 only, or patients withdraw consent but have vital status follow-up if permitted by local laws of jurisdiction, then the follow up vital status data will not be used in the primary analysis and the last assessment date for primary analysis will be the treatment termination date, but their follow up vital status data will be used in the secondary endpoints including time to all cause death and time to CV death. For patients who were lost to follow up prior to Week 140, the last assessment date will be defined as last date of assessment for safety or efficacy. For patients who are ongoing but have not reached Week 140 at the time of the data cut off, the last assessment date for primary endpoint will be the data cutoff date. Patients who have transplantation (i.e., heart transplantation and combined heart and liver transplantation) or intermediate or long term cardiac mechanical circulatory support (i.e., ventricular assist device, total artificial heart) prior to or at Week 140 will be treated as having experienced CV death and the date of transplantation (i.e., heart transplantation and combined heart and liver transplantation) or intermediate or long term cardiac mechanical circulatory support (i.e., ventricular assist device, total artificial heart) will be considered as CV death date in the primary efficacy analysis.

The mean cumulative event rate (i.e., mean cumulative function using Nelson-Aalen method) for the composite of CV-death and recurrent CV clinical events will be plotted over time up to Week 140.

4.9.4. Sensitivity, Supplementary, and Exploratory Analyses of Primary Endpoint

Each component of the primary endpoint is defined as additional secondary endpoint and will be analyzed to support the interpretation of primary analysis result. Details are provided in Section 4.9.5.

In addition to the primary efficacy analysis, the following sensitivity analyses for primary endpoint will be conducted:

- **Sensitivity Analysis 1 (FAS)** – To examine the potential effect of including heart transplantation or intermediate or long term cardiac mechanical circulatory support (i.e., ventricular assist device, total artificial heart) as “CV-deaths” in the primary analysis, a sensitivity analysis will be performed treating all transplantation or intermediate or long term cardiac mechanical circulatory support in the same manner as any other censored observation and otherwise using the same model as the primary analysis.

- **Supplementary Analysis 1 (Time to First Event Analysis) (FAS)** – Time to first adjudicated event defined in the primary endpoint will be compared using log-rank test stratified by randomization stratification factors and NT-proBNP group (≤ 3000; > 3000 ng/L). A weighted log-rank test Fleming-Harrington (FH) (0, 1) by giving more weight to events occurring at later time points will also be performed. Similarly to the primary analysis, the first adjudicated events occurred prior to or at Week 140 will be utilized. The data will also be analyzed using Cox proportional hazards model with treatment and stratification factors and baseline log-transformed NT-proBNP as covariates.

- **Supplementary Analysis 2 (Time to First Event Analysis -Subset of patients not on tafamidis at baseline)** – Time to first adjudicated event defined in the primary endpoint will be repeated in the subset of patients who are not on tafamidis at baseline using log-rank test stratified by NYHA Classification (I and II vs. III), ATTR mutation status (mutated vs. wild-type), 6MWT distance (6MWT ≤ 350 meters vs. > 350 meters) and NT-proBNP group (≤ 3000; > 3000 ng/L). A weighted log-rank test Fleming-Harrington (FH) (0, 1) by giving more weight to events occurring at later time points will also be performed. Similarly to the primary analysis, the first adjudicated events occurred prior to at Week 140 will be utilized. Patients who initiate use of a TTR-stabilizer (tafamidis or acoramidis) will be censored on the start date of use, unless they have an earlier event or are censored earlier per censoring rules in Section 4.9.3. The data will also be analyzed using Cox proportional hazards model with treatment and 3 stratification factors including NYHA Classification (I and II vs. III), ATTR mutation status (mutated vs. wild-type), and 6MWT distance (6MWT ≤ 350 meters vs. > 350 meters) and baseline log-transformed NT-proBNP as covariates.

- **Supplementary Analysis 3 (LWYY Analysis Including All-Cause Death in Composite Endpoint – FAS) –** The composite endpoint will include all-cause death and recurrent CV clinical events using the same analysis method as for the primary analysis.

- **Supplementary Analysis 4 (Negative Binomial Regression Analysis - FAS) –** Frequency of recurrent CV clinical events and CV death included in the primary endpoint at the end of study will be analyzed using a Negative Binomial regression analysis with treatment and 4 stratification factors including NYHA Classification (I and II vs. III), ATTR mutation status (mutated vs. wild-type), current treatment with tafamidis (Yes vs. No), and 6MWT distance (6MWT ≤ 350 meters vs. > 350 meters) and baseline log-transformed NT-proBNP as covariates with an offset of logarithm of time from randomization to censoring or death.

- **Supplementary Analysis 5 Joint Frailty Model (FAS)** – The joint frailty model (Cowling *et al.* 2006) with treatment as fixed-effect factor will be used as a sensitivity analysis to address competing risk of all-cause death on CV recurrent events of the primary endpoint and account for correlation between the two components.

  The joint frailty model is defined as follows.

$$\lambda_{i1}(t) = Y_i(t)\lambda_{10}(t)\exp(\beta_{10}x_i + \tilde{Z}_i)$$

$$\lambda_{i2}(t) = Y_i(t)\lambda_{20}(t)\exp(\beta_{20}x_i + \gamma\tilde{Z}_i)$$

Where $\lambda_{i1}(t)$ is the rate function of the recurrent CV clinical events included in the primary endpoint for subject i, and $\lambda_{i2}(t)$ is the rate function of all-cause death for subject i. λ_{10} and λ_{20} denote baseline hazards. Z_i defined as the random variable "frailty" for subject i, and $\tilde{Z} = \log(Z)$. For frailty Z_i, a gamma distribution with expected mean of 1 and variance of θ will be assumed. γ is the association parameter between $\lambda_{i1}(t)$ and $\lambda_{i2}(t)$. The parameters will be estimated through maximization of likelihood.

The baseline hazard functions for both recurrent CV events and death are approximated by piecewise constant functions. If there is convergency issue, the constant bassline hazard functions will be assumed. If there is convergency issue further, frailty with log-normal distribution can be used.

These analyses can be implemented using SAS NLMIXED procedure.

- **Exploratory analysis for subgroups** will be performed in each subset of the interest using the same analysis method as for the primary analysis, and the corresponding covariate will be removed from the model as appropriate.

4.9.5. Secondary Efficacy Variables

The secondary efficacy variables are defined as in the hierarchical order in Section 4.9.2.6, and primary analyses for secondary endpoints are defined as below.

Continuous variables including change from baseline to Week 140 in 6MWT distance and KCCQ overall summary score will be compared between ION-682884 and placebo treatment groups in the FAS using an ANCOVA model respectively. The ANCOVA model will include stratification factors, baseline value, and the interaction term of current treatment with tafamidis (Yes vs. No) at baseline times treatment group as covariates. Patients with missing endpoint will be imputed using a pattern mixture model. More details for missing data imputation and analysis method are described in Section 4.9.2.3. The least-square mean of change from baseline in 6MWT distance and KCCQ will be plotted.

Recurrent CV clinical events up to Week 140 will be analyzed using the Andersen-Gill method with a robust variance estimator (LWYY) similarly to the primary analysis. For hospitalization for arrhythmia events, only unscheduled hospitalization or ED > 24 hours adjudicated events will be counted as CV clinical events, using the same definition as for the primary endpoint. The cumulative mean event rate for CV clinical events, as well as for each component, will be plotted over time up to Week 140.

Time to all-cause death up to Week 160, time to CV death up to Week 160, time to all-cause death up to Week 140, and time to CV death up to Week 140 will be analyzed using a Cox proportional hazards model with treatment and 4 stratification factors including NYHA Classification (I and II vs. III), ATTR mutation status (mutated vs. wild-type), current treatment with tafamidis (Yes vs. No), 6MWT distance (6MWT ≤ 350 meters vs. > 350 meters), and baseline log-transformed NT-proBNP as covariates. If patients discontinue treatment and agree

alternate follow up or agree survival follow up only, or patients withdraw consent but may have vital status follow-up if permitted by local laws of jurisdiction, their vital status obtained during follow up will be used in analyses. For Week 140 analyses, the last survival follow up date (censoring date or event date) used for analysis will be defined as the earlier of the last known alive/death date or the first dosing date of CS2 study plus 979 days. For Week 160 analyses, the last survival follow up date (censoring date or event date) used for analysis will be defined as the earlier of the last known alive/death date or the first dosing date of CS2 study plus 1119 days. More specifically, for patients who do not enter OLE study, the post treatment follow up data up to Week 160 in CS2 will be included; and for patients who enter OLE study (ION682884-CS12), the follow up data of CS12 will be included if it is within defined last survival follow up date for analysis.

Comparison of primary endpoint (the composite of CV-death and recurrent CV clinical events) up to Week 140 in subgroup patients with tafamidis use at baseline will be conducted using the Andersen-Gill method with a robust variance estimator (LWYY) similarly to the primary analysis.

4.9.6. Sensitivity and Exploratory Analyses of Secondary Endpoints

4.9.6.1. Change from Baseline to Week 140 in 6MWT and KCCQ

- **Sensitivity Analysis 1** (Observed Data) – The primary efficacy analysis will be repeated using observed data without imputation.
- **Sensitivity Analysis 2 (Tipping Point Analysis)**

  An additional sensitivity analysis using the 2-dimensional tipping-point approach will be conducted in the FAS to assess the impact of missing data on the result of the primary analysis. In the tipping point analysis, the data will be imputed as follows, based on the multiply imputed data for the primary analysis:

  For the placebo group, a positive constant shift (δc) will be added to or subtracted from imputed results. The shift will be gradually increased.

  For each value of the placebo shift parameter, a positive constant shift (δt) will be added to or subtracted from imputed values in the active treatment groups. The shift will be increased in a repeated process until the treatment effect estimated from the same ANCOVA model used for the primary analysis is no longer significant at 0.05 level.
- **Supplementary Analysis 1** (Non-Parametric Analysis) – The non-parametric Van-Elteren test by stratification factors (specified in Section 4.9.2.3) will also be performed for 6MWT/KCCQ on multiple imputation data as a sensitivity analysis. Hodges-Lehmann estimates and 95% CIs of the differences between ION-682884 group and placebo group will also be provided. The Van-Elteren test results were combined using Rubin's rules.
- **Supplementary Analysis 2** (Responder Analysis) – A responder analysis based on the change in 6MWT/KCCQ will be conducted to examine whether improvement in response is consistent over a range of response thresholds. A responder is defined as a

patient whose 6MWT/KCCQ change from baseline to Week 140 is greater than or equal to the threshold value. The relationship between response rate and thresholds will be summarized using a cumulative distribution plot. In addition, comparison of response rates for specific threshold values will be done. For 6MWT, thresholds that will be tested will include but not limit to 0, -10, -20, -30, -35, -40, -50, -60, -70, and -80 meters change from the baseline value. For KCCQ, thresholds that will be tested will include but not limit to 10, 5, 0, -5, -10, -15, -20 score change from the baseline value. For each of these specific response thresholds, the response rates at Week 140 for both the treated group and the placebo group will be calculated and plotted against the response threshold. Patients who had missing Week 140 data irrespective of the reason will be considered a non-responder in the analysis. The difference in the estimated response rate and associated 95% CI based on the Mantel-Haenszel approach by stratification factors (specified in Section 4.9.2.3) will be presented for each threshold.

- **Exploratory analysis for subgroups** will be performed in each subset of the interest using the same ANCOVA model and multiple imputation methods detailed in Section 4.9.2.3 for primary analysis, and corresponding covariate will be removed from the model as appropriate.

  Exploratory analysis at other post-baseline timepoints will be performed using the same ANCOVA model and multiple imputation methods detailed in Section 4.9.2.3 for primary analysis.

4.9.6.2. CV Clinical Events

- **Sensitivity Analysis (Competing risk) –** To assess the competing risk of all-cause death events on CV clinical events, a joint frailty model will be performed as specified in Section 4.9.4
- **Supplementary Analysis (Negative Binomial)** – Frequency of CV clinical events at Week 140 will be analyzed using a Negative Binomial regression analysis with treatment and 4 stratification factors including NYHA Classification (I and II vs. III), ATTR mutation status (mutated vs. wild-type), current treatment with tafamidis (Yes vs. No) at baseline, 6MWT distance (6MWT ≤ 350 meters vs. > 350 meters), and baseline log-transformed NT-proBNP as covariates with an offset of logarithm of time from first dose to censoring or death.
- **Supplementary Analysis for Components of CV Clinical Events**

  Each component of the primary endpoint including MI, HF, arrhythmia, stroke/TIA, and HF urgent visits will be analyzed using the same analysis method as for the primary analysis of the primary endpoint. In addition, composite of HF and HF urgent visits will be analyzed similarly.
- **Exploratory analysis for subgroups** will be performed in each subset of the interest using the same method as for the primary analysis, and the corresponding covariate will be removed from the model as appropriate.

4.9.6.3. Time to Event Endpoints (All-cause Death and CV Death)

- **Sensitivity Analysis (Log-rank Test and Weighted Log-rank Test):** Time to event endpoints will be analyzed using log-rank test stratified by stratified by randomization stratification factors and baseline NT-proBNP group (≤ 3000; > 3000 ng/L). P-value, KM % and their 95% CI for each treatment group will be estimated at Week 140 or Week 160 respectively. Kaplan Meier survival curves for each treatment group will be presented. With an anticipated delayed treatment effect based on historical data in ATTR-CM trials, a weighted log-rank test Fleming-Harrington (FH) (0, 1) by giving more weight to events occurring at later time points, will be performed as a sensitivity analysis.
- **Supplementary Analysis (Competing risk) –** To assess the competing risk of non CV death on time to CV death, Fine and Gray's sub-distribution hazard model (Fine & Gray 1999) will be used.
- **Exploratory analysis for subgroups** will be performed in each subset of the interest using the same analysis method as for primary analysis, and the corresponding covariate will be removed from the model as appropriate. Cox proportional hazards model will be performed to evaluate subgroup effect on these endpoints.

4.10. Safety Evaluation

All safety summaries and analyses will be based upon the Safety Set. For AE and lab abnormalities, in addition to summary using frequency and percentages, time to first event will be summarized by Kaplan-Meier (KM) method, Week 140 (i.e., Day 980 from first dose) KM% will be presented. The CI for the difference in KM% between treatment arms will be obtained through application of the normal approximation to the distribution of the difference in KM% estimates.

4.10.1. Adverse Events

AE will be coded using the MedDRA coding system version. The final version used will be designated in the clinical study report.

The following imputation rules will be applied to impute AE start dates under conservative principles. If the month, year, and day are missing, the adverse event start date will be imputed as the treatment start date. If month and day are missing and year is available and is the same year as in treatment start date, the month and day from the treatment start date will be used to impute the missing month and day for the adverse event start date. Otherwise, missing month and day will be imputed as January 01. If day is missing and month and year is available and the month and year are the same month and year in treatment start date, the day from the treatment start date will be used to impute missing day for the adverse event start date. Otherwise, missing day will be imputed as 01.

If fatal AE stop date is a partial date, '28/29/30/31' will be used for the day (dependent on the month and year) and 'Dec' will be used for the month if month is missing. If year is missing, fatal AE end date will not be imputed. If the imputed fatal AE end date is after data cutoff date, then it will be set to the data cutoff date.

If death date is a partial date, it will be imputed using the same rule as fatal AE stop date. The imputation rule will be applied to both safety and efficacy analyses.

Treatment-emergent adverse events (TEAEs) are defined as adverse events that occurred on or after the first administration of Study Drug. Note that imputed dates will be used for defining TEAEs in case of missing or incomplete dates.

In addition, if severity of an AE changes during the study, a separate AE will be recorded for each severity on the AE CRF. "first" and "second" AE will be identify based on AE start date. AE start date of the second record is AE stop date of first record. These linked events should be compared pairwise, and consider two cases, where the AE severity (mild/moderate/severe) and seriousness (Yes/No) are compared between the two records in the pair.

- Case 1: The first AE record in the linked group occurs before first dosing, and the second AE record occurs after first dosing.
  - If the AE severity or seriousness of the second record is worse than that of the first record, then only the second AE is deemed as a TEAE. Otherwise, neither record is considered as TEAE.
- Case 2: Both AE records in the pair occur after first dosing.
  - The worst AE will be deemed as a TEAE.

AE occurred on or after the first administration of Study Drug and leading to study drug discontinuation will be deemed as TEAE.

Drug-Related AEs include AEs with relationship of "related", "possible", and missing relationship.

The following summaries and listings will be provided for TEAEs by treatment group:

- An Overall AE summary table including TEAEs and SAEs.
- A summary table of TEAEs by SOC and PT.
- A summary table of TEAEs by SOC, PT, and maximum severity.
- A summary table of TEAEs by PT.
- A summary table of related TEAEs by SOC and PT.
- A summary table of TEAEs leading to discontinuation of Study Drug by SOC and PT.
- A summary table of serious TEAEs by SOC and PT.
- A summary table of serious TEAEs by SOC, PT, and maximum severity.
- A summary table of related serious TEAEs by SOC and PT.
- A summary table of serious TEAEs leading to discontinuation of Study Drug by SOC and PT.
- A summary table of related serious TEAEs leading to discontinuation of Study Drug by SOC and PT.

- A summary table of TEAEs by SOC, PT and time from first dose to event onset (< 6 months, ≥ 6 months to < 12 months, ≥ 12 months to < 18 months, ≥ 18 months to < 24 months, ≥ 24 months to < 30 months, ≥ 30 months).
- A summary table of most common TEAE with KM% of 5% or greater within any treatment groups at PT level.
- A summary table of most common TEAE with KM% of 5% or greater in ION-682884 45mg arm and 2% greater than placebo arm at PT level.
- A summary of deaths by SOC and PT.
- A listing of all deaths.
- A listing of AEs and SAEs.

The KM% with 95% CI at Week 140 in each treatment group and the difference in KM% with 95% CI between the treatment groups will be provided and considered as the primary summary interpretation for frequency of adverse events.

All TEAEs will be summarized for subgroups defined by the following variables: Age, Sex, Race, Region, ATTR mutation status, current treatment with tafamidis at baseline and NYHA Classification.

Narratives of deaths, SAEs, including early withdrawals from ION‑682884 or placebo and from study due to AE, will also be provided.

4.10.2. Other Significant Adverse Events

4.10.2.1. Adverse Events of Special Interest (AESI)

Adverse events of special interest (AESI), including both serious or non-serious events, is one of scientific and medical concern specific to the Sponsor's product or program, for which ongoing monitoring and rapid communication by the Investigator to the Sponsor could be appropriate. AESI, if defined in the study protocol, are required to be reported by the Investigator to the Sponsor immediately, no more than 24 hours of the Investigator's first knowledge of the event. They are not necessarily those expected to be related to study drug or those most frequently occurring.

For the purpose of this study, severe reductions in platelet count 25 - < 50 × 10^9/L accompanied by major bleeding events (MB) or clinically relevant, non-major bleeding events (CRNMB), or platelet count of < 25 × 10^9/L independent of a MB or CRNMB event are considered as AEs of special interest. AESI will be summarized and listed in the SS.

Each AESI category and the preferred terms within will be further summarized by the following, with the number and percentage of patients, KM% with 95% CI and the difference in KM% with 95% CI provided.

- Maximum severity (mild, moderate, severe)
- Seriousness (death, severe)
- Leading to discontinuation of Study Drug

- Related to Study Drug
- Leading to Study Drug interruption
- Resolved/Recovered

4.10.2.2. Other Adverse Events of Interest

The following list includes other adverse events that have been identified to be of interest:

**Other AEs of Interest** (Medical Concept)	**Case Definition** [a]
Renal Impairment	AE with PT within the SMQ (Broad search): Acute renal failure
Glomerulonephritis	AE with PT: Nephritis; or<br>AE with PT: Glomerulonephritis; or<br>AE with PT: Glomerulonephritis proliferative; or<br>AE with PT: Glomerulonephritis acute; or<br>AE with PT: Glomerulonephritis rapidly progressive; or<br>AE with PT: C3 Glomerulonephritis; or<br>AE with PT: Chronic autoimmune glomerulonephritis; or<br>AE with PT: Glomerulonephritis chronic; or<br>AE with PT: Fibrillary glomerulonephritis; or<br>AE with PT: Glomerulonephritis membranoproliferative; or<br>AE with PT: Glomerulonephritis membranous; or<br>AE with PT: Glomerulonephritis minimal lesion; or<br>AE with PT: Henoch-Schonlein purpura nephritis; or<br>AE with PT: Immune mediated nephritis; or<br>AE with PT: Immunotactoid glomerulonephritis; or<br>AE with PT: Lupus nephritis; or<br>AE with PT: Nephritis allergic; or<br>AE with PT: Nephrotic syndrome
Ocular adverse events potentially related to Vitamin A deficiency	AE with PTs within the Standardized MedDRA query SMQ-(broad search): Optic nerve disorders; or<br>AE with PTs within the SMQ (broad search): Corneal disorders; or<br>AE with PTs within the SMQ (broad search): Retinal disorders
Thrombocytopenia (All grades)	AE with PT: Thrombocytopenia; or<br>AE with PT: Platelet count decreased
Abnormal liver function	AE with PTs within the SMQ: Drug related hepatic disorders -comprehensive search
Adverse events at the injection site	AE with PTs within HLT: Injection site reactions; or<br>AE with PTs within HLT: Administration site reactions NEC
Hemorrhages	AE with PTs within the SMQ: Hemorrhages
AV Block	AE with the PT:<br>• Atrioventricular block<br>• Atrioventricular block complete<br>• Atrioventricular block first degree<br>• Atrioventricular block second degree
Hypersensitivity	AEs with PT in Hypersensitivity SMQ (Broad search)
Immune mediated effects or Vasculitis adverse events	AEs with PT in Immune- mediated/autoimmune disorders SMQ (Broad search)<br>AEs with PT in Vasculitis SMQ (Broad search)

[a] Defined as any subject who had received at least one dose of study drug/treatment and had experienced adverse events that fall within the criteria as listed in the column.

Hemorrhages results will be further classified by type of event, using the following categories: actual bleeds, hematomas/subdermal bleeds, investigations SOC (test result), and conditions associated with acquired alterations in hemostasis. Events classified as actual bleeds or hematomas/subdermal bleeds will be further categorized by whether the event occurred at injection site or not at injection site.

Each medical concept and the preferred terms within will be further summarized by the following, with the number and percentage of patients, KM% with 95% CI and the difference in KM% with 95% CI provided.

- Maximum severity (mild, moderate, severe)
- Seriousness (death, severe)
- Leading to discontinuation of Study Drug
- Related to Study Drug
- Leading to Study Drug interruption
- Resolved/Recovered

4.10.3. Clinical Laboratory Evaluation

Laboratory tests to ensure patient safety include chemistry panel, complete blood count (CBC) with differential, thyroid panel, coagulation panel and urinalysis. Absolute values, change and percent change from baseline will be summarized by visit and treatment group for continuous laboratory tests. No statistical comparisons between treatments will be conducted for these summaries. Except platelet, only central lab data will be summarized in table and figure. For platelet, results from central and local laboratories will be included.

The following urinalysis parameters are continuous: specific gravity, UACR, UPCR, albumin, creatinine, and protein quantitative (mg/dL).

Additional investigation of hepatobiliary laboratory assessments, platelet counts, and renal parameters will be performed, and are detailed in the sections below.

4.10.3.1. Hepatobiliary Laboratory Abnormalities

The number and percentage of patients, KM% with 95% CI at Week 140 and the difference in KM% with 95% CI falling in each of the following categories based on post-baseline assessments will be provided. The categories listed will also be analyzed using Aspartate aminotransferase (AST) in place of Alanine aminotransferase (ALT).

- ALT > 3 × ULN
- ALT > 5 × ULN
- ALT > 8 × ULN
- ALT > 10 × ULN
- ALT > 20 × ULN

- ALT > 3 × ULN - ≤ 5 × ULN
- ALT > 5 × ULN - ≤ 10 × ULN
- ALT > 10 × ULN - ≤ 20 × ULN
- ALT > 3 × ULN or ALT > 2 × baseline if baseline > ULN
- Total bilirubin > 2 × ULN
- INR > 1.5
- ALT > 3 × ULN at any time during post-Baseline or AST > 3 × ULN at any time during post-baseline (or > 2 × Baseline if the Baseline value was > ULN) and (ii) TBL > 2 × ULN or INR > 1.5 at any time during Post-Baseline
- ALT > 3 × ULN at any time during post-baseline or AST > 3 × ULN at any time during post-baseline
  - And with TBL > 2 × ULN at any time during post-baseline
  - And with TBL > 2 × ULN at any time during post-baseline and ALP < 2 × ULN at any time during post-baseline, which is Potential Hy's Law

For patients that had ALT elevation > 3 × ULN, the time from first dose to first ALT elevation > 3 × ULN will be summarized using the following descriptive statistics: mean, standard deviation, median, 25th percentile (P25), 75th percentile (P75), and minimum and maximum.

Shift tables from baseline for ALT, AST, and total bilirubin based on peak (maximum) category will also be provided. Categories for the ALT and AST shift tables will be < = 3 × ULN, > 3 × ULN to ≤ 5 × ULN, > 5 × ULN to ≤ 8 × ULN, and > 8 × ULN. Categories for the total bilirubin shift table will be <= 2 × ULN and > 2 × ULN.

4.10.3.2. Platelets

The number and percentage of patients KM% with 95% CI at Week 140 and the difference in KM% with 95% CI falling in each of the following categories (using available central and local laboratory assessments) based on single post-baseline assessments will be provided:

- Platelet count - Grade 1a [≥ 100 × 10^9/L to < 140 × 10^9/L]
- Platelet count - Grade 1b [≥ 75 × 10^9/L to < 100 × 10^9/L]
- Platelet count - Grade 2 [≥ 50 × 10^9/L to < 75 × 10^9/L]
- Platelet count - Grade 3 [≥ 25 × 10^9/L to < 50 × 10^9/L]
- Platelet count - Grade 4 [< 25 × 10^9L]
- Platelet count < 140 × 10^9/L
- Platelet count < 100 × 10^9/L
- Platelet count < 75 × 10^9/L
- Platelet count < 50 × 10^9/L

- Maximum toxicity grade (Grade 1a, Grade 1b, Grade 2, Grade 3, Grade 4) based on the worst post-baseline value
- Platelet decrease from baseline ≥ 30%
- Platelet count < 140 × 10^9/L and ≥ 30% decrease from baseline
- Platelet decrease from baseline ≥ 50%
- Platelet count < 140 × 10^9/L and ≥ 50% decrease from baseline

Platelet count grades are based on interactions with a regulatory authority and align with the definitions from the National Cancer Institute Common Terminology Criteria for Adverse Events (CTCAE) version 5.0 (except that the CTCAE does not subdivide Grade 1 into Grade 1a and 1b).

Incidence of abnormalities of platelet test results at consecutive planned visit will be summarized, by category (> ULN and < LLN; ULN and LLN are based on central lab), and treatment group. Shift tables from baseline will be provided using the nadir value. Categories for the shift table will be ≥ 140 × 10^9/L, ≥ 100 × 10^9/L to < 140 × 10^9/L, ≥ 75 × 10^9/L to < 100 × 10^9/L, ≥ 50 × 10^9/L to < 75 × 10^9/L, ≥ 25 × 10^9/L to < 50 × 10^9/L, and < 25 × 10^9/L.

For each of the platelet categories investigated, time from first dose to the onset will be summarized for the patients that met the criterion using the following descriptive statistics: mean, standard deviation, median, P25, P75, and minimum and maximum. Kaplan-Meier plots for time to first event will be provided for value < 100 × 10^9/L.

Duration of platelet counts below 140 × 10^9/L or 100 × 10^9/L will be summarized, based on the duration (in weeks) each patient was below 140 × 10^9/L or 100 × 10^9/L.

The post-baseline nadir (absolute value, change from baseline, and percent change from baseline) of platelet count will be summarized by treatment group. Mean platelet counts over time will also be plotted.

4.10.3.3. Renal Parameters

The number and percentage of patients, KM% with 95% CI at Week 140 and the difference in KM% with 95% CI falling in each of the following categories (using available central laboratory assessments) based on post-baseline assessments will be provided:

- Estimated Glomerular Filtration Rate (eGFR) by Chronic Kidney Disease Epidemiology Collaboration (CKD-EPI) ≥ 25% decrease from baseline
- eGFR by CKD-EPI ≥ 50% decrease from baseline
- eGFR by CKD-EPI < 45 mL/min/1.73 m^2 separately for patients with baseline > 60 mL/min/1.73 m^2 and for patients with baseline ≤ 60 mL/min/1.73 m^2
- eGFR by CKD-EPI < 30 mL/min/1.73 m^2 separately for patients with baseline > 60 mL/min/1.73 m^2 and for patients with baseline ≤ 60 mL/min/1.73 m^2
- eGFR by CKD-EPI < 15 mL/min/1.73 m^2 separately for patients with baseline > 60 mL/min/1.73 m^2 and for patients with baseline ≤ 60 mL/min/1.73 m^2
- UPCR > 3 × Baseline and > 1000 mg/g

- UPCR > 5 × Baseline and > 1500 mg/g
- UPCR ≥ 1000 mg/g
- UPCR ≥ 2000 mg/g
- UPCR > 5 × ULN
- UACR > 5 × ULN
- Serum Creatinine Increase > 44.2 µmol/l (0.5 mg/dL) from Baseline

Note: eGFR will be based on CKD-EPI formula (Levey *et al.* 2009) reported in central lab data. For Japanese patients, eGFR results reported in central lab are based on modified 2009 formular, hence these results will be converted to CKD-EPI formula (Levey *et al.* 2009) and included in the analysis.

Incidence of abnormalities of eGFR by CKD-EPI at consecutive visits will be summarized by analysis visit, category (≥ 25% Decrease from Baseline), and treatment group. Shift tables from Baseline for eGFR by CKD-EPI will be provided using the nadir value.

- Categories for the shift table will be ≥ 90 mL/min/1.73 m^2, ≥ 60 mL/min/1.73 m^2 to < 90 mL/min/1.73 m^2, ≥ 30 mL/min/1.73 m^2 to < 60 mL/min/1.73 m^2, ≥ 15 mL/min/1.73 m^2 to < 30 mL/min/1.73 m^2, and < 15 mL/min/1.73 m^2.

The post-baseline eGFR (absolute value, change from baseline, and percent change from baseline) by CKD-EPI will be summarized by treatment group. Mean eGFR by CKD-EPI over time will also be plotted.

4.10.4. Vital Signs, Physical Findings and Other Observations Related to Safety

Vital signs include systolic and diastolic blood pressure, pulse, respiratory rate, and temperature. Weight will also be analyzed. Absolute values at each visit, change and percent change from baseline to each visit in vital signs and weight will be summarized by treatment group. No statistical comparisons between treatments will be performed for this summary.

Shift table from Baseline to the highest value and lowest value at any visit will be presented, respectively.

- Systolic blood pressure: < 90 mmHg, ≥ 90 to ≤ 140 mmHg, > 140 to ≤ 160 mmHg, > 160 mmHg
- Diastolic blood pressure: < 50 mmHg, ≥ 50 to ≤ 90 mmHg, > 90 to ≤ 100 mmHg, > 100 mmHg
- Pulse rate: < 60 bpm, ≥ 60 to ≤ 100 bpm, > 100 bpm

The number and percent of patients meeting any one of the following criteria will be summarized by visit and any visit.

- Body weight: decrease of ≥ 7% from Baseline and increase of ≥ 7% from Baseline
- Temperature: > 38.0 °C, < 36.0 °C
- Respiratory rate: < 12 breaths/min, > 20 breaths/min

- Systolic Blood Pressure: < 90 mmHg, >140 mmHg, > 160 mmHg
- Diastolic Blood Pressure: < 50 mmHg, >90 mmHg, > 100 mmHg
- Pulse Rate: < 60 bpm, > 100 bpm

All vital signs and weight will be listed.

4.10.5. 12-Lead Electrocardiograms (12-Lead ECG)

Absolute values, change and percent change from baseline in ventricular rate and ECG intervals (PR, RR, QRS, QT, QT interval corrected for heart rate [QTc] and QTc interval calculated using Fridericia's formula [QTcF]) will be presented by treatment and visit.

Shift table from baseline to the worst (highest) post-baseline value by treatment group will be used to assess the change in the QTcF interval. The categories for the shift table will be: ≤ 450 msec, > 450 msec to ≤ 480 msec, > 480 msec to ≤ 500 msec, and > 500 msec. The number and percent of patients experiencing an increase from baseline in QTcF interval of greater than 30 msec or 60 msec at any time post-baseline will be summarized by treatment group. This analysis will be presented overall and also by the subgroup whose QTcF was normal or abnormal at baseline. Normal QTcF will be defined as ≤ 450 msec for males or ≤ 470 msec for females. The number and percent of patients with overall qualitative ECG abnormalities will also be summarized. No statistical comparisons between treatments will be performed for these summaries.

Ventricular rate and ECG intervals (PR, RR, QRS, QT, QTcB and QTcF), as well as treatment-emergent abnormalities will be listed.

The number and percent of patients with QRS > 160 ms and > 25% above Baseline will be summarized by visit and any visit.

4.10.6. Ocular Questionnaire

The number and percentage of patients of ocular questionnaire will be summarized by visit and treatment group. Shift tables from baseline in ocular questionnaire will be also summarized by visit and treatment group.

The number and percent of patients that stopped treatment because they met a protocol-defined stopping rules for vitamin A deficiency confirmed by ophthalmologist and discussed with Medical Monitor will be tabulated by treatment group.

4.10.7. Safety Monitoring (Data and Safety Monitoring Board [DSMB])

DSMB will be assembled and meet periodically to review safety, tolerability and efficacy (as needed) data collected of unblinded data collected during the trial.

Details on the safety assessments, frequency of review, meeting schedules and controlled access to unblinded data are outlined in the DSMB Charter.

4.11. Exploratory Analyses

4.11.1. Exploratory Endpoints Analysis

Exploratory analyses for the comparisons of change from Baseline at each post-baseline timepoint include but not limited to the following parameters between ION-682884 or placebo treatment groups based on FAS, unless otherwise specified:

- Echocardiogram parameters of interest. Key exploratory parameters: Global longitudinal strain, Mean Left Ventricle Wall Thickness, stroke volume, and diastolic function (as measured by the lateral E/e' ratio). Additional echocardiogram parameters specified in Section 3.2.4. Select parameters will be presented indexed to body surface area, using the Mosteller formula and calculated from baseline height and body weight: BSA (m^2) = sqrt[height (cm) × weight (kg) / 3600]. The indexed parameter is calculated by dividing un-indexed parameter (e.g., 'left ventricle mass') by the value of the BSA. The following 4 parameters will have indexed versions reported: Left Ventricular End Diastolic Volume Mean, Left Ventricular End Systolic Volume Mean, Left Ventricular Mass, Left Atrial Volume Max.
- Renal function (eGFR)
- Serum biomarkers including (NT-proBNP, hs-cTnT, CK-MB, Galectin-3, sST2, TTR)
- NYHA class, KPS, SF-36, EQ-5D-5L, Norfolk QOL-DN, PGIS and PGIC (PGIS and PGIC will be assessed for purposes of psychometric analyses of selected clinical outcome assessment measures)
- PND Score, COMPASS-31, LLF and 10-meter walk test (only in patients with hereditary ATTR-CM).
- mBMI

Descriptive statistics will be provided for absolute values and change from baseline by visit for continuous exploratory endpoints.

Change from baseline as listed above including echocardiogram parameters, eGFR, serum biomarkers, KPS, SF-36 PCS and MCS, EQ-5D-5L index, EQ VAS, Norfolk QOL-DN total score, COMPASS-31 score, LLF total score, and 10-meter walk test, mBMI will be analyzed using a Mixed Effects Model with Repeated Measures (MMRM) approach. The MMRM model will include the effects of treatment (ION-682884 or placebo), time (categorical), 4 stratification factors (for COMPASS-31 score, LLF total score, and 10-meter walk test only performed in patients with hereditary ATTR-CM, ATTR mutation status will be not included as a covariate in the analysis), treatment-by-time interaction, and baseline value of the endpoint. The MMRM model for change from baseline in eGFR will additionally include baseline log(UACR) and its interaction with time as a covariate. Percent change from baseline in NT-proBNP, TTR, and hs-cTnT will be analyzed using the same MMRM approach. In addition, change from baseline in log-transformed NT-proBNP, and log-transformed hs-cTnT will be summarized and analyzed using a similar MMRM approach, where log-transformed baseline NT-proBNP and hs-cTnT will be included in the models respectively. A subgroup analysis will be performed for TTR using the

subgroup categories defined in Section 4.9.2.7 as well as body weight (< 65 kg, > = 65 kg) as an additional subgroup variable.

In the MMRM approach, an unstructured covariance matrix will be used to model the within-patient errors, and the degrees of freedom will be determined using Kenward-Roger method (Kenward & Roger 1997). In case of convergence issues with the model, the 6 methods below will be conducted in the following sequence to solve the convergency issues.

1. Fisher's scoring in the first 4 iterations (SCORING = 4 option PROC MIXED statement) in SAS.
2. TYPE = FA0(t) option will be used where t is the dimension of covariance matrix.
3. A heterogeneous compound symmetry covariance structure (TYPE = CSH) will be used with robust "sandwich" estimator for the standard errors of the fixed effects parameters.
4. A heterogeneous first-order autoregressive AR(1) structure (TYPE = ARH(1)) will be used with robust "sandwich" estimator for the standard errors of the fixed effects parameters.
5. A homogeneous compound symmetry covariance structure (TYPE = CS) will be used with robust "sandwich" estimator for the standard errors of the fixed effects parameters.
6. A homogeneous first-order autoregressive AR(1) structure (TYPE = AR(1)) will be used with robust "sandwich" estimator for the standard errors of the fixed effects parameters.

Shift tables will be provided for NYHA class, PND, and PGI-S. Furthermore, changes in these variables at Week 140 and at the worst post-baseline visit up to Week 140 will be categorized to two categories: stable or improved versus worsened, and summarized and analyzed using a Cochran-Mantel-Haenszel method stratified by baseline NT-proBNP group (≤ 3000; > 3000 ng/L) and current tafamidis use (Y/N) at baseline. NYHA class after death including heart transplantation and combined heart and liver transplantation, intermediate or long term cardiac mechanical circulatory support (i.e., ventricular assist device or total artificial heart) will be imputed as NYHA class IV in the summary and analysis. Similarly, PND, PGI-S, and PGI-C will be imputed to the worst possible values in these cases.

An analysis of the effect of ION-682884 on the linear rate of change from baseline in eGFR over time (eGFR slope) will be performed using a shared parameter (SP) model (Vonesh *et al.* 2019). The SP model will jointly model eGFR slope with a mixed model and time to all-cause death with a survival (Weibull) model using a shared random effect. Randomization stratification factors, standardized baseline eGFR and its interaction with time, and standardized log (baseline UACR) and its interaction with time will be included as covariates. Parameter estimates will be obtained with maximum likelihood estimation using PROC NLMIXED. For details see the appendix.

Change in eGFR will additionally be analyzed as change from baseline in CKD stage at Week 140, analyzed as stable or improved versus worsening CKD-stage at Week 140 using the Cochran-Mantel-Haenszel method as described for NYHA class and imputing using the worst value (Stage 5) after death including heart transplantation and combined heart and liver transplantation, intermediate or long term cardiac mechanical circulatory support.

CKD Stage is defined as below, where later stages are ‘worse’:

- Stage 1: eGFR ≥ 90 mL/min/1.73 m^2
- Stage 2: eGFR ≥ 60 and < 90 mL/min/1.73 m^2
- Stage 3a: eGFR ≥ 45 and < 60 mL/min/1.73 m^2
- Stage 3b: eGFR ≥ 30 and < 45 mL/min/1.73 m^2
- Stage 4: eGFR ≥ 15 and < 30 mL/min/1.73 m^2
- Stage 5: eGFR < 15 mL/min/1.73 m^2

4.11.2. Immunogenicity (IM) Analysis

Samples collected for IM assessment at baseline (Day 1 pre-dose), during treatment and post-treatment follow-up period including early termination samples will be analyzed for anti-ION-682884 antibodies (ADA).

4.11.2.1. Sample Level ADA Data

An evaluable sample will be designated ‘Positive’ based on both positive screening and confirmation assay results (i.e., confirmed positive result), and otherwise will be deemed ‘Negative’. An unevaluable sample, for example, a sample not being stored properly, or insufficient volume for analysis, will be reported as “unknown”. Sample ADA results (screen positive/negative, confirmed positive/negative or unknown, and when applicable, titer of anti-ION-682884 antibodies) before, during, and after treatment with study drug (ION-682884 or placebo) (sample ADA status) will be listed by treatment, dose, and day of collection.

The sample ADA incidence (number) and incidence rate (percent) at each evaluated study time point will be determined and appropriately summarized by treatment and dose as the total number of and percentage of evaluated subjects with sample ADA negative, positive, and unknown status. Furthermore, titer over time will be also appropriately summarized (using descriptive statistics) as median, quartiles (25% and 75%), and range by treatment and dose.

4.11.2.2. Subject Level ADA Data

Subject ADA status overall (ADASTAT) will be defined as ‘Positive’ status if they have at least one confirmed positive sample result at any time during the treatment or post-treatment evaluation periods; ‘Negative’ status if all evaluated ADA sample results during the treatment and post-treatment evaluation periods are ADA negative and they have at least one evaluable ADA result collected post study drug treatment. Otherwise, a study subject will be assigned ‘Unknown’ ADA status.

Furthermore, subjects with positive overall ADA status will be further classified into different ADA types (ADATYPE) based on their baseline ADA status and change in ADA titer post treatment as described below (Shankar *et al.* 2014):

- Treatment-Emergent ADA: subjects with either treatment-induced ADA or treatment-boosted ADA as described below:

  - Treatment-Induced ADA: ADA developed de novo (seroconversion) following study drug administration (i.e., formation of ADA any time after the initial drug administration in a subject without pre-existing ADA, i.e., baseline negative ADA)
  - Treatment-Boosted ADA: pre-existing ADA that were boosted to a higher level following study drug administration (i.e., any time after the initial drug administration the ADA titer is greater than the baseline titer by a factor of 8-fold or more
- Treatment-Unaffected ADA: pre-existing ADA that were not affected (boosted) following study drug administration (i.e., any time after the initial drug administration the ADA titer is 4-fold or less)

For subjects with negative overall ADA status, ADATYPE would be classified as baseline negative or baseline positive, based on their baseline ADA status. Other subject level IM parameters to be calculated/defined may include but not limited to:

- ADA category (ADACAT) will include subjects with Negative, Treatment-Unaffected, and Treatment-Emergent ADA status as described above.
- Subject ADA Status at Baseline (ADASTATB): "Positive" if the subject has Week 1 Day 1 pre-dose sample (baseline) tested as confirmed positive; "Negative" if the subject has Week 1 Day 1 pre-dose sample (baseline) tested as confirmed negative; "Unknown" if the subject has Week 1 Day 1 pre-dose sample (baseline) unevaluable.
- First day of ADA detected (TFSTADA): i.e., the first day ADA positive sample observed, will be calculated by: the date of first sample has "positive" sample IM status - first dose date +1
- Onset of treatment-emergent ADA (ONSETTE): i.e., the first day treatment-emergent ADA positive sample observed, will be calculated by: the date of first treatment-emergent ADA observed - first dose date +1. This parameter will be calculated for subjects with treatment-induced ADA and treatment-boosted ADA.
- Last Positive ADA Study Day (TLSTADA): defined as the last positive ADA sample observed from the start of study drug treatment and will be calculated by: the date of last sample has "positive" sample IM status - first dose date +1
- Last IM Sampling Study Day (TLSTSAMP): defined as the last ADA sample collected from the start of study drug treatment and will be calculated by: the date of last sample collected - first dose date +1
- Duration of treatment-emergent ADA (DURTE): i.e., the total duration of treatment-emergent ADA during the study, and will be calculated using the last sample result carried forward. This parameter will be calculated for subjects with treatment-induced ADA and treatment-boosted ADA.
- Peak titer (PEAKTIT): the highest titer observed for the subject; peak titer of patients with treatment-emergent ADA and treatment-unaffected ADA will also be summarized separately.

- Peak Titer Quartiles (PEAKTITQ): the quartiles (Q1-Q4) for peak titer in subjects with treatment-emergent ADA will be calculated using PEAKTIT value per subject with treatment-emergent ADA status in the ION-682884 arm population; "Negative" if Negative ADA status; “Treatment-Unaffected” if Treatment-Unaffected ADA status
- Time to peak titer (TPEAKTIT): the time to reach peak titer will be calculated by the date of first peak titer observed- first dose date +1; time to peak titer of patients with treatment-emergent ADA and treatment-unaffected ADA will also be summarized separately.
- Total number of ADA Positive Samples (NOPOSAMP): the total number of ADA samples being confirmed positive for the subject
- Total number of ADA Samples evaluated (NOADASAMT): the total number of ADA samples being collected and analyzed successfully with reportable results for the subject
- For subjects with missing or unknown baseline ADA status, baseline ADA status will be treated as “negative” in determining subject level IM parameters (e.g., treatment- induced ADA).

Lastly, subjects with positive ADA status may further be classified as being transient or persistent ADA response, if there are sufficient number of subjects with transient ADA status. Transient and persistent ADA definitions are defined below and based on (Shankar *et al.* 2014).

Transient ADA response:

- Treatment-induced ADA detected only at one sampling time point during the treatment or follow-up observation period (excluding the last sampling time point, which will be considered persistent unless shown to be undetectable at a later time) or
- Treatment-induced ADA detected at two or more sampling time points during the treatment (including follow-up period if any), where the first and last ADA positive samples (irrespective of any negative samples in between) are separated by a period less than 16 weeks, and the subject’s last sampling time point is ADA-negative.

Persistent ADA response:

- Treatment-induced ADA detected at two or more sampling time points during the treatment (including follow-up period if any), where the first and last ADA positive samples (irrespective of any negative samples in between) are separated by a period of 16 weeks or longer or
- Treatment-induced ADA detected only at the last sampling time point of the study treatment period or at a sampling time point with less than 16 weeks before an ADA-negative last sample.

The subject level ADA prevalence, incidence, and positive ADA response being transient or persistent (if applicable) will be calculated as the number and the proportion (percent) of the study population during the study period by treatment and dose. Subject level IM parameters

(as described above) will be listed by treatment and dose for all evaluable subjects, and also appropriately summarized (using descriptive statistics) as median, quartiles (25% and 75%), and range by treatment and dose. Demographic and other baseline characteristics by subject ADA category (ADACAT) and peak ADA titer quartiles (PEAKTITQ) will also be summarized.

4.11.2.3. Evaluation of IM Impact on PK, PD, Efficacy and Safety

The impact of IM on PK, PD, and safety will be evaluated by stratifying plasma trough and post-treatment ION-682884 concentrations, PD biomarker (TTR) levels, selected clinical efficacy end points and safety measures by subject level ADA data, summarized using typical descriptive statistics, and presented graphically and/or in tables. Efficacy measures to be stratified by subject level ADA data will include but may not be limited to CV-death and CV clinical events. Safety measures to be stratified by subject level ADA data will include but may not be limited to AEs, and lab tests for hematology, liver and kidney functions. Subject level ADA data for evaluation of IM on PK, PD, efficacy, and safety will include, but not limited to subject ADA category (ADACAT) and peak ADA titer quartiles (PEAKTITQ) as defined in Section 4.11.2.2.

Other stratifications (e.g., based on onset of ADA, etc.) of selected PK, efficacy and safety assessments may also be performed if deemed warranted at the discretion of the pharmacokineticist, medical monitor, and/or biostatistician.

4.12. Other Analyses

4.12.1. Pharmacokinetics

Plasma concentrations of ION-682884 (measured as total full-length oligonucleotides or ION-682884-equivalent, i.e., ION-682884-eq, including fully conjugated, partially conjugated, and unconjugated ION-682884), along with the scheduled (nominal) time point will be listed in bioanalytical report (Report 682884-CS02BA01).

For all patients who receive ION-682884 treatment, ION-682884 plasma trough and post-treatment concentrations will be summarized using descriptive statistics by treatment, dose, study day, and scheduled time point, without and with stratification by subject level ADA data (see Section 4.11.2). For the purpose of calculating typical summary descriptive statistics (n, mean, SD, SE, %CV, geometric mean, geometric %CV, median, minimum, and maximum) for plasma concentrations, all BLQ values will be set to zero. Mean plasma concentrations that are below the LLOQ will be presented as BLQ, and the SD, SE, %CV, and geometric %CV will be reported as not applicable. Other stratifications may also be performed if deemed warranted. Samples will be excluded from descriptive statistics if there are large deviations between scheduled and actual sampling times (percent difference between scheduled and actual sampling time greater than 30%), or large deviations between actual dose and nominal dose (percent difference between nominal and actual dose greater than 30%). Any samples excluded from the summary descriptive statistics, if deemed necessary, will be flagged in the analysis dataset (ADaMs) with the reason for exclusion.

Population PK, PK/PD, and exposure-response analysis may be performed using the PK, PD, and safety data from this study, and/or combined with other clinical study data and reported separately in the future.

4.13. Determination of Sample Size

The sample size calculation assumes the following:

- CV death rate of 8.7% for the placebo group during the 140-week period
- 0.476 CV clinical events per patient for the placebo group during the 140-week period
- Significance level (alpha) of 0.05 (two-sided test)
- Treatment duration up to 140 weeks

The sample size calculation assumes a treatment effect of 20% reduction in the relative risk of CV death and recurrent CV clinical events in the treatment arm compared with the placebo arm over a period up to 140 weeks. Using a two-sided test with an alpha level of 0.05, a sample size of 1432 will provide a power about 85% with treatment duration of up to 140 weeks, considering a 15% drop out rate, and 10% incidence of death.

4.14. Changes in the Conduct of the Study or Planned Analysis

No change made to the planned analyses in the Study Protocol.

5. REFERENCES

Andersen, P. K., & Gill, R. D. (1982). Cox's Regression Model for Counting Processes: A Large Sample Study. *The Annals of Statistics*, **10**(4). doi:10.1214/aos/1176345976

Cowling, B. J., Hutton, J. L., & Shaw, J. E. H. (2006). Joint modelling of event counts and survival times. *Journal of the Royal Statistical Society: Series C (Applied Statistics)*, **55**(1), 31–39.

Fine, J. P., & Gray, R. J. (1999). A Proportional Hazards Model for the Subdistribution of a Competing Risk. *Journal of the American Statistical Association*, **94**(446), 496–509.

Hicks, K. A., Mahaffey, K. W., Mehran, R., … Trials, I. S. D. C. for C. (2018). 2017 Cardiovascular and Stroke Endpoint Definitions for Clinical Trials. *Circulation*, **137**(9), 961–972.

Kenward, M. G., & Roger, J. H. (1997). Small sample inference for fixed effects from restricted maximum likelihood. *Biometrics*, **53**(3), 983–97.

Levey, A. S., Stevens, L. A., Schmid, C. H., … Ckd, E. P. I. (2009). A new equation to estimate glomerular filtration rate. *Ann Intern Med*, **150**(9), 604–12.

Lin, D. Y., Wei, L., Yang, I., & Ying, Z. (2000). Semiparametric regression for the mean and rate functions of recurrent events. *Journal of the Royal Statistical Society Series B*, **62**(4), 711–730.

Shankar, G., Arkin, S., Cocea, L., … Yim, S. (2014). Assessment and reporting of the clinical immunogenicity of therapeutic proteins and peptides-harmonized terminology and tactical recommendations. *Aaps j*, **16**(4), 658–73.

Vonesh, E., Tighiouart, H., Ying, J., … Greene, T. (2019). Mixed-effects models for slope-based endpoints in clinical trials of chronic kidney disease. *Statistics in Medicine*, **38**(22), 4218–4239.

6. APPENDIX

Appendix 6.1 Kansas City Cardiomyopathy Questionnaire

Table 1: KCCQ Domains and Scoring

Domain	Question	Scale
Physical Limitation (Q:1a-f)	Dressing yourself	Extremely Limited=1;<br>Quite a bit Limited=2;<br>Moderately Limited=3;<br>Slightly Limited=4;<br>Not at all Limited=5;<br>Limited for other reasons or did not do the activity=missing.
	Showering/Bathing	
	Walking 1 block on level ground	
	Doing yardwork, housework or carrying groceries	
	Climbing a flight of stairs without stopping	
	Hurrying or jogging	
Symptom Stability (Q:2)	My symptoms of heart failure have become…	Much worse=1;<br>Slightly worse=2;<br>Not changed=3;<br>Slightly better=4;<br>Much better=5;<br>I've had no symptoms over the last 2 weeks=3
Symptoms Frequency (Q:3,5,7,9)	How many times did you have swelling in your feet, ankles or legs when you woke up in the morning	Every morning=1;<br>3 or more times a week, but not every day=2;<br>1-2 times a week=3;<br>Less than once a week=4;<br>Never over the past 2 weeks=5.
	How many times has fatigue limited your ability to do what you want	All of the time=1;<br>Several times per day=2;<br>At least once a day=3;<br>3 or more times per week but not every day=4;<br>1-2 times per week=5;<br>Less than once a week=6;<br>Never over the past 2 weeks=7.
	How many times has shortness of breath limited your ability to do what you wanted	
	How many times have you been forced to sleep sitting up in a chair or with at least 3 pillows to prop you up because of shortness of breath	Every night=1;<br>3 or more times per week but not every day=2;<br>1-2 times per week=3;<br>Less than once a week=4;<br>Never over the past 2 weeks=5.
Symptoms Burden (Q:4,6,8)	How much has swelling in your feet, ankles or legs bothered you	Extremely bothersome=1;<br>Quite a bit bothersome=2;<br>Moderately bothersome=3;
	How much has your fatigue bothered you	

Domain	Question	Scale
	How much has your shortness of breath bothered you	Slightly bothersome=4;<br>Not at all bothersome=5;<br>I've had no swelling/fatigue/shortness of breath=5.
Self-efficacy<br>(Q:10,11)	Heart failure symptoms can worsen for a number of reasons. How sure are you that you know what to do, or whom to call, if your heart failure gets worse	Not at all sure=1;<br>Not very sure=2;<br>Somewhat sure=3;<br>Mostly sure=4;<br>Completely sure=5.
	How well do you understand what things you are able to do to keep your heart failure symptoms from getting worse	Do not understand at all=1;<br>Do not understand very well=2;<br>Somewhat understand=3;<br>Mostly understand=4;<br>Completely understand=5.
Quality of Life<br>(Q:12,13,14)	How much has your heart failure limited your enjoyment of life	It has extremely limited my enjoyment of life=1;<br>It has limited my enjoyment of life quite a bit=2;<br>It has moderately limited my enjoyment of life=3;<br>It has slightly limited my enjoyment of life=4;<br>It has not limited my enjoyment of life at all=5.
	If you had to spend the rest of your life with your heart failure the<br>way it is right now, how would you feel about this?	Not at all satisfied=1;<br>Mostly dissatisfied=2;<br>Somewhat satisfied=3;<br>Mostly satisfied=4;<br>Completely satisfied=5.
	How often have you felt discouraged or down in the dumps because of your heart failure	I felt that way all of the time=1;<br>I felt that way most of the time=2;<br>I occasionally felt that way=3;<br>I rarely felt that way=4;<br>I never felt that way=5.
Social Limitation<br>(Q:15a-d)	Hobbies, recreational activities	Severely limited=1;<br>Limited quite a bit=2;<br>Moderately limited=3;<br>Slightly limited=4;<br>Did not limit at all=5;<br>Does not apply or did not do for other reasons=missing.
	Working or doing household chores	
	Visiting family or friends out of your home	
	Intimate relationships with loved ones	

Table 2: Algorithm of KCCQ Scoring

Domain	Question	Algorithm
Physical Limitation	Question 1	Scale scores can be computed providing at least 3 items are not missing.<br>If ≥ 3 items are not missing then Physical Limitation Score = 100 × [(mean of Questions 1a-f actually answered)-1]/4.<br>Otherwise score=.
Symptom Stability	Question 2	If Question 2 is not missing then Symptom Stability Score=100×[(Question 2) - 1]/4.<br>Otherwise score=.
Symptom Frequency	Question 3,5,7,9	Scale scores can be computed providing at least 2 items are non-missing. If ≥ 2 items are not missing then<br>S3= [(Question 3)-1]/4;<br>S5= [(Question 5)-1]/6;<br>S7= [(Question 7)-1]/6;<br>S9= [(Question 9)-1]/4;<br>Symptom Frequency Score=100× (mean of S3, S5, S7, S9)<br>Otherwise score=.
Symptom Burden	Question 4,6,8	If at least one of the items is not missing then Symptom Burden Score=100× [(mean of Questions 4, 6, 8 actually answered)-1]/4<br>Otherwise score=.
Total Symptom Score	Symptom Frequency Score and Symptom Burden Score	Total Symptom Score= mean (Symptom Frequency Score, Symptom Burden Score)
Self-efficacy	Question 10,11	If at least one of the items is not missing then Self-Efficacy Score=100×[(mean of Questions 10, 11 actually answered)-1]/4<br>Otherwise score=.
Quality of Life	Question 12,13,14	If at least one of the items is not missing then Quality of Life Score=100×[(mean of Questions 12, 13, 14 actually answered)-1]/4<br>Otherwise score=.
Social Limitation	Question 15	If at least two of the items are not missing then Social Limitation Score=100×[(mean of Questions 15 a-d actually answered)-1]/4<br>Otherwise score=.
Overall Summary Score	Physical Limitation; Total Symptom Score; Quality of Life; Social Limitation	Overall Summary Score=mean (Physical Limitation, Total Symptom Score, Quality of Life, Social Limitation)
Clinical Summary Score	Physical Limitation; Total Symptom Score	Clinical Summary Score=mean (Physical Limitation, Total Symptom Score

Appendix 6.2 Composite Autonomic Symptom Score (COMPASS-31)

Table 3: COMPASS-31 Scores

Domain	Item	Answer	Points
Orthostatic Intolerance	1	1.Yes	1
		2.No	0
	2	1.Rarely	0
		2.Occasionally	1
		3.Frequently	2
		4.Almost always	3
	3	1.Mild	1
		2.Moderate	2
		3.Severe	3
	4	1.Gotten much worse	3
		2.Gotten somewhat worse	2
		3.Stayed about the same	1
		4.Gotten somewhat better	0
		5.Gotten much better	0
		6.Completely gone	0
Vasomotor	5	1.Yes	1
		2.No	0
	6	1.Hands	1
		2.Feet	1
	7	1.Getting much worse	3
		2.Getting somewhat worse	2
		3.Stayed about the same	1
		4.Getting somewhat better	0
		5.Getting much better	0
		6.Completely gone	0
Secretomotor	8	1.I sweat much more than I used to	1
		2.I sweat somewhat more than I used to	0
		3.I haven’t noticed any change	0
		4.I sweat somewhat less than I used to	1
		5.I sweat much less than I used to	2
	9	1.Yes	1
		2.No	0
	10	1.Yes	1
		2.No	0

Domain	Item	Answer	Points
	11	1.I have not had any of these symptoms	0
		2.Getting much worse	3
		3.Getting somewhat worse	2
		4.Staying about the same	1
		5.Getting somewhat better	0
		6.Getting much better	0
		7.Completely gone	0
Gastrointestinal	12	1.I get full a lot more quickly now than I used to	2
		2.I get full more quickly now than I used to	1
		3.I haven't noticed any change	0
		4.I get full less quickly now than I used to	0
		5.I get full a lot less quickly now than I used to	0
	13	1.Never	0
		2.Sometimes	1
		3.A lot of the time	2
	14	1.Never	0
		2.Sometimes	1
		3.A lot of the time	2
	15	1.Never	0
		2.Sometimes	1
		3.A lot of the time	2
	16	1.Yes	1
		2.No	0
	17	1.Rarely	0
		2.Occasionally	1
		3.Frequently	2
		4.Constantly	3
	18	1.Mild	1
		2.Moderate	2
		3.Severe	3
	19	1.Much worse	3
		2.Somewhat worse	2
		3.Staying the same	1
		4.Somewhat better	0
		5.Much better	0
		6.Completely gone	0
	20	1.Yes	1
		2.No	0

Domain	Item	Answer	Points
	21	1.Rarely	0
		2.Occasionally	1
		3.Frequently	2
		4.Constantly	3
	22	1.Mild	1
		2.Moderate	2
		3.Severe	3
	23	1.Much worse	3
		2.Somewhat worse	2
		3.Staying the same	1
		4.Somewhat better	0
		5.Much better	0
		6.Completely gone	0
Bladder	24	1.Never	0
		2.Occasionally	1
		3.Frequently	2
		4.Constantly	3
	25	1.Never	0
		2.Occasionally	1
		3.Frequently	2
		4.Constantly	3
	26	1.Never	0
		2.Occasionally	1
		3.Frequently	2
		4.Constantly	3
Pupillomotor	27	1.Never	0
		2.Occasionally	1
		3.Frequently	2
		4.Constantly	3
	28	1.Mild	1
		2.Moderate	2
		3.Severe	3
	29	1.Never	0
		2.Occasionally	1
		3.Frequently	2
		4.Constantly	3
	30	1.Mild	1
		2.Moderate	2
		3.Severe	3

Domain	Item	Answer	Points
	31	1.I have not had any of these symptoms	0
		2.Much worse	3
		3.Somewhat worse	2
		4.Staying the same	1
		5.Somewhat better	0
		6.Much better	0
		7.Completely gone	0

Appendix 6.3 EQ-5D Health State Index Score Derivation Algorithm

To derive this index value, the response of each dimension will be first translated into a weight based on the value sets issued by EuroQoL group (Table 4). The index value then can be calculated as 1- sum of weights from these 5 dimensions' response. The possible best value is 1 and the possible worst value varies by country. Validated weights for many countries are available from the EuroQol page of https://euroqol.org/support/analysis-tools/index-value-set-calculators/. If there is a missing response from a dimension, then the index value will not be calculated and be treated as missing. For countries without validated health state utility values yet, the suggestion from EuroQol group, currently, is using the United States EQ-5D-5L value set. The display of the index value is with 3 digits of decimal points.

Table 4: Weight of EQ-5D-5L Response by Region

		**EQ-5D-5L Dimension**				
Region	Response	Mobility	Self-Care	Usual Activities	Pain/ Discomfort	Anxiety/ Depression
UK	1) No problem	0	0	0	0	0
(16/11/2020)	2) Slight problem	0.058	0.050	0.050	0.063	0.078
	3) Moderate problems	0.076	0.080	0.063	0.084	0.104
	4) Severe problems	0.207	0.164	0.162	0.276	0.285
	5) Unable to	0.274	0.203	0.184	0.335	0.289
US	1) No problem	0	0	0	0	0
(01/12/2020)	2) Slight problem	0.096	0.089	0.068	0.060	0.057
	3) Moderate problems	0.122	0.107	0.101	0.098	0.123
	4) Severe problems	0.237	0.220	0.255	0.318	0.299
	5) Unable to	0.322	0.261	0.255	0.414	0.321
Germany	1) No problem	0	0	0	0	0
(17/11/2020)	2) Slight problem	0.026	0.050	0.036	0.057	0.030
	3) Moderate problems	0.042	0.056	0.049	0.109	0.082
	4) Severe problems	0.139	0.169	0.129	0.404	0.244
	5) Unable to	0.224	0.260	0.209	0.612	0.356

Appendix 6.4 eGFR Slope Analysis – SP Model

The mixed model for eGFR slope includes subject level random effects for intercept and slope (S_{0i} and S_{1i}). The random effect for slope is shared with the Weibull model (a frailty term) after being scaled by a fixed effect parameter γ_1 which is estimated when fitting the model. For instance, a negative value of γ_1 would indicate that participants with relatively fast decline in eGFR after conditioning on treatment and model covariates are also more likely to die earlier (again, after conditioning on treatment and covariates). The SP model is specified with the following two sub-models for subject i, one longitudinal mixed model for eGFR at time j, Y_{ij}, and one survival model for the hazard function $h_{\text{i}}(t)$ for all-cause death:

$$\begin{cases} Y_{ij} = \beta_0 + \beta_2 t_{ij} + \beta_3 t_{ij} X_i + \beta_4 Z_{i1} + \beta_5 Z_{i2} + \beta_6 Z_{i3} + \beta_7 Z_{i4} + \beta_8 Z_{i5} + \beta_9 Z_{i6} + \\ \qquad \beta_{10} t_{ij} Z_{i1} + \beta_{11} t_{ij} Z_{i2} + S_{0i} + S_{1i} t_{ij} + \varepsilon_{ij} \\ h_{\text{i}}(t) = h_0(t) exp(\alpha_0 + \alpha_1 X_i + \alpha_2 Z_{i1} + \alpha_3 Z_{i2} + \alpha_4 Z_{i3} + \alpha_5 Z_{i4} + \alpha_6 Z_{i5} + \alpha_7 Z_{i6} + \gamma_1 S_{1i}) \end{cases}$$

Where

- t_{ij} is time indicating the year since first dose of study treatment for subject i at their jth eGFR assessment
- X_i is the treatment indicator, 0 for placebo and 1 for ION-682884
- Z_{i1} is the baseline standardized eGFR
- Z_{i2} is the baseline standardized log-transformed UACR
- Z_{i3} is the baseline NYHA Classification
- Z_{i4} is the baseline ATTR mutation status
- Z_{i5} is the baseline 6MWT distance
- Z_{i6} is the baseline tafamidis use
- S_{0i}, S_{1i} are subject-specific random intercept and slope random variables, assumed to follow a bivariate normal distribution with means 0 and covariance matrix $\mathbf{\Psi} = \begin{bmatrix} \sigma_0^2 & \sigma_{01} \\ \sigma_{01} & \sigma_1^2 \end{bmatrix}$
- ε_{ij} is the random error term, assumed to be independently normally distributed random variables with mean 0 and variance σ_e^2
- $h_0(t)$ is the baseline hazard function. Time to death is assumed to follow a Weibull distribution
- α_1 is the log hazard ratio for the rate of all-cause death, ION-682884 vs. placebo
- γ_1 describes the relationship between longitudinal eGFR slope and time to all-cause death

The SP model parameters are estimated by maximizing the joint likelihood of the following sub-models.

Log-likelihood of the longitudinal sub-model is:

$$l_Y = \sum_{i=1}^{N}\sum_{j=1}^{n_i}\left[\left(1 - I_{ij}\right)\left[-\frac{1}{2}\frac{\left(y_{ij} - \mu_{ij}\right)^2}{\sigma_e^2} - \frac{1}{2}\log\left(\sigma_e^2\right)\right]\right]$$

Where

- $I_{ij} = 0$ for eGFR records; $I_{ij} = 1$ otherwise
- $\mu_{ij} = \beta_0 + \beta_2 t_{ij} + \beta_3 t_{ij} X_i + \beta_4 Z_{i1} + \beta_5 Z_{i2} + \beta_6 Z_{i3} + \beta_7 Z_{i4} + \beta_8 Z_{i5} + \beta_9 Z_{i6} + \beta_{10} t_{ij} Z_{i1} + \beta_{11} t_{ij} Z_{i2} + S_{0i} + S_{1i} t_{ij}$
- n_i is the number of post-Baseline eGFR measurements recorded for the ith subject

Log-likelihood of the survival sub-model is:

$$l_T = \sum_{i=1}^{N}\sum_{j=1}^{n_i} I_{ij}\left[D_i \log(H_i) - (L_i T_i)^{\Gamma}\right]$$

Where

- $D_i = 0$ if censored; $D_i = 1$ otherwise
- $T_i =$ time to all-cause death or censoring
- $H_i = \Gamma L_i^{\Gamma} T_i^{\Gamma - 1}$
- $L_i = exp(\alpha_0 + \alpha_1 X_i + \alpha_2 Z_{i1} + \alpha_3 Z_{i2} + \alpha_4 Z_{i3} + \alpha_5 Z_{i4} + \alpha_6 Z_{i5} + \alpha_7 Z_{i6} + \gamma_1 S_{1i})$
- Γ is the shape parameter of the Weibull survival model

Joint log-likelihood of the sub-models is:

$$l = l_Y + l_T$$

The SP model will be performed using the SAS procedure PROC NLMIXED.

Approval Signatures

Document Name: Statistical Analysis Plan 22 Apr 2026 243920_TT2

Document Number: [redacted]

Parexel Version Number:

System Version Number: 1 .0

Please note that the Document Date and the Document Approval Date of Signature may potentially have a mismatch of plus or minus one (1) day based on the location time zone of the User versus the system

Document Approvals

Reason for signing: Approved	[redacted]

Version:	1
Version Date:	23 Apr 2026
Title:	ION-682884-CS2 Statistical Analysis Plan - Version 6.0

APPROVALS:

[redacted]
