# Supplementary material for: Eplontersen with and without background transthyretin stabilizers in transthyretin amyloid cardiomyopathy: secondary analysis of a phase 3, randomized controlled trial
Source: Nat Med. 2026 Aug 30;32(11):4047–55. doi: 10.1038/s41591-026-04670-6 (PMC13768182; doi:10.1038/s41591-026-04670-6)
Supplement: Supplementary file 2 [file 41591_2026_4670_MOESM2_ESM.pdf]

nature portfolio

Corresponding author(s): Pablo García-Pavía

Last updated by author(s): 12-8-2026

Reporting Summary

Nature Portfolio wishes to improve the reproducibility of the work that we publish. This form provides structure for consistency and transparency in reporting. For further information on Nature Portfolio policies, see our Editorial Policies and the Editorial Policy Checklist.

Statistics

For all statistical analyses, confirm that the following items are present in the figure legend, table legend, main text, or Methods section.

n/a	Confirmed	
☐	☒	The exact sample size (*n*) for each experimental group/condition, given as a discrete number and unit of measurement
☒	☐	A statement on whether measurements were taken from distinct samples or whether the same sample was measured repeatedly
☐	☒	The statistical test(s) used AND whether they are one- or two-sided<br>*Only common tests should be described solely by name; describe more complex techniques in the Methods section.*
☐	☒	A description of all covariates tested
☐	☒	A description of any assumptions or corrections, such as tests of normality and adjustment for multiple comparisons
☐	☒	A full description of the statistical parameters including central tendency (e.g. means) or other basic estimates (e.g. regression coefficient) AND variation (e.g. standard deviation) or associated estimates of uncertainty (e.g. confidence intervals)
☐	☒	For null hypothesis testing, the test statistic (e.g. *F*, *t*, *r*) with confidence intervals, effect sizes, degrees of freedom and *P* value noted<br>*Give P values as exact values whenever suitable.*
☒	☐	For Bayesian analysis, information on the choice of priors and Markov chain Monte Carlo settings
☒	☐	For hierarchical and complex designs, identification of the appropriate level for tests and full reporting of outcomes
☒	☐	Estimates of effect sizes (e.g. Cohen's *d*, Pearson's *r*), indicating how they were calculated

Our web collection on statistics for biologists contains articles on many of the points above.

Software and code

Policy information about availability of computer code

Data collection	Data were collected via web-based electronic case report forms.
Data analysis	Statistical analyses were performed with SAS software, version 9.3 or higher (SAS Institute, Cary, North Carolina, USA) and STATA, version 19 (College Station, Texas, USA).

For manuscripts utilizing custom algorithms or software that are central to the research but not yet described in published literature, software must be made available to editors and reviewers. We strongly encourage code deposition in a community repository (e.g. GitHub). See the Nature Portfolio guidelines for submitting code & software for further information.

Data

Policy information about availability of data

All manuscripts must include a data availability statement. This statement should provide the following information, where applicable:

- Accession codes, unique identifiers, or web links for publicly available datasets
- A description of any restrictions on data availability
- For clinical datasets or third party data, please ensure that the statement adheres to our policy

Data requests from qualified researchers will be considered once all three of the following criteria are met: (1) 12 months from marketing approval of the study drug in both the United States and European Union; (2) 18 months from conclusion of the study; and (3) 6 months from the publication of this article. Data access is

managed to uphold scientific rigor and merit, protect patient data, and avoid potential conflicts of interest or competitive concerns. Data requests are reviewed and processed in a timely manner. For additional information, conditions of data access, and contact details for access requests, visit https://vivli.org/ourmembers/ionis.

Research involving human participants, their data, or biological material

Policy information about studies with human participants or human data. See also policy information about sex, gender (identity/presentation), and sexual orientation and race, ethnicity and racism.

Reporting on sex and gender	Data on sex (male/female) were collected via case report form, based on self-reporting. Ns (%) of male/female participants are reported in baseline characteristics tables, including by treatment received and by whether they reported use of TTR stabilisers at baseline. The proportion of female patients enrolled for CARDIO-TTRansform was consistent with the reported demographics of patients with ATTR-CM; however, the heterogeneity of treatment effect and TTR stabiliser use at baseline by sex, was not evaluated due to the small sample size available for female participants.
Reporting on race, ethnicity, or other socially relevant groupings	Data on race (Asian, Black or African American, Multiple, Other, White) were collected via case report form, based on self-reporting. The n (%) for each racial category are reported in baseline characteristics tables, including by treatment received and by whether they reported use of TTR stabilisers at baseline.
Population characteristics	A total of 1432 patients aged 18–90 years with a diagnosis of ATTR-CM (wild-type or variant), classified as being in New York Heart Association [NYHA] functional class I–III, and with a history of at least one heart failure-associated hospitalisation or heart failure requiring loop diuretic therapy, serum N-terminal prohormone of brain natriuretic peptide concentration ≥600 pg/ml (or ≥1200 pg/ml if in atrial fibrillation) and the ability to walk ≥100 metres on the 6-minute walk test (6MWT) were were randomised and received either eplontersen or placebo. Median age was 77 years, 91% were male. A total of 15% had variant ATTR-CM. The proportion in NYHA functional class I, II, and II were 13%, 70% and 17% NYHA, respectfully. A total of 819 patients were receiving a TTR stabilisers at baseline.
Recruitment	Patients were recruited at 130 hospital/health-care sites across 20 countries. All participants were screened and enrolled by health care providers rather than self-selected and required to meet the studies inclusion and exclusion criteria, which included a requirement that patients had no heart failure associated hospitalisations within the past 4 weeks or malignancy within the past five years. Effects of selection bias were anticipated to be minimal given the studies randomised design.
Ethics oversight	The trial protocol was approved by institutional review boards or ethics committees at each participating site. During the trial an independent Data and Safety Monitoring Board evaluated unblinded data.

Note that full information on the approval of the study protocol must also be provided in the manuscript.

Field-specific reporting

Please select the one below that is the best fit for your research. If you are not sure, read the appropriate sections before making your selection.

☒ Life sciences ☐ Behavioural & social sciences ☐ Ecological, evolutionary & environmental sciences

For a reference copy of the document with all sections, see nature.com/documents/nr-reporting-summary-flat.pdf

Life sciences study design

All studies must disclose on these points even when the disclosure is negative.

Sample size	The overall CARDIO-TTRansform sample size was determined with the following assumptions: an overall cardiovascular death rate of 8.7%, the occurrence of 0.476 cardiovascular clinical events per patient for the placebo group during the double-blind period, a significance level (alpha) of 0.05 (two-sided test), and a treatment duration up to 140 weeks. Sample sizes for the subgroups for these analyses were determined based on the reported use of TTR stabilisers at baseline, which was a randomisation stratification factor in the overall CARDIO-TTRansform population.
Data exclusions	Only data from patients with protocol violations that prevented reporting in accordance with Good Clinical Practice guidelines were excluded.
Replication	Outcomes herein were based on collected clinical data and are not repeatable, directly reproducible due to the nature of the trial.
Randomization	Patients were randomised 1:1, and stratified by NYHA functional class (I/II vs III), TTR variant status (variant vs wild-type), 6MWT distance (≤350 vs >350 m), and TTR stabiliser use at baseline to receive eplontersen 45 mg or placebo.
Blinding	CARDIO-TTRansform was a double-blind, placebo-controlled trial with a double-blind period lasting 140 weeks. Investigators collected the data herein, an independent Data and Safety Monitoring Board evaluated unblinded data during the trial, and an independent and blinded-to-treatment Clinical Event Committee adjudicated all deaths and cardiovascular events

Reporting for specific materials, systems and methods

We require information from authors about some types of materials, experimental systems and methods used in many studies. Here, indicate whether each material, system or method listed is relevant to your study. If you are not sure if a list item applies to your research, read the appropriate section before selecting a response.

Materials & experimental systems

n/a	Involved in the study
☒	☐ Antibodies
☒	☐ Eukaryotic cell lines
☒	☐ Palaeontology and archaeology
☒	☐ Animals and other organisms
☐	☒ Clinical data
☒	☐ Dual use research of concern
☒	☐ Plants

Methods

n/a	Involved in the study
☒	☐ ChIP-seq
☒	☐ Flow cytometry
☒	☐ MRI-based neuroimaging

Clinical data

Policy information about clinical studies

All manuscripts should comply with the ICMJE guidelines for publication of clinical research and a completed CONSORT checklist must be included with all submissions.

Clinical trial registration: NCT04136171

Study protocol: The protocol and statistical analysis plan are available at NEJM.org

Data collection: From March 2020 through July 2023, 2337 patients were screened and 1438 were randomized. Data collection cut off was 10 June 2026. Investigators collected the data at 130 sites across 20 countries. The list of Study Investigators by Country (Centers) are available at NEJM.org

Outcomes: Outcomes included a cardiovascular (CV) death and recurrent CV events up to week 140; changes from baseline in the 6-minute walk test distance at week 140; changes from baseline in the Kansas City Cardiomyopathy Questionnaire overall summary score at week 140; recurrent cardiovascular (CV) events up to week 140; all-cause death up to Week 160 and Week 140; and CV death up to week 160 and week 140 for eplontersen vs placebo in patients with and without baseline TTR stabilisers. The rationale for the predefined outcomes are available at Masri A, et al. Circ Heart Fail. 19, e014205 (2026). All-cause and cardiovascular mortality end points were tested first up to Week 160, and then up to Week 140; the former time point was selected to allow more events to be included to improve the power and precision of the treatment effect estimates. Ascertainment of vital status up to Week 140 was obtained from the double-blind period, and vital status between Week 140 and Week 160 was obtained from fatal events in either the open-label extension study or the 20-week post-treatment evaluation period.

Plants

Seed stocks: N/A

Novel plant genotypes: N/A

Authentication: N/A
